# Immunogenicity, Safety, and Tolerability of V114, a 15-Valent Pneumococcal Conjugate Vaccine, in Immunocompetent Adults Aged 18–49 Years With or Without Risk Factors for Pneumococcal Disease: A Randomized Phase 3 Trial (PNEU-DAY)

**DOI:** 10.1093/ofid/ofab605

**Published:** 2021-12-18

**Authors:** Laura L Hammitt, Dean Quinn, Ewa Janczewska, Francisco J Pasquel, Richard Tytus, K Rajender Reddy, Katia Abarca, Ilsiyar M Khaertynova, Ron Dagan, Jennifer McCauley, Kyeongmi Cheon, Alison Pedley, Tina Sterling, Gretchen Tamms, Luwy Musey, Ulrike K Buchwald

**Affiliations:** 1 Johns Hopkins Bloomberg School of Public Health, Baltimore, Maryland, USA; 2 P3 Research, Wellington Clinical Trial Research Unit, Wellington, New Zealand; 3 The School of Health Sciences in Bytom, Medical University of Silesia, Katowice, Poland; 4 Emory University School of Medicine, Atlanta, Georgia, USA; 5 McMaster University, Ontario, Canada; 6 Perelman School of Medicine, University of Pennsylvania, Philadelphia, Pennsylvania, USA; 7 Escuela de Medicina, Pontificia Universidad Catolica de Chile, Santiago, Chile; 8 Department of Infectious Diseases, Kazan State Medical Academy, Kazan, Russia; 9 Ben-Gurion University, Beer-Sheva, Israel; 10 Merck & Co, Inc, Kenilworth, New Jersey, USA

**Keywords:** pneumococcal vaccine, V114, 15-valent PCV, PCV13, PPSV23

## Abstract

**Background:**

Adults with certain medical and behavioral factors are at increased risk for pneumococcal disease (PD). Sequential vaccination with 13-valent pneumococcal conjugate vaccine (PCV13) followed by 23-valent pneumococcal polysaccharide vaccine (PPSV23) is recommended for at-risk adults in some countries.

**Methods:**

This phase 3 trial evaluated the safety, tolerability, and immunogenicity of sequential administration of either V114 (a 15-valent PCV containing serotypes 1, 3, 4, 5, 6A, 6B, 7F, 9V, 14, 18C, 19A, 19F, 22F, 23F, and 33F) or PCV13, followed 6 months later by PPSV23, in immunocompetent adults aged 18–49 years with or without predefined risk factors for PD (NCT03547167). Overall, 1515 participants were randomized 3:1 to receive either V114 or PCV13, followed by PPSV23.

**Results:**

Most common solicited adverse events (AEs) following administration of V114 or PCV13 as well as PPSV23 were injection-site pain and fatigue. The proportion of participants with AEs was comparable in both groups. V114 and PCV13 were immunogenic based on opsonophagocytic activity (OPA) geometric mean titers (GMTs) 30 days postvaccination for all serotypes contained in each respective vaccine. OPA GMTs to the 2 unique serotypes in V114 were robust in the V114 group. PPSV23 was immunogenic for all 15 serotypes contained in V114 in both vaccination groups, including 22F and 33F.

**Conclusions:**

V114 administered alone or sequentially with PPSV23 is well tolerated and immunogenic for all 15 serotypes, including those not contained in PCV13, in immunocompetent adults aged 18–49 years with or without certain medical or behavioral risk factors for PD.

**Clinical Trials Registration:**

NCT03547167 and EudraCT 2017-004915-38.

*Streptococcus pneumoniae* is associated with significant global burden of disease and is a leading cause of hospitalization, morbidity, and mortality in all age groups [1–3]. Despite the inclusion of pneumococcal vaccines in many national immunization programs [4], incidence of pneumococcal disease (PD) and associated mortality remain high [2]. Chronic conditions (eg, chronic heart, liver, and lung disease, and diabetes mellitus), as well as behavioral factors (eg, smoking and alcoholism), increase the risk of PD regardless of age and have been shown to be predictors of hospitalization and mortality [5–10]. Stacking of these risk factors and advanced disease stages is associated with higher incidence of PD [8–12], approaching incidence rates in immunocompromised individuals [12]. In addition, disparities in income and access to healthcare contribute to an increased burden of PD in some communities, in which environmental, socioeconomic, and housing conditions, as well as a high prevalence of chronic medical conditions, contribute to a higher burden of PD compared with the general population [1, 13].

Pneumococcal vaccines are designed to cover serotypes most frequently associated with severe PD [14] and typically only protect against serotypes included in the vaccine [15]. The 23-valent pneumococcal polysaccharide vaccine (PPSV23) is immunogenic against 23 serotypes that cause 60%–76% of ­invasive disease, with well-described safety and effectiveness profiles, and is indicated in adults aged ≥50 years and persons aged ≥2 years at increased risk for PD [16, 17]. The 13-valent pneumococcal conjugate vaccine (PCV13) covers 13 serotypes and is indicated in persons aged ≥6 weeks [18]. The United States (US) Advisory Committee on Immunization Practices (ACIP) recommends vaccination of at-risk immunocompetent individuals aged 19–64 years who have 1 or more risk factor with a single dose of PPSV23 [19]. In other countries, pneumococcal vaccination recommendations for this population include PCV13 alone or in sequence with PPSV23 [4, 20], as some evidence suggests that functional antibody titers induced by pneumococcal conjugate vaccines (PCVs) are generally higher than those induced by pneumococcal polysaccharide vaccines [21, 22]. Of the current pneumococcal vaccination regimen recommendations, sequential vaccination with PCV13 and PPSV23 offers the broadest serotype coverage.

Since the introduction of PCVs, non-PCV serotypes have emerged as increasingly significant causes of PD across age groups [23, 24], creating a need for PCVs with broader serotype coverage. V114 is a 15-valent PCV for the prevention of invasive and noninvasive PD. It contains 13 serotypes included in PCV13 (1, 3, 4, 5, 6A, 6B, 7F, 9V, 14, 18C, 19A, 19F, and 23F), as well as serotypes 22F and 33F, which have emerged as leading causes of invasive PD (IPD) following the introduction of PCVs [25–27]. In phase 2 studies, V114 was well tolerated and immunogenic in healthy pediatric and adult participants [28, 29]. The purpose of this study was to investigate the safety and immunogenicity of V114 alone, as well as in sequence with PPSV23, in immunocompetent adults aged 18–49 years, including those at increased risk for PD.

## METHODS

### Study Design

This was a phase 3, multicenter, randomized, double-blind, active comparator-controlled study to evaluate the safety, tolerability, and immunogenicity of V114 in pneumococcal vaccine-naive immunocompetent adults aged 18–49 years with or without risk factors for PD (protocol V114-017; NCT03547167 and EudraCT 2017-004915-38). The study was conducted from July 2018 through July 2020 at 79 sites worldwide (Supplementary Table 1). V114 and PCV13 were administered in a blinded fashion and PPSV23 was administered open-label. The study was conducted in accordance with the ethical principles originating from the Declaration of Helsinki, Good Clinical Practice requirements, and applicable country and/or local statutes and regulations regarding independent ethics committee review, informed consent, and the protection of human participants in biomedical research. The study protocol and any amendments, information provided to participants, and any recruitment materials were reviewed and approved by the appropriate institutional review boards and regulatory agencies. Written informed consent was obtained from each participant prior to any study procedure.

Approximately 1500 participants were randomized in a 3:1 ratio to receive a single dose of V114 or PCV13 on Day 1, followed by a single dose of PPSV23 at Month 6. The sample size and randomization ratio were based on attaining an adequate number of participants exposed to V114 across different age categories, and a sufficient number of participants in each risk factor for PD based on its prevalence in the targeted age population. This study was designed as a descriptive study and was not statistically powered to demonstrate differences in opsonophagocytic activity (OPA) or immunoglobulin G (IgG) responses between recipients of V114 and PCV13.

### Participants

Immunocompetent adults aged 18–49 years with or without risk factors for PD were eligible for the study if they were verified as pneumococcal vaccine naive by medical history and record review. The Johns Hopkins Center for American Indian Health (CAIH) enrolled Native American adults with and without risk factors for PD (as defined below) at clinical sites located in the southwest region of the US; non-CAIH sites enrolled participants with ≥1 risk factor for PD. Protocol-defined inclusion criteria had to be met for the risk factors of diabetes mellitus, chronic liver disease, chronic lung disease, chronic heart disease, and current tobacco use (Supplementary Table 2).

Randomization was stratified based on site (CAIH vs non-CAIH site) and type/number of predefined baseline risk factors as well as higher self-reported alcohol use at screening based on a score of ≥5 via the Alcohol Use Disorder Identification Test Concise (AUDIT-C) test [30].

### Vaccines and Administration

V114 (VAXNEUVANCE; Merck Sharp & Dohme Corp, a subsidiary of Merck & Co, Inc, Kenilworth, New Jersey) is a 15-valent PCV. Each 0.5-mL dose contains 2 µg of pneumococcal capsular polysaccharide from serotypes 1, 3, 4, 5, 6A, 7F, 9V, 14, 18C, 19A, 19F, 23F, 22F, and 33F, and 4 µg of serotype 6B, all conjugated to CRM_197_ carrier protein and adjuvanted with 125 µg of aluminum phosphate.

PCV13 (Prevnar 13, Wyeth LLC, marketed by Pfizer, New York, New York) is a 13-valent PCV. Each 0.5-mL dose contains 2.2 µg of pneumococcal capsular polysaccharide from serotypes 1, 3, 4, 5, 6A, 7F, 9V, 14, 18C, 19A, 19F, and 23F, and 4.4 µg of serotype 6B, conjugated to CRM_197_ carrier protein and adjuvanted with 125 µg of aluminum phosphate.

PPSV23 (PNEUMOVAX 23, Merck Sharp & Dohme Corp, a subsidiary of Merck & Co, Inc, Kenilworth, New Jersey) is a 23-valent pneumococcal polysaccharide vaccine. Each dose of PPSV23 contains 25 µg of pneumococcal capsular polysaccharide from serotypes 1, 2, 3, 4, 5, 6B, 7F, 8, 9N, 9V, 10A, 11A, 12F, 14, 15B, 17F, 18C, 19A, 19F, 20, 22F, 23F, and 33F.

V114 and PCV13 were supplied as sterile suspensions and PPSV23 was supplied as a sterile solution. All study vaccines were supplied in prefilled syringes and stored at 2°C–8°C. A 0.5-mL dose of V114 or PCV13 and PPSV23 was administered intramuscularly.

### Safety Assessments

Adverse events (AEs) experienced following receipt of each study vaccine were self-recorded using a Vaccination Report Card and subsequently assessed by investigators. Injection-site reactions (erythema, swelling, and pain) and systemic AEs (muscle pain/myalgia, joint pain/arthralgia, headache, and fatigue) were solicited following each vaccination; nonsolicited injection-site and systemic AEs were also recorded. Information for serious adverse events (SAEs) and deaths, regardless of attribution, were collected from the time of signed consent through the end of study.

All solicited and nonsolicited events were assessed by investigators for severity per the US Food and Drug Administration guidance on toxicity grading in preventive vaccine clinical trials [31]. All injection-site AEs were considered vaccine related. For systemic AEs, relatedness to study vaccine was assessed by investigators.

### Immunogenicity Assessments

Serum samples were drawn prevaccination with PCV (Day 1), 30 days post–PCV vaccination (Day 30), prevaccination with PPSV23 (Month 6), and 30 days post–PPSV23 vaccination (Month 7) to assess immune responses. Functional antibodies, which are thought to correlate best with protection from PD in adults [32], were measured using serotype-specific opsonophagocytic killing activity using a validated microcolony multiplexed opsonophagocytic assay [33]. Serotype-specific pneumococcal capsular polysaccharide IgG antibodies were evaluated using a validated multiplexed electrochemiluminescence assay [34].

### Study Endpoints and Statistical Analysis

#### Analysis Populations

Safety analyses were conducted on the all-participants-as-treated population, which comprised all randomized participants who received the relevant study vaccine for the timepoint of interest.

The per-protocol population was the primary population used for the analysis of immunogenicity data and comprised all randomized participants without protocol deviations that could substantially affect the results of immunogenicity endpoints.

#### Safety Endpoints and Statistical Analyses

The primary safety endpoints were assessed following administration of V114 or PCV13, and the secondary safety endpoints were assessed following administration of PPSV23. The safety endpoints included the proportions of participants with: (1) solicited injection-site AEs from days 1–5 postvaccination, (2) solicited systemic AEs from days 1–14 postvaccination, and (3) any AE or SAE, including vaccine-related SAEs. Point estimates and within-group 95% confidence intervals (CIs) were provided for each treatment group separately for the proportions of participants with AEs using the method proposed by Clopper and Pearson, without multiplicity adjustments [35].

#### Immunogenicity Endpoints and Statistical Analyses

The primary immunogenicity objective was to evaluate serotype-specific OPA geometric mean titers (GMTs) for the 15 serotypes included in V114 at Day 30 within each vaccination group separately.

Secondary immunogenicity endpoints included observed serotype-specific IgG geometric mean concentrations (GMCs) at Day 30, OPA GMTs and IgG GMCs at Month 6 and Month 7, as well as geometric mean fold rises (GMFRs) and proportions of participants with a ≥4-fold rise in OPA and IgG responses immediately prior to, and following, vaccination within each vaccination group separately.

The point estimates and within-group 95% CIs were computed without multiplicity adjustment based on t-distribution and the method proposed by Clopper and Pearson [35] for continuous and dichotomous endpoints, respectively.

Exploratory analyses compared OPA GMTs and IgG GMCs (via estimation of the ratio) between vaccination groups using serotype-specific constrained longitudinal data analysis (cLDA) models [36].

## RESULTS

### Study Population

**Figure 1.**
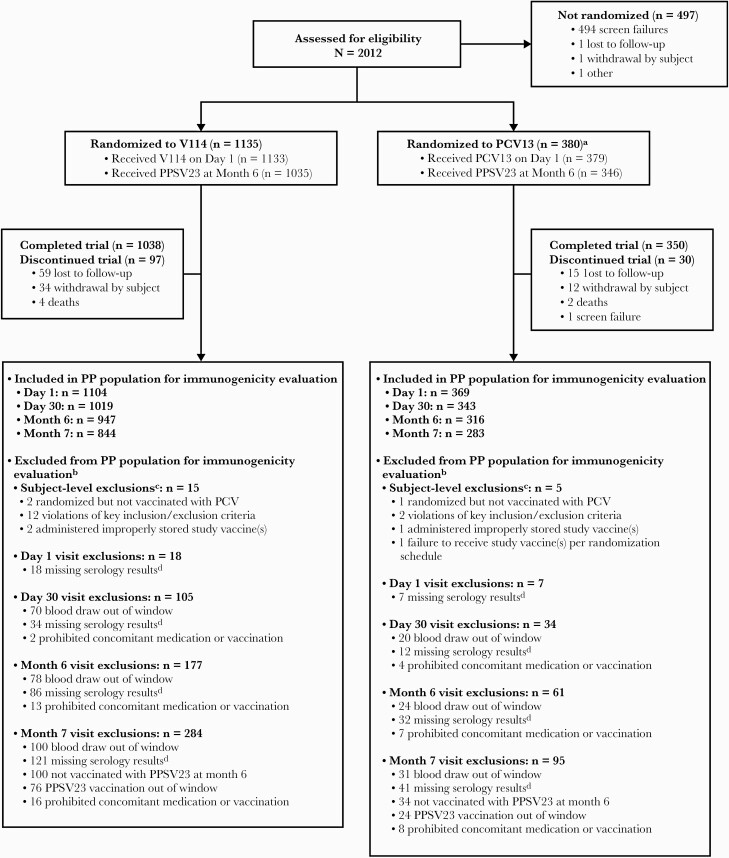
Participant disposition. Percentages are calculated based on the number of subjects randomized unless otherwise noted. Participants could have been considered to complete the study without receipt of PPSV23. ^a^One participant in the PCV13 group incorrectly received V114 and was included in the V114 group for safety analyses. ^b^Subjects may have >1 reason for exclusion. Subjects are displayed in all applicable categories. ^c^Subject-level exclusions result in exclusion from analyses at all timepoints. ^d^Subjects who have missing serology results for all 15 serotypes. Reasons for missing serology results may include discontinuation prior to serum sample collection, failure to provide a serum sample, serum sample lost or damaged, and failure to receive PPSV23 prior to a subsequent serum collection. Abbreviations: PCV, pneumococcal conjugate vaccine; PCV13, 13-valent pneumococcal conjugate vaccine; PP, per-protocol; PPSV23, 23-valent pneumococcal polysaccharide vaccine; V114, 15-valent pneumococcal conjugate vaccine.

A total of 1515 participants were randomized, with 91.6% completing the study (Figure 1). The proportion of participants who discontinued and reasons for study discontinuation were generally comparable across vaccination groups. Most participants (91.2%) received PPSV23.

Demographic and baseline characteristics were generally comparable across vaccination groups (Table 1). The majority (74.8%) of participants had ≥1 risk factor for PD at screening: 54.7% had 1 risk factor, and 20.1% had ≥2 risk factors. The number of risk factors increased with increasing age in both vaccination groups. Most common individual risk factors in patients with a single risk factor were tobacco use (14.6%), chronic lung disease (14.3%), and diabetes mellitus (13.8%). Approximately 25% of participants had no risk factors, all of whom were enrolled at CAIH sites.

**Table 1.** Participant Baseline Characteristics

Characteristic	V114		PCV13		Total	
	No.	(%)	No.	(%)	No.	(%)
Vaccinated participants	1133	(100.0)	379	(100.0)	1512	(100.0)
Sex						
Female	581	(51.3)	200	(52.8)	781	(51.7)
Male	552	(48.7)	179	(47.2)	731	(48.3)
Age, y						
18–29	329	(29.0)	105	(27.7)	434	(28.7)
30–39	351	(31.0)	112	(29.6)	463	(30.6)
40–49	453	(40.0)	162	(42.7)	615	(40.7)
Mean (range)	35.8	(18–49)	35.8	(18–49)	35.8	(18–49)
Race						
White	580	(51.2)	191	(50.4)	771	(51.0)
American Indian/Alaska Native	445	(39.3)	148	(39.1)	593	(39.2)
Black	43	(3.8)	18	(4.7)	61	(4.0)
Native Hawaiian/other Pacific Islander	33	(2.9)	11	(2.9)	44	(2.9)
Asian	15	(1.3)	8	(2.1)	23	(1.5)
Multiple	17	(1.5)	3	(0.8)	20	(1.3)
Ethnicity						
Not Hispanic/Latino	982	(86.7)	337	(88.9)	1319	(87.2)
Hispanic/Latino	135	(11.9)	39	(10.3)	174	(11.5)
Not reported	8	(0.7)	1	(0.3)	9	(0.6)
Unknown	8	(0.7)	2	(0.5)	10	(0.7)
Subjects by enrollment at CAIH/non-CAIH sites						
CAIH sites	439	(38.7)	148	(39.1)	587	(38.8)
Non-CAIH sites	694	(61.3)	231	(60.9)	925	(61.2)
Subjects by risk factors^a^^,^^b^						
With no risk factor^c^	285	(25.2)	96	(25.3)	381	(25.2)
With single risk factor	620	(54.7)	207	(54.6)	827	(54.7)
Tobacco use	165	(14.6)	56	(14.8)	221	(14.6)
Chronic lung disease	163	(14.4)	53	(14.0)	216	(14.3)
Diabetes mellitus	157	(13.9)	51	(13.5)	208	(13.8)
Chronic heart disease	57	(5.0)	20	(5.3)	77	(5.1)
Alcohol consumption^c^^,^^d^	50	(4.4)	18	(4.7)	68	(4.5)
Chronic liver disease	28	(2.5)	9	(2.4)	37	(2.4)
With ≥2 risk factors	228	(20.1)	76	(20.1)	304	(20.1)
Subjects by risk factors and age group^b^						
18–29 y	329	(29.0)	105	(27.7)	434	(28.7)
With no risk factor^c^	131	(11.6)	49	(12.9)	180	(11.9)
With single risk factor	147	(13.0)	40	(10.6)	187	(12.4)
With ≥2 risk factors	51	(4.5)	16	(4.2)	67	(4.4)
30–39 y	351	(31.0)	112	(29.6)	463	(30.6)
With no risk factor^c^	99	(8.7)	26	(6.9)	125	(8.3)
With single risk factor	194	(17.1)	63	(16.6)	257	(17.0)
With ≥2 risk factors	58	(5.1)	23	(6.1)	81	(5.4)
40–49 y	453	(40.0)	162	(42.7)	615	(40.7)
With no risk factor^c^	55	(4.9)	21	(5.5)	76	(5.0)
With single risk factor	279	(24.6)	104	(27.4)	383	(25.3)
With ≥2 risk factors	119	(10.5)	37	(9.8)	156	(10.3)

Table includes all vaccinated participants.

Abbreviations: CAIH, Center for American Indian Health; PCV13, 13-valent pneumococcal conjugate vaccine; V114, 15-valent pneumococcal conjugate vaccine.

Subject is counted for each applicable row and column.

Actual strata assignments were used in the summaries of risk factors.

All subjects with no risk factor and subjects with a single risk factor of alcohol consumption were enrolled at CAIH sites.

The risk factor of alcohol consumption is defined as an Alcohol Use Disorders Identification Test-Consumption (AUDIT-C) score ≥5.

### Safety

#### Following PCV Vaccination

Approximately 80% of study participants in both vaccination groups experienced at least 1 AE (overall and solicited) following vaccination with V114 or PCV13 (Table 2). The proportions of participants who experienced SAEs were low in the V114 and PCV13 groups in the 6 months following the receipt of PCV (4.3% and 3.2%, respectively). No discontinuations or deaths deemed by investigators to be related to study vaccine occurred in either group.

**Table 2.** Summary of Adverse Events After Vaccination With V114 or PCV13

Adverse Event	V114 (n = 1134)			PCV13 (n = 378)		
	No.	(%)	(95% CI)^a^	No.	(%)	(95% CI)^a^
Any AE	960	(84.7)	(82.4–86.7)	312	(82.5)	(78.3–86.2)
Injection site	893	(78.7)	…	272	(72.0)	…
Systemic	707	(62.3)	…	238	(63.0)	…
Any vaccine-related AE^b^	925	(81.6)	(79.2–83.8)	293	(77.5)	(73.0–81.6)
Injection site	893	(78.7)	…	272	(72.0)	…
Systemic	555	(48.9)	…	176	(46.6)	…
Any SAE	49	(4.3)	(3.2–5.7)	12	(3.2)	(1.7–5.5)
Any vaccine-related SAE^b^	0	(0)	(.0–.3)	0	(0)	(.0–.8)
Deaths	3	(0.3)	(.1–.8)	2	(0.5)	(.1–1.9)
Solicited injection-site AEs (day 1 to day 5)						
Injection-site pain	860	(75.8)	(73.2–78.3)	260	(68.8)	(63.8–73.4)
Injection-site swelling	246	(21.7)	(19.3–24.2)	84	(22.2)	(18.1–26.8)
Injection-site erythema	171	(15.1)	(13.0–17.3)	53	(14.0)	(10.7–17.9)
Solicited systemic AEs (day 1 to day 14)						
Fatigue	389	(34.3)	(31.5–37.1)	139	(36.8)	(31.9–41.9)
Myalgia	327	(28.8)	(26.2–31.6)	100	(26.5)	(22.1–31.2)
Headache	300	(26.5)	(23.9–29.1)	94	(24.9)	(20.6–29.5)
Arthralgia	144	(12.7)	(10.8–14.8)	44	(11.6)	(8.6–15.3)

Table includes all participants vaccinated with V114 or PCV13. One participant in the PCV13 group incorrectly received V114 and was included in the V114 group for safety analyses. Reported AEs include nonserious AEs within 14 days of vaccination with V114 or PCV13 and SAEs occurring Day 1 through Month 6.

Abbreviations: AE, adverse event; CI, confidence interval, PCV13, 13-valent pneumococcal conjugate vaccine; SAE, serious adverse event; V114, 15-valent pneumococcal conjugate vaccine.

Estimated CIs are calculated based on the exact binomial method proposed by Clopper and Pearson and are provided in accordance with the statistical analysis plan.

Deemed related to study vaccine by investigators.

**Figure 2.**
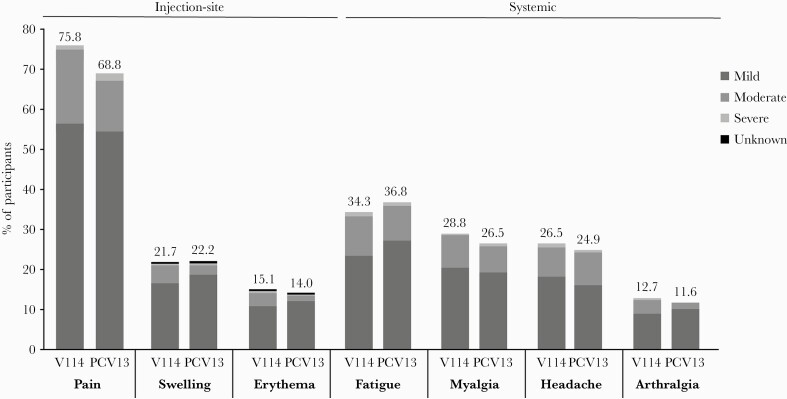
Proportion of participants with solicited AEs after vaccination with V114 or PCV13, by severity. Solicited AEs collected postvaccination with V114 (n = 1134) or PCV13 (n = 378). One participant in the PCV13 group incorrectly received V114 and was included in the V114 group for safety analyses. Every subject is counted a single time for each applicable specific AE. Injection-site events were solicited from day 1 to day 5 following vaccination. Systemic events were solicited from day 1 to day 14 following vaccination. The height of the stacked bar represents the total percentage of participants reporting the AE. Per the US Food and Drug Administration guidance on toxicity grading in preventive vaccine clinical trials, the severity of AEs was categorized as mild (grade 1), moderate (grade 2), severe (grade 3), or potentially life-threatening (grade 4). For solicited injection-site erythema and injection-site swelling, mild events measured >0 to ≤5 cm, moderate events measured >5 to ≤10 cm, and severe events measured >10 cm. For injection-site pain and systemic AEs, the severity was defined by the degree to which the event affected daily activities and the use of medications for pain relief [31]. The severity grades (mild, moderate, or severe) within the bar indicate the proportion of the total attributed to each respective category. Abbreviations: AE, adverse event; PCV13, 13-valent pneumococcal conjugate vaccine; V114, 15-valent pneumococcal conjugate vaccine.

The proportions of participants with solicited AEs were generally comparable across vaccination groups (Table 2; Figure 2). The most frequently reported solicited injection-site AE in the V114 and PCV13 groups was injection-site pain (75.8% and 68.8%, respectively). The most frequently reported solicited systemic AE was fatigue (34.3% and 36.8%, respectively). In both vaccination groups, most solicited AEs were transient (≤3 days; Supplementary Table 3) and mild in severity (Figure 2).

#### Following PPSV23 Vaccination

Approximately 76% of participants in both vaccination groups experienced at least 1 AE (overall and solicited) following vaccination with PPSV23 (Table 3). The proportion of participants who experienced SAEs was <1% in both vaccination groups in the 30 days following receipt of PPSV23. No participants died during the follow-up period for PPSV23 (Months 6–7).

**Table 3.** Summary of Adverse Events After Vaccination With PPSV23

Adverse Event	V114 (n = 1036)			PCV13 (n = 345)		
	No.	(%)	(95% CI)^a^	No.	(%)	(95% CI)^a^
Any AE	787	(76.0)	(73.2–78.5)	264	(76.5)	(71.7–80.9)
Injection-site AEs	739	(71.3)	…	241	(69.9)	…
Systemic AEs	528	(51.0)	…	179	(51.9)	…
Any vaccine-related AE^b^	766	(73.9)	(71.2–76.6)	250	(72.5)	(67.4–77.1)
Injection-site AEs	739	(71.3)	…	241	(69.9)	…
Systemic AEs	447	(43.1)	…	151	(43.8)	…
Any SAE	3	(0.3)	(.1–.8)	3	(0.9)	(.2–2.5)
Any vaccine-related SAE^b^	0	(0)	(.0–.3)	1	(0.3)	(.0–1.6)
Deaths	0	(0)	(.0–.3)	0	(0)	(.0–.9)
Solicited injection-site AEs (day 1 to day 5)						
Injection-site pain	713	(68.8)	(65.9–71.6)	231	(67.0)	(61.7–71.9)
Injection-site swelling	305	(29.4)	(26.7–32.3)	111	(32.2)	(27.3–37.4)
Injection-site erythema	234	(22.6)	(20.1–25.3)	88	(25.5)	(21.0–30.5)
Solicited systemic AEs (day 1 to day 14)						
Fatigue	312	(30.1)	(27.3–33.0)	106	(30.7)	(25.9–35.9)
Myalgia	250	(24.1)	(21.6–26.9)	88	(25.5)	(21.0–30.5)
Headache	220	(21.2)	(18.8–23.9)	73	(21.2)	(17.0–25.9)
Arthralgia	124	(12.0)	(10.1–14.1)	38	(11.0)	(7.9–14.8)

Table includes all participants vaccinated with PPSV23. One participant in the PCV13 group incorrectly received V114 and was included in the V114 group for safety analyses. Reported AEs include nonserious AEs within 14 days of vaccination with PPSV23 and SAEs occurring Month 6 (prevaccination with PPSV23) through Month 7 (30 days postvaccination with PPSV23).

Abbreviations: AE, adverse event; CI, confidence interval; PCV13, 13-valent pneumococcal conjugate vaccine; PPSV23, 23-valent pneumococcal polysaccharide vaccine; SAE, severe adverse event; V114, 15-valent pneumococcal conjugate vaccine.

Estimated Cls are calculated based on the exact binomial method proposed by Clopper and Pearson and are provided in accordance with the statistical analysis plan.

Deemed related to study vaccine by investigators.

**Figure 3.**
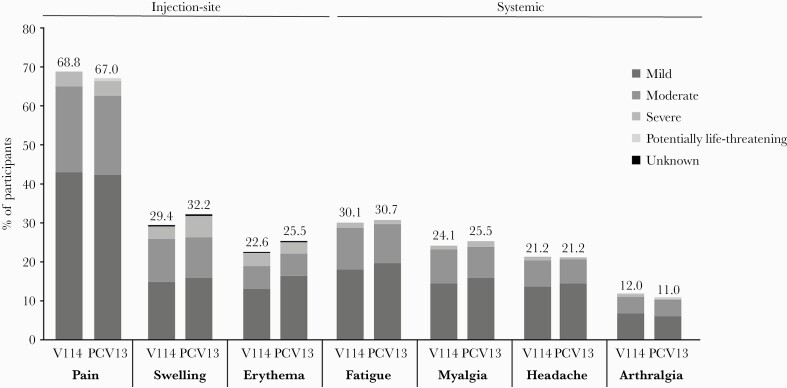
Proportion of participants with solicited AEs after vaccination with PPSV23, by severity. Solicited AEs collected postvaccination with PPSV23 6 months after vaccination with 15-valent pneumococcal conjugate vaccine (V114; n = 1036) or 13-valent pneumococcal conjugate vaccine (PCV13; n = 345). One participant in the PCV13 group incorrectly received V114 and was included in the V114 group for safety analyses. Every subject is counted a single time for each applicable specific AE. Injection-site events were solicited from day 1 to day 5 following vaccination. Systemic events were solicited from day 1 to day 14 following vaccination. The height of the stacked bar represents the total percentage of participants reporting the AE. Per the US Food and Drug Administration guidance on toxicity grading in preventive vaccine clinical trials, the severity of AEs was categorized as mild (grade 1), moderate (grade 2), severe (grade 3), or potentially life-threatening (grade 4). For solicited injection-site erythema and injection-site swelling, mild events measured >0 to ≤5 cm, moderate events measured >5 to ≤10 cm, and severe events measured >10 cm. For injection-site pain and systemic AEs, the severity was defined by the degree to which the event affected daily activities and the use of medications for pain relief. A severity of “potentially life-threatening” corresponds to a grade 4 AE [31]. The severity grades (mild, moderate, severe, or potentially life-threatening) within the bar indicate the proportion of the total attributed to each respective category. Abbreviations: AE, adverse event; PCV13, 13-valent pneumococcal conjugate vaccine; PPSV23, 23-valent pneumococcal polysaccharide vaccine; V114, 15-valent pneumococcal conjugate vaccine.

The proportions of participants with solicited AEs following vaccination with PPSV23 were generally comparable across vaccination groups (Figure 3; Table 3). Similar to what was observed following vaccination with PCV, the most frequently reported solicited injection-site AE and solicited systemic AE in both groups were injection-site pain and fatigue, respectively. Most solicited AEs were transient in both vaccination groups (≤3 days; Supplementary Table 4) and mild in severity (Figure 3).

### Immunogenicity

#### Following PCV Vaccination

**Figure 4.**
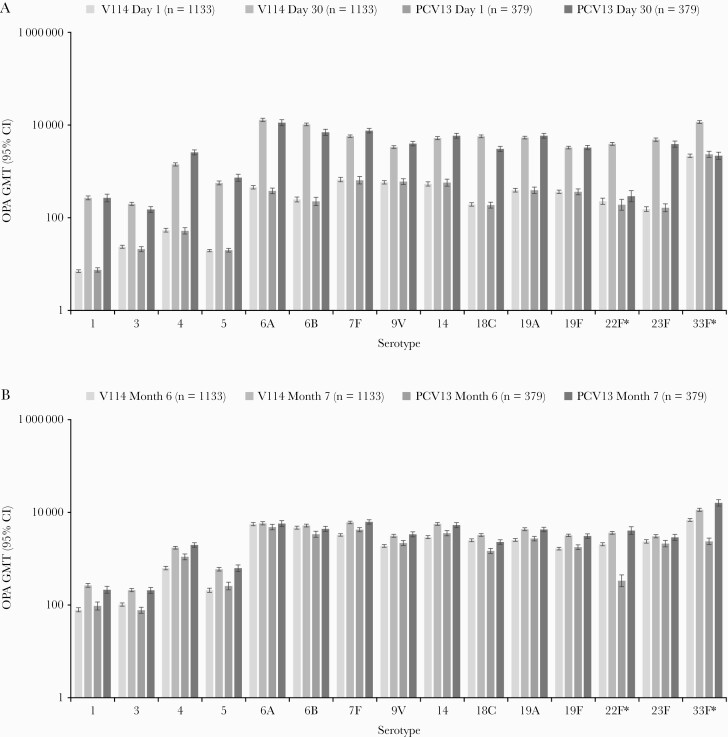
Serotype-specific OPA GMTs from baseline (Day 1) to 30 days postvaccination with pneumococcal conjugate vaccine (Day 30) (*A*) and from baseline (Month 6) to 30 days postvaccination with 23-valent pneumococcal polysaccharide vaccine (PPSV23; Month 7) (*B*). *Serotypes unique to V114. Abbreviations: CI, confidence interval; GMT, geometric mean titer (1/dil); OPA, opsonophagocytic activity; PCV13, 13-valent pneumococcal conjugate vaccine; V114, 15-valent pneumococcal conjugate vaccine.

**Figure 5.**
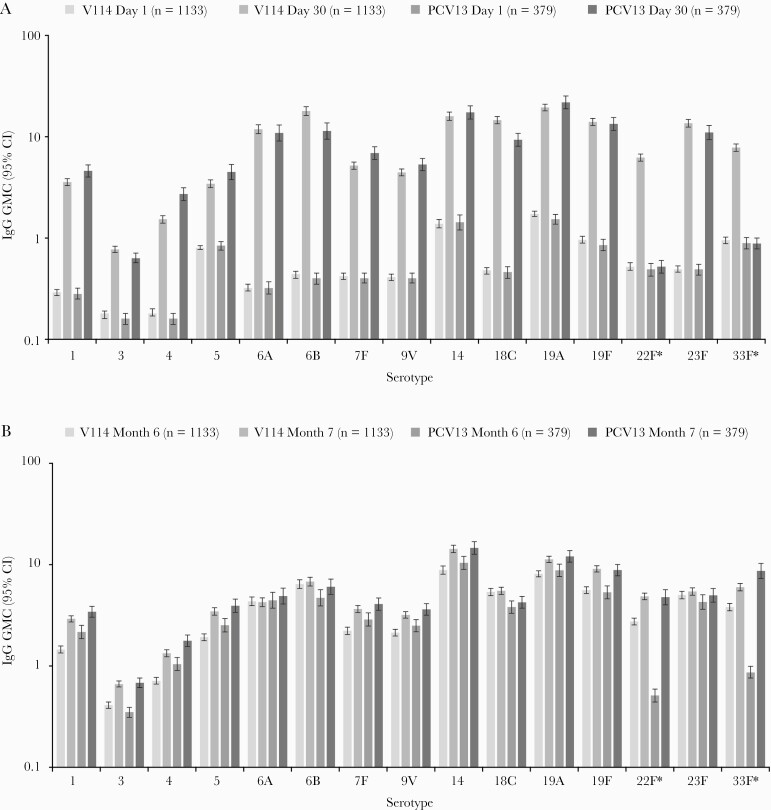
Serotype-specific IgG GMCs from baseline (Day 1) to 30 days postvaccination with pneumococcal conjugate vaccine (Day 30) (*A*) and from baseline (Month 6) to 30 days postvaccination with 23-valent pneumococcal polysaccharide vaccine (PPSV23; Month 7) (*B*). *Serotypes unique to 15-valent pneumococcal conjugate vaccine. Abbreviations: CI, confidence interval; GMC, geometric mean concentration (µg/mL); IgG, immunoglobulin G; PCV13, 13-valent pneumococcal conjugate vaccine; V114, 15-valent pneumococcal conjugate vaccine.

At 30 days postvaccination, both V114 and PCV13 were immunogenic in pneumococcal vaccine-naive immunocompetent adults aged 18–49 years with or without risk factors for PD, as assessed by OPA GMTs for all serotypes contained in the respective vaccines (Figure 4A). Serotype-specific OPA GMTs and IgG GMCs were generally comparable across vaccination groups for the 13 shared serotypes, and higher in the V114 group than in the PCV13 group for serotypes 22F and 33F (Figures 4A and 5A). Of note, baseline OPA titers for serotype 33F were higher than baseline titers for other serotypes in both vaccination groups. This assay-related phenomenon has been observed in other studies of the V114 development program. Baseline OPA titers for serotype 33F were comparable in the V114 and PCV13 groups and did not increase in the PCV13 group after the first vaccination with PCV13, which does not contain serotype 33F. Serotype-specific IgG GMCs at Day 30 also showed that V114 was immunogenic for all 15 vaccine serotypes and that PCV13 was immunogenic for its 13 vaccine serotypes. Results observed for OPA GMTs and IgG GMCs were consistent with results observed for OPA and IgG serotype-specific GMFRs and proportions of participants with a ≥4-fold rise from Day 1 to Day 30. OPA GMTs and IgG GMCs decreased from Day 30 to Month 6 but remained above baseline for all the serotypes contained in the respective vaccine (Supplementary Tables 5 and 7). OPA GMT ratios and IgG GMC ratios at Day 30 are graphically displayed in Supplementary Figures 1*A* and 2*A*, respectively. OPA GMT ratios (V114/PCV13) for the 13 shared serotypes varied between serotypes, ranging from 0.75 for serotype 7F to 1.85 for serotype 18C. Conversely, IgG GMC ratios ranged from 0.52 for serotype 4 to 1.53 for serotype 18C at Day 30. As anticipated, OPA GMT ratios and IgG GMC ratios were higher for the 2 serotypes unique to V114, ranging from 5.55 for serotype 33F (OPA GMT ratio) to 13.05 for serotype 22F (OPA GMT ratio). In both vaccination groups, there was a trend toward lower serotype-specific OPA GMTs in the older age group (40–49 years) compared with the younger age group (18–29 years; Supplementary Table 6). Reverse cumulative distribution curves (RCDCs) for serotype-specific OPA titers and IgG concentrations at Day 30 were consistent with the results of the above immunogenicity analyses (Supplementary Figures 3 and 4).

#### Following PPSV23 Vaccination

The administration of PPSV23 6 months after V114 or PCV13 was immunogenic, and both OPA GMTs and IgG GMCs were generally comparable across vaccination groups for all 15 serotypes (including 22F and 33F), as assessed by serotype-specific OPA GMTs and IgG GMCs at Month 7 and the GMFR and proportion of participants with ≥4-fold rise between Month 6 and Month 7 (Figures 4B and 5B; Supplementary Tables 5 and 7; Supplementary Figures 1*B* and 2*B*). RCDCs for serotype-specific OPA titers and IgG concentrations at Month 7 were consistent with results of the above immunogenicity analyses (Supplementary Figures 3 and 4).

## DISCUSSION

This study demonstrates the immunogenicity, safety, and tolerability of V114 in pneumococcal vaccine-naive immunocompetent adults aged 18–49 years with or without risk factors for PD, further contributing to the overall safety and immunogenicity profile of V114 to support its use in populations who would benefit most from the prevention of PD. Adults with ≥1 risk factor for PD are at increased risk of pneumococcal infection compared with healthy age-matched adults, with the risk of PD increasing as the number of risk factors increases [8–12]. In addition, at-risk adults are more likely to be hospitalized or die from PD than otherwise healthy age-matched adults [5, 7, 9, 37, 38]. In this study, V114 was immunogenic across all age groups (18–29, 30–39, and 40–49 years), with a trend toward lower immune responses in older participants, who also had a higher frequency of medical or behavioral risk conditions that could affect the response to vaccination.

V114 induced robust immune responses at Day 30 against its unique serotypes, 22F and 33F, which are epidemiologically important as common serotypes causing IPD [25–27]. Immune responses to the 13 serotypes shared by PCV13 and V114 were generally comparable between the 2 vaccination groups. As such, V114 has the potential to provide protection against these 2 additional serotypes while maintaining immune responses to serotypes shared with PCV13. Sequential administration of PPSV23 6 months after V114 was also well tolerated and immunogenic for all 15 serotypes. In participants who received PCV13 on Day 1, PPSV23 elicited immune responses to serotypes 22F and 33F, while maintaining responses to the 13 serotypes contained in PCV13.

In general, OPA and IgG responses decreased between Day 30 and Month 6 but remained above prevaccination levels measured at Day 1. At Month 7, OPA and IgG responses increased relative to levels measured immediately prior to PPSV23 vaccination; however, post-PPSV23 OPA titers and IgG concentrations were also lower than those measured at Day 30 (post-PCV) for some shared serotypes. This is consistent with previous evaluations of immune responses following sequential PCV/PPSV23 administration in adults and is likely due to the lack of boosting effect in individuals with high levels of preexisting antibodies [21, 39]. Despite this apparent lack of boosting observed with PPSV23 for some of the serotypes shared by V114 and PCV13, sequential PCV/PPSV23 regimens for at-risk immunocompetent adults, as well as older adults, have been implemented in some countries [4, 40]. The primary aim of sequential vaccination of PCV and PPSV23 is to broaden serotype coverage, as PPSV23 contains 9 serotypes not included in V114 and 11 not included in PCV13. Recent studies have supported sequential PCV/PPSV23 regimens by demonstrating that PPSV23 induced robust immune responses to 6 non-PCV13 serotypes when given either 2 or 6 months after PCV13 [41] and 7 non-PCV13 serotypes when given 1 month after PCV13 [42]. Other studies have evaluated the influence of different vaccine sequences and time intervals between PCV and PPSV23 administration on immune responses to shared serotypes between the PCV and PPSV23. In a study comparing PCV/PPSV23 vs PPSV23/PCV vaccination sequences in pneumococcal vaccine-naive adults aged 60–64 years, PPSV23 administered 1 year after PCV13 elicited noninferior responses for all 12 common serotypes and serotype 6A and statistically significantly greater responses for 11 of the 12 common serotypes (except serotype 14), compared with responses following PCV13 administered 1 year after PPSV23 [21]. Sequential PCV13/PPSV23 administration with either a 1- or 4-year interval also demonstrated significantly greater or noninferior responses for most of the shared serotypes compared with responses from PCV13 or PPSV23 alone in this population [21, 39]. Although no head-to-head studies have compared the effect of various time intervals between PCV and PPSV23 administration on immune response, longer intervals leave individuals unprotected against disease caused by serotypes unique to PPSV23. Therefore, the potential benefit of a shorter time interval (<1 year) should be considered.

The absence of accepted serotype-specific immunologic correlates of protection against PD, the heterogeneity of underlying medical conditions, the 3:1 randomization scheme of the study, and the lack of prespecified hypothesis testing limit the interpretability of between-group comparisons. However, comparisons made between vaccination groups in this study regarding immunogenicity refer to observed trends of OPA and IgG responses and the totality of the observed immunogenicity results (OPA GMTs, IgG GMCs, ≥4-fold rise in responses, and RCDCs), which do not suggest clinically meaningful differences. Regarding safety, absolute differences in the proportions of participants with specific AEs were small and the majority of AEs were mild to moderate in intensity and of short duration, following either conjugate vaccine when administered alone and in sequence with PPSV23.

Serotypes contained in PPSV23 but not in V114 were not evaluated at Month 7; however, in recent studies, PPSV23 induced robust immune responses to 6 non-PCV13 serotypes when given either 2 or 6 months after PCV13 [41] and 7 non-PCV13 serotypes when given 1 month after PCV13 [42].

In this study, approximately 20% of participants had ≥2 risk factors for PD. The disease burden, morbidity, and mortality from PD are higher in this population than in individuals with 0 or 1 risk factors and are close to those seen in patients who are immunocompromised [12], underscoring the need for PD prevention. A detailed analysis of safety and immunogenicity by the number and type of risk factors has recently been presented [43] and will be part of a future manuscript.

## CONCLUSIONS

In pneumococcal vaccine-naive immunocompetent adults 18–49 years of age with or without medical or behavioral risk factors for PD, V114 is well tolerated and immunogenic for all 15 serotypes contained in the vaccine, including 2 serotypes not contained in PCV13. Sequential administration of PPSV23 6 months after V114 is also well tolerated and maintains immune responses for shared serotypes. Therefore, V114 has the potential to broaden serotype coverage and protect against PD caused by serotypes not contained in other currently licensed PCVs.

## Supplementary Material

ofab605_suppl_Supplementary_Materials

ofab605_suppl_Supplementary_Protocol

## Notes

***Author contributions.*** L. L. H.: conception, design, or planning of the study; acquisition of the data; interpretation of the results; drafting of the manuscript; and reviewing or revising the manuscript for important intellectual content. F. J. P. and K. C.: conception, design, or planning of the study; acquisition of the data; interpretation of the results; and reviewing or revising the manuscript for important intellectual content. D. Q., E. J., K. R. R., and K. A.: acquisition of the data; interpretation of the results; and reviewing or revising the manuscript for important intellectual content. R. T.: interpretation of the results and drafting of the manuscript. I. M. K.: acquisition of the data and reviewing or revising the manuscript for important intellectual content. R. D.: analysis of the data; interpretation of the results; drafting of the manuscript; and reviewing or revising the manuscript for important intellectual content. J. M.: conception, design, or planning of the study; drafting of the manuscript; and reviewing or revising the manuscript for important intellectual content. A. P.: conception, design, or planning of the study; analysis of the data; interpretation of the results; and reviewing or revising the manuscript for important intellectual content. T. S. and G. T.: conception, design, or planning of the study and reviewing or revising the manuscript for important intellectual content. L. M.: conception, design, or planning of the study; analysis of the data; interpretation of the results; drafting of the manuscript; and reviewing or revising the manuscript for important intellectual content. U. K. B.: conception, design, or planning of the study; analysis of the data, acquisition of the data; interpretation of the results; drafting of the manuscript; and reviewing or revising the manuscript for important intellectual content.

***Acknowledgments.*** We would like to thank each of the participants, study staff, and investigators in the V114-017 (PNEU-DAY) study group (see Supplementary Table 1) for their invaluable contributions to this study. Medical writing support, including assisting authors with the development of the outline and initial draft and incorporation of comments, was provided by Cindy Cheung, MBBS (MD), and editorial support, including figure preparation, formatting, proofreading, and submission, was provided by Ian Norton, PhD, both of Scion, London, UK, supported by Merck and Co, Inc, according to Good Publication Practice guidelines.

***Patient consent.*** Written informed consent was obtained from each participant prior to any study procedure. The design of the study was approved by the appropriate local institutional review boards and regulatory agencies.

***Data sharing.*** The data sharing policy of Merck Sharp & Dohme Corp (a subsidiary of Merck & Co, Inc, Kenilworth, New Jersey), including restrictions, is available at http://engagezone.msd.com/ds_documentation.php through the EngageZone site or via email to dataaccess@merck.com.

***Disclaimer.*** The sponsor was involved in the study design, collection, analysis, and interpretation of data, as well as data checking of information provided in the manuscript. However, ultimate responsibility for opinions, conclusions, and data interpretation lies with the authors.

***Financial support.*** This study was supported by Merck Sharp & Dohme Corp, a subsidiary of Merck & Co, Inc, Kenilworth, New Jersey.

***Potential conflicts of interest.*** L. L. H. has received research grants paid to her institution from Merck and Pfizer. E. J. has received honoraria from AbbVie, Bristol-Myers Squibb, Roche, and Merck. F. J. P. has received consulting fees from AstraZeneca, Lilly, and Merck; speaker fees from Merck and Boehringer Ingelheim; and research grants paid to his institution from Merck, Dexcom, and Insulet. K. R. R. has received consulting fees from Merck, served on an advisory board for Novartis–data and safety monitoring board, and received research support paid to his institution from Merck. R. D. has received grants and personal fees from Pfizer and Merck and grants from MedImmune. A. P., G. T., J. M., K. C., L. M., T. S., and U. K. B. are employees of Merck Sharp & Dohme Corp, and may own stock and/or stock options in Merck & Co, Inc. All other authors report no potential conflicts of interest.

The full list of study investigators is shown in Supplementary Table 1.
