# Supplementary material for: Immunogenicity, Safety, and Tolerability of V114, a 15-Valent Pneumococcal Conjugate Vaccine, in Immunocompetent Adults Aged 18–49 Years With or Without Risk Factors for Pneumococcal Disease: A Randomized Phase 3 Trial (PNEU-DAY)
Source: Open Forum Infect Dis. 2021 Dec 18;9(3):ofab605. doi: 10.1093/ofid/ofab605 (PMC8826015; doi:10.1093/ofid/ofab605)
Supplement: ofab605_suppl_Supplementary_Materials [file ofab605_suppl_supplementary_materials.docx]

# Supplementary materials

Supplementary Figure 1. Estimated OPA GMT ratios at Day 30 (a) and Month 7(b).


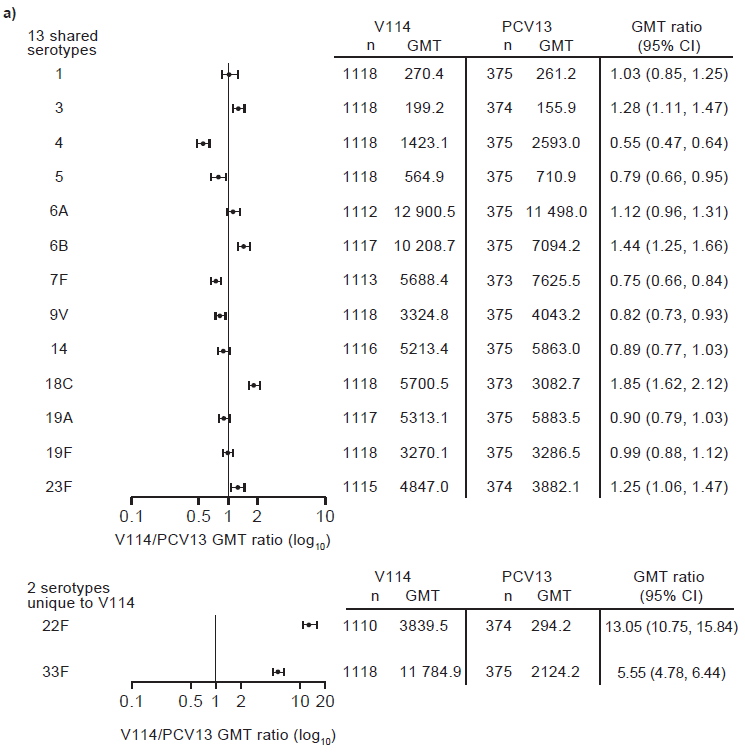


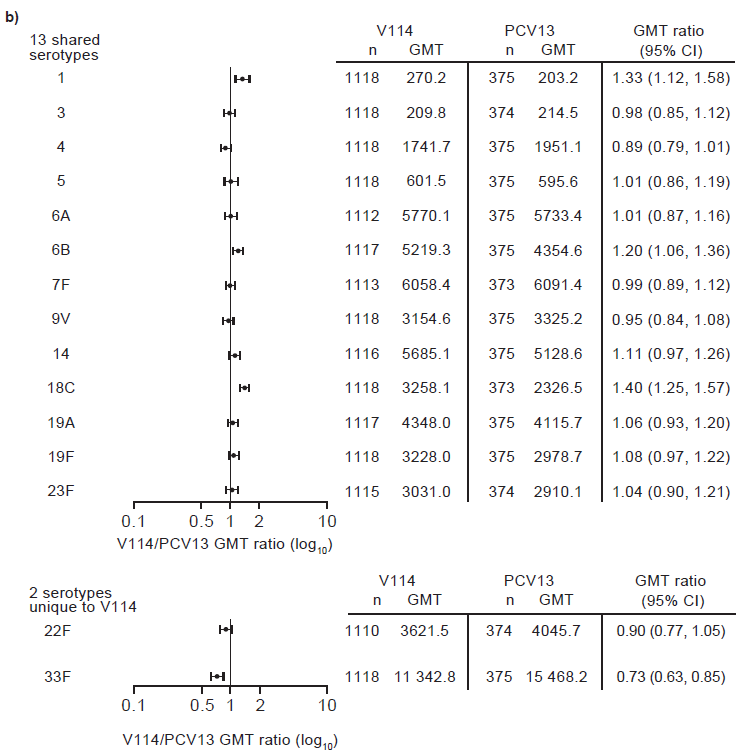


Day 30 is 30 days following vaccination with V114 or PCV13, and Month 7 is 30 days following vaccination with PPSV23.
Abbreviations: CI, confidence interval; GMT, geometric mean titer (1/dil); OPA, opsonophagocytic activity; PCV13, 13-valent pneumococcal conjugate vaccine; PPSV23, 23-valent pneumococcal polysaccharide vaccine; V114, 15-valent pneumococcal conjugate vaccine.

Supplementary Figure 2. Estimated IgG GMC ratios at Day 30 (a) and Month 7 (b).


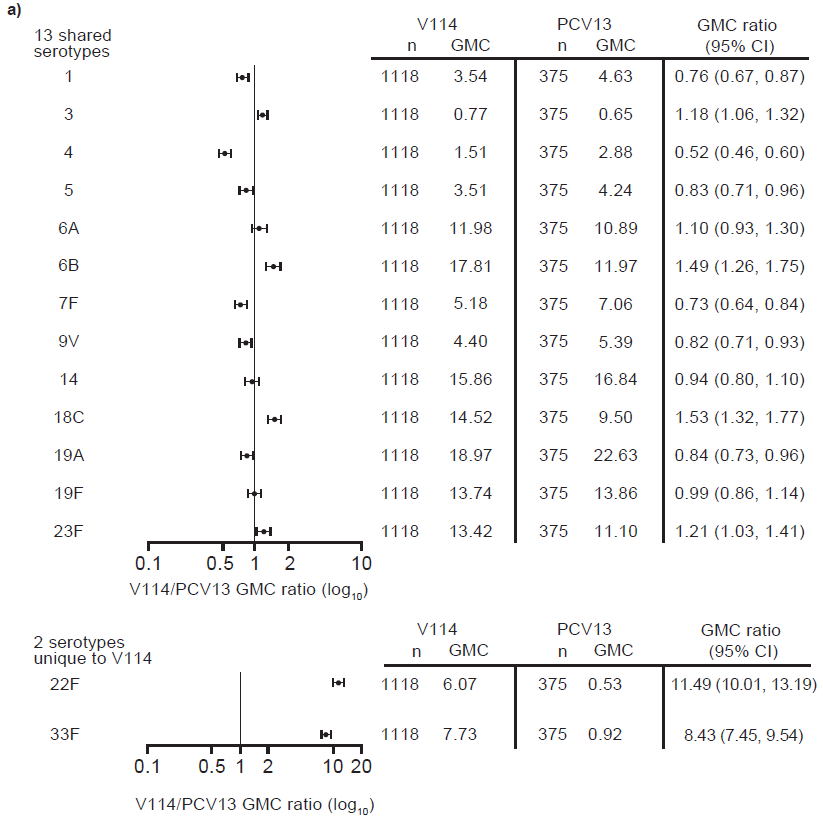


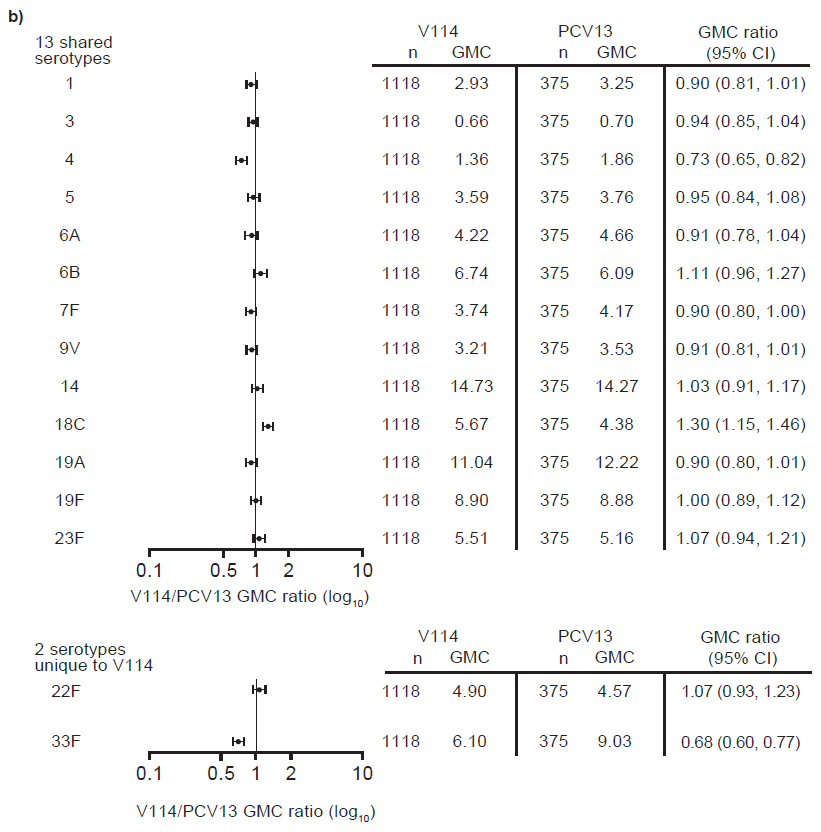


Day 30 is 30 days following vaccination with V114 or PCV13, and Month 7 is 30 days following vaccination with PPSV23.
Abbreviations: CI, confidence interval; GMC, geometric mean concentration (µg/mL); IgG, immunoglobulin G; PCV13, 13-valent pneumococcal conjugate vaccine; PPSV23, 23-valent pneumococcal polysaccharide vaccine; V114, 15-valent pneumococcal conjugate vaccine.


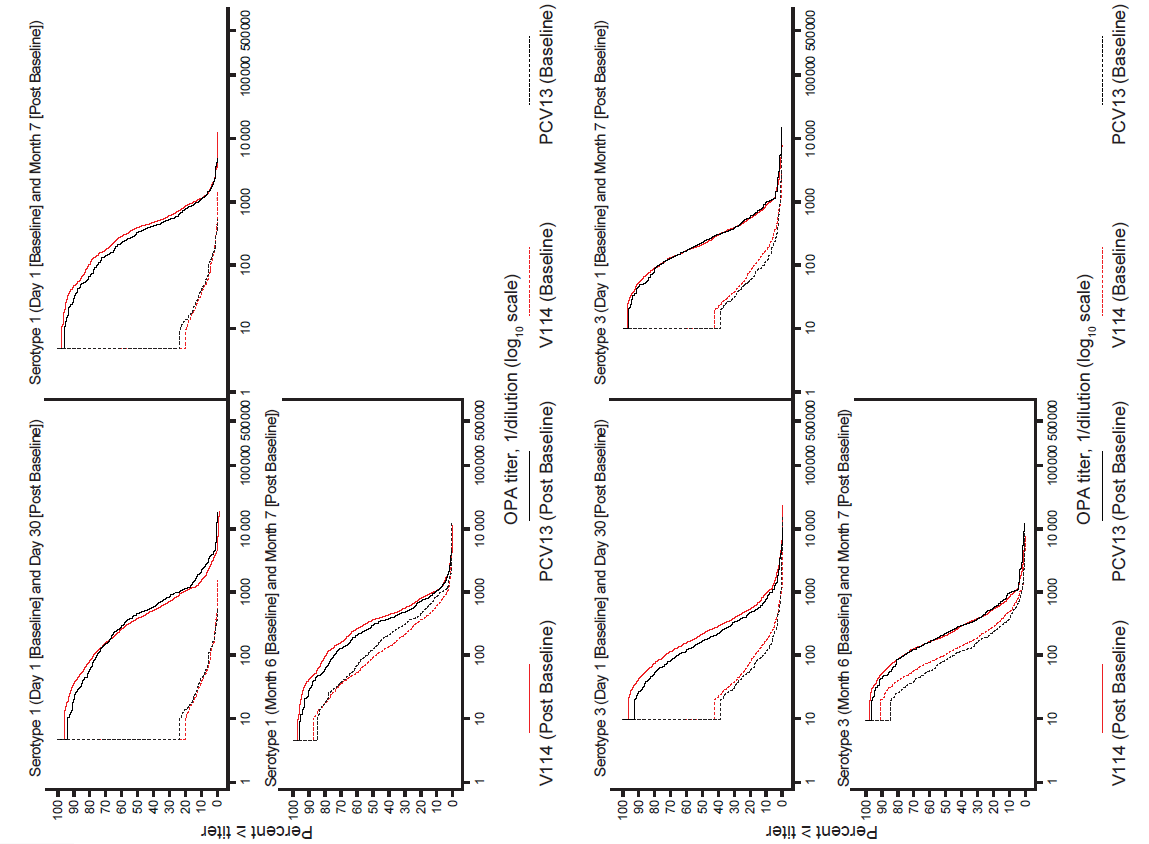
Supplementary Figure 3. Reverse cumulative distribution curves of OPA titers.


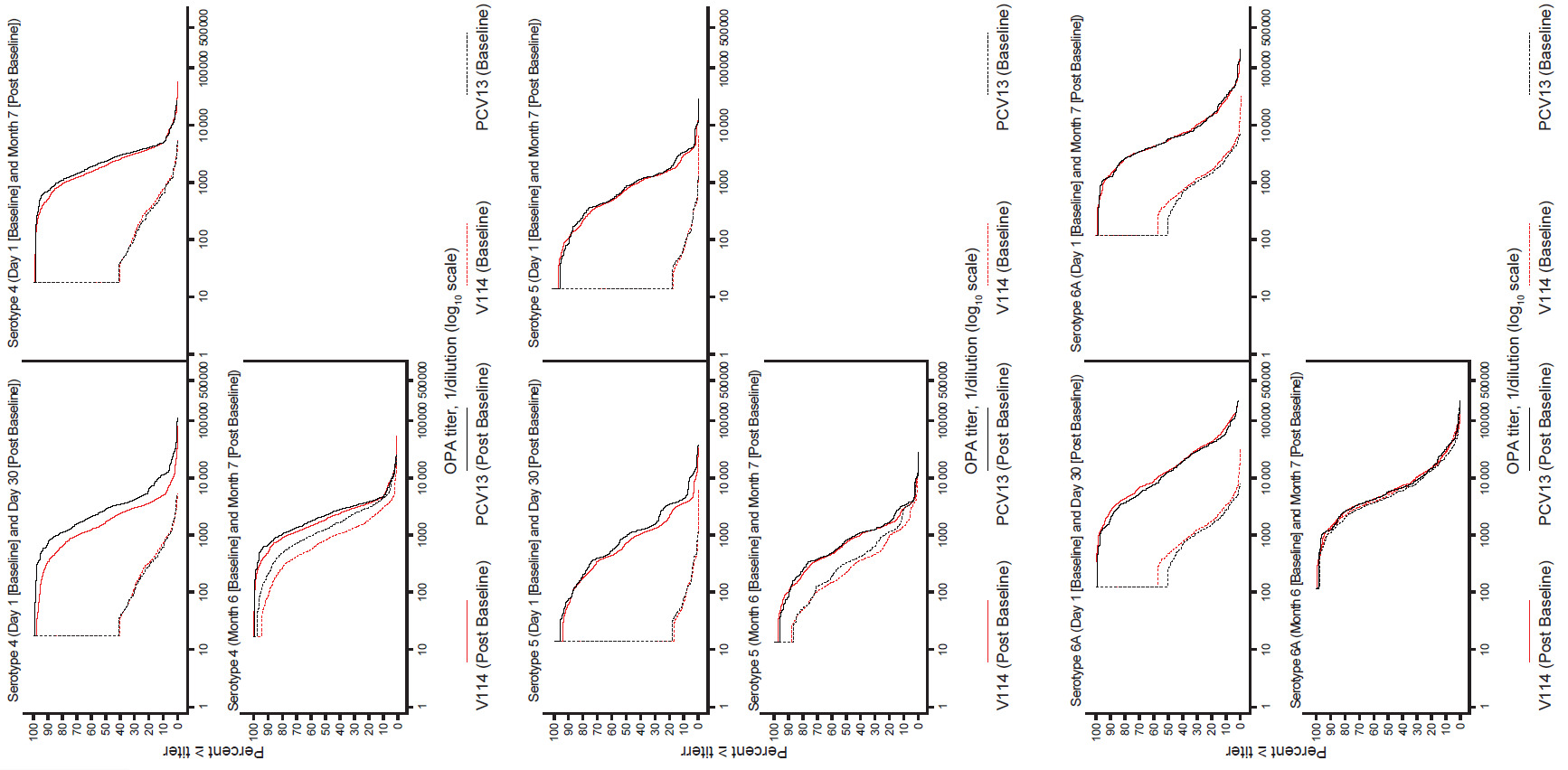

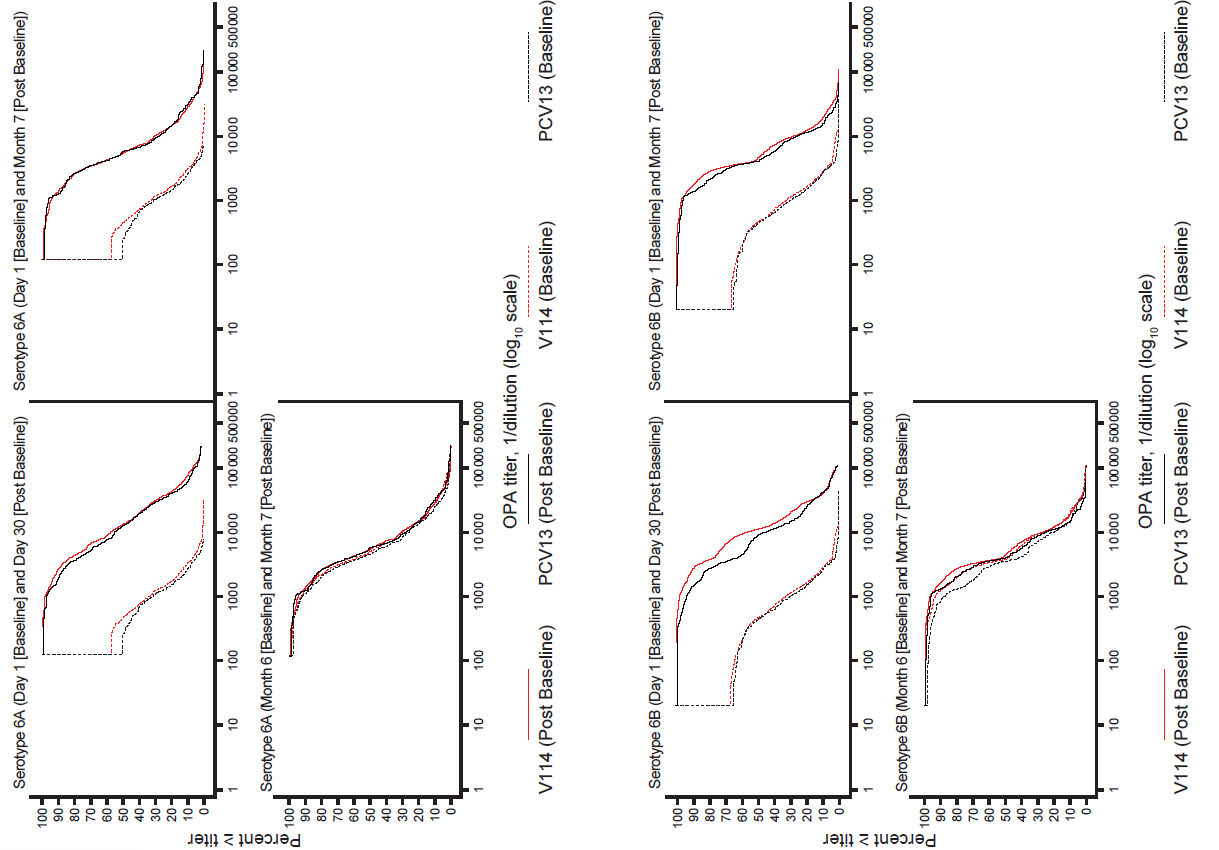

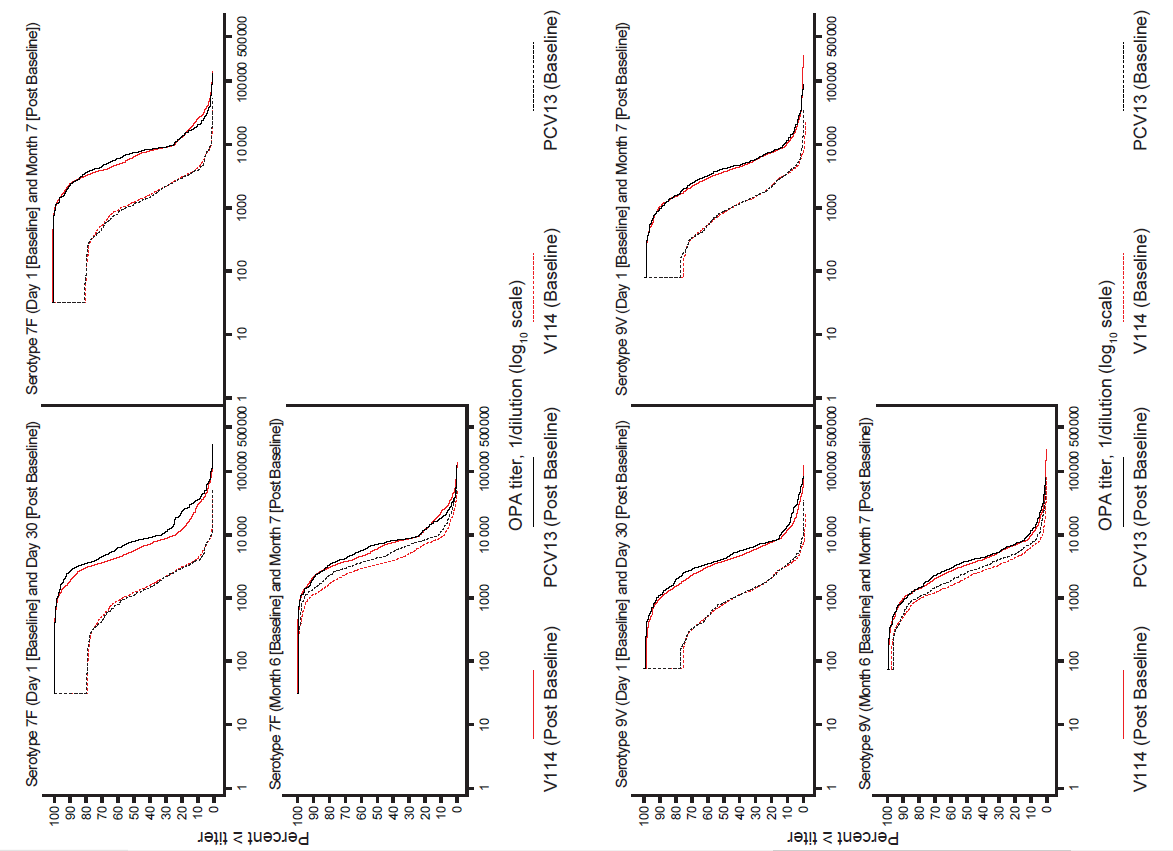

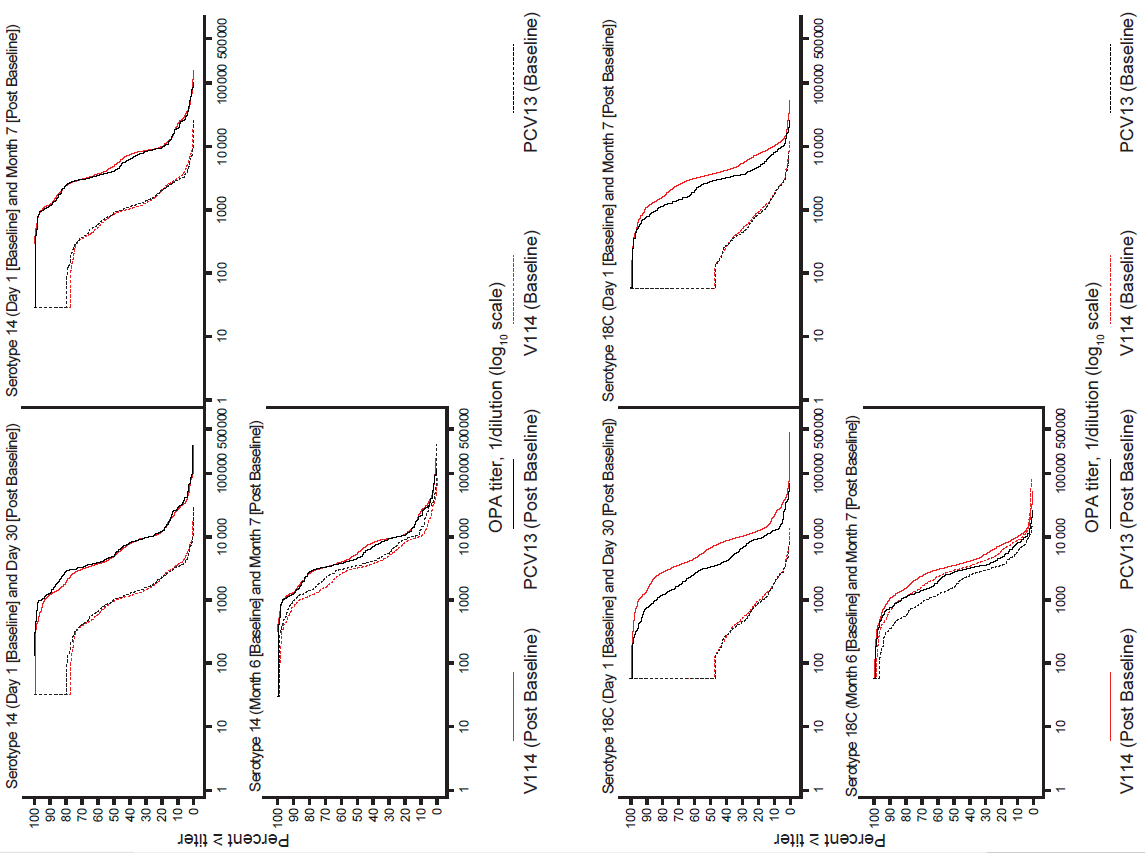

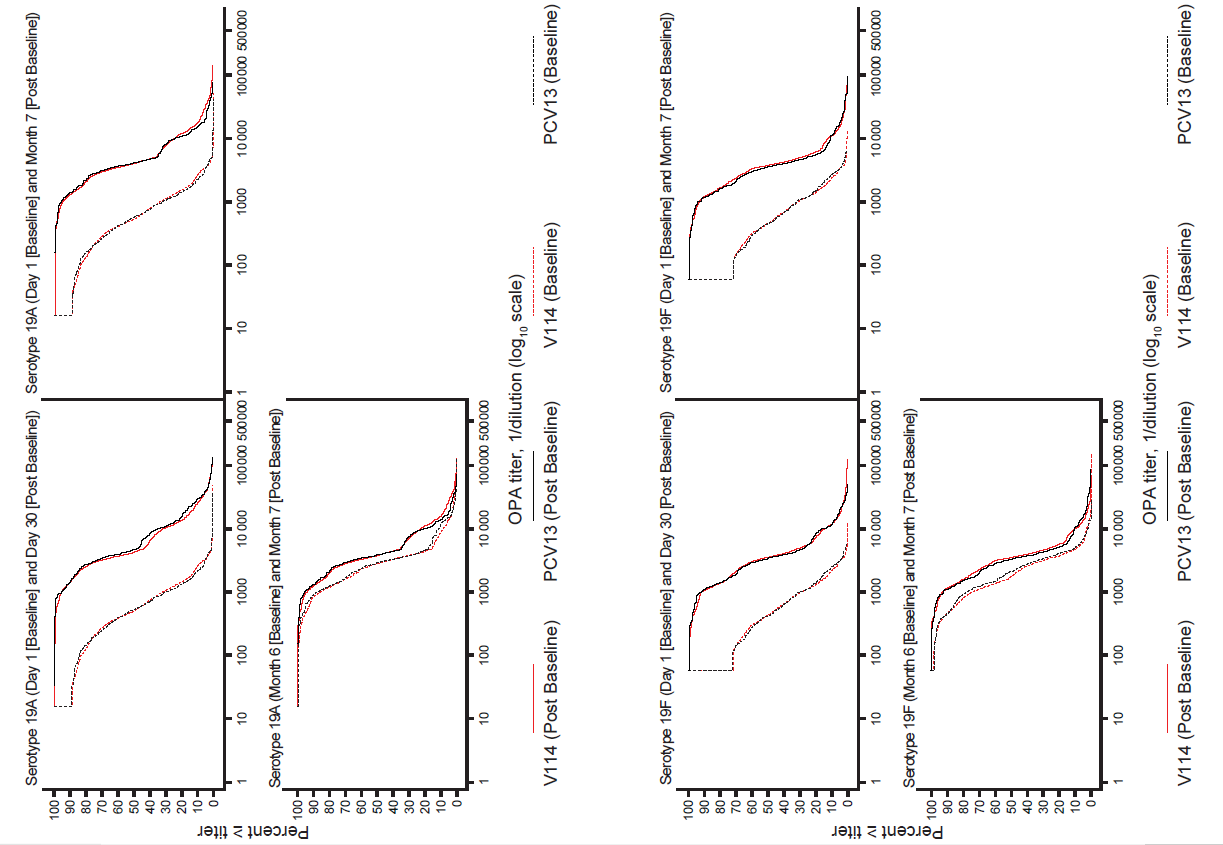

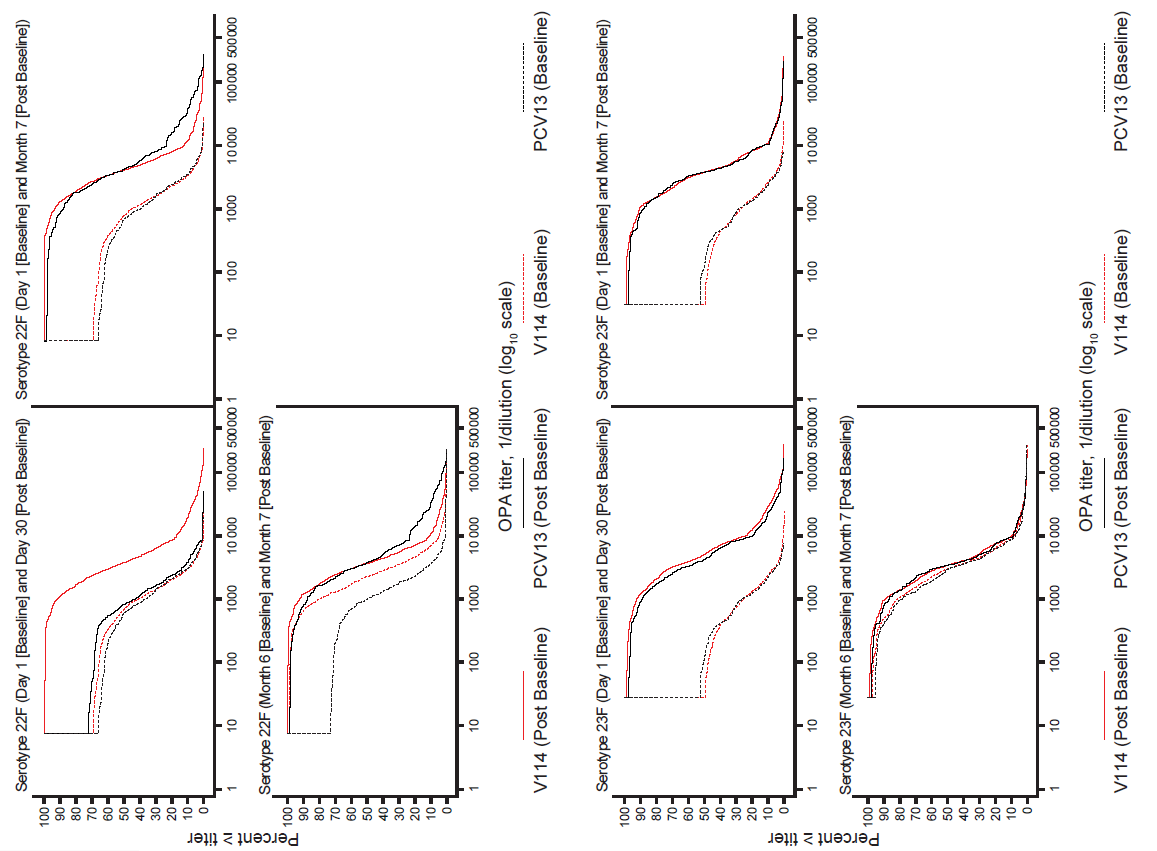

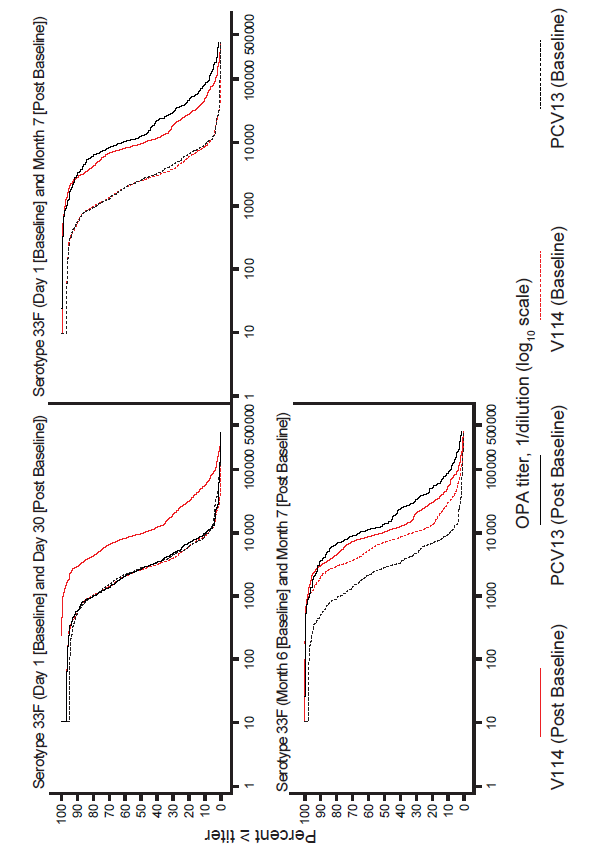


Abbreviations: OPA, opsonophagocytic activity; PCV13, 13-valent pneumococcal conjugate vaccine; V114, 15-valent pneumococcal conjugate vaccine.


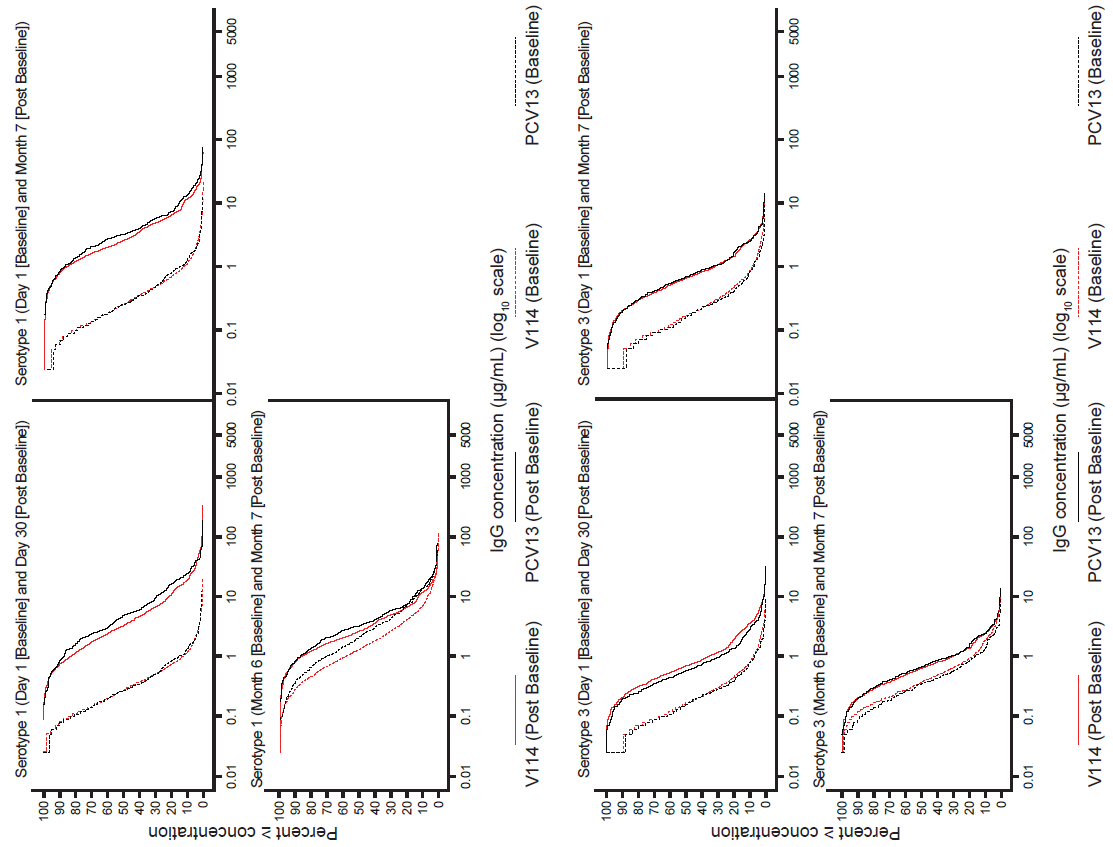
Supplementary Figure 4. Reverse cumulative distribution curves of IgG concentrations.


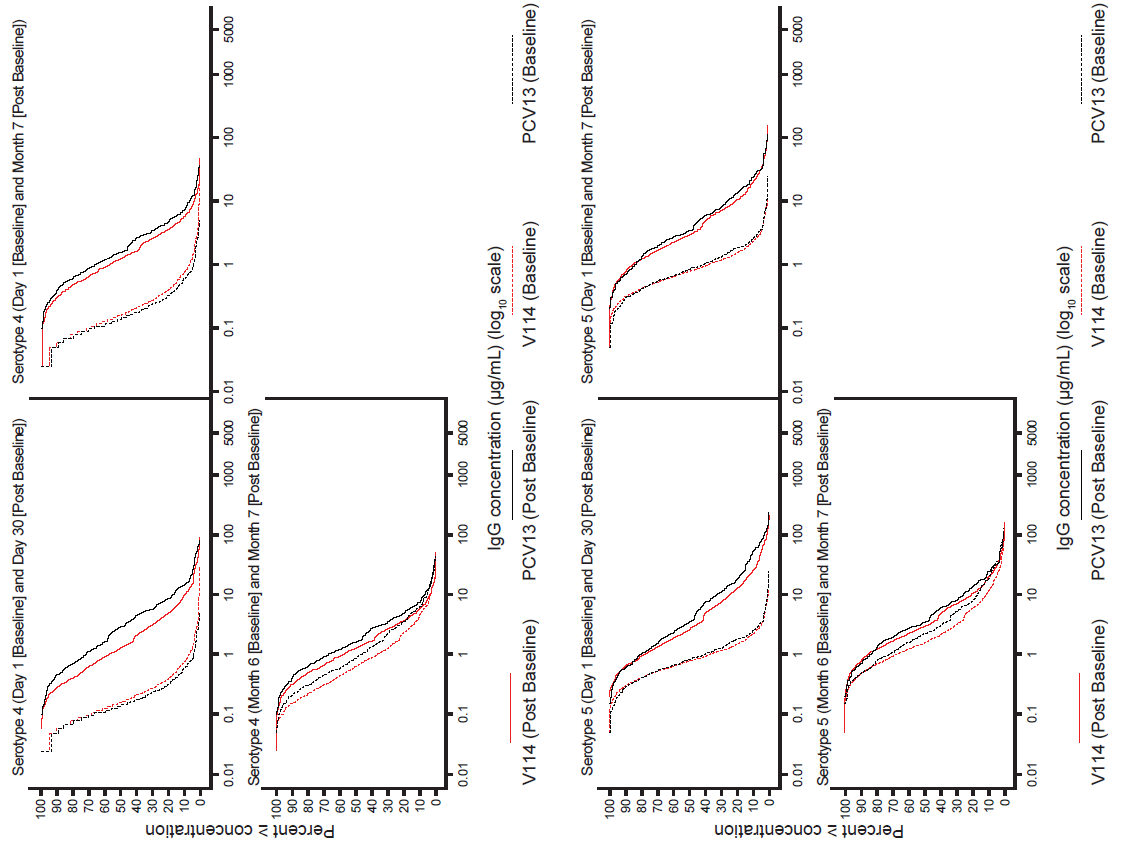


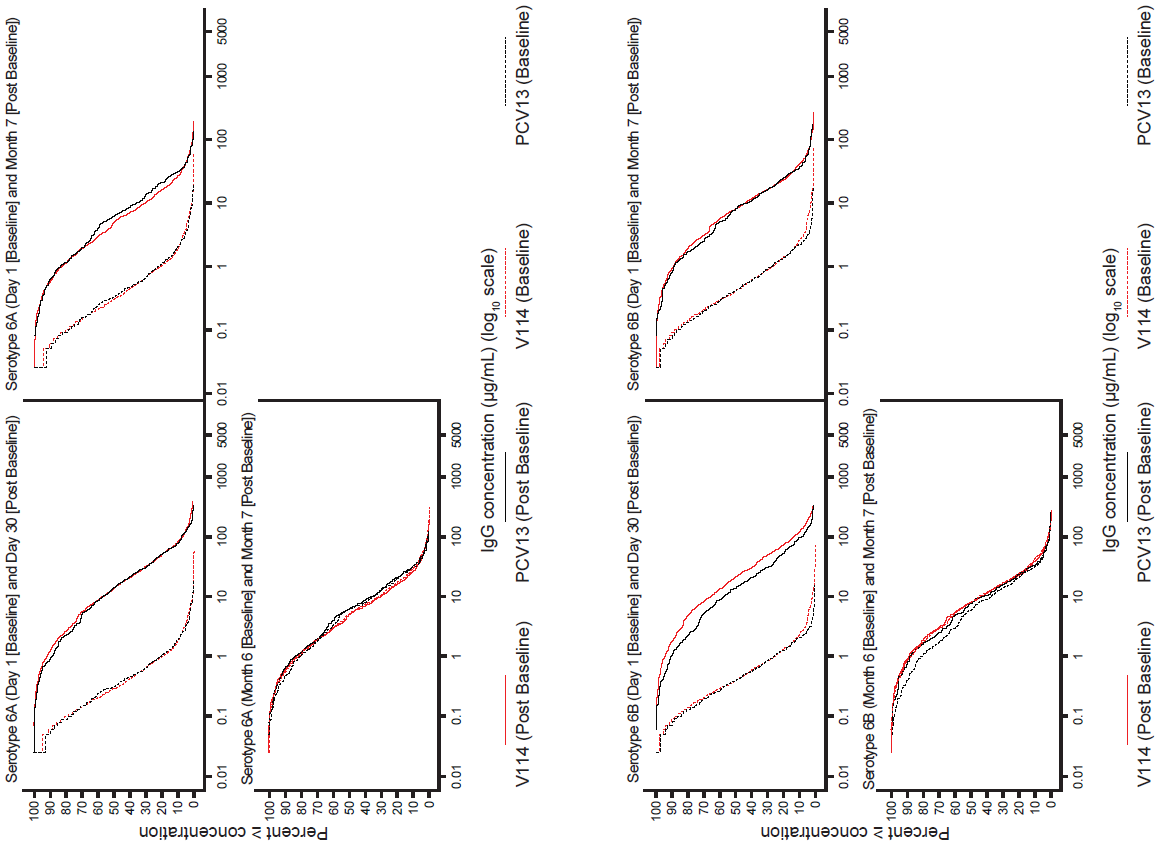


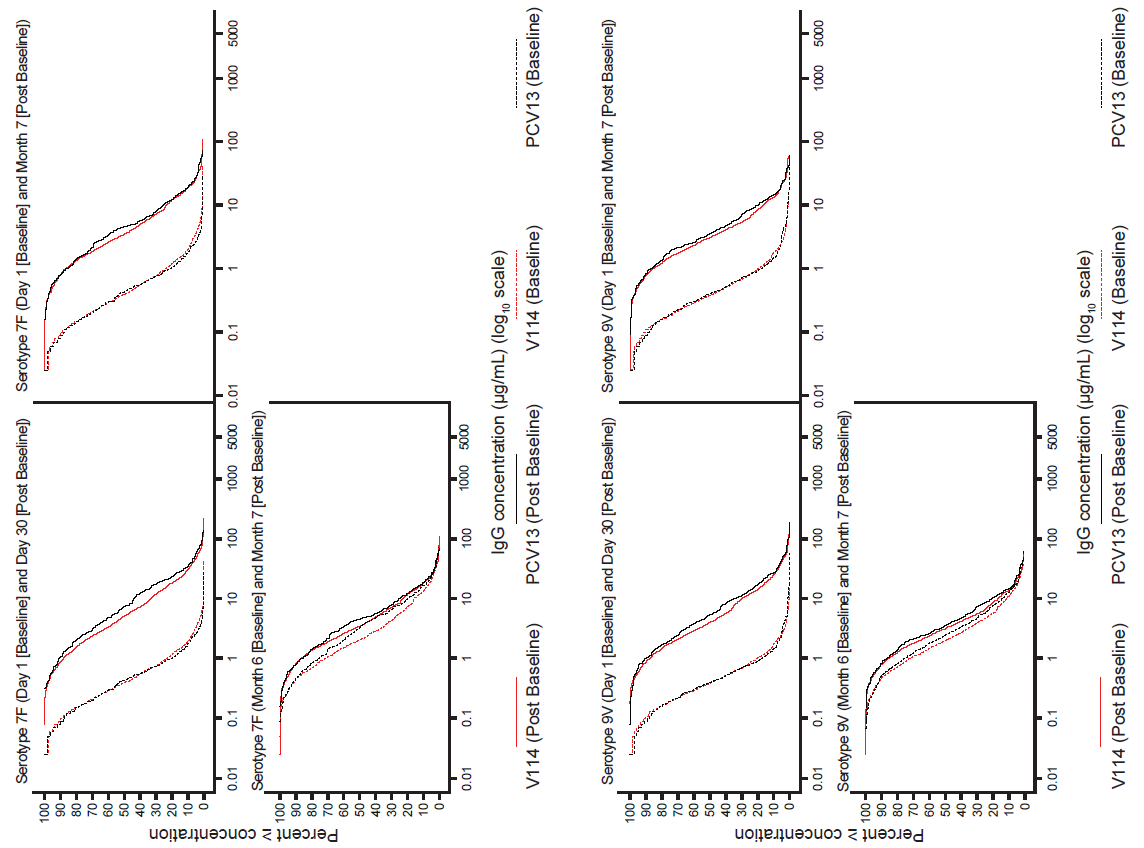


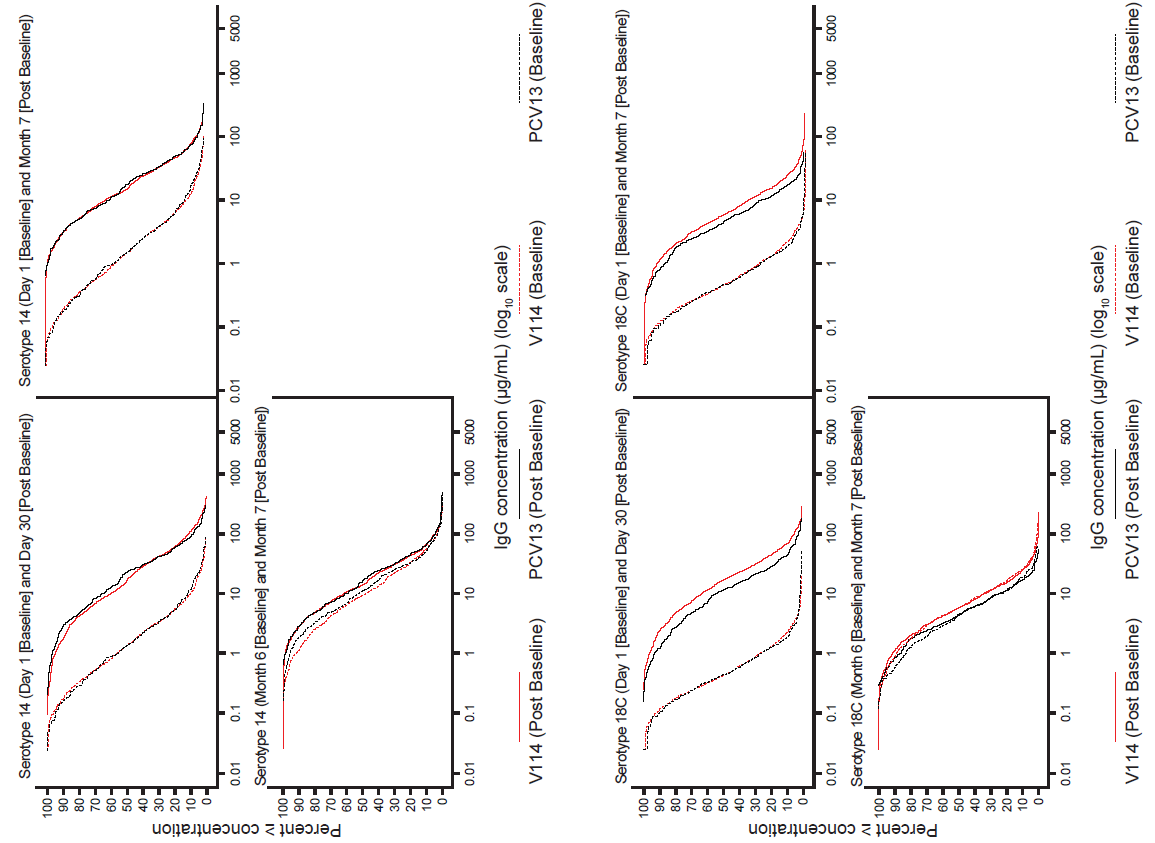


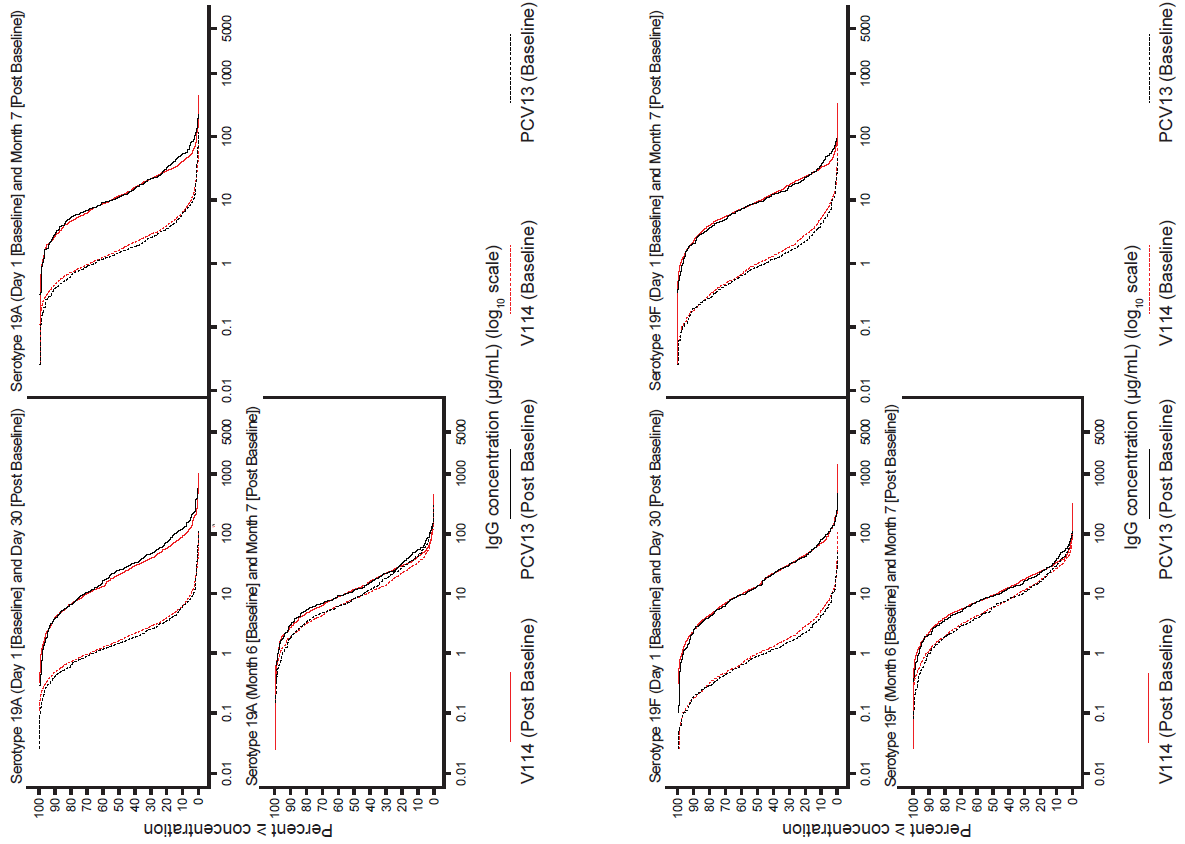


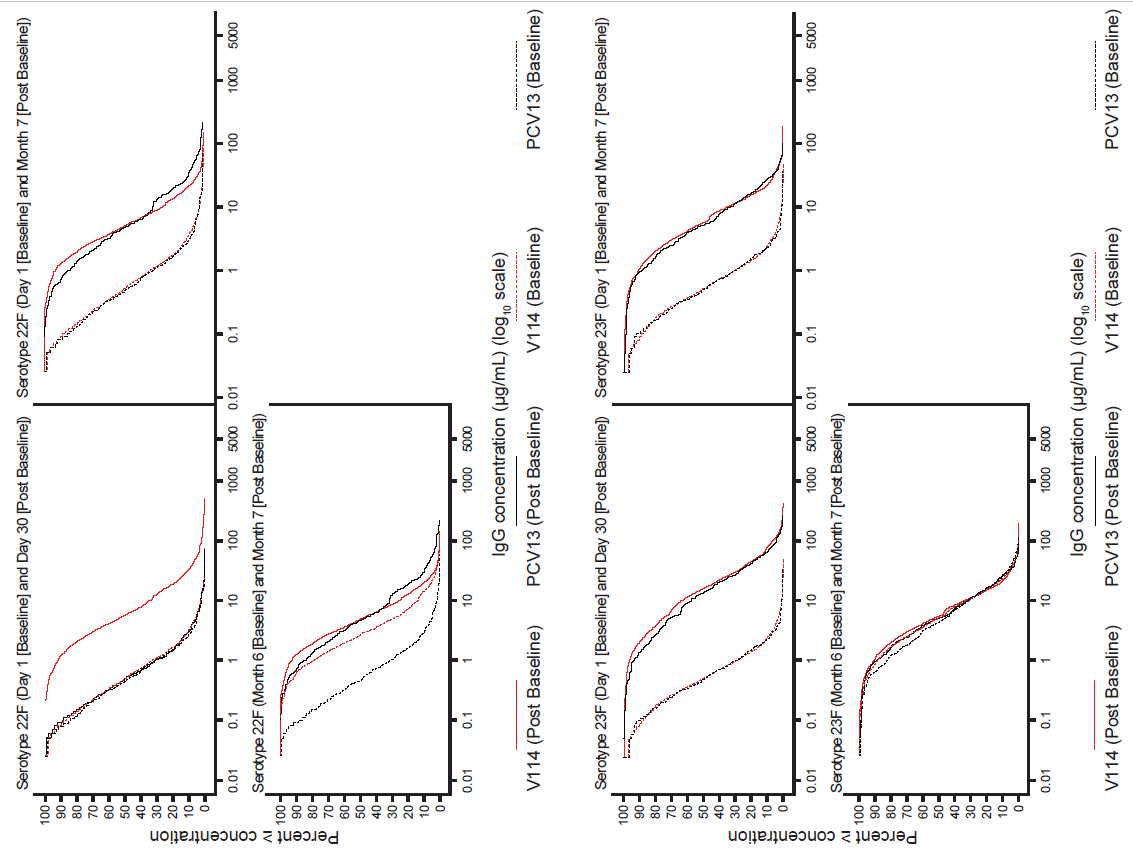


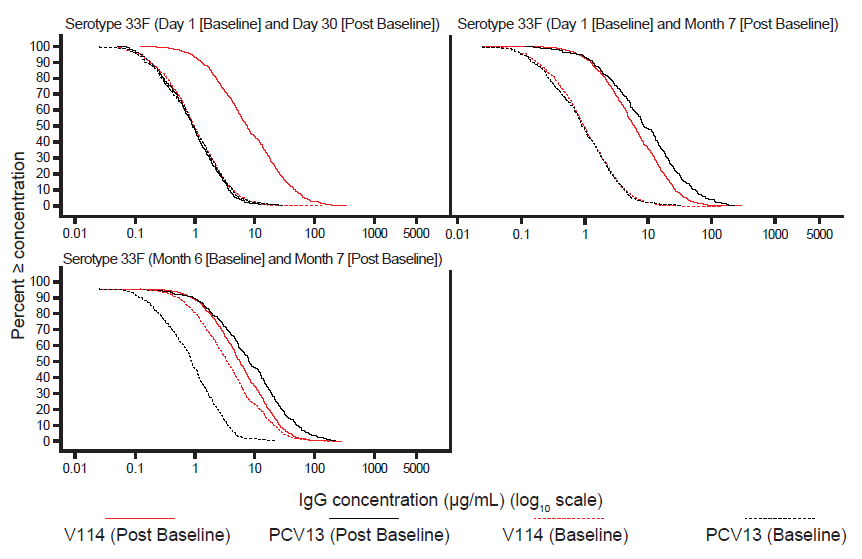


Abbreviations: IgG, immunoglobulin G; PCV13, 13-valent pneumococcal conjugated vaccine; V114, 15-valent pneumococcal conjugate vaccine.

Supplementary Table 1. List of Investigators from V114-017 (PNEU-DAY).

| **Location** | **Primary investigator** | **Sub-investigators** |
| --- | --- | --- |
| United States | Laura Hammitt | Laura Brown, Estar Denny, Megan Gardner, Lindsay Grant, Jane Halpern, Kirstin Howell, Tally Jones, Katherine O’Brien, Alicia Portillo, Raymond Reid, Nina Ritchie, Kristen Roessler, Mathuram Santosham, Carol Tso, Dan VanDeRiet, Robert Weatherholtz |
| United States | Madhavi Ampajwala | Sander J. Gothard, Lynn Gross, Shelley Loomstein, Lindsay Osborn, Janelle Plourde |
| United States | William David Byars | Thomas Victor Ballard, Cathleen Castay Jeffers, Ronald Keith Mayfield |
| United States | Sidney E Clevinger | Karen Canganelli, Lynn M. Craggs, Jay Klein, Julady Mederos, John P. Nardandrea, Mandy Roomy, Joseph Sorrentino, Erin Zimmer |
| United States | Jeffrey G. Geohas | Yijia Geohas, Ewa Mioduszewska, Anil Samson, Nicole Yamagiwa |
| United States | Matthew Charles Hall | Ray C. Haselby |
| United States | Ekram Hanna | Shannon Broschard, Fiore J. Copare, John Hatzantonis, Jeanette Jimenez-Silva |
| United States | Wayne Harper | Lisa M. Cohen, Miroslav, Gavazov, Douglas Wadeson |
| United States | Kyla R Lee | John Conzemius |
| United States | Sashi K. Makam | William Chrvala, Traci Dolbeer, Lindsey Hill, Jennifer Nazaroff, Dana Stivella, Kimberly Szwartz |
| United States | David J. Morin | Shane A. Daniell, Ashley Helton, Curtis Jantzi, Mindy Johnson, Natasha Kanipe, Emily J. Morawski, Emily Price, Lori L. Ray, Hannah Wheeler |
| United States | Olayemi O. Osiyemi | Christina Campbell, Jose Menajovsky-Chaves |
| United States | Francisco Pasquel | Georgia M Davis, Maya Fayfman, Guillermo Umpierrez |
| United States | K. Rajender Reddy | Lorie Sinese, Ethan Weinberg |
| United States | Ernie Riffer | Barbara Berry, Austin Blanchard, Meghan Caldron, Shelby  Chouinard, Annamarie Haught, Agustin Herber, Erika Hoffman, Ryan Imber, Yuka Koyama, Barbara Lipschitz, Stephanie Milroy, Gina Nguyen, Dorothy Trimmer, Kelly Vesely |
| United States | Jose Luis Ruiz | Anselmo Humberto Humaran, Roberto Morejon |
| United States | Richard Earl Rupp | Lemuel Aigbivbalu, Megan Berman, Cori M. Burkett, Laura Porterfield |
| United States | Christopher A. Smith | Mark Booth, Jennifer Clarke, Lisa Condefer-Smith, Amber M.  Conklin, Jennifer Cutton, John D. Gordon, Tracey Loid, Melinda Plumley, Margaret Radez, Kelly Stoquert |
| United States | Mary Delila Tipton | Curtis Kenneth Andrews, Megan Ellice Crookston, Kathy Stretch Garcia, Martin A. Hollingsworth, Thanh Nakata, Christine Pyne, Alexsis Silva, Vilate Marie Thur, Ami White |
| United States | Joseph Leo Yozviak | Cynthia E. Beitler, Lauri L. Centolanza, Traci Eichelberger, Marcelo Gareca, Margaret Hoffman-Terry, Terry Kloiber, Victoria Leib, Arun Mancheril, Hugh A. Marsh, Andrew Orzel |
| United States | Veronica Garcia Fragoso | Maria Gabriela Becerra, Teresa M. Becker, Stacey Eckert, Ashraf A. Jafri, Cherry Khaykin, Vicki E. Miller, Alexandra Schwob, Teodoro Seminario |
| United States | Rebecca J. Bertsch | Shamela Barnett, Jordan R. Duncan, Gretchen M. Foley, Emily  Kelley, Sherry Walentiny |
| United States | Mark H. Gotfried | Larissa Anthony, Stephen R. Anthony, Kellie Barbour, Jacquie  Chilberg, Vince DeLaCruz, Li Yi Fu, Lynda S. Heaphy, Shawn ONeal |
| United States | Edward F. Kent | Mark Lazarovich |
| United States | Allison Lambert | Sonja Arganbright, Benjamin S. Bartsch, Breanna Diehl, Jeffrey C. Elmer, Jessica Horton, Donald S. Howard, Ronnie Mantilla, Steven Richardson, Carolina See, Michelle A. Sherwood, Jacquelyn Soderberg |
| United States | Faisal Wahid | - |
| United States | John Elsen | Gabriella M Delgado, Kenneth T Kruchten |
| United States | John E. Ervin | Deanna Biggs, Barbara Bradshaw, Sandra Bradshaw, Erin  Flanagan, Jason Huntington, Sarah Jokerst, Kiesha Kelly-Goodson, Josh Miller, Christine D. Morgan, Yvette Witherell |
| United States | James Feldman | Jawad Ashour, Amir Kashani, George Mammen, Rajen Mehta, Anil Odhav, Albina Pace, Kelly Parkes, Madeline Peek, Jessica  Schneider, Selim Sekili, John Sunew |
| United States | Zeid Kayali | Amanda M. Siegel |
| United States | Michael B Jacobs | Kathleen Menasche, Vincent Mirkil |
| United States | Jose F. Cardona | Lincoln A Garay, Evelio H Sosa |
| United States | Zulfiqar Mirza /  John McGinty | Jithendra Choudary, Rubinder Dab |
| United States | Vijay Jayachandran | Sukesh Burjonroppa, Arun Padala |
| Canada | Andre Frechette | Guy Chouinard, Michel Jobin |
| Canada | Richard Tytus | Stephen Tytus |
| Canada | Guy Tellier | Gilbert Gagne |
| Canada | Murdo Ferguson | Harold Berghuis, Linda Marie Ferguson |
| Canada | Anthony Dowell | Ronald Dandurand |
| Canada | Jean-Sebastien Gauthier | Louise Frenette, Luc Larrivee, Paule Royer |
| Canada | Sam Henein | Norman Kalyniuk |
| Canada | Edward Tam | Kwok Yeong Yik |
| Canada | Daniel A. Landry | Ronald Leonard Bourgeois |
| Chile | Alejandro Afani | Carla Bastias, Rolando Campillay |
| Chile | Victor Saavedra Cecim | Cindy Jimenez, Valeria Palma, Carla C. Saavedra, Dayana Santamaria |
| Chile | Miguel O. Ryan | Jimena Reyes, Enrique Vera |
| Chile | Katie Abarca | Magdalena De Aguirre, Violeta Rivas |
| Chile | Manuel Munoz | Herman Schneider |
| Poland | Marta Wachowicz-Tobolik | Magdalena Maria Dudek, Lucyna Losinska |
| Poland | Grazyna Pulka | Joanna Pabian-Macina, Karolina Pelka, Jerzy Soja |
| Poland | Katarzyna Szymkowiak | Agnieszka Ciesiolkiewicz-Wojcik, Marek Dwojak, Ewa  Jazwinska-Tarnawska, Ewa Krecipro-Nizinska, Zofia Ruzga, Anna Sidorowicz-Bialynicka |
| Poland | Ewa Janczewska | Arkadiusz Pisula, Wioleta Warunek |
| Poland | Aleksandra Szymczak | Jacek Gasiorowski, Weronika Rymer, Bartosz Szetela |
| Poland | Beata Bobel-Olchowik | Dagmara Borzych-Duzalka, Marek Cesarz, Malgorzata Jagielska-Plata, Milena Kowalewska-Celejewska, Monika Kuligowska-Jakubowska, Jakub Lata, Ingrid Raubo-Biezunska, Katarzyna Szudejko |
| Poland | Pawel Miekus | Bartosz Borowski, Beata Jacus, Marcin Konarzewski, Daniel  Potorski, Andrzej Urbaniak |
| Poland | Waldemar Halota | Edyta Grabczewska, Anita Olczak |
| Poland | Grzegorz Kania | Maciej Kania, Joanna Kania-Niklas, Ewa Okonska-Jagoda |
| Russia | Yury Grigorievich Shvarts | Mikhail Aristarin, Svetlana Ermasova, Anastasia Khasheva |
| Russia | Roman S. Kozlov | Aleksandr Alekseevich Muravev, Anatoly Aleksandrovich  Nikulin |
| Russia | Ilsiyar Khaertynova | Alisa Rasimovna Bilalova, Elza Gaifullina, Venera Shakirova |
| Australia | Mark Theo Bloch | Timothy G Barnes, Katharine Jane Bessey, Robert Burton, Jacqueline Engelander, Andrew Gowers, Jane Hunt, Gary Lee, Dick Cheong Quan, Shiva Rayar |
| Australia | Sheetal Bull | James Vandeleur |
| Australia | James Vandeleur /  Joshua Kim | Sheetal Bull, Gregor Cantlay |
| Australia | Andrew Ostor | Stephen Hall, Nicole McKay, Louise Murdoch |
| Australia | Peter Clyne / Ushma Narsai / Mariam Chaalan | Christopher Argent, Mariam Chaalan, Dimuthu Samaranayake, James Vandeleur |
| Australia | David Martin Colquhoun | Jane Anne Hamlyn, Lynette Denise Williams |
| Australia | Martin Weltman | Kyung Ho Choi, Grace Lee, Neha Tiwari |
| New Zealand | Simon J. Carson | Monica Leeanne Ford |
| New Zealand | Michael John Williams | Dean Tasker |
| New Zealand | Barney Montgomery | Summer Hassan, Annika Lam, Ryan Yeu |
| New Zealand | Susan Smith | John Derek Anthony Richmond |
| New Zealand | Dean Richard Quinn | Joanna Victoria Joseph |
| New Zealand | Diane Hanfelt-Goade | Philip Garden, James Heaton |
| New Zealand | Edward Gane | Leanne Barnett, Paul Hamilton, Mitee Kushant Kapadia, Bridget Maher, Christian Schwabe, Sophie Wynne |
| New Zealand | Richard William Troughton | Danielle Lea Thompson |

If the original investigator has been replaced, that investigator’s name is followed by a slash (/) and the replacement investigator’s name.

Supplementary Table 2. Study Inclusion and Exclusion Criteria.

| **Inclusion criteria** |
| --- |
| 1. Male or female 18–49 years of age (inclusive) |
| 1. Native American from clinical sites of the Center for American Indian Health (CAIH) in good health without any of the risk conditions for pneumococcal disease listed below (any underlying chronic illness must be documented to be in stable condition) |
| **OR** |
| Native American from clinical sites of the CAIH OR a participant from sites other than the CAIH with ≥1 of the following risk conditions for pneumococcal disease: |
| 1. Diabetes mellitus Type 1 or Type 2, receiving treatment with at least 1 approved antidiabetic medication; with hemoglobin A1c <10% at screening (Visit 1) |
| 1. Chronic liver disease with compensated cirrhosis (Child-Pugh Class A) due to non-alcoholic fatty liver disease, chronic hepatitis B, chronic hepatitis C, or alcoholic liver disease, diagnosed by clinician’s assessment, with at least 1 of the following liver staging assessments (Note: In cases where more than 1 staging assessment is available, only 1 result may be used to determine fibrosis status. Biopsy should be preferred over every other test and FibroScan^®^ over the blood test-based assessment. Participants with hepatitis C must have either completed a course of treatment with direct antiviral therapy and had a 12-week treatment-free follow-up or be expected to remain untreated during the duration of this study): |
| 1. Prior liver biopsy demonstrating cirrhosis |
| 1. FibroScan performed with an interpretable score >12.5 kPa within 5 years of Visit 1 (screening) |
| 1. A FibroTest^®^ (FibroSure^®^) with Fibrosis Score >0.75 performed within 5 years of Visit 1 and an aspartate aminotransferase (AST): platelet ratio index (APRI) of >2 at Visit 1 (screening). APRI formula: AST ÷ lab upper limit of normal for AST x 100 ÷ (platelet count ÷ 100) (APRI calculation to be provided by the central laboratory) |
| 1. Prior imaging study with evidence of cirrhosis and an APRI of >2 at Visit 1 (screening) |
| 1. Prior imaging study with evidence of cirrhosis with splenomegaly and platelet count <120,000/μL at Visit 1 (Screening) |
| 1. Confirmed diagnosis of chronic obstructive pulmonary disease (COPD) with spirometric data in the preceding 5 years showing post-bronchodilator forced expiratory volume in 1 second (FEV_1_) over forced vital capacity (FVC) ratio (FEV_1_/FVC) <0.7, and FEV_1_ ≥30% predicted, corresponding to spirometric Global Initiative for Chronic Obstructive Lung Disease (GOLD) Stage 1 to 3 by severity assessment. For otherwise eligible individuals with a clinical history of COPD who do not have spirometry data from the preceding 5 years, spirometry can be performed at Visit 1 during the screening process by adequately trained personnel according to accepted guidelines |
| 1. Confirmed diagnosis of mild or moderate persistent asthma with documented reversible airflow obstruction on spirometry consistent with guidelines for the diagnosis of asthma performed within 5 years prior to Visit 1, and receipt of guideline-directed therapy for mild-to-moderate asthma. For otherwise eligible individuals with a clinical history of asthma who are receiving guideline directed therapy for mild-to-moderate asthma but do not have spirometry data from the previous 5 years, spirometry can be performed at Visit 1 during the screening process by adequately trained personnel according to accepted guidelines for assessing reversibility. Inhaled bronchodilator and/or inhaled steroid therapy should be withheld prior to spirometry for a “wash-out” as per recommended guidelines |
| 1. Confirmed diagnosis of chronic heart disease due to 1 of the following conditions documented within the last 5 years (Note: Participants must have New York Heart Association (NYHA) heart failure Class 1 to 3 at Visit 1 (screening) and receive guideline-directed oral heart failure treatment): |
| 1. Heart failure with reduced ejection fraction (EF): transthoracic echocardiography (TTE) or transesophageal echocardiography (TEE) with left ventricular EF <40% |
| 1. Heart failure with preserved EF as diagnosed by a cardiologist based on clinical signs and symptoms, a TTE/TEE with left ventricular EF ≥50%, and either 1) plasma brain natriuretic peptide (BNP) >100 pg/mL or NT-proBNP >300 pg/mL or 2) at least 1 prior hospitalization or emergency room visit for heart failure |
| 1. Non-cyanotic congenital heart disease |
| 1. Current smoker who has smoked at least 100 cigarettes during lifetime, is currently smoking every day or most days of the week, and is not currently receiving smoking cessation therapy at Visit 1 (screening) or planning to receive therapy during the study 2. Based on the known association of alcohol abuse/misuse with increased morbidity and mortality of PD,^1-4^ all participants completed an Alcohol Use Disorder Identification Test-Concise (AUDIT-C) at screening. An AUDIT-C score of ≥5 identified individuals with potentially harmful alcohol use and counted as an additional risk factor for stratification purposes.^5^ |
| 1. Receiving stable medical management for the conditions listed in Inclusion Criterion 1a–f, if applicable, for at least 3 months with no anticipated major change expected for the duration of the study |
| 1. A female participant is eligible to participate if she is not pregnant, not breastfeeding, and at least 1 of the following conditions applies: |
| 1. Not a woman of childbearing potential (WOCBP) |
| **OR** |
| 1. A WOCBP who agrees to use contraceptive methods during the treatment period and for at least 6 weeks after the last dose of study intervention |
| 1. The participant provides written informed consent for the study. The participant may also provide consent for future biomedical research. However, the participant may participate in the main study without participating in future biomedical research. Native American participants enrolled via the CAIH will not participate in future biomedical research |
| **Exclusion criteria** |
| 1. History of active hepatitis with elevation in pre-treatment aspartate transaminase (AST) or alanine transaminase (ALT) values >5 times the upper limit of normal within 3 months of Visit 1 (screening) |
| 1. History of diabetic ketoacidosis or >1 episode of severe, symptomatic hypoglycemia within 3 months of Visit 1 (screening) |
| 1. Myocardial infarction, acute coronary syndrome, transient ischemic attack, or ischemic or hemorrhagic stroke within 3 months of Visit 1 (screening) |
| 1. History of severe pulmonary hypertension with World Health Organization functional class ≥3 or history of Eisenmenger syndrome |
| 1. History of invasive pneumococcal disease (positive blood culture, positive cerebrospinal fluid culture, or positive culture at another sterile site) or known history of other culture-positive pneumococcal disease within 3 years of Visit 2 (Day 1) |
| 1. Known hypersensitivity to any component of pneumococcal polysaccharide vaccine, pneumococcal conjugate vaccine, or any diphtheria toxoid-containing vaccine |
| 1. Known or suspected impairment of immunological function including, but not limited to a history of congenital or acquired immunodeficiency, documented HIV infection, functional or anatomic asplenia, or history of autoimmune disease |
| 1. History of malignancy ≤5 years prior to signing informed consent (including hepatocellular carcinoma), except for adequately treated basal cell or squamous cell skin cancer or *in situ* cervical cancer |
| 1. History of Stage 4 or 5 chronic kidney disease (glomerular filtration rate <30 mL/min/1.73 m^2^) or nephrotic syndrome |
| 1. History of alcohol withdrawal or alcohol withdrawal seizure in the past 12 months |
| 1. History of coagulation disorder contraindicating intramuscular vaccinations |
| 1. *Recent febrile illness (defined as oral or tympanic temperature ≥100.4°F [≥38.0°C]; axillary or temporal temperature ≥99.4°F [≥37.4°C]; or rectal temperature ≥101.4°F [≥38.6°C]) or received antibiotic therapy for any acute illness occurring within 72 hours before receipt of study vaccine |
| 1. History of hospitalization within 3 months of Visit 1 (screening) |
| 1. Planned organ transplantation (heart, liver, lung, kidney, or pancreas) or other planned major surgery during the duration of this study |
| 1. Expected survival for less than 1 year according to the investigator’s judgment |
| 1. A woman of childbearing potential who has a positive urine or serum pregnancy test before the first vaccination at Visit 2 (Day 1) |
| 1. Prior administration of any pneumococcal vaccine or expected to receive any pneumococcal vaccine during the study outside of the protocol |
| 1. Received systemic corticosteroids (prednisone equivalent of ≥20 mg/day) for ≥14 consecutive days and has not completed intervention at least 30 days before study entry |
| 1. Received systemic corticosteroids exceeding physiologic replacement doses (approximately 5 mg/day prednisone equivalent) within 14 days before vaccination. (Note: Topical, ophthalmic, intra-articular or soft tissue [eg, bursa, tendon steroid injections], and inhaled/nebulized steroids are permitted) |
| 1. Receiving immunosuppressive therapy, including chemotherapeutic agents used to treat cancer or other conditions, and interventions associated with organ or bone marrow transplantation, or autoimmune disease |
| 1. Receiving immunomodulatory therapy with biological agents such as monoclonal antibodies directed against interleukin or cytokine pathways that could potentially interfere with immunogenicity assessment |
| 1. Received any licensed, non-live vaccine within the 14 days before receipt of any study vaccine or is scheduled to receive any licensed, non-live vaccine within 30 days following receipt of any study vaccine. Exception: Inactivated influenza vaccine may be administered but must be given at least 7 days before receipt of any study vaccine or at least 15 days after receipt of any study vaccine^a^ |
| 1. Received any live vaccine within 30 days before receipt of any study vaccine or is scheduled to receive any live vaccine within 30 days following receipt of any study vaccine^a^ |
| 1. Received a blood transfusion or blood products, including immunoglobulins within the 6 months before receipt of study vaccine or is scheduled to receive a blood transfusion or blood product within 30 days of receipt of study vaccine. Autologous blood transfusions are not considered an exclusion criterion |
| 1. Receiving chronic home oxygen therapy |
| 1. Is currently participating in or has participated in an interventional clinical study with an investigational compound or device within 2 months of participating in this current study |
| 1. Is, at the time of signing informed consent, a user of recreational or illicit drugs or has had a recent history (within the last year) of drug abuse or dependence as assessed by the study investigator |
| 1. Has history or current evidence of any condition, therapy, laboratory abnormality or other circumstance that might expose the participant to risk by participating in the study, confound the results of the study, or interfere with the participant’s participation for the full duration of the study |
| 1. Is or has an immediate family member (eg, spouse, parent/legal guardian, sibling, or child) who is investigational site or Sponsor staff directly involved with this study |
| 1. Diabetes mellitus with hemoglobin A1c ≥10% at Visit 1 (screening) |
| 1. Chronic liver disease with Child-Pugh Class B or C cirrhosis at Visit 1 (screening) |
| 1. Chronic lung disease with chronic obstructive pulmonary disease GOLD Stage 4 or severe persistent asthma at Visit 1 (screening) |
| 1. Chronic heart disease with New York Heart Association (NYHA) heart failure Class 4 at Visit 1 (screening) |

^a^If the participant meets these exclusion criteria at screening, the Day 1 visit may be rescheduled for a time when these criteria are not met.

Supplementary Table 3. Maximum Duration of Solicited AEs After Vaccination with V114 or PCV13.

|  | **V114 n = 1134** | | **PCV13 n = 378** | |
| --- | --- | --- | --- | --- |
|  | **n** | **(%)** | **n** | **(%)** |
| **With ≥1 solicited AE** | 934 | (82.4) | 298 | (78.8) |
| ≤3 days | 661 | (58.3) | 214 | (56.6) |
| >3 days and ≤5 days | 147 | (13.0) | 49 | (13.0) |
| >5 days and ≤10 days | 85 | (7.5) | 19 | (5.0) |
| >10 days | 41 | (3.6) | 16 | (4.2) |
| **Solicited injection-site AEs** |  |  |  |  |
| Injection-site erythema | 171 | (15.1) | 53 | (14.0) |
| ≤3 days | 129 | (11.4) | 36 | (9.5) |
| >3 days and ≤5 days | 20 | (1.8) | 15 | (4.0) |
| >5 days and ≤10 days | 20 | (1.8) | 1 | (0.3) |
| >10 days | 2 | (0.2) | 1 | (0.3) |
| Injection-site pain | 860 | (75.8) | 260 | (68.8) |
| ≤3 days | 695 | (61.3) | 209 | (55.3) |
| >3 days and ≤5 days | 131 | (11.6) | 37 | (9.8) |
| >5 days and ≤10 days | 29 | (2.6) | 9 | (2.4) |
| >10 days | 5 | (0.4) | 5 | (1.3) |
| Injection-site swelling | 246 | (21.7) | 84 | (22.2) |
| ≤3 days | 188 | (16.6) | 65 | (17.2) |
| >3 days and ≤5 days | 37 | (3.3) | 15 | (4.0) |
| >5 days and ≤10 days | 18 | (1.6) | 3 | (0.8) |
| >10 days | 3 | (0.3) | 1 | (0.3) |
| **Solicited systemic AEs** |  |  |  |  |
| Arthralgia | 144 | (12.7) | 44 | (11.6) |
| ≤3 days | 112 | (9.9) | 40 | (10.6) |
| >3 days and ≤5 days | 14 | (1.2) | 1 | (0.3) |
| >5 days and ≤10 days | 10 | (0.9) | 2 | (0.5) |
| >10 days | 8 | (0.7) | 1 | (0.3) |
| Fatigue | 389 | (34.3) | 139 | (36.8) |
| ≤3 days | 304 | (26.8) | 101 | (26.7) |
| >3 days and ≤5 days | 33 | (2.9) | 19 | (5.0) |
| >5 days and ≤10 days | 35 | (3.1) | 13 | (3.4) |
| >10 days | 17 | (1.5) | 6 | (1.6) |
| Headache | 300 | (26.5) | 94 | (24.9) |
| ≤3 days | 250 | (22.0) | 76 | (20.1) |
| >3 days and ≤5 days | 22 | (1.9) | 7 | (1.9) |
| >5 days and ≤10 days | 17 | (1.5) | 6 | (1.6) |
| >10 days | 11 | (1.0) | 5 | (1.3) |
| Myalgia | 327 | (28.8) | 100 | (26.5) |
| ≤3 days | 262 | (23.1) | 80 | (21.2) |
| >3 days and ≤5 days | 40 | (3.5) | 11 | (2.9) |
| >5 days and ≤10 days | 19 | (1.7) | 5 | (1.3) |
| >10 days | 6 | (0.5) | 4 | (1.1) |

Every participant is counted a single time for each applicable specific adverse event and is classified according to the longest duration. Injection-site erythema, injection-site pain, and injection-site swelling were solicited from Day 1 to Day 5 following vaccination. Arthralgia, fatigue, headache, and myalgia were solicited from Day 1 to Day 14 following vaccination. Ongoing adverse events are categorized as having duration >10 days.

Abbreviations: AE, adverse event; PCV13, 13-valent pneumococcal conjugate vaccine; V114, 15-valent pneumococcal conjugate vaccine.

Supplementary Table 4. Maximum Duration of Solicited AEs After Vaccination PPSV23.

|  | **V114 n = 1036** | | **PCV13 n = 345** | |
| --- | --- | --- | --- | --- |
|  | **n** | **(%)** | **n** | **(%)** |
| **With ≥1 solicited AE** | 771 | (74.4) | 258 | (74.8) |
| ≤3 days | 549 | (53.0) | 186 | (53.9) |
| >3 days and ≤5 days | 155 | (15.0) | 52 | (15.1) |
| >5 days and ≤10 days | 52 | (5.0) | 10 | (2.9) |
| >10 days | 15 | (1.4) | 10 | (2.9) |
| **Solicited injection-site AEs** |  |  |  |  |
| Injection-site erythema | 234 | (22.6) | 88 | (25.5) |
| ≤3 days | 183 | (17.7) | 73 | (21.2) |
| >3 days and ≤5 days | 39 | (3.8) | 10 | (2.9) |
| >5 days and ≤10 days | 11 | (1.1) | 2 | (0.6) |
| >10 days | 1 | (0.1) | 3 | (0.9) |
| Injection-site pain | 713 | (68.8) | 231 | (67.0) |
| ≤3 days | 555 | (53.6) | 187 | (54.2) |
| >3 days and ≤5 days | 131 | (12.6) | 35 | (10.1) |
| >5 days and ≤10 days | 24 | (2.3) | 6 | (1.7) |
| >10 days | 3 | (0.3) | 3 | (0.9) |
| Injection-site swelling | 305 | (29.4) | 111 | (32.2) |
| ≤3 days | 241 | (23.3) | 93 | (27.0) |
| >3 days and ≤5 days | 48 | (4.6) | 14 | (4.1) |
| >5 days and ≤10 days | 14 | (1.4) | 3 | (0.9) |
| >10 days | 2 | (0.2) | 1 | (0.3) |
| **Solicited systemic AEs** |  |  |  |  |
| Arthralgia | 124 | (12.0) | 38 | (11.0) |
| ≤3 days | 100 | (9.7) | 34 | (9.9) |
| >3 days and ≤5 days | 16 | (1.5) | 3 | (0.9) |
| >5 days and ≤10 days | 3 | (0.3) | 1 | (0.3) |
| >10 days | 5 | (0.5) | 0 | (0.0) |
| Fatigue | 312 | (30.1) | 106 | (30.7) |
| ≤3 days | 254 | (24.5) | 89 | (25.8) |
| >3 days and ≤5 days | 32 | (3.1) | 11 | (3.2) |
| >5 days and ≤10 days | 16 | (1.5) | 3 | (0.9) |
| >10 days | 10 | (1.0) | 3 | (0.9) |
| Headache | 220 | (21.2) | 73 | (21.2) |
| ≤3 days | 187 | (18.1) | 61 | (17.7) |
| >3 days and ≤5 days | 19 | (1.8) | 8 | (2.3) |
| >5 days and ≤10 days | 14 | (1.4) | 1 | (0.3) |
| >10 days | 0 | (0.0) | 3 | (0.9) |
| Myalgia | 250 | (24.1) | 88 | (25.5) |
| ≤3 days | 215 | (20.8) | 70 | (20.3) |
| >3 days and ≤5 days | 26 | (2.5) | 14 | (4.1) |
| >5 days and ≤10 days | 7 | (0.7) | 2 | (0.6) |
| >10 days | 2 | (0.2) | 2 | (0.6) |

Every participant is counted a single time for each applicable specific adverse event and is classified according to the longest duration. Injection-site erythema, injection-site pain, and injection-site swelling were solicited from Day 1 to Day 5 following vaccination. Arthralgia, fatigue, headache, and myalgia were solicited from Day 1 to Day 14 following vaccination. Ongoing adverse events are categorized as having duration >10 days.

Abbreviations: AE, adverse event; PCV13, 13-valent pneumococcal conjugate vaccine; PPSV23, 23-valent pneumococcal polysaccharide vaccine; V114, 15-valent pneumococcal conjugate vaccine.

# Supplementary Table 5. Summary of OPA Responses.

|  | **Endpoint** | **Timepoint** | **V114 (N = 1133)** | | | **PCV13 (N = 379)** | | |
| --- | --- | --- | --- | --- | --- | --- | --- | --- |
| **13 shared serotypes** | | | **n** | **Observed response** | **(95% CI)^a^** | **n** | **Observed response** | **(95% CI)^a^** |
| **1** | **GMT** | Day 1  Day 30  Month 6  Month 7 | 1100  1019  946  841 | 7.0  268.6  79.5  266.6 | (6.6–7.5)  (243.7–296.0)  (71.7–88.3)  (243.6–291.8) | 368  341  315  281 | 7.5  267.2  95.8  214.4 | (6.7–8.3)  (220.4–323.9)  (78.6–116.7)  (180.7–254.5) |
|  | **GMFR** | Day 1 to Day 30  Day 1 to Month 7  Month 6 to Month 7 | 1001  826  839 | 22.8  22.6  32 | (20.6–25.1)  (20.6–24.8)  (2.9–3.5) | 334  276  280 | 21.9  17.4  2.0 | (18.4–26.2)  (14.8–20.5)  (1.7–2.2) |
|  | **% ≥4-fold rise** | Day 1 to Day 30  Day 1 to Month 7  Month 6 to Month 7 | 1001  826  839 | 83.9% (840/1001)  87.7% (724/826)  40.2% (337/839) | (81.5–86.1)  (85.2–89.8)  (36.8–43.6) | 334  276  280 | 81.4% (272/334)  83.7% (231/276)  21.4% (60/280) | (76.8–85.5)  (78.8–87.9)  (16.8–26.7) |
| **3** | **GMT** | Day 1  Day 30  Month 6  Month 7 | 1095  1004  932  837 | 23.6  199.3  102.9  211.0 | (21.9–25.4)  (184.6–215.2)  (94.9–111.4)  (195.2–228.1) | 363  340  311  279 | 21.0  150.6  77.6  208.0 | (18.5–23.7)  (130.6–173.8)  (67.0–89.8)  (179.7–240.7) |
|  | **GMFR** | Day 1 to Day 30  Day 1 to Month 7  Month 6 to Month 7 | 982  820  826 | 5.8  6.1  2.0 | (5.4–6.3)  (5.6–6.6)  (1.9–2.1) | 329  270  274 | 4.8  6.4  2.4 | (4.3–5.5)  (5.6–7.3)  (2.2–2.7) |
|  | **% ≥4-fold rise** | Day 1 to Day 30  Day 1 to Month 7  Month 6 to Month 7 | 982  820  826 | 62.2% (611/982)  66.5% (545/820)  19.2% (159/826) | (59.1–65.3)  (63.1–69.7)  (16.6–22.1) | 329  270  274 | 56.8% (187/329)  66.7% (180/270)  25.5% (70/274) | (51.3–62.3)  (60.7–72.3)  (20.5–31.1) |
| **4** | **GMT** | Day 1  Day 30  Month 6  Month 7 | 1090  1016  945  840 | 53.8  1416.0  630.1  1734.5 | (48.9–59.1)  (1308.9–1531.8)  (576.5–688.7)  (1620.7–1856.4) | 361  342  315  283 | 52.1  2576.1  1100.9  1980.6 | (44.4–61.1)  (2278.0–2913.2)  (952.5–1272.3)  (1771.3–2214.6) |
|  | **GMFR** | Day 1 to Day 30  Day 1 to Month 7  Month 6 to Month 7 | 988  820  837 | 17.9  21.6  2.7 | (16.2–19.8)  (19.5–23.9)  (2.5–2.9) | 329  273  281 | 33.4  25.6  1.7 | (28.1–39.7)  (21.7–30.3)  (1.5–1.9) |
|  | **% ≥4-fold rise** | Day 1 to Day 30  Day 1 to Month 7  Month 6 to Month 7 | 988  820  837 | 79.0% (781/988)  84.3% (691/820)  30.8% (258/837) | (76.4–81.5)  (81.6–86.7)  (27.7–34.1) | 329  273  281 | 87.8% (289/329)  86.4% (236/273)  14.9% (42/281) | (83.8–91.2)  (81.8–90.3)  (11.0–19.7) |
| **5** | **GMT** | Day 1  Day 30  Month 6  Month 7 | 1103  1018  944  844 | 19.4  564.8  209.3  595.1 | (18.4–20.5)  (512.7–622.2)  (189.1–231.5)  (544.5–650.5) | 369  343  316  283 | 19.9  731.1  258.5  626.7 | (18.1–21.8)  (613.6–871.0)  (213.4–313.1)  (531.7–738.7) |
|  | **GMFR** | Day 1 to Day 30  Day 1 to Month 7  Month 6 to Month 7 | 1003  831  840 | 17.3  17.8  2.7 | (15.7–18.9)  (16.3–19.3)  (2.5–2.9) | 337  279  282 | 21.1  18.3  2.1 | (18.0–24.7)  (15.8–21.1)  (1.9–2.4) |
|  | **% ≥4-fold rise** | Day 1 to Day 30  Day 1 to Month 7  Month 6 to Month 7 | 1003  831  840 | 83.4% (837/1003)  86.8% (721/831)  32.5% (273/840) | (81.0–85.7)  (84.3–89.0)  (29.3–35.8) | 337  279  282 | 84.9% (286/337)  87.1% (243/279)  23.0% (65/282) | (80.6–88.5)  (82.6–90.8)  (18.3–28.4) |
| **6A** | **GMT** | Day 1  Day 30  Month 6  Month 7 | 1010  1006  929  830 | 454.6  12 928.8  5589.7  5810.3 | (417.9–494.5)  (11 923.4–14 019.0)  (5157.5–6058.1)  (5366.9–6290.3) | 335  335  311  276 | 379.3  11 282.4  4814.2  5739.9 | (329.7–436.4)  (9718.8–13 097.5)  (4203.8–5513.3)  (4974.4–6623.1) |
|  | **GMFR** | Day 1 to Day 30  Day 1 to Month 7  Month 6 to Month 7 | 910  754  818 | 21.7  9.6  1.0 | (19.6–23.9)  (8.7–10.5)  (1.0–1.1) | 298  247  270 | 21.4  11.0  1.1 | (18.0–25.5)  (9.3–13.0)  (1.0–1.2) |
|  | **% ≥4-fold rise** | Day 1 to Day 30  Day 1 to Month 7  Month 6 to Month 7 | 910  754  818 | 87.5% (796/910)  73.5% (554/754)  3.9% (32/818) | (85.1 –89.6)  (70.2–76.6)  (2.7–5.5) | 298  247  270 | 85.2% (254/298)  80.2% (198/247)  5.9% (16/270) | (80.7–89. 1)  (74.6–84.9)  (3.4–9.4) |
| **6B** | **GMT** | Day 1  Day 30  Month 6  Month 7 | 1070  1014  945  843 | 248.4  10 336.9  4686.7  5215.2 | (220.1–280.5)  (9649.4–11 073.4)  (4354.7–5044.1)  (4863.6–5592.2) | 360  342  314  283 | 225.7  6995.7  3383.1  4412.4 | (183.8–277.2)  (6024.7–8123.2)  (2913.1–3929.0)  (3892.8–5001.5) |
|  | **GMFR** | Day 1 to Day 30  Day 1 to Month 7  Month 6 to Month 7 | 969  802  842 | 32.2  16.6  1.1 | (28.6–36.3)  (14.7–18.7)  (1.1–1.2) | 328  273  280 | 25.0  15.7  1.2 | (20.6–30.5)  (12.8–19.2)  (1.1–1.4) |
|  | **% ≥4-fold rise** | Day 1 to Day 30  Day 1 to Month 7  Month 6 to Month 7 | 969  802  842 | 84.7% (821/969)  75.8% (608/802)  4.6% (39/842) | (82.3–86.9)  (72.7–78.7)  (3.3–6.3) | 328  273  280 | 83.8% (275/328)  74.4% (203/273)  6.4% (18/280) | (79.4–87.7)  (68.7–79.4)  (3.9–10.0) |
| **7F** | **GMT** | Day 1  Day 30  Month 6  Month 7 | 1032  1019  942  843 | 667.3  5756.4  3265.3  6070.5 | (598.7–744.3)  (5410.4–6124.6)  (3075.5–3466.7)  (5699.7–6465.6) | 348  342  315  283 | 645.4  7588.9  4240.4  6223.9 | (536.2–776.9)  (6775.3–8500.2)  (3828.0–4697.3)  (5595.3–6923.0) |
|  | **GMFR** | Day 1 to Day 30  Day 1 to Month 7  Month 6 to Month 7 | 945  777  837 | 7.3  7.7  1.9 | (6.6–8.2)  (6.9–8.6)  (1.8–2.0) | 318  264  281 | 10.0  8.2  1.4 | (8.3–1 2.0)  (6.7–9.9)  (1.3–1.6) |
|  | **% ≥4-fold rise** | Day 1 to Day 30  Day 1 to Month 7  Month 6 to Month 7 | 945  777  837 | 56.8% (537/945)  59.8% (465/777)  14.6% (122/837) | (53.6–60.0)  (56.3–63.3)  (12.3–17.2) | 318  264  281 | 64.5% (205/318)  60.2% (159/264)  7.5% (21/281) | (58.9–69.7)  (54.0–66.2)  (4.7–11.2) |
| **9V** | **GMT** | Day 1  Day 30  Month 6  Month 7 | 1079  1015  940  842 | 582.4  3355.1  1891.6  3133.1 | (535.8–633.1)  (3135.4–3590.1)  (1767.4–2024.5)  (2918.4–3363.7) | 364  343  313  282 | 602.1  3983.7  2176.6  3364.1 | (521.2–695.7)  (3557.8–4460.7)  (1916.0–2472.8)  (2972.2–3807.6) |
|  | **GMFR** | Day 1 to Day 30  Day 1 to Month 7  Month 6 to Month 7 | 977  810  835 | 4.9  4.5  1.6 | (4.5–5.3)  (4.2–4.9)  (1.5–1.7) | 332  273  278 | 5.7  4.9  1.5 | (5.0–6.6)  (4.2–5.7)  (1.4–1.7) |
|  | **% ≥4-fold rise** | Day 1 to Day 30  Day 1 to Month 7  Month 6 to Month 7 | 977  810  835 | 51.5% (503/977)  50.5% (409/810)  11.9% (99/835) | (48.3–54.7)  (47.0–54.0)  (9.7–1 4.2) | 332  273  278 | 55.4% (184/332)  52.4% (143/273)  10.4% (29/278) | (49.9–60.8)  (46.3–58.4)  (7.1–14.6) |
| **14** | **GMT** | Day 1  Day 30  Month 6  Month 7 | 1073  1016  946  843 | 537.1  5228.9  2929.5  5644.9 | (483.9–596.1)  (4847.6–5640.2)  (2718.3–3157.0)  (5262.5–6055.2) | 363  343  314  283 | 573.0  5889.8  3560.1  5317.6 | (481.7–681.5)  (5218.2–6647.8)  (3123.3–4057.9)  (4686.1–6034.1) |
|  | **GMFR** | Day 1 to Day 30  Day 1 to Month 7  Month 6 to Month 7 | 973  809  841 | 8.2  9.2  2.0 | (7.4–9.2)  (8.2–10.3)  (1.8–2.1) | 331  275  280 | 8.7  8.0  1.5 | (7.2–10.6)  (6.6–9.7)  (1.3–1.6) |
|  | **% ≥4-fold rise** | Day 1 to Day 30  Day 1 to Month 7  Month 6 to Month 7 | 973  809  841 | 59.8% (582/973)  64.9% (525/809)  17.8% (150/841) | (56.7–62.9)  (61.5–68.2)  (15.3–20.6) | 331  275  280 | 60.4% (200/331)  58.9% (162/275)  8.6% (24/280) | (54.9–65.7)  (52.8–64.8)  (5.6–12.5) |
| **18C** | **GMT** | Day 1  Day 30  Month 6  Month 7 | 1075  1014  945  842 | 194.0  5709.0  2501.1  3260.6 | (177.9–211.6)  (5331.1–6113.6)  (2332.9–2681.5)  (3057.3–3477.5) | 361  343  315  281 | 187.6  3063.2  1473.3  2294.4 | (161.8–217.4)  (2699.8–3475.5)  (1296.1–1674.7)  (2052.5–2564.8) |
|  | **GMFR** | Day 1 to Day 30  Day 1 to Month 7  Month 6 to Month 7 | 973  805  839 | 20.4  11.1  1.3 | (18.6–22.4)  (10.2–12.2)  (1.3–1.4) | 331  273  279 | 11.7  9.1  1.6 | (10.1–13.6)  (7.9–10.5)  (1.4–1.7) |
|  | **% ≥4-fold rise** | Day 1 to Day 30  Day 1 to Month 7  Month 6 to Month 7 | 973  805  839 | 84.5% (822/973)  77.5% (624/805)  5.4% (45/839) | (82.1–86.7)  (74.5–80.4)  (3.9–7.1) | 331  273  279 | 75.8% (251/331)  76.2% (208/273)  14.3% (40/279) | (70.8–80.3)  (70.7–81.1)  (10.4–19.0) |
| **19A** | **GMT** | Day 1  Day 30  Month 6  Month 7 | 1076  1015  942  836 | 394.0  5369.9  2542.3  4336.2 | (358.8–432.7)  (5017.7–5746.8)  (2379.3–2716.4)  (4038.6–4655.6) | 358  343  316  283 | 392.6  5888.0  2721.2  4286.4 | (334.8–460.5)  (5228.2–6631.0)  (2436.7–3039.1)  (3838.6–4786.4) |
|  | **GMFR** | Day 1 to Day 30  Day 1 to Month 7  Month 6 to Month 7 | 975  806  830 | 12.5  9.6  1.7 | (11.3–13.9)  (8.6–10.6)  (1.6–1.8) | 326  271  282 | 13.6  9.7  1.5 | (11.4–16.2)  (8.1 –11.6)  (1.4–1.7) |
|  | **% ≥4-fold rise** | Day 1 to Day 30  Day 1 to Month 7  Month 6 to Month 7 | 975  806  830 | 72.1% (703/975)  68.2% (550/806)  15.9% (132/830) | (69.2–74.9)  (64.9–71.4)  (13.5–18.6) | 326  271  282 | 75.2% (245/326)  70.5% (191/271)  12.1% (34/282) | (70.1–79.8)  (64.7–75.8)  (8.5–16.4) |
| **19F** | **GMT** | Day 1  Day 30  Month 6  Month 7 | 1084  1018  946  844 | 364.2  3266.3  1654.7  3198.6 | (335.2–395.8)  (3064.4–3481.4)  (1549.1–1767.4)  (3011.0–3397.8) | 361  343  315  282 | 362.6  3272.7  1778.8  3085.4 | (313.0–420.0)  (2948.2–3632.9)  (1587.2–1993.6)  (2770.7–3435.9) |
|  | **GMFR** | Day 1 to Day 30  Day 1 to Month 7  Month 6 to Month 7 | 985  818  843 | 7.5  6.9  1.9 | (6.9–8.2)  (6.3–7.6)  (1.8–2.0) | 329  272  280 | 7.4  7.1  1.6 | (6.4–8.6)  (6.1 –8.3)  (1.5–1.8) |
|  | **% ≥4-fold rise** | Day 1 to Day 30  Day 1 to Month 7  Month 6 to Month 7 | 985  818  843 | 64.3% (633/985)  61.0% (499/818)  17.7% (149/843) | (61.2–67.3)  (57.6–64.4)  (15.2–20.4) | 329  272  280 | 65.7% (216/329)  62.9% (171/272)  12.9% (36/280) | (60.2–70.8)  (56.8–68.6)  (9.2–17.4) |
| **23F** | **GMT** | Day 1  Day 30  Month 6  Month 7 | 1043  1016  946  839 | 153.8  4853.5  2365.7  3057.3 | (137.0–172.7)  (4469.8–5270.2)  (2167.4–2582.1)  (2823.0–3311.0) | 344  340  314  283 | 164.2  3887.3  2117.8  2896.0 | (134.9–199.9)  (3335.8–4530.0)  (1799.8–2492.1)  (2494.1–3362.7) |
|  | **GMFR** | Day 1 to Day 30  Day 1 to Month 7  Month 6 to Month 7 | 945  780  837 | 22.5  14.0  1.3 | (20.0–25.4)  (12.4–15.7)  (1.2–1.4) | 310  260  280 | 17.8  12.6  1.4 | (14.6–21.7)  (10.3–15.4)  (1.2–1.6) |
|  | **% ≥4-fold rise** | Day 1 to Day 30  Day 1 to Month 7  Month 6 to Month 7 | 945  780  837 | 78.7% (744/945)  73 .5% (573/780)  9.2% (77/837) | (76.0–81.3)  (70.2–76.5)  (7.3–11.4) | 310  260  280 | 77.7% (241/310)  71.9% (187/260)  11.4% (32/280) | (72.7–82.2)  (66.0–77.3)  (7.9–15.7) |
| **2 serotypes unique to V114** | | |  |  |  |  |  |  |
| **22F** | **GMT** | Day 1  Day 30  Month 6  Month 7 | 985  1005  932  837 | 227.0  3926.5  2054.4  3624.0 | (194.4–265.0)  (3645.9–4228.7)  (1909.0–2210.8)  (3384.5–3880.3) | 341  320  286  280 | 190.9  291.6  335.6  4060.2 | (145.5–250.4)  (221.8–383.6)  (250.9–449.1)  (3358.6–4908.4) |
|  | **GMFR** | Day 1 to Day 30  Day 1 to Month 7  Month 6 to Month 7 | 885  742  822 | 13.9  12.1  1.8 | (11.8–16.3)  (10.4–14.3)  (1.7–1.9) | 290  254  253 | 1.3  16.6  9.6 | (1.1–1.6)  (12.2–22.6)  (7.1–13 .0) |
|  | **% ≥4-fold rise** | Day 1 to Day 30  Day 1 to Month 7  Month 6 to Month 7 | 885  742  822 | 58.9% (521/885)  59.0% (438/742)  16.7% (137/822) | (55.5–62.1)  (55.4–62.6)  (14.2–19.4) | 290  254  253 | 15.5% (45/290)  65.4% (166/254)  52.6% (133/253) | (11.5–20.2)  (59.2–71.2)  (46.2–58.9) |
| **33F** | **GMT** | Day 1  Day 30  Month 6  Month 7 | 1083  1014  945  837 | 2178.4  11 627.8  6852.0  11 356.6 | (2000.6–2371.9)  (10 824.6–12 490.7)  (6398.7–7337.4)  (10 492.4–12 291.9) | 363  338  313  282 | 2333.8  2180.6  2373.5  16 053.2 | (1997.5–2726.9)  (1828.7–2600.2)  (2021.5–2786.8)  (13 688.1–18 827.1) |
|  | **GMFR** | Day 1 to Day 30  Day 1 to Month 7  Month 6 to Month 7 | 979  810  834 | 5.4  5.1  1.6 | (4.9–5.9)  (4.6–5.7)  (1.5–1.8) | 326  273  278 | 1.0  6.6  6.5 | (0.8–1.1)  (5.5–8.0)  (5.5–7.6) |
|  | **% ≥4-fold rise** | Day 1 to Day 30  Day 1 to Month 7  Month 6 to Month 7 | 979  810  834 | 52.9% (518/979)  53.3% (432/810)  15.8% (132/834) | (49.7–56.1)  (49.8–56.8)  (13.4–18.5) | 326  273  278 | 3.1% (10/326)  60.8% (166/273)  62.6% (174/278) | (1.5–5.6)  (54.7–66.6)  (56.6–68.3) |

Day 1 is pre-vaccination with PCV, Day 30 is 30 days following vaccination with PCV, Month 6 is 6 months following vaccination with PCV and pre-vaccination with PPSV23, and Month 7 is 30 days following vaccination with PPSV23.

Abbreviations: CI, confidence interval; GMFR, geometric mean fold rise; GMT, geometric mean titer (1/dil); OPA, opsonophagocytic activity; PCV13, 13-valent pneumococcal conjugate vaccine; V114, 15-valent pneumococcal conjugate vaccine.

^a^For the continuous endpoints, the within-group 95% Cls are obtained by exponentiating the Cls of the mean of the natural log values based on the t-distribution. For the dichotomous endpoints, the within-group 95% Cls are based on the exact binomial method proposed by Clopper and Pearson.^6^

Supplementary Table 6. Serotype-specific OPA GMTs 30 Days After Vaccination With V114 or PCV13 by Age Group.

| **13 shared serotypes** | **Age group, years** | **V114** | | | **PCV13** | | |
| --- | --- | --- | --- | --- | --- | --- | --- |
|  |  | **n** | **Observed GMT** | **(95% CI)^a^** | **n** | **Observed GMT** | **(95% CI)^a^** |
| **1** | 18–29 | 290 | 337.2 | (288.5–394.0) | 91 | 240.6 | (163.0–355.1) |
|  | 30–39 | 318 | 255.6 | (213.2–306.4) | 102 | 252.7 | (179.3–356.0) |
|  | 40–49 | 411 | 237.7 | (202.1–279.6) | 148 | 296.2 | (220.5–397.9) |
| **3** | 18–29 | 288 | 184.1 | (161.7–209.5) | 90 | 144.0 | (110.1–188.3) |
|  | 30–39 | 316 | 182.5 | (157.4–211.6) | 102 | 160.6 | (123.7–208.3) |
|  | 40–49 | 400 | 226.3 | (200.3–255.6) | 148 | 148.1 | (118.2–185.7) |
| **4** | 18–29 | 289 | 1563.8 | (1364.8–1791.9) | 92 | 3133.5 | (2502.6–3923.4) |
|  | 30–39 | 317 | 1384.2 | (1221.7–1568.2) | 103 | 2661.0 | (2123.8–3334.0) |
|  | 40–49 | 410 | 1343.6 | (1168.2–1545.4) | 147 | 2227.7 | (1835.4–2703.9) |
| **5** | 18–29 | 289 | 692.4 | (591.9–809.9) | 92 | 803.7 | (599.0–1078.3) |
|  | 30–39 | 318 | 543.5 | (452.9–652.3) | 103 | 734.3 | (545.2–988.9) |
|  | 40–49 | 411 | 504.1 | (429.9–591.2) | 148 | 687.2 | (507.9–929.7) |
| **6A** | 18–29 | 284 | 21 578.1 | (18 857.0–24 691.8) | 90 | 16 905.6 | (13 308.4–21 475.2) |
|  | 30–39 | 314 | 13 586.0 | (11 755.1–15 701.9) | 100 | 11 059.1 | (8386.1–14 584.1) |
|  | 40–49 | 408 | 8712.4 | (7687.5–9874.0) | 145 | 8899.7 | (6989.6–11 331.9) |
| **6B** | 18–29 | 288 | 14 439.8 | (12 872.5–16 197.9) | 92 | 11 108.2 | (8572.5–14 394.1) |
|  | 30–39 | 315 | 11 639.5 | (10 460.3–12 951.6) | 103 | 7507.1 | (5756.4–9790.1) |
|  | 40–49 | 411 | 7467.2 | (6634.1–8405.0) | 147 | 4985.2 | (3938.5–6310.2) |
| **7F** | 18–29 | 290 | 6732.6 | (6019.1–7530.7) | 91 | 8969.8 | (7342.2–10 958.1) |
|  | 30–39 | 318 | 5600.4 | (5037.7–6225.9) | 103 | 8017.1 | (6459.7–9950.0) |
|  | 40–49 | 411 | 5264.9 | (4750.4–5835.1) | 148 | 6590.9 | (5522.9–7865.5) |
| **9V** | 18–29 | 289 | 4160.0 | (3727.1–4643.1) | 92 | 5261.7 | (4411.0–6276.4) |
|  | 30–39 | 317 | 3387.4 | (3019.1–3800.5) | 103 | 3554.0 | (2862.6–4412.4) |
|  | 40–49 | 409 | 2860.8 | (2542.9–3218.4) | 148 | 3628.0 | (3020.6–4357.6) |
| **14** | 18–29 | 288 | 7896.1 | (6955.3–8964.1) | 92 | 9848.2 | (7650.3–12 677.5) |
|  | 30–39 | 317 | 5312.5 | (4616.3–6113.7) | 103 | 5734.3 | (4683.5–7020.9) |
|  | 40–49 | 411 | 3869.6 | (3442.8–4349.2) | 148 | 4359.1 | (3675.9–5169.3) |
| **18C** | 18–29 | 287 | 7347.7 | (6596.3–8184.8) | 92 | 3364.1 | (2691.9–4204.0) |
|  | 30–39 | 316 | 5537.4 | (4861.9–6306.8) | 103 | 2730.7 | (2206.8–3379.0) |
|  | 40–49 | 411 | 4900.3 | (4383.8–5477.6) | 148 | 3130.4 | (2527.8–3876.8) |
| **19A** | 18–29 | 289 | 6785.9 | (6024.8–7643.2) | 92 | 7159.9 | (5790.2–8853.5) |
|  | 30–39 | 318 | 5850.6 | (5211.5–6568.2) | 103 | 5959.3 | (4993.8–7111.5) |
|  | 40–49 | 408 | 4255.4 | (3805.4–4758.7) | 148 | 5170.4 | (4195.6–6371.7) |
| **19F** | 18–29 | 290 | 4416.4 | (3935.1–4956.7) | 92 | 4303.0 | (3525.4–5252.2) |
|  | 30–39 | 318 | 3440.0 | (3097.0–3821.0) | 103 | 3489.9 | (2887.3–4218.2) |
|  | 40–49 | 410 | 2534.7 | (2285.3–2811.2) | 148 | 2639.9 | (2257.9–3086.6) |
| **23F** | 18–29 | 288 | 7258.1 | (6340.6–8308.5) | 92 | 7162.2 | (5542.6–9254.9) |
|  | 30–39 | 317 | 4997.1 | (4364.5–5721.3) | 101 | 3671.4 | (2836.0–4752.9) |
|  | 40–49 | 411 | 3579.5 | (3108.5–4122.0) | 147 | 2758.1 | (2154.7–3530.4) |
| **2 serotypes unique to V114** | | | | | | | |
| **22F** | 18–29 | 288 | 4616.5 | (4056.3–5254.0) | 87 | 416.9 | (249.5–696.6) |
|  | 30–39 | 317 | 3947.7 | (3469.3–4492.2) | 96 | 345.1 | (212.6–560.3) |
|  | 40–49 | 400 | 3479.6 | (3072.8–3940.2) | 137 | 206.6 | (133.5–319.5) |
| **33F** | 18–29 | 287 | 13 835.1 | (12 085.6–15 838.0) | 92 | 3418.2 | (2605.7–4484.0) |
|  | 30–39 | 317 | 12 801.7 | (11 294.2–14 510.5) | 103 | 1888.3 | (1300.4–2741.9) |
|  | 40–49 | 410 | 9558.0 | (8543.7–10 692.6) | 143 | 1811.3 | (1392.9–2355.2) |

Abbreviations: CI, confidence interval; GMT, geometric mean titer (1/dil); OPA, opsonophagocytic activity; PCV13, 13-valent pneumococcal conjugate vaccine; V114, 15-valent pneumococcal conjugate vaccine.
^a^The within-group 95% Cls are obtained by exponentiating the Cls of the mean of the natural log values based on the t-distribution.

Supplementary Table 7. Summary of IgG Antibody Responses.

|  | **Endpoint** | **Timepoint** | **V114 (N = 1133)** | | | **PCV13 (N = 379)** | | |
| --- | --- | --- | --- | --- | --- | --- | --- | --- |
| **13 shared serotypes** | | | **n** | **Observed response** | **(95% CI)^a^** | **n** | **Observed response** | **(95% CI)^a^** |
| **1** | **GMC** | Day 1  Day 30  Month 6  Month 7 | 1105  1020  947  844 | 0.29  3.56  1.45  2.91 | (0.27–0.31)  (3.29–3.85)  (1.34–1.57)  (2.71–3.12) | 370  343  316  283 | 0.28  4.59  2.16  3.42 | (0.25–0.32)  (4.00–5.26)  (1.86–2.51)  (3.02–3.87) |
|  | **GMFR** | Day 1 to Day 30  Day 1 to Month 7  Month 6 to Month 7 | 1007  833  843 | 12.2  10.1  2.1 | (11.3–13.2)  (9.4–10.8)  (2.0–2.2) | 338  280  282 | 15.7  11.4  1.5 | (13.9–17.8)  (10.1–12.8)  (1.4–1.7) |
|  | **% ≥4-fold rise** | Day 1 to Day 30  Day 1 to Month 7  Month 6 to Month 7 | 1007  833  843 | 81.2% (818/1007)  81.4% (678/833)  16.4% (138/843) | (78.7–83 .6)  (78.6–84.0)  (13.9–19.0) | 338  280  282 | 86.4% (292/338)  83.6% (234/280)  8.2% (23/282) | (82.3–89.9)  (78.7–87.7)  (5.2–12.0) |
| **3** | **GMC** | Day 1  Day 30  Month 6  Month 7 | 1105  1017  945  844 | 0.18  0.77  0.41  0.66 | (0.16–0.19)  (0.72–0.83)  (0.38–0.44)  (0.62–0.71) | 370  343  316  283 | 0.16  0.63  0.35  0.68 | (0.14–0.18)  (0.57–0.71)  (0.31–0.39)  (0.61–0.76) |
|  | **GMFR** | Day 1 to Day 30  Day 1 to Month 7  Month 6 to Month 7 | 1004  833  842 | 4.1  3.5  1.7 | (3.8–4.3)  (3.3–3.7)  (1.6–1.7) | 338  280  282 | 3.6  3.8  2.0 | (3.2–4.0)  (3 .4–4.2)  (1.8–2.1) |
|  | **% ≥4-fold rise** | Day 1 to Day 30  Day 1 to Month 7  Month 6 to Month 7 | 1004  833  842 | 44.8% (450/1004)  40.7% (339/833)  6.3% (53/842) | (41.7–48.0)  (37.3–44.1)  (4.8–8.2) | 338  280  282 | 41.1% (139/338)  46.1% (129/280)  13.8% (39/282) | (35.8–46.6)  (40.1–52.1)  (10.0–1 8.4) |
| **4** | **GMC** | Day 1  Day 30  Month 6  Month 7 | 1103  1015  945  843 | 0.18  1.53  0.71  1.33 | (0.17–0.20)  (1.40–1.66)  (0.66–0.77)  (1.23–1.44) | 370  343  316  281 | 0.16  2.71  1.04  1.77 | (0.14–0.18)  (2.34–3.13)  (0.90–1.21)  (1.55–2.02) |
|  | **GMFR** | Day 1 to Day 30  Day 1 to Month 7  Month 6 to Month 7 | 1001  830  840 | 8.0  7.1  2.0 | (7.4–8.6)  (6.7–7.6)  (1.9–2.1) | 338  278  280 | 16.2  10.7  1.6 | (14.3–18.4)  (9.5–12.0)  (1.5–1.7) |
|  | **% ≥4-fold rise** | Day 1 to Day 30  Day 1 to Month 7  Month 6 to Month 7 | 1001  830  840 | 70.1% (702/1001)  71.2% (591/830)  15.2% (128/840) | (67.2–73.0)  (68.0–74.3)  (12.9–17.8) | 338  278  280 | 86.1% (291/338)  82.0% (228/278)  7.5% (21/280) | (81.9–89.6)  (77.0–86.3)  (4.7–11.2) |
| **5** | **GMC** | Day 1  Day 30  Month 6  Month 7 | 1105  1020  947  844 | 0.80  3.43  1.92  3.45 | (0.77–0.84)  (3.14–3 .75)  (1.77–2.07)  (3.17–3.75) | 370  342  316  283 | 0.84  4.48  2.52  3.92 | (0.76–0.92)  (3.77–5.32)  (2.16–2.93)  (3.37–4.56) |
|  | **GMFR** | Day 1 to Day 30  Day 1 to Month 7  Month 6 to Month 7 | 1007  833  843 | 4.3  4.3  1.9 | (4.0–4.7)  (4.0–4.6)  (1.8–1.9) | 337  280  282 | 5.2  4.6  1.5 | (4.5–6.0)  (4.1–5.2)  (1.4–1.6) |
|  | **% ≥4-fold rise** | Day 1 to Day 30  Day 1 to Month 7  Month 6 to Month 7 | 1007  833  843 | 44.1% (444/1007)  48.7% (406/833)  1 2.1% (102/843) | (41.0–47.2)  (45.3–52.2)  (10.0–14.5) | 337  280  282 | 50.4% (170/337)  51.8% (145/280)  5.7% (16/282) | (45.0–55.9)  (45.8–57.8)  (3.3–9.1) |
| **6A** | **GMC** | Day 1  Day 30  Month 6  Month 7 | 1105  1020  947  843 | 0.32  11.84  4.33  4.25 | (0.30–0.35)  (10.71–13.10)  (3.93–4.78)  (3.84–4.69) | 370  343  316  283 | 0.32  10.87  4.44  4.88 | (0.28–0.37)  (9.06–1 3 .05)  (3.71–5.32)  (4.08–5.85) |
|  | **GMFR** | Day 1 to Day 30  Day 1 to Month 7  Month 6 to Month 7 | 1007  832  842 | 35.5  12.3  1.0 | (32.5–38.8)  (11.3–13.4)  (1.0–1.0) | 338  280  282 | 31.3  13.9  1.0 | (26.7–36.6)  (12.0–1 6.1)  (1.0–1.1) |
|  | **% ≥4-fold rise** | Day 1 to Day 30  Day 1 to Month 7  Month 6 to Month 7 | 1007  832  842 | 93.3% (940/1007)  81.6% (679/832)  0.6% (5/842) | (91.6–94.8)  (78.8–84.2)  (0.2–1.4) | 338  280  282 | 88.2% (298/338)  81.8% (229/280)  1.1% (3/282) | (84.2–91.4)  (76.8–86.1)  (0.2–3.1) |
| **6B** | **GMC** | Day 1  Day 30  Month 6  Month 7 | 1104  1020  947  844 | 0.44  17.90  6.41  6.79 | (0.40–0.47)  (16.21–19.76)  (5.81–7.08)  (6.15–7.49) | 370  342  316  283 | 0.40  11.36  4.69  6.04 | (0.35–0.45)  (9.45–13.66)  (3.90–5.65)  (5.08–7.19) |
|  | **GMFR** | Day 1 to Day 30  Day 1 to Month 7  Month 6 to Month 7 | 1006  832  843 | 40.5  15.3  1.1 | (37.1–44.2)  (14.1–16.6)  (1.0–1.1) | 337  280  282 | 28.0  14.9  1.2 | (24.2–32.4)  (13.0–17.0)  (1.2–1.3) |
|  | **% ≥4-fold rise** | Day 1 to Day 30  Day 1 to Month 7  Month 6 to Month 7 | 1006  832  843 | 93.4% (940/1006)  86.4% (719/832)  0.7% (6/843) | (91.7–94.9)  (83.9–88.7)  (0.3–1.5) | 337  280  282 | 90.5% (305/337)  85.4% (239/280)  2.8% (8/282) | (86.9–93.4)  (80.7–89.3)  (1.2–5.5) |
| **7F** | **GMC** | Day 1  Day 30  Month 6  Month 7 | 1105  1020  947  844 | 0.42  5.18  2.22  3.64 | (0.39–0.45)  (4.77–5.62)  (2.04–2.41)  (3.36–3.94) | 370  343  316  283 | 0.40  6.88  2.87  4.07 | (0.36–0.45)  (5.95–7.95)  (2.47–3.34)  (3.54–4.68) |
|  | **GMFR** | Day 1 to Day 30  Day 1 to Month 7  Month 6 to Month 7 | 1007  833  843 | 12.2  8.7  1.7 | (11.3–13 .2)  (8.1 –9.3)  (1.6–1.8) | 338  280  282 | 16.9  10.1  1.4 | (14.8–19.3)  (9.0–11.3)  (1.3–1.5) |
|  | **% ≥4-fold rise** | Day 1 to Day 30  Day 1 to Month 7  Month 6 to Month 7 | 1007  833  843 | 79.3% (799/1007)  77.4% (645/833)  8.4% (71/843) | (76.7–81.8)  (74.4–80.2)  (6.6–10.5) | 338  280  282 | 86.4% (292/338)  84.3% (236/280)  4.3% (12/282) | (82.3–89.9)  (79.5–88.3)  (2.2–7.3) |
| **9V** | **GMC** | Day 1  Day 30  Month 6  Month 7 | 1105  1019  944  844 | 0.41  4.44  2.13  3.18 | (0.38–0.44)  (4.11–4.80)  (1.97–2.30)  (2.96–3 .43) | 370  343  316  283 | 0.40  5.31  2.49  3.61 | (0.36–0.45)  (4.62–6. 1 0)  (2.17–2.86)  (3.17–4. 12) |
|  | **GMFR** | Day 1 to Day 30  Day 1 to Month 7  Month 6 to Month 7 | 1006  833  841 | 10.6  7.6  1.5 | (9.8–11.4)  (7.1–8.1)  (1.5–1.6) | 338  280  282 | 12.8  8.5  1.4 | (11.3–14.5)  (7.6–9.5)  (1.3–1.5) |
|  | **% ≥4-fold rise** | Day 1 to Day 30  Day 1 to Month 7  Month 6 to Month 7 | 1006  833  841 | 76.5% (770/1006)  71.8% (598/833)  6.3% (53/841) | (73.8–79. 1)  (68.6–74.8)  (4.8–8.2) | 338  280  282 | 84.0% (284/338)  77.1% (216/280)  3.9% (11/282) | (79.7–87.8)  (71.8–8 1.9)  (2.0–6.9) |
| **14** | **GMC** | Day 1  Day 30  Month 6  Month 7 | 1105  1020  946  844 | 1.38  15.91  8.83  14.28 | (1.26–1.52)  (14.46–17.51)  (8.04–9.69)  (13.13–15.53) | 370  343  316  283 | 1.43  17.35  10.39  14.59 | (1.20–1.69)  (14.93–20.16)  (8.96–12.06)  (12.64–16.85) |
|  | **GMFR** | Day 1 to Day 30  Day 1 to Month 7  Month 6 to Month 7 | 1007  833  843 | 11.3  10.3  1.7 | (10.3–12.5)  (9.4–11.3)  (1.6–1.8) | 338  280  282 | 11.5  10.0  1.4 | (9.7–13.7)  (8.5–11.8)  (1.3–1.5) |
|  | **% ≥4-fold rise** | Day 1 to Day 30  Day 1 to Month 7  Month 6 to Month 7 | 1007  833  843 | 70.8% (713/1007)  76.7% (639/833)  9.8% (83/843) | (67.9–73.6)  (73.7–79.5)  (7.9–12.1) | 338  280  282 | 68.6% (232/338)  70.7% (198/280)  3.9% (11/282) | (63.4–73.6)  (65.0–76.0)  (2.0–6.9) |
| **18C** | **GMC** | Day 1  Day 30  Month 6  Month 7 | 1105  1020  947  844 | 0.48  14.57  5.36  5.50 | (0.44–0.51)  (13.42–15.81)  (4.93–5.82)  (5.07–5.98) | 370  343  316  283 | 0.46  9.32  3.80  4.24 | (0.40–0.52)  (8.06–1 0.79)  (3.30–4.38)  (3.71 –4.84) |
|  | **GMFR** | Day 1 to Day 30  Day 1 to Month 7  Month 6 to Month 7 | 1007  833  843 | 30.5  11.5  1.1 | (28.0–33.1)  (10.7–12.4)  (1.0–1.1) | 338  280  282 | 20.0  9.3  1.1 | (17.2–23.2)  (8.2–1 0.4)  (1.1–1.2) |
|  | **% ≥4-fold rise** | Day 1 to Day 30  Day 1 to Month 7  Month 6 to Month 7 | 1007  833  843 | 91.1% (917/1007)  82.7% (689/833)  0.6% (5/843) | (89.1–92.8)  (80.0–85.2)  (0.2–1.4) | 338  280  282 | 87.3% (295/338)  80.4% (225/280)  0.7% (2/282) | (83.2–90.6)  (75.2–84.8)  (0.1–2.5) |
| **19A** | **GMC** | Day 1  Day 30  Month 6  Month 7 | 1105  1020  946  844 | 1.73  19.41  8.10  11.26 | (1.63–1.84)  (18.00–20.93)  (7.55–8.69)  (10.47–12.11) | 370  343  316  283 | 1.53  21.79  8.77  12.04 | (1.37–1.71)  (18.85–25.19)  (7.63–10.09)  (10.57–13.72) |
|  | **GMFR** | Day 1 to Day 30  Day 1 to Month 7  Month 6 to Month 7 | 1007  833  842 | 11.1  6.4  1.4 | (10.2–12.0)  (6.0–6.9)  (1.3–1.4) | 338  280  282 | 14.0  7.5  1.4 | (12.2–16.0)  (6.7–8.5)  (1.3–1.4) |
|  | **% ≥4-fold rise** | Day 1 to Day 30  Day 1 to Month 7  Month 6 to Month 7 | 1007  833  842 | 75.3% (758/1007)  65.8% (548/833)  3.4% (29/842) | (72.5–77.9)  (62.5–69.0)  (2.3–4.9) | 338  280  282 | 82.0% (277/338)  74.6% (209/280)  4.3% (12/282) | (77.4–85.9)  (69.1–79.6)  (2.2–7.3) |
| **19F** | **GMC** | Day 1  Day 30  Month 6  Month 7 | 1105  1018  947  844 | 0.96  13.98  5.59  9.07 | (0.89–1 .04)  (12.90–15.14)  (5.1 7–6.03)  (8.45–9.74) | 370  343  316  283 | 0.85  13.35  5.32  8.81 | (0.75–0.97)  (11.52–15.47)  (4.59–6.16)  (7.74–10.02) |
|  | **GMFR** | Day 1 to Day 30  Day 1 to Month 7  Month 6 to Month 7 | 1005  833  843 | 14.4  9.0  1.6 | (13.2–15.7)  (8.3–9.7)  (1.6–1.7) | 338  280  282 | 15.3  9.8  1.6 | (13.1–17.7)  (8.6–11.2)  (1.5–1.7) |
|  | **% ≥4-fold rise** | Day 1 to Day 30  Day 1 to Month 7  Month 6 to Month 7 | 1005  833  843 | 80.0% (804/1005)  74.5% (621/833)  8.1% (68/843) | (77.4–82.4)  (71.4–77.5)  (6.3–10.1) | 338  280  282 | 80.8% (273/338)  78.9% (221/280)  6.4% (18/282) | (76.2–84.8)  (73.7–83.6)  (3.8–9.9) |
| **23F** | **GMC** | Day 1  Day 30  Month 6  Month 7 | 1105  1019  946  844 | 0.49  13.57  5.00  5.42 | (0.46–0.53)  (12.44–14.80)  (4.59–5.44)  (4.98–5.89) | 370  343  316  282 | 0.49  10.98  4.27  4.96 | (0.43–0.55)  (9.34–12.91)  (3.62–5.04)  (4.25–5.81) |
|  | **GMFR** | Day 1 to Day 30  Day 1 to Month 7  Month 6 to Month 7 | 1006  833  842 | 26.5  10.8  1.1 | (24.2–29.0)  (9.9–11.7)  (1.1–1.2) | 338  279  281 | 22.0  10.3  1.2 | (19.0–25.5)  (9.0–11.7)  (1.1–1.2) |
|  | **% ≥4-fold rise** | Day 1 to Day 30  Day 1 to Month 7  Month 6 to Month 7 | 1006  833  842 | 89.2% (897/1006)  77.8% (648/833)  0.5% (4/842) | (87.1–91.0)  (74.8–80.6)  (0.1–1.2) | 338  279  281 | 87.6% (296/338)  78.1% (218/279)  1.1% (3/281) | (83.6–90.9)  (72.8–82.8)  (0.2–3.1) |
| **2 serotypes unique to V114** | | |  |  |  |  |  |  |
| **22F** | **GMC** | Day 1  Day 30  Month 6  Month 7 | 1105  1020  947  844 | 0.52  6.22  2.74  4.85 | (0.48–0.57)  (5.74–6.74)  (2.54–2.96)  (4.50–5.23) | 370  342  316  283 | 0.49  0.52  0.51  4.76 | (0.42–0.56)  (0.45–0.60)  (0.44–0.59)  (4.01 –5.65) |
|  | **GMFR** | Day 1 to Day 30  Day 1 to Month 7  Month 6 to Month 7 | 1007  833  843 | 11.4  8.9  1.8 | (10.4–12.6)  (8.1–9.7)  (1.7–1.9) | 337  280  282 | 1.0  8.9  8.8 | (1.0–1.1)  (7.5–10.7)  (7.4–10.4) |
|  | **% ≥4-fold rise** | Day 1 to Day 30  Day 1 to Month 7  Month 6 to Month 7 | 1007  833  843 | 73.1% (736/1007)  68.8% (573/833)  10.2% (86/843) | (70.2–75.8)  (65.5–71.9)  (8.2–12.4) | 337  280  282 | 1.5% (5/337)  65.7% (184/280)  64.9% (183/282) | (0.5–3.4)  (59.8–71.3)  (59.0–70.5) |
| **33F** | **GMC** | Day 1  Day 30  Month 6  Month 7 | 1105  1020  947  844 | 0.95  7.79  3.80  5.98 | (0.88–1.02)  (7.16–8.48)  (3.49–4.13)  (5.50–6.50) | 370  342  316  283 | 0.89  0.88  0.86  8.66 | (0.78–1.01)  (0.78–1.00)  (0.76–0.99)  (7.31–10.26) |
|  | **GMFR** | Day 1 to Day 30  Day 1 to Month 7  Month 6 to Month 7 | 1007  833  843 | 8.3  6.4  1.6 | (7.7–8.9)  (6.0–6.9)  (1.6–1.7) | 337  280  282 | 1.0  9.6  9.9 | (1.0–1.0)  (8.4–11.1)  (8.6–11.3) |
|  | **% ≥4-fold rise** | Day 1 to Day 30  Day 1 to Month 7  Month 6 to Month 7 | 1007  833  843 | 70.0% (705/1007)  68.2% (568/833)  7.5% (63/843) | (67.1–72.8)  (64.9–71.3)  (5.8–9.5) | 337  280  282 | 0.3% (1/337)  75.7% (212/280)  75.9% (214/282) | (0.0–1.6)  (70.3–80.6)  (70.5–80.8) |

Day 1 is pre-vaccination with PCV, Day 30 is 30 days following vaccination with PCV, Month 6 is 6 months following vaccination with PCV and pre-vaccination with PPSV23, and Month 7 is 30 days following vaccination with PPSV23.

Abbreviations: CI, confidence interval; GMC, geometric mean concentration; GMFR, geometric mean fold rise; IgG, immunoglobulin G; PCV13, 13-valent pneumococcal conjugate vaccine; V114, 15-valent pneumococcal conjugate vaccine.

^a^For the continuous endpoints, the within-group 95% Cls are obtained by exponentiating the Cls of the mean of the natural log values based on the t-distribution. For the dichotomous endpoints, the within-group 95% Cls are based on the exact binomial method proposed by Clopper and Pearson.^6^

# Supplementary references

1. Demirdal T, Sen P, Emir B. Predictors of mortality in invasive pneumococcal disease: a meta-analysis. *Expert Rev Anti Infect Ther*. Jul 2021;19(7):927-944. doi:10.1080/14787210.2021.1858799

2. Grau I, Ardanuy C, Calatayud L, Schulze MH, Linares J, Pallares R. Smoking and alcohol abuse are the most preventable risk factors for invasive pneumonia and other pneumococcal infections. *Int J Infect Dis*. Aug 2014;25:59-64. doi:10.1016/j.ijid.2013.12.013

3. Mooney JD, Imarhiagbe M, Ling J. Should UK Pneumococcal Vaccine Eligibility Criteria Include Alcohol Dependency in Areas with High Alcohol-Related Mortality? *Vaccines (Basel)*. May 2 2018;6(2)doi:10.3390/vaccines6020025

4. Samuelson DR, Siggins RW, Ruan S, et al. Alcohol consumption increases susceptibility to pneumococcal pneumonia in a humanized murine HIV model mediated by intestinal dysbiosis. *Alcohol*. Nov 2019;80:33-43. doi:10.1016/j.alcohol.2018.08.012

5. Rumpf HJ, Hapke U, Meyer C, John U. Screening for alcohol use disorders and at-risk drinking in the general population: psychometric performance of three questionnaires. *Alcohol Alcohol*. May-Jun 2002;37(3):261-8. doi:10.1093/alcalc/37.3.261

6. Clopper CJ, Pearson ES. The use of confidence or fiducial limits illustrated in the case of the binomial. *Biometrika*. 1934;26:404–413.
