# Supplementary material for: Immunogenicity, Safety, and Tolerability of V114, a 15-Valent Pneumococcal Conjugate Vaccine, in Immunocompetent Adults Aged 18–49 Years With or Without Risk Factors for Pneumococcal Disease: A Randomized Phase 3 Trial (PNEU-DAY)
Source: Open Forum Infect Dis. 2021 Dec 18;9(3):ofab605. doi: 10.1093/ofid/ofab605 (PMC8826015; doi:10.1093/ofid/ofab605)
Supplement: ofab605_suppl_Supplementary_Protocol [file ofab605_suppl_supplementary_protocol.pdf]

Merck & Co., Inc. policy on posting of redacted study protocols on journal websites is described in Guidelines for Publication of Clinical Trials in the Scientific Literature on the www.merck.com website.

For publicly posted protocols, the Company redacts content that contains proprietary and private information.

This report may include approved and non-approved uses, formulations, or treatment regimens. The results reported may not reflect the overall profile of a product. Before prescribing any product mentioned in this report, healthcare professionals should consult local prescribing information for the product approved in their country.

Copyright © 2018 Merck Sharp & Dohme Corp., a subsidiary of Merck & Co., Inc. All Rights Reserved. Not for regulatory or commercial use.

Title Page

THIS PROTOCOL AMENDMENT AND ALL OF THE INFORMATION RELATING TO IT ARE CONFIDENTIAL AND PROPRIETARY PROPERTY OF MERCK SHARP & DOHME CORP., A SUBSIDIARY OF MERCK & CO., INC., NJ, U.S.A. (MSD).

Protocol Title: A Phase 3, Multicenter, Randomized, Double-blind, Active Comparator-controlled Study to Evaluate the Safety, Tolerability, and Immunogenicity of V114 Followed by Administration of PNEUMOVAX™23 Six Months Later in Immunocompetent Adults Between 18 and 49 Years of Age at Increased Risk for Pneumococcal Disease (PNEU – DAY).

This protocol amendment is applicable only to Russia.

Protocol Number: 017-03

Compound Number: V114

Sponsor Name:

Merck Sharp & Dohme Corp., a subsidiary of Merck & Co., Inc.

(hereafter referred to as the Sponsor or MSD)

Legal Registered Address:

One Merck Drive

P.O. Box 100

Whitehouse Station, New Jersey, 08889-0100, U.S.A.

Regulatory Agency Identifying Number(s):

IND	14977
EudraCT	2017-004915-38

Approval Date: 28 January 2019

Sponsor Signatory

Typed Name: Date
Title:

Protocol-specific Sponsor contact information can be found in the Investigator Study File Binder (or equivalent).

Investigator Signatory

I agree to conduct this clinical study in accordance with the design outlined in this protocol and to abide by all provisions of this protocol.

Typed Name: Date
Title:

DOCUMENT HISTORY

Document	Date of Issue	Overall Rationale
Amendment 3	**28-JAN-2019**	The primary purpose of this amendment is to include country-specific requirements for the Ministry of Healthcare (MoH) of the Russian Federation.
Amendment 2	31-AUG-2018	The primary purpose of this amendment is to include a spirometry assessment at Visit 1 (Screening) for those otherwise eligible individuals who have a clinical history of asthma or chronic obstructive pulmonary disease (COPD) and who do not have spirometry results from within the previous 5 years. This change ensures that those individuals with a clinical diagnosis of asthma or COPD who do not have spirometry results available within the previous 5 years meet the documentation requirements for inclusion in the study.
Amendment 1	01-JUN-2018	The primary purpose of this amendment is to remove the collection of medical device incidents from the protocol. The implementation of the FDA guidance for reporting events related to combination products was delayed until January 2020. Therefore, the protocol is not within the scope of medical device reporting requirements.
Original Protocol	19-APR-2018	Not applicable.

Confidential
07Y9YV 054WRH

PROTOCOL AMENDMENT SUMMARY OF CHANGES

Amendment: 03

Overall Rationale for the Amendments:

The primary purpose of this amendment is to include country-specific requirements for the Ministry of Healthcare (MoH) of the Russian Federation.

Summary of Changes Table:

**Section # and Name**	**Description of Change**	**Brief Rationale**
2.2.2 Preclinical and Clinical Studies	Added a cross-reference to Appendix 10.7.1	
Section 4.2 Scientific Rationale for Study Design	Added a cross-reference to Appendix 10.7.1	
10.7 Appendix 7: Country-specific Requirements	• Expanded discussion of clinical study experience with V114<br>• Added rationale for studying V114 as part of a sequential vaccination regimen in immunocompetent adults at increased risk for pneumococcal disease	Country-specific Appendix was added in response to MoH of the Russian Federation

Confidential

07Y9YV 054WRH

Table of Contents

DOCUMENT HISTORY 3
PROTOCOL AMENDMENT SUMMARY OF CHANGES 4
1 PROTOCOL SUMMARY 12
1.1 Synopsis 12
1.2 Schema 17
1.3 Schedule of Activities (SoA) 18
1.3.1 Schedule of Activities for the Main Study 18
1.3.2 Schedule of Activities for Native Americans Enrolled through the Johns Hopkins Center for American Indian Health (CAIH) Participating in the Substudy 25
2 INTRODUCTION 32
2.1 Study Rationale 32
2.2 Background 33
2.2.1 V114 and Pneumococcal Disease 33
2.2.2 Preclinical and Clinical Studies 34
2.2.3 Information on Other Study-related Therapy 34
2.2.3.1 Prevnar 13™ 34
2.2.3.2 PNEUMOVAX™23 35
2.3 Benefit/Risk Assessment 35
3 HYPOTHESIS, OBJECTIVES, AND ENDPOINTS 36
4 STUDY DESIGN 38
4.1 Overall Design 38
4.1.1 Main Study 38
4.1.2 Substudy 39
4.2 Scientific Rationale for Study Design 40
4.2.1 Rationale for Endpoints 41
4.2.1.1 Immunogenicity Endpoints 41
4.2.1.2 Safety Endpoints 42
4.2.1.3 Future Biomedical Research 42
4.2.2 Rationale for the Use of Comparator/Placebo 42
4.3 Justification for Dose 43
4.4 Beginning and End of Study Definition 43
4.4.1 Clinical Criteria for Early Study Termination 43
5 STUDY POPULATION 43
5.1 Inclusion Criteria 43
5.2 Exclusion Criteria 46

5.3 Lifestyle Considerations 49
5.4 Screen Failures 49
5.5 Participant Replacement Strategy 49
6 STUDY INTERVENTION 49
6.1 Study Intervention(s) Administered 49
6.2 Preparation/Handling/Storage/Accountability 51
6.2.1 Dose Preparation 51
6.2.2 Handling, Storage, and Accountability 51
6.3 Measures to Minimize Bias: Randomization and Blinding 51
6.3.1 Intervention Assignment 51
6.3.2 Stratification 52
6.3.3 Blinding 53
6.4 Study Intervention Compliance 54
6.5 Concomitant Therapy 54
6.5.1 Rescue Medications and Supportive Care 54
6.6 Dose Modification (Escalation/Titration/Other) 55
6.7 Intervention After the End of the Study 55
6.8 Clinical Supplies Disclosure 55
7 DISCONTINUATION OF STUDY INTERVENTION AND PARTICIPANT WITHDRAWAL 55
7.1 Discontinuation of Study Intervention 55
7.2 Participant Withdrawal From the Study 56
7.3 Lost to Follow-up 56
8 STUDY ASSESSMENTS AND PROCEDURES 57
8.1 Administrative and General Procedures 58
8.1.1 Informed Consent 58
8.1.1.1 General Informed Consent 59
8.1.1.2 Consent and Collection of Specimens for Future Biomedical Research 59
8.1.2 Inclusion/Exclusion Criteria 59
8.1.3 Participant Identification Card 59
8.1.4 Medical History 60
8.1.5 Prior and Concomitant Medications Review 60
8.1.5.1 Prior Medications 60
8.1.5.2 Concomitant Medications 60
8.1.6 Assignment of Screening Number 60
8.1.7 Assignment of Treatment/Randomization Number 61
8.1.8 Study Intervention Administration 61

Confidential
07Y9YV 054WRH

8.1.8.1 Timing of Dose Administration 62
8.1.9 Vaccination Report Card 62
8.1.10 6-month Safety Follow-up Questionnaire 63
8.1.11 Discontinuation and Withdrawal 63
8.1.11.1 Withdrawal From Future Biomedical Research 63
8.1.12 Participant Blinding/Unblinding 63
8.1.13 Calibration of Equipment 64
8.2 Immunogenicity Assessments 64
8.2.1 Multiplex Opsonophagocytic Assay (MOPA) 65
8.2.2 Electrochemiluminescence (ECL) 65
8.3 Safety Assessments 66
8.3.1 Physical Examinations 66
8.3.2 Pregnancy Test 66
8.3.3 Body Temperature Measurement 66
8.3.4 Safety Assessments and Use of the Paper VRC/eVRC 66
8.3.5 Clinical Laboratory Assessments 67
8.4 Adverse Events (AEs), Serious Adverse Events (SAEs), and Other Reportable Safety Events 67
8.4.1 Time Period and Frequency for Collecting AE, SAE, and Other Reportable Safety Event Information 68
8.4.2 Method of Detecting AEs, SAEs, and Other Reportable Safety Events 69
8.4.3 Follow-up of AE, SAE, and Other Reportable Safety Event Information 70
8.4.4 Regulatory Reporting Requirements for SAE 70
8.4.5 Pregnancy and Exposure During Breastfeeding 70
8.4.6 Disease-related Events and/or Disease-related Outcomes Not Qualifying as AEs or SAEs 70
8.4.7 Events of Clinical Interest (ECIs) 71
8.5 Treatment of Overdose 71
8.6 Pharmacokinetics 71
8.7 Pharmacodynamics 71
8.8 Future Biomedical Research Sample Collection 71
8.9 Planned Genetic Analysis Sample Collection 71
8.10 Biomarkers 71
8.11 Medical Resource Utilization and Health Economics 72
8.12 Visit Requirements 72
8.12.1 Screening 72
8.12.2 Treatment Period/Vaccination Visit 72
8.12.3 Discontinued Participants Continuing to be Monitored in the Study 72

Confidential

07Y9YV 054WRH

9 STATISTICAL ANALYSIS PLAN 72
9.1 Statistical Analysis Plan Summary....72
9.2 Responsibility for Analyses/In-house Blinding 74
9.3 Hypotheses/Estimation 75
9.4 Analysis Endpoints....75
9.4.1 Immunogenicity Endpoints....75
9.4.2 Safety Endpoints 75
9.5 Analysis Populations....76
9.5.1 Immunogenicity Analysis Populations 76
9.5.2 Safety Analysis Populations 77
9.6 Statistical Methods....77
9.6.1 Statistical Methods for Immunogenicity Analyses 77
9.6.2 Statistical Methods for Safety Analyses 79
9.6.3 Demographic and Baseline Characteristics 81
9.7 Interim Analyses 81
9.8 Multiplicity 81
9.9 Sample Size and Power Calculations 82
9.9.1 Sample Size and Power for Immunogenicity Analyses 82
9.9.2 Sample Size and Power for Safety Analyses 82
9.10 Subgroup Analyses....83
9.11 Compliance (Medication Adherence)....83
9.12 Extent of Exposure....83
10 SUPPORTING DOCUMENTATION AND OPERATIONAL CONSIDERATIONS....84
10.1 Appendix 1: Regulatory, Ethical, and Study Oversight Considerations 84
10.1.1 Code of Conduct for Clinical Trials....84
10.1.2 Financial Disclosure....86
10.1.3 Data Protection....86
10.1.3.1 Confidentiality of Data 86
10.1.3.2 Confidentiality of Participant Records....86
10.1.3.3 Confidentiality of IRB/IEC Information....87
10.1.4 Committees Structure....87
10.1.4.1 External Data Monitoring Committee 87
10.1.4.2 Executive Oversight Committee 87
10.1.4.3 Scientific Advisory Committee....87
10.1.5 Publication Policy 88
10.1.6 Compliance with Study Registration and Results Posting Requirements 88
10.1.7 Compliance with Law, Audit, and Debarment 88

Confidential

07Y9YV 054WRH

10.1.8 Data Quality Assurance 89
10.1.9 Source Documents 90
10.1.10 Study and Site Closure 90
10.2 Appendix 2: Clinical Laboratory Tests 91
10.3 Appendix 3: Adverse Events: Definitions and Procedures for Recording, Evaluating, Follow-up, and Reporting 92
10.3.1 Definition of AE 92
10.3.2 Definition of SAE 93
10.3.3 Additional Events Reported 94
10.3.4 Recording AE and SAE 94
10.3.5 Reporting of AE, SAE, and Other Reportable Safety Events to the Sponsor 99
10.4 Appendix 4: Medical Device Incidents: Definition and Procedures for Recording, Evaluating, Follow-up, and Reporting 101
10.5 Appendix 5: Contraceptive Guidance and Pregnancy Testing 102
10.5.1 Definitions 102
10.5.2 Contraception Requirements 103
10.5.3 Pregnancy Testing 104
10.6 Appendix 6: Collection and Management of Specimens for Future Biomedical Research (Does not apply to CAIH participants) 104
10.7 Appendix 7: Country-specific Requirements 109
10.7.1 Country-specific Request from Ministry of Healthcare of the Russian Federation 109
10.7.1.1 Clinical Experience With V114 109
10.7.1.2 Rationale for Testing V114 in Study Population With a Sequential Vaccination Regimen 110
10.8 Appendix 8: Abbreviations 113
11 REFERENCES 115

LIST OF TABLES

Table 1 Study Interventions 50
Table 2 Approximate Blood Volumes Drawn by Study Visit and by Sample Types for Participants Enrolled at non-CAIH Sites....................58
Table 3 Approximate Blood Volumes Drawn by Study Visit and by Sample Types for Participants Enrolled at CAIH Sites 58
Table 4 Reporting Time Periods and Time Frames for Adverse Events and Other Reportable Safety Events....................69
Table 5 Analysis Strategy for Immunogenicity Variables....................79
Table 6 Analysis Strategy for Safety Parameters Following Each Vaccination 80
Table 7 Within-group 95% CIs for Varying Hypothetical OPA GMTs and Varying Standard Deviations....................82
Table 8 Protocol-required Safety Laboratory Assessments 91
Table 9 Contraceptive Methods 103
Table 10 Completed Clinical Studies in Immunocompetent Volunteers 109
Table 11 V114-006: Safety Profile of V114 in Study Participants With and Without Specific Comorbid Conditions....................110

LIST OF FIGURES

Figure 1 V114-017 Study Design..17

Confidential

1 PROTOCOL SUMMARY

1.1 Synopsis

Protocol Title: A Phase 3, Multicenter, Randomized, Double-blind, Active Comparator-controlled Study to Evaluate the Safety, Tolerability, and Immunogenicity of V114 Followed by Administration of PNEUMOVAX™23 Six Months Later in Immunocompetent Adults Between 18 and 49 Years of Age at Increased Risk for Pneumococcal Disease (PNEU – DAY).

Short Title: Safety and Immunogenicity of V114 in Adults at Risk for Pneumococcal Disease

Acronym: PNEUmococcal Conjugate Vaccine Trials: V114-017 (PNEU – DAY)

Hypotheses, Objectives, and Endpoints:

Objectives and endpoints described below will be evaluated in pneumococcal vaccine-naïve participants 18 to 49 years of age (inclusive) who are at increased risk for pneumococcal disease due to (1) an underlying medical condition, (2) behavioral habits such as smoking, or (3) living in a community/environment with increased risk of disease transmission.

Primary Objectives	Primary Endpoints
- Objective: To evaluate the safety and tolerability of V114 and Prevnar 13™ with respect to the proportion of participants with adverse events (AEs) within each vaccination group separately.	Following vaccination with V114 or Prevnar 13™:<br><br>- Solicited injection-site AEs from Day 1 through Day 5 postvaccination<br><br>- Solicited systemic AEs from Day 1 through Day 14 postvaccination<br><br>- Vaccine-related serious adverse events (SAEs) from Day 1 to Month 6
- Objective: To evaluate the serotype-specific opsonophagocytic activity (OPA) Geometric Mean Titers (GMTs) at 30 days postvaccination (Day 30) with V114 and Prevnar 13™ within each vaccination group separately.	- Serotype-specific OPA responses for the 15 serotypes in V114 at Day 30

Confidential
07Y9YV 054WRH

Secondary Objectives	Secondary Endpoints
- Objective: To evaluate the safety and tolerability of PNEUMOVAX™23 administered 6 months following V114 and following Prevnar 13™ with respect to the proportion of participants with AEs within each vaccination group separately.	Following vaccination with PNEUMOVAX™23:<br><br>- Solicited injection-site AEs from Day 1 through Day 5 postvaccination<br><br>- Solicited systemic AEs from Day 1 through Day 14 postvaccination<br><br>- Vaccine-related SAEs from Month 6 to Month 7
- Objective: To evaluate the serotype-specific Immunoglobulin G (IgG) Geometric Mean Concentrations (GMCs) at 30 days postvaccination (Day 30) with V114 and Prevnar 13™ within each vaccination group separately.	- Serotype-specific IgG responses for the 15 serotypes in V114 at Day 30
- Objective: To evaluate the serotype-specific Geometric Mean Fold Rises (GMFRs) and proportions of participants with a ≥4-fold rise from prevaccination (Day 1) to 30 days postvaccination (Day 30) for both OPA and IgG responses for participants administered V114 and for participants administered Prevnar 13™ within each vaccination group separately.	- Serotype-specific OPA and IgG responses for the 15 serotypes in V114 at Day 1 and Day 30
- Objective: To evaluate the serotype-specific (1) OPA GMTs and IgG GMCs at 30 days postvaccination with PNEUMOVAX™23 (Month 7), (2) GMFRs and proportions of participants with a ≥4-fold rise from prevaccination (Day 1) to 30 days postvaccination with PNEUMOVAX™23 (Month 7) for both OPA and IgG responses, (3) GMFRs and proportions of participants with a ≥4-fold rise from prevaccination with PNEUMOVAX™23 (Month 6) to 30 days postvaccination with PNEUMOVAX™23 (Month 7) for both OPA and IgG responses for participants administered V114 and separately for participants administered Prevnar 13™ 6 months before receipt of PNEUMOVAX™23.	- Serotype-specific OPA and IgG responses for the 15 serotypes in V114 at Day 1, Month 6, and Month 7

Overall Design:

Study Phase	Phase 3
Primary Purpose	Prevention
Indication	Pneumococcal disease
Population	Pneumococcal vaccine-naïve participants 18 to 49 years of age (inclusive) who are at increased risk for pneumococcal disease due to (1) an underlying medical condition, (2) behavioral habits such as smoking, or (3) living in a community/environment with increased risk of disease transmission.
Study Type	Interventional
Intervention Model	Parallel<br>This is a multi-site study.
Type of Control	Active control without placebo
Study Blinding	Double-blind
Masking	Participant<br>Investigator
Estimated Duration of Study	The Sponsor estimates that the study will require approximately 20 months from the time the first participant signs the informed consent until the last participant's last study-related telephone call or visit.<br><br>For purposes of analysis and reporting, the overall study ends when the Sponsor receives the last serology assay result.

Number of Participants:

Approximately 1500 individuals will be randomly assigned in a 3:1 ratio to receive either V114 (1125 participants) or Prevnar 13™ (375 participants) at Visit 2 (Day 1). Approximately 600 participants will be Native American living on or near a reservation in the southwestern United States who are enrolled in the study through clinical sites of Johns Hopkins CAIH.

A subset of Native American participants will be asked to participate in a substudy in which naso- and oropharyngeal swabs and urine samples will be collected to support the development of a novel diagnostic assay for the detection of pneumococcal disease.

Confidential
07Y9YV 054WRH

Enrollment into the substudy will be halted when 300 participants have been enrolled or when general enrollment into the main study at the CAIH sites is completed, whichever event comes first.

Intervention Groups and Duration:

<table>
<tr><td>Intervention Groups</td><td>
<table>
<tr><th>Intervention Group Name</th><th>Vaccine</th><th>Dose Strength</th><th>Dose Frequency</th><th>Route of Admin.</th><th>Vaccination Regimen</th><th>Use</th></tr>
<tr><td rowspan="2">V114</td><td>V114</td><td>Refer to IB</td><td>Single dose</td><td>IM</td><td>Single Dose at Visit 2 (Day 1)</td><td>Experimental</td></tr>
<tr><td>PNEUMOVAX ™23</td><td>Refer to product labeling</td><td>Single dose</td><td>IM</td><td>Single Dose at Visit 4 (Month 6)</td><td>Experimental</td></tr>
<tr><td rowspan="2">Prevnar 13™</td><td>Prevnar 13™</td><td>Refer to product labeling</td><td>Single dose</td><td>IM</td><td>Single Dose at Visit 2 (Day 1)</td><td>Experimental</td></tr>
<tr><td>PNEUMOVAX ™23</td><td>Refer to product labeling</td><td>Single dose</td><td>IM</td><td>Single Dose at Visit 4 (Month 6)</td><td>Experimental</td></tr>
<tr><td colspan="7">B = Investigator's Brochure; IM = intramuscular</td></tr>
</table>
</td></tr>
<tr><td>Total Number</td><td>2 intervention groups</td></tr>
<tr><td>Duration of Participation</td><td>Each participant will participate in the study for approximately 7 months from the time the participant signs the Informed Consent Form (ICF) through the final contact.</td></tr>
</table>

Study Governance Committees:

Steering Committee	No
Executive Oversight Committee	Yes
Data Monitoring Committee	Yes
Clinical Adjudication Committee	No
Study governance considerations are outlined in Appendix 1.	

Study Accepts Healthy Volunteers: Yes

A list of abbreviations used in this document can be found in Appendix 8.

07Y9YV 054WRH
Confidential

1.2 Schema

The key components of the study design are depicted in Figure 1.

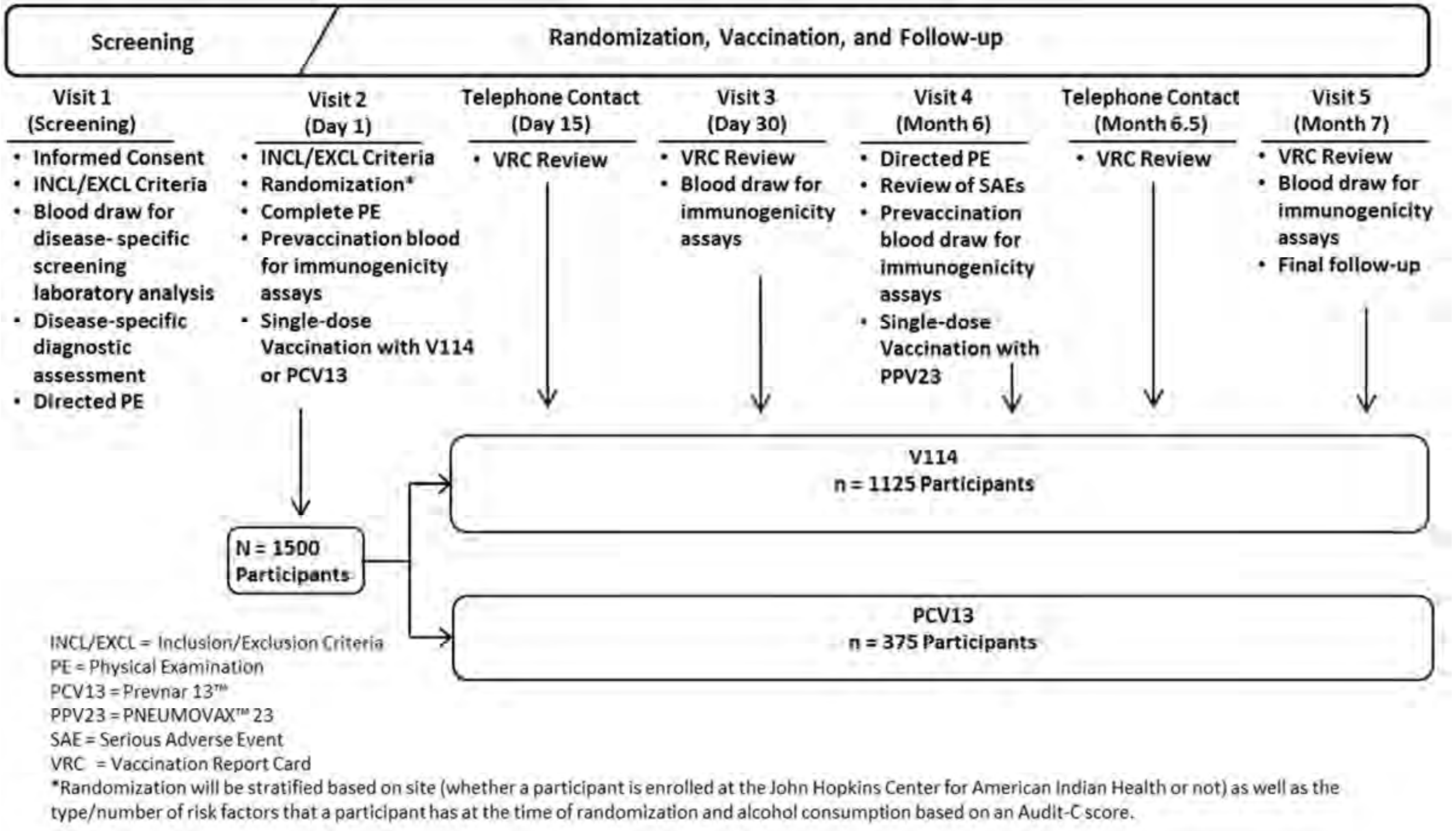

Figure 1 V114-017 Study Design

Confidential

1.3 Schedule of Activities (SoA)

1.3.1 Schedule of Activities for the Main Study

Study Period	Screening	Intervention					Follow-up	
**Visit Number:**	**1**	**2**	**Telephone Contact[a]**	**3**	**4**	**Telephone Contact[a]**	**5**	
**Scheduled Time:**	**Screening**	**Day 1**	**Day 15**	**Day 30**	**Month 6**	**Month 6.5**	**Month 7**	**Comments**
**Visit Window:**		**30 days or less after Visit 1**	**Day 15 to Day 19 after Visit 2[b]**	**Day 30 to Day 44 after Visit 2[b]**	**Day 166 to Day 194 after Visit 2[b]**	**Day 15 to Day 19 after Visit 4[c]**	**Day 30 to Day 44 after Visit 4[c]**	The results from all screening procedures at Visit 1 must be available before enrollment into the study at Visit 2 (Day 1).
**Administrative and General Procedures**								
Informed Consent	X							Consent must be obtained before any study procedures.
Informed Consent for Future Biomedical Research **(Note: Consent and samples will not be collected from Native American participants enrolled via Johns Hopkins Center for American Indian Health [CAIH])**	X							Informed consent for future biomedical research samples must be obtained before the blood sample (DNA sample) is collected.
Assignment of Screening Number	X							
Participant Identification Card	X							
Inclusion/Exclusion Criteria	X	X						
Medical History	X	X						The participant's medical history for the 5 years prior to these visits will be reviewed. History of tobacco use will also be collected for all participants.

Confidential
07Y9YV 054WRH

**Study Period**	**Screening**	**Intervention**					**Follow-up**	
**Visit Number:**	**1**	**2**	**Telephone Contact[a]**	**3**	**4**	**Telephone Contact[a]**	**5**	
**Scheduled Time:**	**Screening**	**Day 1**	**Day 15**	**Day 30**	**Month 6**	**Month 6.5**	**Month 7**	**Comments**
**Visit Window:**		**30 days or less after Visit 1**	**Day 15 to Day 19 after Visit 2[b]**	**Day 30 to Day 44 after Visit 2[b]**	**Day 166 to Day 194 after Visit 2[b]**	**Day 15 to Day 19 after Visit 4[c]**	**Day 30 to Day 44 after Visit 4[c]**	The results from all screening procedures at Visit 1 must be available before enrollment into the study at Visit 2 (Day 1).
**Disease-specific Laboratory and Diagnostic Assessments:** Diabetes mellitus: hemoglobin A1c Chronic liver disease: aspartate aminotransferase, alanine aminotransferase, total bilirubin, prothrombin time/international normalized ratio, albumin, platelets Chronic lung disease: spirometry	X							Spirometry can be performed at screening if prospective participants have a clinical history of COPD or asthma consistent with inclusion criteria but no supporting documentation by spirometry as required by Inclusion Criteria 1c and 1d.
Alcohol Use Disorders Identification Test (AUDIT-C)		X						AUDIT-C results will be used for stratification.
Assignment of Randomization Number		X						
Prior/Concomitant Medication and Non-Study Vaccination Review		X	X	X	X	X	X	
V114 or Prevnar 13™ Administration (Blinded)		X						At Visit 2 (Day 1), participants will receive either a single dose of V114 (or a single dose of Prevnar 13™.
PNEUMOVAX™23 Administration (Open-label)					X			At Visit 4 (Month 6), participants will receive a single dose of PNEUMOVAX™23.
Provide Electronic Vaccination Report Card (eVRC) to Participants Not Enrolled through the CAIH		X			X			Participants will be provided an eVRC at Visit 2 (Day 1) and Visit 4 (Month 6) to record adverse events (AEs) and body temperature measurements. Instructions for using the eVRC will be reviewed with the participant.

Study Period	Screening	Intervention					Follow-up	
Visit Number:	1	2	Telephone Contact[a]	3	4	Telephone Contact[a]	5	
Scheduled Time:	Screening	Day 1	Day 15	Day 30	Month 6	Month 6.5	Month 7	Comments
Visit Window:		30 days or less after Visit 1	Day 15 to Day 19 after Visit 2[b]	Day 30 to Day 44 after Visit 2[b]	Day 166 to Day 194 after Visit 2[b]	Day 15 to Day 19 after Visit 4[c]	Day 30 to Day 44 after Visit 4[c]	The results from all screening procedures at Visit 1 must be available before enrollment into the study at Visit 2 (Day 1).
Provide Paper Vaccination Report Card (VRC) to Native American participants enrolled via Johns Hopkins CAIH		X			X			Participants will be provided a paper VRC at Visit 2 (Day 1) and Visit 4 (Month 6) to record AEs and body temperature measurements. Instructions for using the paper VRC will be reviewed with the participant.
Review Paper VRC/eVRC Data with Participant			X[a]	X		X[a]	X	Site personnel will discuss information entered into the paper VRC/eVRC with participants during the telephone contacts conducted on Day 15 and Month 6.5. A full review of the completed paper VRC/eVRC will occur at Visit 3 (Day 30) and Visit 5 (Month 7).
Collect Paper VRC/eVRC from participant				X			X	
6-month Safety Follow-up Questionnaire					X			Site personnel will review the information in the 6-month Safety Follow-up Questionnaire provided by the Sponsor with the participant. Data to be reported from this discussion will include SAEs and/or any updates to previously reported safety information.
**Safety Procedures**								
Directed Physical Examination	X				X			To be performed by the investigator or medically qualified designee for determination of disease-specific physical assessments at Visit 1 (Screening) and before PNEUMOVAX™23 vaccination at Visit 4 (Month 6).

**Study Period**	**Screening**	**Intervention**					**Follow-up**	**Comments**
**Visit Number:**	**1**	**2**	**Telephone Contact[a]**	**3**	**4**	**Telephone Contact[a]**	**5**	
**Scheduled Time:**	**Screening**	**Day 1**	**Day 15**	**Day 30**	**Month 6**	**Month 6.5**	**Month 7**	
**Visit Window:**		**30 days or less after Visit 1**	**Day 15 to Day 19 after Visit 2[b]**	**Day 30 to Day 44 after Visit 2[b]**	**Day 166 to Day 194 after Visit 2[b]**	**Day 15 to Day 19 after Visit 4[c]**	**Day 30 to Day 44 after Visit 4[c]**	The results from all screening procedures at Visit 1 must be available before enrollment into the study at Visit 2 (Day 1).
Complete Physical Examination		X						To be performed by the investigator or medically qualified designee before study vaccine is administered.
Pregnancy Test – if applicable		X			X			A pregnancy test consistent with local requirements (sensitive to at least 25 IU beta human chorionic gonadotropin [β-hCG]) must be performed before administration of the study vaccine in females who are of reproductive potential. Urine or serum tests can be used, and results must be negative before vaccination can occur. Pregnancy tests can be repeated as needed based on local requirements.
Body Temperature Measurement Before Vaccination		X			X			Each participant's body temperature must be taken before vaccination. Participants who present with fever (oral or tympanic temperature ≥100.4°F [≥38.0°C]; axillary or temporal temperature ≥99.4°F [≥37.4°C]; or rectal temperature ≥101.4°F [≥38.6°C]) will have the vaccination delayed until fever is resolved for 72 hours.

Confidential

07Y9YV 054WRH

**Study Period**	**Screening**	**Intervention**					**Follow-up**	
**Visit Number:**	**1**	**2**	**Telephone Contact[a]**	**3**	**4**	**Telephone Contact[a]**	**5**	
**Scheduled Time:**	**Screening**	**Day 1**	**Day 15**	**Day 30**	**Month 6**	**Month 6.5**	**Month 7**	**Comments**
**Visit Window:**		**30 days or less after Visit 1**	**Day 15 to Day 19 after Visit 2[b]**	**Day 30 to Day 44 after Visit 2[b]**	**Day 166 to Day 194 after Visit 2[b]**	**Day 15 to Day 19 after Visit 4[c]**	**Day 30 to Day 44 after Visit 4[c]**	The results from all screening procedures at Visit 1 must be available before enrollment into the study at Visit 2 (Day 1).
30-Minute Postvaccination Observation Period		X			X			To be performed by blinded study site personnel only.
AE Monitoring	X	X	X	X	X	X	X	AEs (serious and nonserious) are to be reported from Days 1 through 14 following each vaccination. Serious adverse events (SAEs) and deaths are to be reported throughout the duration of an individual's study participation. AEs that occur after the consent form is signed but before allocation/randomization are also to be reported (Section 8.4.1).

Confidential

07Y9YV 054WRH

**Study Period**	**Screening**	**Intervention**					**Follow-up**	
**Visit Number:**	**1**	**2**	**Telephone Contact**[a]	**3**	**4**	**Telephone Contact**[a]	**5**	
**Scheduled Time:**	**Screening**	**Day 1**	**Day 15**	**Day 30**	**Month 6**	**Month 6.5**	**Month 7**	**Comments**
**Visit Window:**		**30 days or less after Visit 1**	**Day 15 to Day 19 after Visit 2**[b]	**Day 30 to Day 44 after Visit 2**[b]	**Day 166 to Day 194 after Visit 2**[b]	**Day 15 to Day 19 after Visit 4**[c]	**Day 30 to Day 44 after Visit 4**[c]	The results from all screening procedures at Visit 1 must be available before enrollment into the study at Visit 2 (Day 1).
**Immunogenicity Procedures**								
Serum for Immunogenicity Assays (including retention serum)		X		X	X		X	Blood samples must be collected before study vaccination when applicable.<br>• After completion of opsonophagocytic (OPA) and electrochemiluminescence (ECL) testing, serum samples will be stored to conduct any additional study-related testing as required by regulatory agencies or the Sponsor. Leftover sera from the study may be used for the development and/or validation of pneumococcal assays after completion of all study-related immunogenicity testing; this applies only to sera received from randomized study participants who provided consent for future biomedical research.<br>• For participants enrolled via the CAIH, any sera remaining after OPA and ECL testing will be returned to the CAIH for destruction.

**Study Period**	**Screening**	**Intervention**					**Follow-up**	
**Visit Number:**	**1**	**2**	**Telephone Contact**[a]	**3**	**4**	**Telephone Contact**[a]	**5**	
**Scheduled Time:**	**Screening**	**Day 1**	**Day 15**	**Day 30**	**Month 6**	**Month 6.5**	**Month 7**	**Comments**
**Visit Window:**		**30 days or less after Visit 1**	**Day 15 to Day 19 after Visit 2**[b]	**Day 30 to Day 44 after Visit 2**[b]	**Day 166 to Day 194 after Visit 2**[b]	**Day 15 to Day 19 after Visit 4**[c]	**Day 30 to Day 44 after Visit 4**[c]	The results from all screening procedures at Visit 1 must be available before enrollment into the study at Visit 2 (Day 1).
**Future Biomedical Research**								
Blood (DNA) for Future Biomedical Research		X						Collected from randomized participants who provided consent for future biomedical research (Section 8.8). Native American participants enrolled via the CAIH will not participate in future biomedical research.

[a] For participants enrolled via the CAIH, this visit may be conducted in person at the discretion of the investigator.
[b] When scheduling the Telephone Contact (Day 15), Visit 3 (Day 30), and Visit 4 (Month 6), “Day 1” is the date of Visit 2.
[c] When scheduling the Telephone Contact (Month 6.5) and Visit 5 (Month 7), “Day 1” is the date of Visit 4.

Confidential

07Y9YV 054WRH

1.3.2 Schedule of Activities for Native Americans Enrolled through the Johns Hopkins Center for American Indian Health (CAIH) Participating in the Substudy

**Study Period**	**Screening**	**Intervention**									**Follow-up**	
**Visit Number:**	**1**	**2**	**2A[a]**	**2B[a]**	**2C[a]**	**3**	**4**	**4A[a]**	**4B[a]**	**4C[a]**	**5**	
**Scheduled Time:**	**Screening**	**Day 1**	**Day 3**	**Day 8**	**Day 15**	**Day 30**	**Month 6**	**Day 183**	**Day 188**	**Day 195**	**Month 7**	**Comments**
**Visit Window:**		**30 days or less after Visit 1**	**Day 2 to Day 4 after Visit 2[b]**	**Day 7 to Day 9 after Visit 2[b]**	**Day 15 to Day 17 after Visit 2[b]**	**Day 30 to Day 44 after Visit 2[b]**	**Day 166 to Day 194 after Visit 2[b]**	**Day 2 to Day 4 after Visit 4[c]**	**Day 7 to Day 9 after Visit 4[c]**	**Day 15 to Day 17 after Visit 4[c]**	**Day 30 to Day 44 after Visit 4[c]**	The results from all screening procedures at Visit 1 must be available before enrollment into the study at Visit 2 (Day 1).
**Administrative Procedures and General Procedures**												
Informed Consent (to include consent for nasopharyngeal [NP] and oropharyngeal [OP] swabs and urine collection substudy)	X											Consent must be obtained before any study procedures.
Assignment of Screening Number	X											
Participant Identification Card	X											
Inclusion/Exclusion Criteria	X	X										
Medical History	X	X										The participant's medical history for the 5 years prior to these visits will be reviewed. History of tobacco use will also be collected for all participants.

**Study Period**	**Screening**	**Intervention**									**Follow-up**	
**Visit Number:**	**1**	**2**	**2A[a]**	**2B[a]**	**2C[a]**	**3**	**4**	**4A[a]**	**4B[a]**	**4C[a]**	**5**	
**Scheduled Time:**	**Screening**	**Day 1**	**Day 3**	**Day 8**	**Day 15**	**Day 30**	**Month 6**	**Day 183**	**Day 188**	**Day 195**	**Month 7**	**Comments**
**Visit Window:**		**30 days or less after Visit 1**	**Day 2 to Day 4 after Visit 2[b]**	**Day 7 to Day 9 after Visit 2[b]**	**Day 15 to Day 17 after Visit 2[b]**	**Day 30 to Day 44 after Visit 2[b]**	**Day 166 to Day 194 after Visit 2[b]**	**Day 2 to Day 4 after Visit 4[c]**	**Day 7 to Day 9 after Visit 4[c]**	**Day 15 to Day 17 after Visit 4[c]**	**Day 30 to Day 44 after Visit 4[c]**	The results from all screening procedures at Visit 1 must be available before enrollment into the study at Visit 2 (Day 1).
**Disease-specific Laboratory and Diagnostic Assessments:** Diabetes mellitus: hemoglobin A1c Chronic liver disease: aspartate aminotransferase, alanine aminotransferase, total bilirubin, prothrombin time/international normalized ratio, albumin, platelets Chronic lung disease: spirometry	X											Spirometry can be performed at screening if prospective participants have a clinical history of COPD or asthma consistent with inclusion criteria but no supporting documentation by spirometry as required by Inclusion Criteria 1c and 1d.
Alcohol Use Disorders Identification Test (AUDIT-C)		X										AUDIT-C results will be used for stratification.
Assignment of Randomization Number		X										
Prior/Concomitant Medication and Non-Study Vaccination Review		X			X	X	X			X	X	
V114 or Prevnar 13™ Administration (Blinded)		X										At Visit 2 (Day 1), participants will receive either a single dose of V114 or a single dose of Prevnar 13™.
PNEUMOVAX™23 Administration (Open-label)							X					At Visit 4 (Month 6), all participants will receive a single dose of PNEUMOVAX™23.

**Study Period**	**Screening**	**Intervention**									**Follow-up**	
**Visit Number:**	**1**	**2**	**2A[a]**	**2B[a]**	**2C[a]**	**3**	**4**	**4A[a]**	**4B[a]**	**4C[a]**	**5**	
**Scheduled Time:**	**Screening**	**Day 1**	**Day 3**	**Day 8**	**Day 15**	**Day 30**	**Month 6**	**Day 183**	**Day 188**	**Day 195**	**Month 7**	**Comments**
**Visit Window:**		**30 days or less after Visit 1**	**Day 2 to Day 4 after Visit 2[b]**	**Day 7 to Day 9 after Visit 2[b]**	**Day 15 to Day 17 after Visit 2[b]**	**Day 30 to Day 44 after Visit 2[b]**	**Day 166 to Day 194 after Visit 2[b]**	**Day 2 to Day 4 after Visit 4[c]**	**Day 7 to Day 9 after Visit 4[c]**	**Day 15 to Day 17 after Visit 4[c]**	**Day 30 to Day 44 after Visit 4[c]**	The results from all screening procedures at Visit 1 must be available before enrollment into the study at Visit 2 (Day 1).
Provide Paper Vaccination Report Card (VRC) to Participants		X					X					Participants will be provided a paper VRC at Visit 2 (Day 1) and Visit 4 (Month 6) to record adverse events (AEs) and body temperature measurements. Instructions for using the paper VRC will be reviewed with the participant.
Review Paper VRC Data with Participant					X	X				X	X	Site personnel will fully review all information entered onto the paper VRC with CAIH Substudy participants during Visit 2C and Visit 4C. A full review of the completed paper VRC will occur at Visit 3 (Day 30) and Visit 5 (Month 7) if not reviewed and collected at the previous visit.

Confidential
07Y9YV 054WRH

**Study Period**	**Screening**	**Intervention**									**Follow-up**	
**Visit Number:**	**1**	**2**	**2A[a]**	**2B[a]**	**2C[a]**	**3**	**4**	**4A[a]**	**4B[a]**	**4C[a]**	**5**	
**Scheduled Time:**	**Screening**	**Day 1**	**Day 3**	**Day 8**	**Day 15**	**Day 30**	**Month 6**	**Day 183**	**Day 188**	**Day 195**	**Month 7**	**Comments**
**Visit Window:**		**30 days or less after Visit 1**	**Day 2 to Day 4 after Visit 2[b]**	**Day 7 to Day 9 after Visit 2[b]**	**Day 15 to Day 17 after Visit 2[b]**	**Day 30 to Day 44 after Visit 2[b]**	**Day 166 to Day 194 after Visit 2[b]**	**Day 2 to Day 4 after Visit 4[c]**	**Day 7 to Day 9 after Visit 4[c]**	**Day 15 to Day 17 after Visit 4[c]**	**Day 30 to Day 44 after Visit 4[c]**	The results from all screening procedures at Visit 1 must be available before enrollment into the study at Visit 2 (Day 1).
Collect Paper VRC from Participant					X	X				X	X	The paper VRC after the first vaccination can be collected at either Visit 2C or Visit 3, and the paper VRC after the second vaccination can be collected at either Visit 4C or Visit 5 at the discretion of the study personnel. A full review of each completed paper VRC with the participant will occur at the time of collection by the site.
6-month Safety Follow-up Questionnaire							X					Site personnel will review the information in the 6-month Safety Follow-up Questionnaire provided by the Sponsor with the participant. Data to be reported from this discussion will include SAEs and/or any updates to previously reported safety information

**Study Period**	**Screening**	**Intervention**									**Follow-up**	
**Visit Number:**	**1**	**2**	**2A**[a]	**2B**[a]	**2C**[a]	**3**	**4**	**4A**[a]	**4B**[a]	**4C**[a]	**5**	
**Scheduled Time:**	**Screening**	**Day 1**	**Day 3**	**Day 8**	**Day 15**	**Day 30**	**Month 6**	**Day 183**	**Day 188**	**Day 195**	**Month 7**	**Comments**
**Visit Window:**		**30 days or less after Visit 1**	**Day 2 to Day 4 after Visit 2**[b]	**Day 7 to Day 9 after Visit 2**[b]	**Day 15 to Day 17 after Visit 2**[b]	**Day 30 to Day 44 after Visit 2**[b]	**Day 166 to Day 194 after Visit 2**[b]	**Day 2 to Day 4 after Visit 4** [c]	**Day 7 to Day 9 after Visit 4** [c]	**Day 15 to Day 17 after Visit 4** [c]	**Day 30 to Day 44 after Visit 4** [c]	The results from all screening procedures at Visit 1 must be available before enrollment into the study at Visit 2 (Day 1).
**Safety Procedures**												
Directed Physical Examination	X						X					To be performed by the investigator or medically qualified designee at Visit 1 (Screening) for determination of disease-specific physical assessments, and before PNEUMOVAX™23 vaccination at Visit 4 (Month 6).
Complete Physical Examination		X										To be performed by the investigator or medically qualified designee before study vaccine is administered.
Pregnancy Test – if applicable		X					X					A pregnancy test consistent with local requirements (sensitive to at least 25 IU beta human chorionic gonadotropin [β-hCG]) must be performed before administration of the study vaccine in females who are of reproductive potential. Urine or serum tests can be used, and results must be negative before vaccination can occur. Pregnancy tests can be repeated as needed based on local requirements.

**Study Period**	**Screening**	**Intervention**									**Follow-up**	
**Visit Number:**	**1**	**2**	**2A[a]**	**2B[a]**	**2C[a]**	**3**	**4**	**4A[a]**	**4B[a]**	**4C[a]**	**5**	
**Scheduled Time:**	**Screening**	**Day 1**	**Day 3**	**Day 8**	**Day 15**	**Day 30**	**Month 6**	**Day 183**	**Day 188**	**Day 195**	**Month 7**	**Comments**
**Visit Window:**		**30 days or less after Visit 1**	**Day 2 to Day 4 after Visit 2[b]**	**Day 7 to Day 9 after Visit 2[b]**	**Day 15 to Day 17 after Visit 2[b]**	**Day 30 to Day 44 after Visit 2[b]**	**Day 166 to Day 194 after Visit 2[b]**	**Day 2 to Day 4 after Visit 4[c]**	**Day 7 to Day 9 after Visit 4[c]**	**Day 15 to Day 17 after Visit 4[c]**	**Day 30 to Day 44 after Visit 4[c]**	The results from all screening procedures at Visit 1 must be available before enrollment into the study at Visit 2 (Day 1).
Body Temperature Measurement Before Vaccination		X					X					Each participant's body temperature must be taken before vaccination. Participants who present with fever (oral or tympanic temperature ≥100.4°F [≥38.0°C]; axillary or temporal temperature ≥99.4°F [≥37.4°C]; or rectal temperature ≥101.4°F [≥38.6°C]) will have the vaccination delayed until fever is resolved for 72 hours.
30-Minute Postvaccination Observation Period		X					X					To be performed by blinded study site personnel only.
AE Monitoring	X	X	X	X	X	X	X	X	X	X	X	AEs (serious and nonserious) are to be reported from Days 1 through 14 following each vaccination. Serious adverse events (SAEs) and deaths are to be reported throughout the duration of an individual's study participation. AEs that occur after the consent form is signed but before allocation/randomization are also to be reported (Section 8.4.1).

Confidential
07Y9YV 054WRH

**Study Period**	**Screening**	**Intervention**									**Follow-up**	
**Visit Number:**	**1**	**2**	**2A**[a]	**2B**[a]	**2C**[a]	**3**	**4**	**4A**[a]	**4B**[a]	**4C**[a]	**5**	
**Scheduled Time:**	**Screening**	**Day 1**	**Day 3**	**Day 8**	**Day 15**	**Day 30**	**Month 6**	**Day 183**	**Day 188**	**Day 195**	**Month 7**	**Comments**
**Visit Window:**		**30 days or less after Visit 1**	**Day 2 to Day 4 after Visit 2**[b]	**Day 7 to Day 9 after Visit 2**[b]	**Day 15 to Day 17 after Visit 2**[b]	**Day 30 to Day 44 after Visit 2**[b]	**Day 166 to Day 194 after Visit 2**[b]	**Day 2 to Day 4 after Visit 4**[c]	**Day 7 to Day 9 after Visit 4**[c]	**Day 15 to Day 17 after Visit 4**[c]	**Day 30 to Day 44 after Visit 4**[c]	The results from all screening procedures at Visit 1 must be available before enrollment into the study at Visit 2 (Day 1).
**Immunogenicity Procedures**												
Serum for Immunogenicity Assays		X				X	X				X	Blood samples must be collected before study vaccination when applicable. Any sera remaining after OPA and ECL testing will be returned to the CAIH for destruction.
**Substudy Assay Development Procedures**												
NP and OP swab collection for NP/OP colonization testing; urine sample collection for urine antigen detection assay		X	X	X	X	X	X	X	X	X	X	Any samples from NP/OP swab and urine collection remaining after protocol-specified testing has been completed will be returned to the CAIH for destruction or for further testing by CAIH if agreed upon between Native American community and CAIH.

[a] Substudy-specific visit.
[b] When scheduling these visits, “Day 1” is the date of Visit 2.
[c] When scheduling these visits, “Day 1” is the date of Visit 4.

Confidential
07Y9YV 054WRH

2 INTRODUCTION

Merck Sharp & Dohme Corp. (MSD) is developing an investigational 15-valent pneumococcal conjugate vaccine (PCV) (referred to as V114) for prevention of pneumococcal disease caused by the serotypes contained in the vaccine. V114 contains the 13 serotypes (1, 3, 4, 5, 6A, 6B, 7F, 9V, 14, 18C, 19F, 19A, 23F) present in the licensed vaccine Prevnar 13™ (pneumococcal 13-valent conjugate vaccine [diphtheria CRM_{197} protein], Wyeth Pharmaceuticals, a subsidiary of Pfizer, Inc., Philadelphia, PA), plus 2 additional serotypes (22F, 33F).

2.1 Study Rationale

Chronic heart, liver and lung disease, and diabetes increase the risk for invasive and noninvasive pneumococcal disease [Weycker, D., et al 2016] [Curcio, D., et al 2015] [Morton, J. B., et al 2017] [Torres, A., et al 2015]. Immunocompetent persons with 1 or more of these conditions are classified as immunocompetent ‘at risk’ by the Advisory Committee on Immunization Practices (ACIP), in contrast to the ‘high risk’ group of immunocompromised individuals [Centers for Disease Control and Prevention (CDC) 2010].

In the United States (US), the ACIP guidelines have included recommendations for pneumococcal vaccination for adults with diabetes and chronic cardiac, lung, and liver disease since the first pneumococcal polysaccharide vaccine was marketed in 1978 [Center for Disease Control 1978]; asthma and smoking were added as specific risk factors in 2010 [Centers for Disease Control and Prevention (CDC) 2010]. Currently, PNEUMOVAX™23 (pneumococcal vaccine, polyvalent [23-valent], MSD) is the only pneumococcal vaccine recommended by the ACIP for at-risk immunocompetent adults 19 to 64 years of age. However, in other countries, pneumococcal vaccine recommendations for this population also include Prevnar 13™, or sequential vaccination with Prevnar 13™ and PNEUMOVAX™23, as some evidence suggests that functional antibody responses induced by PCVs are superior to those induced by pneumococcal polysaccharide vaccines [Centers for Disease Control and Prevention (CDC) 2010] [Center for Disease Control and Prevention 2012] [Falkenhorst, G., et al 2017] [Australian Government Department of Health 2017] [French High Council for Public Health 2017] [Castiglia, P. 2014] [Jackson, L. A., et al 2013].

The burden of pneumococcal disease is disproportionally high in some Native American communities on tribal land compared to the general US population [Weatherholtz, R., et al 2010]. While the youngest and oldest individuals are at highest risk, elevated rates of invasive pneumococcal disease (IPD) are found throughout all age groups. Among Navajo adults, chronic heart failure, alcohol use, and unemployment are associated with an increased risk of IPD [Watt, J. P., et al 2007]. Many Native American populations experience a high burden of chronic medical conditions and poor socioeconomic risk factors. In 1997, the ACIP recommended routine use of PNEUMOVAX™23 for individuals ages 2 through 64 years of age belonging to Alaska Natives or certain American Indian tribes, in addition to the age-based recommendation for individuals ≥ 65 years [Centers for Disease Control and Prevention 1997]. This recommendation was removed in 2010 due to the routine use of

Confidential
07Y9YV 054WRH

PCVs in infants. However, an option was retained for targeted vaccination of adults living in areas with elevated risk for IPD compared to the general US population [Centers for Disease Control and Prevention (CDC) 2010].

Despite ACIP vaccination recommendations and the herd protection provided to adults due to routine infant PCV immunization, rates of IPD caused by serotypes included in currently marketed pneumococcal vaccines, as well as those not included in any vaccine, remain higher in at-risk adults than in healthy individuals [Pilishvili, T., et al 2014]. The associated morbidity and mortality and healthcare costs are substantial [Weycker, D., et al 2016] [Shea, K. M., et al 2014] and emphasize the need for new preventive strategies, including PCVs with extended serotype coverage.

This clinical study is designed to evaluate the safety, tolerability, and immunogenicity of a single dose of V114 and Prevnar 13™ in immunocompetent, at-risk individuals, followed by sequential administration of PNEUMOVAX™23 6 months later. This study design will assess the safety of V114 for 6 months, while also providing data on the safety and immunogenicity of the sequential administration of PNEUMOVAX23™ in this population. The data from this study will contribute to the overall safety database and immunogenicity profile of V114 to support initial licensure in adults 18 through 49 years of age.

2.2 Background

2.2.1 V114 and Pneumococcal Disease

Refer to the Investigator's Brochure (IB) for V114 for more detailed background, including information on pneumococcal disease burden.

Streptococcus pneumoniae is a significant cause of disease worldwide, with clinical manifestations including pneumonia, meningitis, otitis media, sinusitis, and sepsis. Adults with comorbid conditions, in particular immunocompromised individuals, have a higher incidence of IPD morbidity and mortality in all age groups compared to adults without the comorbid conditions [Lexau, C. A., et al 2005].

Currently licensed pneumococcal conjugate vaccines (eg, Prevnar™, Synflorix™, and Prevnar 13™) were first implemented in infant immunization programs in many countries worldwide. Prevnar™ was first licensed in 2000 and later replaced by Prevnar 13™ in 2009 for the European Union and 2010 for the United States. Although Prevnar 13™ is indicated for children and adults, Synflorix™ is only indicated for children up to 5 years of age. Widespread use of PCVs has reduced the burden of pneumococcal disease caused by the serotypes contained in the vaccines in children who were targeted by the vaccination programs and unvaccinated individuals from other age groups (herd protection) [Centers for Disease Control and Prevention 2008] [Ruckinger, S., et al 2009] [Farrell, D. J, et al 2007] [Pilishvili, Tamara, et al 2010] [Lexau, C. A., et al 2005] [Metlay, J. P., et al 2006] [Whitney, Cynthia G., et al 2003] [Moore, M. R., et al 2015] [Lepoutre, A., et al 2015] [Weiss, S., et al 2015] [Martinelli, D., et al 2014] [Guevara, M., et al 2016] [Waight, P. A., et al 2015] [Jokinen, J., et al 2015] [Palmu, A. A., et al 2015] [Wagenvoort, G. H., et al 2016].

Confidential
07Y9YV 054WRH

Prevnar 13™ was also shown to be efficacious against vaccine-type nonbacteremic pneumococcal pneumonia and IPD in adults ≥65 years of age [Bonten, M. J., et al 2015]. These study results were the basis of the recommendation from the ACIP for the sequential administration of Prevnar 13™ followed at least 12 months later by PNEUMOVAX™23 in adults ≥65 years of age [Tomczyk, S., et al 2014] [Kobayashi, M., et al 2015].

Although cases of IPD have decreased following implementation of PCVs, an increase in IPD caused by serotypes not covered by currently available vaccines has been observed. V114 contains 2 additional serotypes (22F, 33F) compared with Prevnar 13™. The selection of 22F and 33F was primarily based on epidemiological importance of these 2 additional serotypes. Recent data from the US suggest that 22F is the most common serotype not included in Prevnar 13™ in adults ≥18 years of age, causing 13% of IPD cases. Serotype 33F is associated with an additional 5% of IPD cases [Moore, M. R., et al 2015]. Data from the United Kingdom also showed that, in 2013/2014, 22F and 33F are frequent serotypes in adults ≥65 years of age, accounting for approximately 10% and 4% of IPD cases in that age group, respectively [Waight, P. A., et al 2015]. Data from 2014 reported in the 2016 annual epidemiological report on IPD by the European Centre for Disease Prevention and Control showed that both serotypes 22F and 33F are among the most common serotypes causing IPD [European Centre for Disease Prevention and Control 2016].

The serotypes included in V114 will provide broad coverage of the leading serotypes associated with pneumococcal disease worldwide. V114 is designed to meet continuing medical and public health needs for PCVs globally, as well as to address the emergence of pneumococcal disease caused by serotypes not contained in currently licensed PCVs. V114 is designed to offer broader serotype coverage than Prevnar 13™ against pneumococcal disease in both pediatric and adult populations.

2.2.2 Preclinical and Clinical Studies

Refer to the IB for information on completed preclinical and clinical studies conducted with V114. See Appendix 10.7.1 for Country-specific requirements for Russia.

2.2.3 Information on Other Study-related Therapy

2.2.3.1 Prevnar 13™

Refer to approved labeling for detailed background information on Prevnar 13™.

Prevnar 13™ contains all of the pneumococcal serotypes included in Prevnar™ (4, 6B, 9V, 14, 18C, 19F, 23F) plus 6 additional serotypes (1, 3, 5, 6A, 7F, and 19A).

The adult indication was approved based on immune responses elicited by Prevnar 13™ in comparison with PNEUMOVAX™23. A placebo-controlled clinical efficacy trial (Community Acquired Pneumonia Immunization Trial in Adults [CAPiTA]) evaluated the efficacy of Prevnar 13™ against pneumococcal pneumonia and IPD in immunocompetent adults ≥65 years of age. Results from CAPiTA showed that Prevnar 13™ was 45.6% (95% confidence interval [CI]: 21.8% to 62.5%) efficacious against vaccine-type nonbacteremic

Confidential

07Y9YV 054WRH

pneumococcal pneumonia and 75.0% (95% CI: 41.4% to 90.8%) efficacious against vaccine-type IPD in adults ≥65 years of age [Bonten, M. J., et al 2015].

A post hoc analysis of the CAPiTA trial in individuals with defined comorbidities, including diabetes mellitus, chronic heart disease, respiratory or liver disease, asplenia, and smoking, showed higher vaccine efficacy in individuals with diabetes mellitus and a non-significant trend towards lower efficacy in people with respiratory diseases [Huijts, S. M., et al 2017].

Prevnar™ and Prevnar 13™ are also known as Prevenar™ and Prevenar 13™ in many countries outside of the US; these vaccines are referred to as Prevnar™ and Prevnar 13™ throughout this document.

2.2.3.2 PNEUMOVAX™23

Refer to approved labeling for detailed background information on PNEUMOVAX™23.

PNEUMOVAX™23 is comprised of the polysaccharides from 23 of the most important serotypes causing disease in adults (1, 2, 3, 4, 5, 6B, 7F, 8, 9N, 9V, 10A, 11A, 12F, 14, 15B, 17F, 18C, 19A, 19F, 20, 22F, 23F, and 33F). The formulation is not adjuvanted and no carrier protein is used.

In the US, PNEUMOVAX™23 is recommended by the ACIP for routine use in immunocompetent adults ≥65 years of age as sequential administration at least 1 year after Prevnar 13™ [Kobayashi, M., et al 2015]. It is also recommended for use in adults aged 19 through 64 years of age with underlying medical conditions that increase the risk for serious pneumococcal infection. These conditions include, but are not limited to, chronic heart disease, chronic lung disease (including chronic obstructive pulmonary disease, emphysema, and asthma), diabetes mellitus, alcoholism, chronic liver disease (including cirrhosis), and cigarette smoking [Centers for Disease Control and Prevention 2010].

PNEUMOVAX™23 is also recommended in immunocompromised adults ≥19 years of age; in this population, a dose of PNEUMOVAX™23 is recommended ≥8 weeks following a dose of Prevnar 13™ [Kobayashi, M., et al 2015]. Many countries follow similar age-based and/or risk-based recommendations for the use of PNEUMOVAX™23 [Castiglia, P. 2014].

2.3 Benefit/Risk Assessment

It cannot be guaranteed that participants in clinical studies will directly benefit from treatment during participation, as clinical studies are designed to provide information about the safety and effectiveness of an investigational vaccine.

Approximately 25% of participants will receive Prevnar 13™, the standard of care, as the active comparator in this study. V114 is expected to provide comparable immune responses to Prevnar 13™ for the shared serotypes while providing additional coverage for the 2 serotypes (22F, 33F) unique to V114. It is unknown if the investigational V114 will have the same benefit as Prevnar 13™.

Additional details regarding specific benefits and risks for participants participating in this clinical study may be found in the accompanying IB and Informed Consent Form (ICF).

3 HYPOTHESIS, OBJECTIVES, AND ENDPOINTS

Objectives and endpoints described below will be evaluated in pneumococcal vaccine-naïve participants 18 to 49 years of age (inclusive) who are at increased risk for pneumococcal disease due to (1) an underlying medical condition, (2) behavioral habits such as smoking, or (3) living in a community/environment with increased risk of disease transmission.

Objectives	Endpoints
Primary	
• **Objective**: To evaluate the safety and tolerability of V114 and Prevnar 13™ with respect to the proportion of participants with adverse events (AEs) within each vaccination group separately.	Following vaccination with V114 or Prevnar 13™:<br>• Solicited injection-site AEs from Day 1 through Day 5 postvaccination<br>• Solicited systemic AEs from Day 1 through Day 14 postvaccination<br>• Vaccine-related serious adverse events (SAEs) from Day 1 to Month 6
• **Objective:** To evaluate the serotype-specific opsonophagocytic activity (OPA) Geometric Mean Titers (GMTs) at 30 days postvaccination (Day 30) with V114 and Prevnar 13™ within each vaccination group separately.	• Serotype-specific OPA responses for the 15 serotypes in V114 at Day 30
Secondary	
• **Objective:** To evaluate the safety and tolerability of PNEUMOVAX™23 administered 6 months following V114 and following Prevnar 13™ with respect to the proportion of participants with AEs within each vaccination group separately.	Following vaccination with PNEUMOVAX™23:<br>• Solicited injection-site AEs from Day 1 through Day 5 postvaccination<br>• Solicited systemic AEs from Day 1 through Day 14 postvaccination<br>• Vaccine-related SAEs from Month 6 to Month 7

Confidential
07Y9YV 054WRH

Objectives	Endpoints
• **Objective:** To evaluate the serotype-specific Immunoglobulin G (IgG) Geometric Mean Concentrations (GMCs) at 30 days postvaccination (Day 30) with V114 and Prevnar 13™ within each vaccination group separately.	• Serotype-specific IgG responses for the 15 serotypes in V114 at Day 30
• **Objective:** To evaluate the serotype-specific Geometric Mean Fold Rises (GMFRs) and proportions of participants with a ≥4-fold rise from prevaccination (Day 1) to 30 days postvaccination (Day 30) for both OPA and IgG responses for participants administered V114 and for participants administered Prevnar 13™ within each vaccination group separately.	• Serotype-specific OPA and IgG responses for the 15 serotypes in V114 at Day 1 and Day 30
• **Objective:** To evaluate the serotype-specific (1) OPA GMTs and IgG GMCs at 30 days postvaccination with PNEUMOVAX™23 (Month 7), (2) GMFRs and proportions of participants with a ≥4-fold rise from prevaccination (Day 1) to 30 days postvaccination with PNEUMOVAX™23 (Month 7) for both OPA and IgG responses, (3) GMFRs and proportions of participants with a ≥4-fold rise from prevaccination with PNEUMOVAX™23 (Month 6) to 30 days postvaccination with PNEUMOVAX™23 (Month 7) for both OPA and IgG responses for participants administered V114 and separately for participants administered Prevnar 13™ 6 months before receipt of PNEUMOVAX™23.	• Serotype-specific OPA and IgG responses for the 15 serotypes in V114 at Day 1, Month 6, and Month 7

Confidential
07Y9YV 054WRH

Objectives	Endpoints
Tertiary/Exploratory	
• **Objective:** To evaluate the serotype-specific OPA GMTs and IgG GMCs at 30 days postvaccination (Day 30) with V114 compared with Prevnar 13™.	• Serotype-specific OPA and IgG responses for the 15 serotypes in V114 at Day 30
• **Objective:** To evaluate the serotype-specific OPA GMTs and IgG GMCs at 30 days postvaccination with PNEUMOVAX™23 (Month 7) for participants administered V114 compared with participants administered Prevnar 13™ 6 months before receipt of PNEUMOVAX™23.	• Serotype-specific OPA and IgG responses for the 15 serotypes in V114 at Month 7

4 STUDY DESIGN

4.1 Overall Design

4.1.1 Main Study

Approximately 1500 individuals will be randomly assigned in a 3:1 ratio to receive either V114 (1125 participants) or Prevnar 13™ (375 participants) on Visit 2 (Day 1). All participants will also receive a single dose of PNEUMOVAX™23 at Visit 4 (Month 6).

Of the 1500 participants, approximately 600 will be Native American participants enrolled through clinical sites of the Johns Hopkins Center for American Indian Health (CAIH).

Participants must belong to at least 1 of the following risk categories:

1. Native American enrolled through a CAIH site
2. diabetes mellitus
3. chronic liver disease
4. chronic lung disease
5. chronic heart disease
6. current smoker

Confidential
07Y9YV 054WRH

Participants with underlying chronic lung, liver, or heart disease, diabetes, or smoking are to meet disease-specific enrollment criteria as outlined in Section 5.1 and are to be on stable treatment for their medical condition for at least 3 months, without any anticipated treatment changes.

Participants will be followed for local and systemic AEs through Day 14 following each vaccination. Information for SAEs and deaths, regardless of whether the events are considered to be vaccine-related by the investigator, will be collected from the time consent is signed through completion of participation in the study. An external Data Monitoring Committee (DMC) will conduct a periodic review of safety and tolerability data for the adult V114 Phase 3 program. A description of the structure, function, and guidelines for decision-making by the DMC, along with the timing and content of the safety reviews, will be outlined in the DMC charter. Information regarding the composition of the DMC is provided in Appendix 1.

Blood samples for immunogenicity assays will be drawn immediately before V114 or Prevnar 13™ vaccination at Visit 2 (Day 1), at 30 days postvaccination at Visit 3 (Day 30), immediately before PNEUMOVAX™23 vaccination at Visit 4 (Month 6), and 30 days postvaccination at Visit 5 (Month 7).

For participants NOT enrolled via the CAIH, after completion of OPA and electrochemiluminescence (ECL) testing, serum samples will be stored to conduct any additional study-related testing as required by regulatory agencies or the Sponsor. Leftover sera from the study may be used for the development and/or validation of pneumococcal assays after completion of all study-related immunogenicity testing; this applies only to sera received from randomized study participants who provided consent for future biomedical research.

For participants enrolled via the CAIH, any sera remaining after OPA and ECL testing will be returned to the CAIH for destruction. Based on community preference, Native American participants enrolled via the CAIH will not participate in future biomedical research.

Specific procedures to be performed during the study, as well as their prescribed times and associated visit windows, are outlined in the Schedule of Activities (SoA) in Section 1.3. Details of each procedure are provided in Section 8.

4.1.2 Substudy

The Sponsor is developing a multivalent pneumococcal serotype-specific urine antigen detection assay (UAD) using serotype-specific monoclonal capture and detection antibodies in a sandwich-binding format on the Luminex platform as a tool for diagnosing invasive and non-invasive pneumococcal disease due to serotypes contained in V114. This substudy will be conducted in the Native American cohort enrolled via CAIH due to higher nasopharyngeal (NP) carriage rates in this population than the general population and due to feasibility of frequent sample collection via home visits. Each Native American participant enrolled through CAIH sites will be asked to participate in a substudy and enrollment will be halted

Confidential
07Y9YV 054WRH

either when 300 participants have been enrolled in the substudy or when enrollment at CAIH sites is completed for the main study, whichever event comes first.

In addition to the procedures prescribed for the main study, substudy procedures will include the collection of NP and oropharyngeal (OP) swabs and urine samples at defined time points before and after each vaccine administration. The combined procedures from the main study and the substudy are outlined in the Substudy SoA in Section 1.3.2. Samples will be collected within 10 days before each vaccine administration (V114 or Prevnar 13™, and PNEUMOVAX™23) for baseline measurements and at the postvaccination visits indicated in the Substudy SoA.

This substudy will evaluate whether vaccination with V114 or with Prevnar 13™ and/or NP/OP colonization with *S. pneumoniae* will lead to a positive UAD for any of the 15 pneumococcal serotypes included in V114. The rationale is based on experiences with other pneumococcal UADs. It has been shown that pneumococcal vaccination can turn the commercially available BinaxNOW™ *S. pneumoniae*, which detects the C-polysaccharide common to different pneumococcal serotypes, as well as investigative serotype-specific UADs positive [Priner, M., et al 2008] [Grijalva, C. G., et al 2015]. Furthermore, NP colonization with *S. pneumoniae* can lead to a positive BinaxNOW™ *S. pneumoniae* in adults and children and to a positive serotype-specific UAD in children [Esposito, S., et al 2004] [Turner, P., et al 2011] [Knoll, M. D., et al 2016]. The impact of NP/OP colonization on serotype-specific UADs in adults is unknown. In this substudy, an NP/OP swabs will be evaluated for the presence of pneumococcal serotypes via polymerase chain reaction testing [Gillis, H. D., et al 2017].

An initial assessment on the day of vaccination will establish a baseline for each participant. It is unknown for how long pneumococcal antigens could be excreted in urine after vaccination. Therefore, multiple postvaccination samples will be taken to be able to provide estimates for the duration of the impact of vaccination on UAD performance. A UAD which turns positive for 1 or more serotypes in the days following vaccination in the absence of NP/OP colonization with the same serotype(s) or clinical pneumococcal disease is likely due to the pneumococcal vaccine administered.

There are no hypotheses associated with the NP/OP colonization and urine antigen testing conducted within this substudy. The results will be summarized in a report separate from the Clinical Study Report (CSR).

4.2 Scientific Rationale for Study Design

After infancy, the risk for pneumococcal disease increases with age and many guidelines include age-based criteria for pneumococcal immunization recommendations. However, chronic medical conditions can also predispose younger adults to pneumococcal disease [Weycker, D., et al 2016]. With a rise of obesity and metabolic syndrome-related illnesses, many individuals are suffering from more than 1 of these chronic diseases. Risk stacking and advanced disease stages are associated with disproportionally higher occurrence of pneumococcal infections and associated mortality in these individuals [Curcio, D., et al

Confidential
07Y9YV 054WRH

2015] [Morton, J. B., et al 2017] [Torres, A., et al 2015]. It is therefore important to evaluate the safety and immunogenicity of V114 in this population.

This study will investigate the safety, tolerability, and immunogenicity of V114 in pneumococcal vaccine-naïve participants 18 to 49 years of age (inclusive) who are at increased risk for pneumococcal disease due to (1) an underlying medical condition, (2) behavioral habits such as smoking, or (3) living in a community/environment with increased risk of disease transmission (Native American living on or around a reservation in the southwestern United States who is enrolled through clinical sites of the CAIH). The study population is representative of individuals with high morbidity due to pneumococcal disease, who may benefit from pneumococcal vaccination. Alcohol use increases the risk for pneumococcal disease but its impact on the immune response to pneumococcal vaccines is not well described [Bhatty, M., et al 2011] [McMahon, B. J., et al 1993]. Enrollment in this study will be stratified by the level of alcohol consumption of the participant based on results of the Alcohol Use Disorders Identification Test (AUDIT-C) screening.

Individuals with end-stage heart, liver or lung disease and those with uncontrolled diabetes will be excluded from this study in order to allow them to prioritize their medical care over participation in this study and to avoid interference of medical treatment with the safety and immunogenicity of the vaccine.

See Appendix 10.7.1 for Country-specific requirements for Russia.

4.2.1 Rationale for Endpoints

4.2.1.1 Immunogenicity Endpoints

Sera from participants will be used to measure vaccine-induced, serotype-specific OPA and IgG responses for all 15 serotypes (1, 3, 4, 5, 6A, 6B, 7F, 9V, 14, 18C, 19A, 19F, 22F, 23F, and 33F) included in V114 using the Multiplexed Opsonophagocytic Assay (MOPA) assay and pneumococcal electrochemiluminescence (PnECL) assay.

Several studies have shown a positive correlation between serotype-specific IgG antibody concentrations and OPA titers in children and adults [Centers for Disease Control and Prevention 2010] [Anttila, M., et al 1999] [Romero-Steiner, S., et al 1997]. OPA assesses levels of functional antibodies capable of opsonizing pneumococcal capsular polysaccharides for presentation to phagocytic cells for engulfment and subsequent killing, and therefore, is considered an important immunologic surrogate for protection against IPD in adults. Note that no serotype-specific IgG concentration or OPA titer have been defined as the threshold value that correlates with protection in adults.

Details on the immunogenicity endpoints evaluated in this study can be found in Section 9.4.1.

Confidential
07Y9YV 054WRH

4.2.1.2 Safety Endpoints

The safety endpoints evaluated in this study were selected based on the product's safety profile demonstrated in previous studies, published data from marketed PCVs, and feedback received from regulatory agencies during product development.

Vaccination report cards (VRCs) will be used to record AEs during the postvaccination periods, as defined in Section 8.1.9. Participants enrolled via the CAIH will use paper VRCs and all other subjects will use electronic VRCs (eVRCs). This choice is based on community preference within the CAIH sites.

The VRCs were structured as recommended in the final Food and Drug Administration Patient Reported Outcome Guidance [U.S. Food and Drug Administration 2009].

Details on the safety endpoints evaluated in this study can be found in Section 8.3.4 and Section 9.4.2.

Details on AEs, including definitions and reporting requirements, can be found in Appendix 3.

4.2.1.3 Future Biomedical Research

The Sponsor will conduct future biomedical research on specimens for which consent was provided during this study. This research may include genetic analyses (DNA), gene expression profiling (ribonucleic acid [RNA]), proteomics, metabolomics (serum, plasma), and/or the measurement of other analytes, depending on which specimens are consented for future biomedical research.

Such research is for biomarker testing to address emergent questions not described elsewhere in the protocol (as part of the main study) and will only be conducted on specimens from appropriately consented participants. The objective of collecting/retaining specimens for future biomedical research is to explore and identify biomarkers that inform the scientific understanding of diseases and/or their therapeutic treatments. The overarching goal is to use such information to develop safer, more effective drugs/vaccines, and/or to ensure that participants receive the correct dose of the correct drug/vaccine at the correct time. The details of this future biomedical research substudy are presented in Appendix 6.

Native American participants enrolled via the CAIH will not participate in future biomedical research.

4.2.2 Rationale for the Use of Comparator/Placebo

This study utilizes Prevnar 13™ as the active comparator, as this is the only PCV licensed in many developed countries for use in adults.

Prevnar 13™ is approved for adults 18 years of age and older for the prevention of pneumococcal pneumonia and invasive disease caused by 13 *S. pneumoniae* serotypes (1, 3, 4, 5, 6A, 6B, 7F, 9V, 14, 18C, 19A, 19F, and 23F).

Confidential
07Y9YV 054WRH

Refer to approved labeling for detailed background information on Prevnar 13™.

4.3 Justification for Dose

The dosing regimen of V114 is similar to that used in previous adult V114 clinical studies in which 1 dose has resulted in a robust immune response (refer to the V114 IB for more detailed information on dose selection).

The doses of Prevnar 13™ and PNEUMOVAX™23 selected for use in this study are the approved doses.

4.4 Beginning and End of Study Definition

The overall study begins when the first participant signs the ICF. The overall study ends when the last participant completes the last study-related telephone-call or visit, withdraws from the study, or is lost to follow-up (ie, the participant is unable to be contacted by the investigator).

For purposes of analysis and reporting, the overall study ends when the Sponsor receives the last serology assay result.

4.4.1 Clinical Criteria for Early Study Termination

There are no prespecified criteria for terminating the study early.

5 STUDY POPULATION

Male/Female participants between the ages of 18 and 49 years (inclusive) who are at increased risk for pneumococcal disease due to (1) an underlying medical condition, (2) behavioral habits such as smoking, or (3) living in a community/environment with increased risk of disease transmission will be enrolled in this study.

Prospective approval of protocol deviations to recruitment and enrollment criteria, also known as protocol waivers or exemptions, is not permitted.

5.1 Inclusion Criteria

Participants are eligible to be included in the study only if all of the following criteria apply:

1. Native American from clinical sites of the CAIH in good health without any of the risk conditions for pneumococcal disease listed below (any underlying chronic illness must be documented to be in stable condition);

   OR

Native American from clinical sites of the CAIH **OR** a participant from sites other than the CAIH with ≥1 of the following risk conditions for pneumococcal disease:

a. Diabetes mellitus Type 1 or Type 2, receiving treatment with at least 1 approved anti-diabetic medication; with hemoglobin A1c (HgA1c) <10% at Screening (Visit 1).

b. Chronic liver disease with compensated cirrhosis (Child-Pugh Class A) due to non-alcoholic fatty liver disease, chronic hepatitis B, chronic hepatitis C, or alcoholic liver disease, diagnosed by clinician's assessment, with at least 1 of the following liver staging assessments (**Note:** In cases where more than 1 staging assessment is available, only 1 result may be used to determine fibrosis status. Biopsy should be preferred over every other test and FibroScan® over the blood test based assessment. Participants with hepatitis C must have either completed a course of treatment with direct antiviral therapy and had a 12-week treatment-free follow-up or be expected to remain untreated during the duration of this study):

   i. Prior liver biopsy demonstrating cirrhosis

   ii. FibroScan® performed with an interpretable score >12.5 kPa within 5 years of Visit 1 (Screening).

   iii. A FibroTest® (FibroSure®) with Fibrosis Score >0.75 performed within 5 years of Visit 1 and an aspartate aminotransferase (AST): platelet ratio index (APRI) of >2 at Visit 1 (Screening) *APRI formula: AST ÷ lab upper limit of normal (ULN) for AST x 100 ÷ (platelet count ÷ 100) (APRI calculation to be provided by the central laboratory)*.

   iv. Prior imaging study with evidence of cirrhosis and an APRI of >2 at Visit 1 (Screening)

   v. Prior imaging study with evidence of cirrhosis with splenomegaly and platelet count <120,000/µL at Visit 1 (Screening)

c. Confirmed diagnosis of chronic obstructive pulmonary disease (COPD) with spirometric data in the preceding 5 years showing post-bronchodilator forced expiratory volume in 1 second (FEV_1) over forced vital capacity (FVC) ratio (FEV_1/FVC) <0.7, and FEV_1 ≥30% predicted, corresponding to spirometric Global Initiative for Chronic Obstructive Lung Disease Stage 1 to 3 by severity assessment. For otherwise eligible individuals with a clinical history of COPD who do not have spirometry data from the preceding 5 years, spirometry can be performed at Visit 1 during the screening process by adequately trained personnel according to accepted guidelines.

d. Confirmed diagnosis of mild or moderate persistent asthma with documented reversible airflow obstruction on spirometry consistent with guidelines for the diagnosis of asthma performed within 5 years prior to Visit 1, and receipt of

Confidential

07Y9YV 054WRH

guideline-directed therapy for mild to moderate asthma. For otherwise eligible individuals with a clinical history of asthma who are receiving guideline directed therapy for mild to moderate asthma but do not have spirometry data from the previous 5 years, spirometry can be performed at Visit 1 during the screening process by adequately trained personnel according to accepted guidelines for assessing reversibility. Inhaled bronchodilator and/or inhaled steroid therapy should be withheld prior to spirometry for a “wash-out” as per recommended guidelines.

e. Confirmed diagnosis of chronic heart disease due to 1 of the following conditions documented within the last 5 years (**Note**: Participants must have New York Heart Association (NYHA) heart failure Class 1 to 3 at Visit 1 (Screening) and receive guideline directed oral heart failure treatment):

   i. Heart failure with reduced ejection fraction (EF): transthoracic echocardiography (TTE) or transesophageal echocardiography (TEE) with left ventricular EF $<40\%$

   ii. Heart failure with preserved EF as diagnosed by a cardiologist based on clinical signs and symptoms, a TTE/TEE with left ventricular EF $\geq 50\%$, and either 1) plasma brain natriuretic peptide (BNP) >100 pg/mL or NT-proBNP >300 pg/mL or 2) at least 1 prior hospitalization or emergency room visit for heart failure

   iii. Non-cyanotic congenital heart disease

f. Current smoker who has smoked at least 100 cigarettes during lifetime, is currently smoking every day or most days of the week, and is not currently receiving smoking cessation therapy at Visit 1 (Screening) or planning to receive therapy during the study.

2. Receiving stable medical management for the conditions listed in Inclusion Criterion 1a-f, if applicable, for at least 3 months with no anticipated major change expected for the duration of the study.

Demographics

3. Participant is male or female 18 to 49 years of age (inclusive).

Female Participants

4. A female participant is eligible to participate if she is not pregnant (Appendix 5), not breastfeeding, and at least 1 of the following conditions applies:

   a. Not a woman of childbearing potential (WOCBP) as defined in Appendix 5.

      OR

b. A WOCBP who agrees to use 1 of the contraceptive methods as defined in Appendix 5 during the treatment period and for at least 6 weeks after the last dose of study intervention.

Informed Consent

5. The participant provides written informed consent for the study. The participant may also provide consent for future biomedical research. However, the participant may participate in the main study without participating in future biomedical research.

   Native American participants enrolled via the CAIH will not participate in future biomedical research.

5.2 Exclusion Criteria

Participants are excluded from the study if any of the following criteria apply:

Medical Conditions

1. History of active hepatitis with elevation in pretreatment aspartate transaminase (AST) or alanine transaminase (ALT) values >5 times the ULN within 3 months of Visit 1 (Screening).

2. History of diabetic ketoacidosis or >1 episode of severe, symptomatic hypoglycemia within 3 months of Visit 1 (Screening).

3. Myocardial infarction, acute coronary syndrome, transient ischemic attack, or ischemic or hemorrhagic stroke within 3 months of Visit 1 (Screening).

4. History of severe pulmonary hypertension with World Health Organization functional class ≥3 or history of Eisenmenger syndrome.

5. History of IPD (positive blood culture, positive cerebrospinal fluid culture, or positive culture at another sterile site) or known history of other culture-positive pneumococcal disease within 3 years of Visit 2 (Day 1).

6. Known hypersensitivity to any component of pneumococcal polysaccharide vaccine, PCV, or any diphtheria toxoid-containing vaccine.

7. Known or suspected impairment of immunological function including, but not limited to a history of congenital or acquired immunodeficiency, documented HIV infection, functional or anatomic asplenia, history of autoimmune disease (including but not limited to the autoimmune conditions outlined in the Investigator Trial File Binder for this study).

Confidential
07Y9YV 054WRH

8. History of malignancy ≤5 years prior to signing informed consent (including hepatocellular carcinoma), except for adequately treated basal cell or squamous cell skin cancer or in situ cervical cancer.

9. History of Stage 4 or 5 Chronic Kidney Disease (Glomerular Filtration Rate below 30 mL/min/1.73 m^2) or nephrotic syndrome.

10. History of alcohol withdrawal or alcohol withdrawal seizure in the past 12 months.

11. History of coagulation disorder contraindicating intramuscular vaccinations.

12. *Recent febrile illness (defined as oral or tympanic temperature ≥100.4°F [≥38.0°C]; axillary or temporal temperature ≥99.4°F [≥37.4°C]; or rectal temperature ≥101.4°F [≥38.6°C]) or received antibiotic therapy for any acute illness occurring within 72 hours before receipt of study vaccine.

13. History of hospitalization within 3 months of Visit 1 (Screening).

14. Planned organ transplantation (heart, liver, lung, kidney, or pancreas) or other planned major surgery during the duration of this study.

15. Expected survival for less than 1 year according to the investigator's judgment.

16. A WOCBP who has a positive urine or serum pregnancy test before the first vaccination at Visit 2 (Day 1).

Prior/Concomitant Therapy

17. Prior administration of any pneumococcal vaccine or expected to receive any pneumococcal vaccine during the study outside of the protocol.

18. Received systemic corticosteroids (prednisone equivalent of ≥20 mg/day) for ≥14 consecutive days and has not completed intervention at least 30 days before study entry.

19. Received systemic corticosteroids exceeding physiologic replacement doses (approximately 5 mg/day prednisone equivalent) within 14 days before vaccination. (**Note**: Topical, ophthalmic, intra-articular or soft-tissue [eg, bursa, tendon steroid injections], and inhaled/nebulized steroids are permitted).

20. Receiving immunosuppressive therapy, including chemotherapeutic agents used to treat cancer or other conditions, and interventions associated with organ or bone marrow transplantation, or autoimmune disease.

21. Receiving immunomodulatory therapy with biological agents such as monoclonal antibodies directed against interleukin or cytokine pathways which could potentially interfere with immunogenicity assessment.

Confidential

07Y9YV 054WRH

22. *Received any licensed, non-live vaccine within the 14 days before receipt of any study vaccine or is scheduled to receive any licensed, non-live vaccine within 30 days following receipt of any study vaccine. Exception: Inactivated influenza vaccine may be administered but must be given at least 7 days before receipt of any study vaccine or at least 15 days after receipt of any study vaccine.

23. *Received any live vaccine within 30 days before receipt of any study vaccine or is scheduled to receive any live vaccine within 30 days following receipt of any study vaccine.

24. Received a blood transfusion or blood products, including immunoglobulins within the 6 months before receipt of study vaccine or is scheduled to receive a blood transfusion or blood product within 30 days of receipt of study vaccine. Autologous blood transfusions are not considered an exclusion criterion.

25. Receiving chronic home oxygen therapy.

Prior/Concurrent Clinical Study Experience

26. Is currently participating in or has participated in an interventional clinical study with an investigational compound or device within 2 months of participating in this current study.

Other Exclusions

27. Is, at the time of signing informed consent, a user of recreational or illicit drugs or has had a recent history (within the last year) of drug abuse or dependence as assessed by the study investigator.

28. Has history or current evidence of any condition, therapy, lab abnormality or other circumstance that might expose the participant to risk by participating in the study, confound the results of the study, or interfere with the participant's participation for the full duration of the study.

29. Is or has an immediate family member (eg, spouse, parent/legal guardian, sibling, or child) who is investigational site or Sponsor staff directly involved with this study.

30. Diabetes mellitus with HgA1c ≥10% at Visit 1 (Screening).

31. Chronic liver disease with Child-Pugh Class B or C cirrhosis at Visit 1(Screening).

32. Chronic lung disease with COPD GOLD Stage 4 or severe persistent asthma at Visit 1 (Screening).

33. Chronic heart disease with NYHA heart failure Class 4 at Visit 1 (Screening).

For items with an asterisk (*), if the participant meets these exclusion criteria, the Day 1 Visit may be rescheduled for a time when these criteria are not met.

Confidential

07Y9YV 054WRH

5.3 Lifestyle Considerations

No lifestyle restrictions are required.

5.4 Screen Failures

Screen failures are defined as participants who consent to participate in the clinical study, but are not subsequently randomized in the study. A minimal set of screen failure information is required to ensure transparent reporting of screen failure participants to meet the Consolidated Standards of Reporting Trials (CONSORT) publishing requirements and to respond to queries from regulatory authorities. Minimal information includes demography, screen failure details, eligibility criteria, and any AEs or SAEs meeting reporting requirements as outlined in the data entry guidelines.

5.5 Participant Replacement Strategy

A participant who withdraws from the study will not be replaced.

6 STUDY INTERVENTION

Study intervention is defined as any investigational intervention(s), marketed product(s), placebo, or medical device(s) intended to be administered to a study participant according to the study protocol.

Clinical supplies [V114, Prevnar 13™, PNEUMOVAX™23] will be packaged to support enrollment. Clinical supplies will be affixed with a clinical label in accordance with regulatory requirements.

6.1 Study Intervention(s) Administered

The study intervention(s) to be used in this study are outlined in Table 1.

07Y9YV 054WRH
Confidential

Table 1 Study Interventions

Arm Name	Arm Type	Intervention Name	Type	Dose Formulation	Unit Dose Strength(s)	Dosage Level(s)	Route of Admin	Vaccination Regimen	Use	IMP/ NIMP	Sourcing
V114	Experimental	V114	Biological /Vaccine	Sterile suspension (prefilled syringe)	Refer to IB	0.5 mL	IM	Single Dose at Visit 2 (Day 1)	Experimental	IMP	Central
		PNEUMOVAX™23	Biological /Vaccine	Sterile solution (prefilled syringe)	Refer to product labeling	0.5 mL	IM	Single Dose at Visit 4 (Month 6)	Experimental	IMP	Central
Prevnar 13™	Active Comparator	Prevnar 13™	Biological /Vaccine	Sterile suspension (prefilled syringe)	Refer to product labeling	0.5 mL	IM	Single Dose at Visit 2 (Day 1)	Experimental	IMP	Central
		PNEUMOVAX™23	Biological /Vaccine	Sterile solution (prefilled syringe)	Refer to product labeling	0.5 mL	IM	Single Dose at Visit 4 (Month 6)	Experimental	IMP	Central

Admin = administration; IB = Investigator's Brochure; IM = intramuscular; IMP = investigational medicinal product; NIMP = non-investigational medicinal product
The definition of IMP and NIMP is based on guidance issued by the European Commission. Regional and/or country differences of the definition of IMP/NIMP may exist. In these circumstances, local legislation is followed.

All supplies indicated in Table 1 will be provided per the "Sourcing" row depending upon local country operational requirements. Every attempt should be made to source these supplies from a single lot/batch number.

Refer to Section 8.1.8 for details regarding administration of the study intervention.

Confidential

6.2 Preparation/Handling/Storage/Accountability

6.2.1 Dose Preparation

There are no specific calculations or evaluations required to be performed in order to administer the proper dose to each participant. The rationale for selection of doses to be used in this study is provided in Section 4.3.

6.2.2 Handling, Storage, and Accountability

The investigator or designee must confirm appropriate temperature conditions have been maintained during transit for all study intervention received, and any discrepancies are reported and resolved before use of the study intervention.

Only participants enrolled in the study may receive study intervention, and only authorized site staff may supply or administer study intervention. All study interventions must be stored in a secure, environmentally controlled, and monitored (manual or automated) area in accordance with the labeled storage conditions with access limited to the investigator and authorized site staff.

The investigator, institution, or the head of the medical institution (where applicable) is responsible for study intervention accountability, reconciliation, and record maintenance (ie, receipt, reconciliation, and final disposition records).

For all study sites, the local country Sponsor personnel or designee will provide appropriate documentation that must be completed for drug accountability and return, or local discard and destruction if appropriate. Where local discard and destruction is appropriate, the investigator is responsible for ensuring that a local discard/destruction procedure is documented.

The study site is responsible for recording the lot number, manufacturer, and expiry date for any locally purchased product (if applicable) as per local guidelines unless otherwise instructed by the Sponsor.

The investigator shall take responsibility for and shall take all steps to maintain appropriate records and ensure appropriate supply, storage, handling, distribution, and usage of study interventions in accordance with the protocol and any applicable laws and regulations.

6.3 Measures to Minimize Bias: Randomization and Blinding

6.3.1 Intervention Assignment

Treatment allocation/randomization will occur centrally using an interactive response technology (IRT) system. There are 2 study intervention arms. Participants will be assigned randomly in a 3:1 ratio to V114 or Prevnar 13™, respectively.

6.3.2 Stratification

Randomization will be stratified based on:

1. whether a participant is enrolled at CAIH (approximately 600 Native American participants, 40% of the total enrollment) or not.

2. the type/number of risk factors for pneumococcal disease a participant has at the time of randomization.

Risk factors for pneumococcal disease include any chronic condition listed under Inclusion Criteria 1a – 1f (Section 5.1). In addition, based on the association of alcohol consumption and pneumococcal disease, an AUDIT-C score ≥5 will count as an additional risk factor for stratification. Note that Native American participants enrolled at CAIH sites can be enrolled in the study without having any of the chronic conditions listed under Inclusion Criteria 1a – 1f. For these participants, the AUDIT-C score can be the only risk factor for stratification. All participants who are not enrolled at CAIH sites must have ≥1 of the chronic conditions listed under Inclusion Criteria 1a – 1f. Therefore, for participants who are not enrolled at CAIH sites, the AUDIT-C score cannot be the only risk factor for stratification. However, an AUDIT-C score ≥5 contributes to the strata for assessment of risk stacking (by placing participants into the strata of 2 or ≥3 risk factors as shown below).

Categories of risk factors for stratified randomization will be defined as follows:

<u>For participants enrolled at CAIH, randomization will be stratified into 1 of the following 9 levels/strata:</u>

- Individuals with only chronic liver disease
- Individuals with only diabetes mellitus
- Individuals with only chronic lung disease
- Individuals with only chronic heart disease
- Individuals who are a current smoker only
- Individuals who only have an AUDIT-C score ≥5
- 2 of the risk factors: chronic liver disease, diabetes mellitus, chronic lung disease, chronic heart disease, current smoker, AUDIT-C score ≥5
- 3 or more of the risk factors: chronic liver disease, diabetes mellitus, chronic lung disease, chronic heart disease, current smoker, AUDIT-C score ≥5
- Individuals with no risk factors who are Native American but do not fall into 1 of the 8 strata above

Confidential
07Y9YV 054WRH

For participants NOT enrolled at CAIH, randomization will be stratified into 1 of the following 7 levels/strata:

- Individuals with only chronic liver disease
- Individuals with only diabetes mellitus
- Individuals with only chronic lung disease
- Individuals with only chronic heart disease
- Individuals who are a current smoker only
- 2 of the risk factors: chronic liver disease, diabetes mellitus, chronic lung disease, chronic heart disease, current smoker, AUDIT-C score ≥5[†]
- 3 or more of the risk factors: chronic liver disease, diabetes mellitus, chronic lung disease, chronic heart disease, current smoker, AUDIT-C score ≥5[†]

[†]While an AUDIT-C score ≥5 cannot be the only risk factor for stratification, an AUDIT-C score ≥5 can contribute to the strata for assessment of risk stacking.

6.3.3 Blinding

A double-blinding technique will be used. V114 and Prevnar 13™ will be prepared and/or dispensed by an unblinded pharmacist or unblinded qualified study site personnel. The participant and the investigator who is involved in the clinical evaluation of the participants will remain blinded to the group assignments.

Because the V114 and Prevnar 13™ have a different appearance, a member of the study site staff will be unblinded for the purposes of receiving, maintaining, preparing and/or dispensing, and administering the study vaccines. PNEUMOVAX™23 will also be prepared and/or dispensed and administered by unblinded study site staff for consistency even though it will be provided open label in this study. In order to avoid bias, the unblinded study personnel will have no further contact with study participants for any study-related procedures/assessments after administration of study vaccines, which includes all safety follow-up procedures. Additionally, blinded site personnel will not be present in the exam room when study vaccines are administered. Contact between participants and unblinded study personnel after vaccination administration is strictly prohibited. Blinded site personnel will be responsible for all safety and immunogenicity follow-up procedures after vaccine administration.

An unblinded Clinical Research Associate will monitor vaccine accountability at the study site. All other Sponsor personnel or delegate(s) and Merck Research Laboratories employees directly involved with the conduct of this study will remain blinded to the participant-level intervention assignment.

See Section 8.1.12 for a description of the method of unblinding a participant during the study should such action be warranted.

Confidential
07Y9YV 054WRH

6.4 Study Intervention Compliance

Interruptions from the protocol specified plan for V114 or Prevnar 13™ vaccination at Visit 2 (Day 1) and PNEUMOVAX™23 vaccination at Visit 4 (Month 6) vaccination require consultation between the investigator and the Sponsor and written documentation of the collaborative decision on participant management.

6.5 Concomitant Therapy

Medications or vaccinations specifically prohibited in the exclusion criteria are not allowed during the ongoing study. If there is a clinical indication for any medication or vaccination specifically prohibited, discontinuation from study intervention may be required. The investigator should discuss any questions regarding this with the Sponsor Clinical Director. The final decision on any supportive therapy or vaccination rests with the investigator and/or the participant's primary physician. However, the decision to continue the participant on study intervention requires the mutual agreement of the investigator, the Sponsor, and the participant.

Listed below (items 1 to 4) are specific restrictions for concomitant therapy or vaccination during the course of the study:

1. Any administration of a non-study pneumococcal vaccine is prohibited during the study.
2. Live and non-live vaccines may only be administered prior to or following the receipt of study vaccine according to the time frames specified in Exclusion Criteria (Section 5.2). **Exception**: Inactivated influenza vaccine may be administered but must be given at least 7 days before receipt of any study vaccine or at least 15 days after receipt of any study vaccine.
3. Participants should not receive systemic corticosteroids (prednisone equivalent of ≥20 mg/day for ≥14 consecutive days) starting from 30 days prior to through 30 days following vaccination. **Note**: Topical, ophthalmic, intra-articular or soft-tissue (eg, bursa, tendon steroid injections), and inhaled/nebulized steroids are permitted.
4. Participants should not receive systemic corticosteroids exceeding physiologic replacement doses (prednisone equivalent dose >5 mg/day) within 14 days before any vaccination. **Note**: Topical, ophthalmic, intra-articular or soft-tissue (eg, bursa, tendon steroid injections), and inhaled/nebulized steroids are permitted.

Any deviation from the above requires consultation between the investigator and the Sponsor and written documentation of the collaborative decision on participant management.

6.5.1 Rescue Medications and Supportive Care

No rescue or supportive medications are specified to be used in this study.

Confidential

07Y9YV 054WRH

6.6 Dose Modification (Escalation/Titration/Other)

No dose modification is allowed in this study.

6.7 Intervention After the End of the Study

There is no study-specified intervention following the end of the study.

6.8 Clinical Supplies Disclosure

This study is blinded but supplies are provided open label; therefore, an unblinded pharmacist or unblinded qualified study site personnel will be used to maintain the blinding of study staff who are directly involved in the clinical evaluation of participants in the study. Study intervention identity (name, strength, or potency) is included in the label text; random code/disclosure envelopes or lists are not provided.

The emergency unblinding call center will use the intervention/randomization schedule for the study to unblind participants and to unmask study intervention identity. The emergency unblinding call center should only be used in cases of emergency (see Section 8.1.12). In the event that the emergency unblinding call center is not available for a given site in this study, the central electronic intervention allocation/randomization system (IRT) should be used to unblind participants and to unmask study intervention identity. The Sponsor will not provide random code/disclosure envelopes or lists with the clinical supplies.

See Section 8.1.12 for a description of the method of unblinding a participant during the study, should such action be warranted.

7 DISCONTINUATION OF STUDY INTERVENTION AND PARTICIPANT WITHDRAWAL

7.1 Discontinuation of Study Intervention

Discontinuation of study intervention does not represent withdrawal from the study.

As certain data on clinical events beyond study intervention discontinuation may be important to the study, they must be collected through the participant's last scheduled follow-up, even if the participant has discontinued study intervention. Therefore, all participants who discontinue study intervention prior to completion of the protocol-specified vaccination regimen will still continue to participate in the study as specified in Section 1.3 and Section 8.12.3 unless the participant withdraws consent (Section 7.2).

Participants may discontinue study intervention at any time for any reason or be discontinued from the study intervention at the discretion of the investigator should any untoward effect occur. In addition, a participant may be discontinued from study intervention by the investigator or the Sponsor if study intervention is inappropriate, the study plan is violated, or for administrative and/or other safety reasons. Specific details regarding procedures to be performed at study intervention discontinuation are provided in Section 8.1.11.

Confidential

07Y9YV 054WRH

A participant must be discontinued from study intervention but continue to be monitored in the study for any of the following reasons:

- The participant requests to discontinue study intervention.
- The participant has a medical condition or personal circumstance which, in the opinion of the investigator and/or Sponsor, placed the participant at unnecessary risk from continued administration of study intervention.
- The participant has a confirmed positive urine or serum pregnancy test before vaccination with PNEUMOVAX™23 at Visit 4 (Month 6).

For participants who are discontinued from study intervention but continue to be monitored in the study, see Section 1.3 and Section 8.12.3 for those procedures to be completed at each specified visit.

Discontinuation from study intervention is “permanent.” Once a participant is discontinued, he/she shall not be allowed to restart study intervention.

7.2 Participant Withdrawal From the Study

A participant must be withdrawn from the study if the participant withdraws consent from the study.

If a participant withdraws from the study, they will no longer receive study treatment or be followed at scheduled protocol visits.

Specific details regarding procedures to be performed at the time of withdrawal from the study, as well as specific details regarding withdrawal from future biomedical research, are outlined in Section 8.1.11. The procedures to be performed should a participant repeatedly fail to return for scheduled visits and/or if the study site is unable to contact the participant are outlined in Section 7.3.

7.3 Lost to Follow-up

If a participant fails to return to the clinic for a required study visit and/or if the site is unable to contact the participant, the following procedures are to be performed:

- The site must attempt to contact the participant and reschedule the missed visit. If the participant is contacted, the participant should be counseled on the importance of maintaining the protocol-specified visit schedule.
- The investigator or designee must make every effort to regain contact with the participant at each missed visit (eg, telephone calls and/or a certified letter to the participant’s last known mailing address or locally equivalent methods). These contact attempts should be documented in the participant’s medical record.
- Note: A participant is not considered lost to follow-up until the last scheduled visit for the individual participant. The missing data for the participant will be managed via the prespecified statistical data handling and analysis guidelines.

Confidential

07Y9YV 054WRH

8 STUDY ASSESSMENTS AND PROCEDURES

- Study procedures and their timing are summarized in the SoA.
- Adherence to the study design requirements, including those specified in the SoA, is essential and required for study conduct.
- The investigator is responsible for ensuring that procedures are conducted by appropriately qualified or trained staff. Delegation of study site personnel responsibilities will be documented in the Investigator Study File Binder (or equivalent).
- All study-related medical decisions must be made by an investigator who is a qualified physician.
- All screening evaluations must be completed and reviewed to confirm that potential participants meet all eligibility criteria. The investigator will maintain a screening log to record details of all participants screened and to confirm eligibility or record reasons for screening failure, as applicable.
- Additional evaluations/testing may be deemed necessary by the investigator and or the Sponsor for reasons related to participant safety. In some cases, such evaluation/testing may be potentially sensitive in nature (eg, HIV, Hepatitis C), and thus local regulations may require that additional informed consent be obtained from the participant. In these cases, such evaluations/testing will be performed in accordance with those regulations.

The approximate blood volumes drawn by study visit and sample type are shown in Table 2 for the main study and Table 3 for the substudy. The maximum amount of blood collected from each participant at each study visit for immunogenicity assessments will not exceed 40 mL. For those participants that consent to future biomedical research, an additional blood sample of approximately 8.5 mL will be taken.

Native American participants enrolled via the CAIH will not participate in future biomedical research.

Repeat or unscheduled samples may be taken for safety reasons or for technical issues with the samples.

C Confidential

07Y9YV 054WRH

Table 2 Approximate Blood Volumes Drawn by Study Visit and by Sample Types for Participants Enrolled at non-CAIH Sites

**Study Visit**	**Visit 1 Screening**	**Visit 2 Day 1**	**Visit 3 Day 30**	**Visit 4 Month 6**	**Visit 5 Month 7**
**Disease-specific laboratory assessments; only for subjects with:**<br>Diabetes mellitus: Hemoglobin A1c<br>Chronic liver disease: AST, ALT, total bilirubin, PT/INR, albumin, platelets	0 to 12 mL	N/A	N/A	N/A	N/A
Serum for immunogenicity assays (including retention serum)	N/A	40 mL	40 mL	40 mL	40 mL
Blood (DNA) for future biomedical research	N/A	8.5 mL	N/A	N/A	N/A
Expected total (mL)	0 to 12 mL	48.5 mL	40 mL	40 mL	40 mL

ALT = alanine aminotransferase; AST = aspartate aminotransferase; DNA = deoxyribonucleic acid; INR = international normalized ratio; N/A = not applicable; PT = prothrombin time

Table 3 Approximate Blood Volumes Drawn by Study Visit and by Sample Types for Participants Enrolled at CAIH Sites

**Study Visit**	**Visit 1 Screening**	**Visit 2 Day 1**	**Visit 3 Day 30**	**Visit 4 Month 6**	**Visit 5 Month 7**
**Disease-specific laboratory assessments; only for subjects with:**<br>Diabetes mellitus: Hemoglobin A1c<br>Chronic liver disease: AST, ALT, total bilirubin, PT/INR, albumin, platelets	0 to 12 mL	N/A	N/A	N/A	N/A
Serum for immunogenicity assays (including retention serum)	N/A	40 mL	40 mL	40 mL	40 mL
Expected total (mL)	0 to12 mL	40 mL	40 mL	40 mL	40 mL

ALT = alanine aminotransferase; AST = aspartate aminotransferase; INR = international normalized ratio; N/A = not applicable; PT = prothrombin time

8.1 Administrative and General Procedures

8.1.1 Informed Consent

The investigator or medically qualified designee (consistent with local requirements) must obtain documented consent from each potential participant prior to participating in a clinical study or future biomedical research. If there are changes to the participant’s status during the study (eg, health or age of majority requirements), the investigator or medically qualified designee must ensure the appropriate consent is in place.

Confidential
07Y9YV 054WRH

8.1.1.1 General Informed Consent

Consent must be documented by the participant's dated signature on a consent form along with the dated signature of the person conducting the consent discussion.

A copy of the signed and dated consent form should be given to the participant before participation in the study.

The initial ICF, any subsequent revised written ICF, and any written information provided to the participant must receive the Institutional Review Board/Independent Ethics Committee's (IRB/IEC's) approval/favorable opinion in advance of use. The participant should be informed in a timely manner if new information becomes available that may be relevant to the participant's willingness to continue participation in the study. The communication of this information will be provided and documented via a revised consent form or addendum to the original consent form that captures the participant's dated signature.

Specifics about a study and the study population will be added to the consent form template at the protocol level.

The informed consent will adhere to IRB/IEC requirements, applicable laws and regulations, and Sponsor requirements.

8.1.1.2 Consent and Collection of Specimens for Future Biomedical Research

The investigator or medically qualified designee will explain the future biomedical research consent to the participant, answer all of his/her questions, and obtain written informed consent before performing any procedure related to the future biomedical research substudy. A copy of the informed consent will be given to the participant.

Native American participants enrolled via the CAIH will not participate in future biomedical research.

8.1.2 Inclusion/Exclusion Criteria

All inclusion and exclusion criteria will be reviewed by the investigator, who is a qualified physician or medically qualified designee, to ensure that the participant qualifies for the study.

If the participant meets any of the Exclusion Criteria with an asterisk (*), Visit 1 (Screening) may be rescheduled for a time when these criteria are not met.

8.1.3 Participant Identification Card

All participants will be given a participant identification card identifying them as participants in a research study. The card will contain study site contact information (including direct telephone numbers) to be used in the event of an emergency. The investigator or qualified designee will provide the participant with a participant identification card immediately after

Confidential
07Y9YV 054WRH

the participant provides written informed consent. At the time of intervention allocation/randomization, site personnel will add the intervention/randomization number to the participant identification card.

The participant identification card also contains contact information for the emergency unblinding call center so that a healthcare provider can obtain information about study intervention in emergency situations where the investigator is not available.

8.1.4 Medical History

A medical history will be obtained by the investigator or qualified designee before vaccination at Visit 2 (Day 1). The participant's medical history for the 5 years prior to Visit 2 (Day 1) will be obtained to ensure that the participant satisfies the inclusion and exclusion criteria of the study. History of tobacco use will also be collected for all participants.

8.1.5 Prior and Concomitant Medications Review

8.1.5.1 Prior Medications

The investigator or qualified designee will review and record prior vaccinations and medications taken by the participant within 30 days before the first dose of study vaccine at Visit 2 (Day 1).

8.1.5.2 Concomitant Medications

The investigator or qualified designee will record medication, if any, taken by the participant during the study.

New and/or concomitant medications taken after Visit 2 (Day 1) and non-study vaccines received since Visit 2 will be recorded with the paper VRC/eVRC as specified in Section 8.3.4.

8.1.6 Assignment of Screening Number

All consented participants will be given a unique screening number that will be used to identify the participant for all procedures that occur prior to randomization. Each participant will be assigned only 1 screening number. Screening numbers must not be re-used for different participants.

Any participant who is screened multiple times will retain the original screening number assigned at the initial screening visit. Specific details on the screening/rescreening visit requirements are provided in Section 8.12.1.

Confidential
07Y9YV 054WRH

8.1.7 Assignment of Treatment/Randomization Number

All eligible participants will be randomly allocated and will receive a treatment/randomization number. The treatment/randomization number identifies the participant for all procedures occurring after treatment allocation/randomization. Once a treatment/randomization number is assigned to a participant, it can never be re-assigned to another participant.

A single participant cannot be assigned more than 1 treatment/randomization number.

8.1.8 Study Intervention Administration

V114 or Prevnar 13™ will be administered at Visit 2 (Day 1). PNEUMOVAX™23 will be administered at Visit 4 (Month 6).

Study vaccines should be removed from the refrigerator no more than 1 hour before vaccination. The time of removal and time of vaccination should be documented in the participant’s chart.

Unblinded study personnel not otherwise involved in the conduct of the study will prepare and administer V114, Prevnar 13™, or PNEUMOVAX™23 at the study site. PNEUMOVAX™23 will be administered open label. Study vaccines should be prepared and administered by appropriately qualified members of the study personnel (eg, physician, nurse, physician’s assistant, nurse practitioner, pharmacist, or medical assistant) as allowed by local/state, country, and institutional guidance.

Prior to administration of V114, the unblinded pharmacist should shake the product vigorously to obtain a homogenous white suspension. If white-colored insoluble particle appears, the unblinded pharmacist should use rapid, horizontal hand shaking for 5 to 10 seconds while holding the syringe in between the thumb and index finger until complete resuspension. This action should be repeated, as necessary. If appearance is otherwise, the vaccine should not be administered. The vaccine should not be used if the vaccine cannot be resuspended.

Study personnel should follow the preparation and administration instructions for Prevnar 13™ and PNEUMOVAX™23 as specified in the product labels.

Study vaccine will be administered as a single 0.5-mL intramuscular injection in the deltoid region of the participant’s arm. Adequate treatment provision, including epinephrine and equipment for maintaining an airway, should be available for immediate use should an anaphylactic or anaphylactoid reaction occur [Centers for Disease Control and Prevention 2015]. Vaccination information, such as Component Identification Number and time of vaccination, must be recorded on the appropriate electronic case report form (eCRF) per the Data Entry Guidelines.

All safety and immunogenicity assessments will be conducted by blinded personnel, and the participant will be blinded to the intervention (ie, V114 or Prevnar 13™) received.

Confidential
07Y9YV 054WRH

8.1.8.1 Timing of Dose Administration

Vaccinations may be administered at any time of day, and without regard to timing of meals.

Each participant's body temperature must be taken before each vaccine administration. Individuals who present with fever (oral or tympanic temperature ≥100.4°F [≥38.0°C]; axillary or temporal temperature ≥99.4°F [≥37.4°C]; or rectal temperature ≥101.4°F [≥38.6°C]) will have the vaccination delayed until fever is resolved for at least 72 hours.

The collection of blood samples and administration of pregnancy tests (if applicable) must be done before each vaccine administration.

All participants will be observed for 30 minutes after each vaccination for any immediate reactions. This observation must be performed by blinded site personnel for all study vaccines (Section 1.3 and Section 6.3.3).

8.1.9 Vaccination Report Card

Participants enrolled via the CAIH will use paper VRCs and all other subjects will use eVRCs. This choice is based on community preference within the CAIH sites.

The eVRC was developed to be administered electronically via a hand-held device. The paper VRCs/eVRCs were structured as recommended in the final Food and Drug Administration Patient-Reported Outcome Guidance [U.S. Food and Drug Administration 2009]. The investigator or delegate will train the participant in the use of the paper VRC and eVRCs at Visit 2 (Day 1) and Visit 4 (Month 6).

Temperatures, injection-site reactions, vaccine-specific complaints, other complaints or illnesses, and concomitant medications or vaccinations will be recorded on the paper VRC/eVRC as described in Section 1.3 and Section 8.3.4.

For the main study, site personnel will discuss information entered into the paper VRC/eVRC with participants during the telephone contacts conducted on Day 15 and Month 6.5. A full review of the completed paper VRC/eVRC will occur at Visit 3 (Day 30) and Visit 5 (Month 7).

For Native Americans enrolled through the CAIH participating in the substudy, site personnel will discuss information entered into the paper VRC with participants during Visit 2C and Visit 4C. The paper VRC can be collected at either Visit 2C or Visit 3, and at either Visit 4C or Visit 5 at the discretion of the study personnel. A full review of the completed paper VRC with the participant will occur at the time of collection by the site.

For the AEs outlined above, the investigator will use the information provided by the participant both on the paper VRC/eVRC, and verbally at the time of paper VRC/eVRC review, to apply the appropriate assessment of intensity and toxicity as described in Appendix 3.

Confidential
07Y9YV 054WRH

8.1.10 6-month Safety Follow-up Questionnaire

Site personnel will review the information in the 6-month Safety Follow-up Questionnaire provided by the Sponsor with the participant. Data to be reported from this discussion will include SAEs and/or any updates to previously reported safety information.

8.1.11 Discontinuation and Withdrawal

Participants who discontinue study intervention prior to completion of the protocol-specified vaccinations should be encouraged to continue to be followed for all remaining study visits.

When a participant withdraws from participation in the study, all applicable activities scheduled for the final study visit should be performed (at the time of withdrawal). Any AEs that are present at the time of withdrawal should be followed in accordance with the safety requirements outlined in Section 8.4.

8.1.11.1 Withdrawal From Future Biomedical Research

Participants may withdraw their consent for future biomedical research. Participants may withdraw consent at any time by contacting the principal investigator for the main study. If medical records for the main study are still available, the investigator will contact the Sponsor using the designated mailbox (clinical.specimen.management@merck.com). Subsequently, the participant's consent for future biomedical research will be withdrawn. A letter will be sent from the Sponsor to the investigator confirming the withdrawal. It is the responsibility of the investigator to inform the participant of completion of withdrawal. Any analyses in progress at the time of request for withdrawal or already performed prior to the request being received by the Sponsor will continue to be used as part of the overall research study data and results. No new analyses would be generated after the request is received.

In the event that the medical records for the main study are no longer available (eg, if the investigator is no longer required by regulatory authorities to retain the main study records) or the specimens have been completely anonymized, there will no longer be a link between the participant's personal information and their specimens. In this situation, the request for specimen withdrawal cannot be processed.

Native American participants enrolled via the CAIH will not participate in future biomedical research.

8.1.12 Participant Blinding/Unblinding

STUDY INTERVENTION IDENTIFICATION INFORMATION IS TO BE UNMASKED ONLY IF NECESSARY FOR THE WELFARE OF THE PARTICIPANT. EVERY EFFORT SHOULD BE MADE NOT TO UNBLIND.

For emergency situations where the investigator or medically qualified designee (consistent with local requirements) needs to identify the drug used by a participant and/or the dosage administered, he/she will contact the emergency unblinding call center by telephone and

make a request for emergency unblinding. As requested by the investigator or medically qualified designee the emergency unblinding call center will provide the information to him/her promptly and report unblinding to the Sponsor. Prior to contacting the emergency unblinding call center to request unblinding of a participant's treatment assignment, the investigator who is a qualified physician should make reasonable attempts to enter the intensity/toxicity grade of the AEs observed, the relation to study intervention, the reason thereof, etc., in the medical chart. If it is not possible to record this assessment in the chart prior to the unblinding, the unblinding should not be delayed.

In the event that unblinding has occurred, the circumstances around the unblinding (eg, date, reason, and person performing the unblinding) must be documented promptly, and the Sponsor Clinical Director notified as soon as possible.

Once an emergency unblinding has taken place, the principal investigator, site personnel, and Sponsor personnel may be unblinded so that the appropriate follow-up medical care can be provided to the participant.

Participants whose treatment assignment has been unblinded by the investigator or medically qualified designee and/or nonstudy treating physician should continue to be monitored in the study. Participants may receive PNEUMOVAX™23 at Visit 4 (Month 6) after a benefit/risk assessment and consultation between the investigator/delegate and Sponsor.

Additionally, the investigator or medically qualified designee must go into the IRT system and perform the unblind in the IRT system to update drug disposition. In the event that the emergency unblinding call center is not available for a given site in this study, the IRT system should be used for emergency unblinding in the event that this is required for participant safety.

8.1.13 Calibration of Equipment

The investigator or qualified designee has the responsibility to ensure that any device or instrument used for a clinical evaluation/test during a clinical study that provides information about inclusion/exclusion criteria and/or safety or efficacy parameters shall be suitably calibrated and/or maintained to ensure that the data obtained is reliable and/or reproducible. Documentation of equipment calibration must be retained as source documentation at the study site.

8.2 Immunogenicity Assessments

Sera from participants will be used to measure vaccine-induced OPA and IgG for serotypes included in V114 and Prevnar 13™. These endpoints will be tested for all blood draws. Blood collection, storage, and shipment instructions for serum samples will be provided in the operations/laboratory manual.

Confidential
07Y9YV 054WRH

The MOPA will be used for measuring OPA GMTs. Opsonization of pneumococci for phagocytosis is an important mechanism by which antibodies to polysaccharides protect against disease in vivo. The OPA assay is a useful tool for assessing the protective function of serotype-specific antibodies and therefore, the immunogenicity of pneumococcal vaccine formulations.

Measurement of serotype-specific IgG will be measured using the PnECL v2.0 assay to assess the concentration of binding antibodies to capsular polysaccharide of *S. pneumoniae* for the serotypes included in the study vaccines.

8.2.1 Multiplex Opsonophagocytic Assay (MOPA)

The MOPA, developed and published by Professor Moon Nahm (Director of the United States World Health Organization pneumococcal serology reference laboratory and National Institutes of Health pneumococcal reference laboratories), is a multiplexed OPA assay capable of measuring 4 serotypes at a time, against a total of 16 serotypes of pneumococci [Burton, Robert L. and Nahm, Moon H. 2006]. The OPA is an antibody-mediated killing assay that measures the ability of human serum to kill *S. pneumoniae* serotypes with the help of complement and phagocytic effector cells. The ability of the assay to simultaneously test 4 serotypes/run reduces the amount of serum needed for testing. The assay readout is the opsonization index, which is the reciprocal of the highest dilution that gives ≥50% bacterial killing, as determined by comparison to assay background controls. MSD has developed and optimized the MOPA in a high throughput micro-colony platform. The MOPA assay for all 15 V114 serotypes has undergone validation. The validation study evaluated various performance parameters of the assay, including precision, relative accuracy/dilutional linearity, and specificity. The validation results were evaluated against prespecified acceptance criteria for each of the parameters.

8.2.2 Electrochemiluminescence (ECL)

MSD has developed and optimized a multiplex, ECL-based detection method for the quantitation of IgG serotype-specific antibodies to the 15 pneumococcal polysaccharide serotypes contained in V114. The PnECL v2.0 assay is based on the Meso-Scale Discovery technology, which employs disposable multi-spot microtiter plates. The benefits of the ECL multiplex technology over the prior enzyme-linked immunosorbent assay methodology include speed, equivalent or better sensitivity, increased dynamic range, the ability to multiplex, and reduction in required serum sample and reagent volumes. The measurement of immune responses to the 15 serotypes included in V114 is performed using an assay format consisting of 2 groups of 7 and 8 serotypes each. The PnECL v2.0 assay for all 15 serotypes has undergone validation. The validation study evaluated various performance parameters of the assay, including precision, ruggedness, relative accuracy, dilutional linearity, selectivity, and specificity. The validation results were evaluated against prespecified acceptance criteria for each of the parameters.

Confidential
07Y9YV 054WRH

8.3 Safety Assessments

Details regarding specific safety procedures/assessments to be performed in this study are provided. The total amount of blood/tissue to be drawn/collected over the course of the study (from prestudy to poststudy visits), including approximate blood/tissue volumes drawn/collected by visit and by sample type per participant, can be found in Section 8.

Planned time points for all safety assessments are provided in the SoA.

8.3.1 Physical Examinations

A complete physical examination will be conducted by an investigator or medically qualified designee (consistent with local requirements) before vaccination at Visit 2 (Day 1).

A directed physical examination will be conducted by an investigator or medically qualified designee (consistent with local requirements) at Visit 1(Screening) to address disease-specific inclusion/exclusion criteria, such as Child-Pugh Class for participants with chronic liver disease and NYHA Class for participants with chronic heart disease. A directed physical examination will also be conducted before vaccination at Visit 4 (Month 6).

Investigators should pay special attention to clinical signs related to previous illnesses.

8.3.2 Pregnancy Test

A pregnancy test consistent with local requirements (sensitive to at least 25 IU beta human chorionic gonadotropin [β-hCG]) must be performed before vaccination at Visit 2 (Day 1) and Visit 4 (Month 6) in WOCBP as described in Section 1.3. Pregnancy tests can be repeated as needed based on local requirements. Urine or serum tests can be used, and results must be negative before vaccination can occur.

8.3.3 Body Temperature Measurement

Each participant's body temperature should be taken before vaccination as described in Section 1.3.

Oral body temperatures will also be documented by participants using their paper VRC/eVRC during the VRC-specified postvaccination follow-up period (Section 8.3.4).

For this study, any oral or tympanic temperature ≥100.4°F (≥38.0°C), axillary or temporal temperature ≥99.4°F (≥37.4°C), or rectal temperature ≥101.4°F (≥38.6°C) will be considered an AE of fever. All fevers must be reported Day 1 through Day 14, unless the fever is a symptom of another reported AE.

8.3.4 Safety Assessments and Use of the Paper VRC/eVRC

All participants will be observed for 30 minutes after each vaccination for any immediate reactions. If any immediate AEs are observed during this period, the time at which the event

07Y9YV 054WRH Confidential

occurred within this timeframe, as well as the event itself, any concomitant medications that were administered, and resolution of the event, must be recorded on the appropriate eCRF.

Participants will use the paper VRC/eVRC (Section 8.1.9) to document the following information:

- Oral body temperatures measured Day 1 (day of vaccination) through Day 5 postvaccination;
- Solicited injection-site AEs (redness/erythema, swelling, tenderness/pain) Day 1 through Day 5 postvaccination;
- Solicited systemic AEs (muscle pain/myalgia, joint pain/arthralgia, headache, tiredness/fatigue) Day 1 through Day 14 postvaccination;
- Any other injection-site or systemic AEs Day 1 through Day 14 postvaccination; and
- Concomitant medications and non-study vaccinations Day 1 to Day 14 postvaccination.

8.3.5 Clinical Laboratory Assessments

- Refer to Appendix 2 for the list of clinical laboratory tests to be performed and to the SoA for the timing and frequency.
- All protocol-required laboratory assessments, as defined in Appendix 2, must be conducted in accordance with the laboratory manual and the SoA.
- If laboratory values from nonprotocol specified laboratory assessments performed at the institution’s local laboratory require a change in study participant management or are considered clinically significant by the investigator (eg, SAE or AE or dose modification), then the results must be recorded in the appropriate CRF (eg, SLAB).
- For any laboratory tests with values considered clinically significantly abnormal during participation in the study or within 30 to 44 days after the last dose of study intervention, every attempt should be made to perform repeat assessments until the values return to normal or baseline or if a new baseline is established as determined by the investigator.

8.4 Adverse Events (AEs), Serious Adverse Events (SAEs), and Other Reportable Safety Events

The definitions of an AE or SAE, as well as the method of recording, evaluating, and assessing causality of AE and SAE and the procedures for completing and transmitting AE, SAE, and other reportable safety event reports can be found in Appendix 3.

Adverse events, SAEs, and other reportable safety events will be reported by the participant (or, when appropriate, by a caregiver or surrogate).

Confidential
07Y9YV 054WRH

The investigator and any designees are responsible for detecting, documenting, and reporting events that meet the definition of an AE or SAE, as well as other reportable safety events. Investigators remain responsible for following up AE, SAEs, and other reportable safety events for outcome according to Section 8.4.3.

The investigator, who is a qualified physician, will assess events that meet the definition of an AE or SAE as well as other reportable safety events with respect to seriousness, intensity/toxicity and causality.

8.4.1 Time Period and Frequency for Collecting AE, SAE, and Other Reportable Safety Event Information

All AEs, SAEs, and other reportable safety events that occur after the consent form is signed but before allocation/randomization must be reported by the investigator if they cause the participant to be excluded from the study, or are the result of a protocol-specified intervention, including but not limited to washout or discontinuation of usual therapy, diet, placebo treatment, or a procedure.

All AEs, SAEs, and other reportable safety events must be reported by the investigator from the day of allocation/randomization to the first vaccination and from the day of each vaccination through 14 days postvaccination. SAEs must also be reported throughout the duration of the individual's participation in the study, regardless of whether or not related to the Sponsor's product.

Additionally, any SAE brought to the attention of an investigator at any time outside of the time period specified in the previous paragraph also must be reported immediately to the Sponsor if the event is either:

1. A death that occurs prior to the participant completing the study.

OR

2. An SAE that is considered by an investigator who is a qualified physician to be vaccine related.

Investigators are not obligated to actively seek AE or SAE or other reportable safety events in former study participants. However, if the investigator learns of any SAE, including a death, at any time after a participant has been discharged from the study, and he/she considers the event to be reasonably related to the study intervention or study participation, the investigator must promptly notify the Sponsor.

All initial and follow-up AEs, SAEs, and other reportable safety events will be recorded and reported to the Sponsor or designee within the time frames as indicated in Table 4.

Confidential
07Y9YV 054WRH

Table 4 Reporting Time Periods and Time Frames for Adverse Events and Other Reportable Safety Events

Type of Event	Reporting Time Period: Consent to Randomization/ Allocation	Reporting Time Period: Randomization/ Allocation through Protocol-Specified Follow-up Period	Reporting Time Period: After the Protocol Specified Follow-up Period	Timeframe to Report Event and Follow-up Information to SPONSOR:
Non-Serious Adverse Event (NSAE)	Report if:<br>- due to protocol-specified intervention<br>- causes exclusion<br>- participant is receiving placebo run-in or other run-in treatment	Report all	Not required	Per data entry guidelines
Serious Adverse Event (SAE)	Report if:<br>- due to protocol-specified intervention<br>- causes exclusion<br>- participant is receiving placebo run-in or other run-in treatment	Report all	Report if:<br>- drug/vaccine related<br>- any death until participant completion of study<br>(Follow ongoing to outcome)	Within 24 hours of learning of event
Pregnancy/Lactation Exposure	Report if:<br>- due to intervention<br>- causes exclusion	Report all	Previously reported – Follow to completion/termination; report outcome	Within 24 hours of learning of event
Event of Clinical Interest	There are no Events of Clinical Interest for this study			Not applicable
Cancer	Report if:<br>- due to intervention<br>- causes exclusion	Report all	Not required	Within 5 calendar days of learning of event
Overdose	Report if:<br>- receiving placebo run-in or other run-in medication	Report all	Not required	Within 5 calendar days of learning of event

8.4.2 Method of Detecting AEs, SAEs, and Other Reportable Safety Events

Care will be taken not to introduce bias when detecting AE and/or SAE and other reportable safety events. Open-ended and nonleading verbal questioning of the participant is the preferred method to inquire about AE occurrence.

Confidential
07Y9YV 054WRH

8.4.3 Follow-up of AE, SAE, and Other Reportable Safety Event Information

After the initial AE/SAE report, the investigator is required to proactively follow each participant at subsequent visits/contacts. All AE, SAE, and other reportable safety events including pregnancy and exposure during breastfeeding, cancer, and overdose will be followed until resolution, stabilization, until the event is otherwise explained, or the participant is lost to follow-up (as defined in Section 7.3). In addition, the investigator will make every attempt to follow all nonserious AEs that occur in randomized participants for outcome. Further information on follow-up procedures is given in Appendix 3.

8.4.4 Regulatory Reporting Requirements for SAE

Prompt notification (within 24 hours) by the investigator to the Sponsor of SAE is essential so that legal obligations and ethical responsibilities towards the safety of participants and the safety of a study intervention under clinical investigation are met.

The Sponsor has a legal responsibility to notify both the local regulatory authority and other regulatory agencies about the safety of a study intervention under clinical investigation. All AEs will be reported to regulatory authorities, IRB/IECs, and investigators in accordance with all applicable global laws and regulations (ie, per ICH Topic E6 (R2) Guidelines for Good Clinical Practice [GCP]).

Investigator safety reports must be prepared for suspected unexpected serious adverse reactions (SUSARs) according to local regulatory requirements and Sponsor policy and forwarded to investigators as necessary.

An investigator who receives an investigator safety report describing an SAE or other specific safety information (eg, summary or listing of SAE) from the Sponsor will file it along with the IB and will notify the IRB/IEC, if appropriate according to local requirements.

8.4.5 Pregnancy and Exposure During Breastfeeding

Although pregnancy and infant exposure during breastfeeding are not considered AEs, any pregnancy or infant exposure during breastfeeding in a participant (spontaneously reported to the investigator or their designee) that occurs during the study are reportable to the Sponsor.

All reported pregnancies must be followed to the completion/termination of the pregnancy. Pregnancy outcomes of spontaneous abortion, missed abortion, benign hydatidiform mole, blighted ovum, fetal death, intrauterine death, miscarriage, and stillbirth must be reported as serious events (Important Medical Events). If the pregnancy continues to term, the outcome (health of infant) must also be reported.

8.4.6 Disease-related Events and/or Disease-related Outcomes Not Qualifying as AEs or SAEs

This is not applicable to this study.

Confidential
07Y9YV 054WRH

8.4.7 Events of Clinical Interest (ECIs)

There are no events of clinical interest for this study.

8.5 Treatment of Overdose

In this study, an overdose is the administration of more than 1 dose of any individual study vaccine in any 24-hour period.

No specific information is available on the treatment of overdose.

Decisions regarding dose interruptions or modifications will be made by the investigator in consultation with the Sponsor Clinical Director based on the clinical evaluation of the participant.

All reports of overdose must be reported by the investigator within 5 calendar days to the Sponsor either by electronic media or paper. Electronic reporting procedures can be found in the electronic data collection (EDC) data entry guidelines. Paper reporting procedures can be found in the Investigator Trial File Binder (or equivalent).

8.6 Pharmacokinetics

PK parameters will not be evaluated in this study.

8.7 Pharmacodynamics

Pharmacodynamic parameters will not be evaluated in this study.

8.8 Future Biomedical Research Sample Collection

If the participant signs the future biomedical research consent, the following specimens will be obtained as part of future biomedical research:

- DNA for future research
- Leftover study serum after completion of immunogenicity testing stored for future research

Native American participants enrolled via the CAIH will not participate in future biomedical research.

8.9 Planned Genetic Analysis Sample Collection

Planned genetic analysis samples will not be evaluated in this study.

8.10 Biomarkers

Biomarkers are not evaluated in this study.

Confidential
07Y9YV 054WRH

8.11 Medical Resource Utilization and Health Economics

Medical Resource Utilization and Health Economics are not evaluated in this study.

8.12 Visit Requirements

Visit requirements are outlined in Section 1.3. Specific procedure-related details are provided in Section 8.

8.12.1 Screening

Screening procedures will be conducted at Visit 1 as outlined in Section 1.3.

8.12.2 Treatment Period/Vaccination Visit

Requirements during the treatment period are outlined in Section 1.3.

8.12.3 Discontinued Participants Continuing to be Monitored in the Study

In this trial, a participant may discontinue from study intervention (ie, vaccination) but continue to participate in subsequent protocol visits as outlined in Section 1.3, as long as the participant does not withdraw consent. Protocol-specified activities, including blood draws for immunogenicity assessments and AE monitoring, should occur at these visits.

9 STATISTICAL ANALYSIS PLAN

This section outlines the statistical analysis strategy and procedures for the study. Changes to analyses made after the protocol has been finalized, but prior to unblinding, will be documented in a supplemental Statistical Analysis Plan and referenced in the CSR for the study. Post hoc exploratory analyses will be clearly identified in the CSR.

Other planned analyses (ie, those specific to the substudy or future biomedical research) are beyond the scope of this document or will be documented in separate analysis plans.

9.1 Statistical Analysis Plan Summary

Key elements of the Statistical Analysis Plan are summarized below; the comprehensive plan is provided in Sections 9.2 through 9.12.

Confidential
07Y9YV 054WRH

**Study Design Overview**	A Phase 3, Multicenter, Randomized, Double-Blind, Active Comparator-Controlled Study to Evaluate the Safety, Tolerability, and Immunogenicity of V114 Followed by Administration of PNEUMOVAX™23 Six Months Later in Immunocompetent Adults Between 18 and 49 Years of Age at Increased Risk for Pneumococcal Disease.
**Intervention Assignment**	Participants will be randomly assigned in a 3:1 ratio to V114 or Prevnar 13™, respectively. Randomization will be stratified based on the categories defined in Section 6.3.2.
**Analysis Populations**	**Immunogenicity**: Per-Protocol (PP)<br>**Safety**: All Participants as Treated (APaT)
**Primary Endpoint(s)**	**Immunogenicity**:<br>• The serotype-specific OPA GMTs at 30 days postvaccination with V114 and Prevnar 13™ (Day 30)<br>**Safety**:<br>• Proportion of participants with solicited injection-site AEs (redness/erythema, swelling, tenderness/pain) from Day 1 through Day 5 following V114 or Prevnar 13™<br>• Proportion of participants with solicited systemic AEs (muscle pain/myalgia, joint pain/arthralgia, headache, and tiredness/fatigue) from Day 1 through Day 14 following V114 or Prevnar 13™<br>• Proportion of participants with vaccine-related SAEs in the 6 months following V114 or Prevnar 13™ from Day 1 through Month 6
**Key Secondary Endpoints**	**Immunogenicity**:<br>• Serotype-specific IgG GMCs at 30 days postvaccination with V114 and Prevnar 13™ (Day 30)<br>• Serotype-specific OPA GMTs and IgG GMCs at 30 days postvaccination with PNEUMOVAX™23 (Month 7)<br>**Safety**:<br>• Proportion of participants with solicited injection-site AEs (redness/erythema, swelling, tenderness/pain) from Day 1 through Day 5 following PNEUMOVAX™23<br>• Proportion of participants with solicited systemic AEs (muscle pain/myalgia, joint pain/arthralgia, headache, and tiredness/fatigue) from Day 1 through Day 14 following PNEUMOVAX™23<br>• Proportion of participants with vaccine-related SAEs in the 30 days following PNEUMOVAX™23 from Month 6 through Month 7

Confidential

07Y9YV 054WRH

**Statistical Methods for Key Immunogenicity Analyses**	Immunogenicity analyses will be conducted for each of the 15 pneumococcal serotypes separately. To address the primary immunogenicity objective, evaluation of the OPA GMTs at 30 days postvaccination with V114 or Prevnar 13™ (Day 30) will include descriptive summaries and within-group 95% CIs will be calculated for each vaccination group.<br><br>A similar statistical approach will be used to evaluate the IgG GMCs at 30 days postvaccination with V114 or Prevnar 13™ (Day 30) and the OPA GMTs and IgG GMCs at 30 days postvaccination with PNEUMOVAX™23 (Month 7).
**Statistical Methods for Key Safety Analyses**	The analysis strategy for safety parameters following each vaccination is described in Section 9.6.2. Safety parameters will be summarized via descriptive statistics. In addition, for select safety parameters, 95% within-group confidence intervals will be provided.
**Interim Analyses**	To support the periodic review of safety and tolerability data across the V114 Phase 3 program, an external unblinded statistician will provide unblinded interim safety summaries to an independent DMC for their review. There are no plans to conduct an interim analysis of unblinded immunogenicity data in this study. However, unblinded immunogenicity data will be made available to the DMC upon request to enable a benefit-risk assessment.
**Multiplicity**	No adjustment will be made for multiplicity.
**Sample Size and Power**	**Immunogenicity**:<br>The planned sample size is 1500 participants. Participants are to be randomly assigned in a 3:1 ratio to V114 or Prevnar 13™, respectively (resulting in approximately 1125 participants in the V114 group and approximately 375 participants in the Prevnar 13™ group). It is assumed that approximately 1013 participants in the V114 group and 337 in the Prevnar 13™ group will be evaluable for PP immunogenicity analyses at Day 30 (90% evaluability rate). There are no hypotheses to be evaluated, but Section 9.9.1 provides information about the expected variability of the OPA GMTs given the sample size.<br>**Safety:**<br>Section 9.9.2 provides information about the ability of this study to estimate the incidence of AEs within the V114 group.

9.2 Responsibility for Analyses/In-house Blinding

The statistical analysis of the data obtained from this study will be the responsibility of the Clinical Biostatistics department of the Sponsor.

This study will be conducted as a double-blind study under in-house blinding procedures. The official, final database will not be unblinded until medical/scientific review has been performed, protocol deviations have been identified, and data have been declared final and complete.

The Clinical Biostatistics department will generate the randomized allocation schedule(s) for study intervention assignment. Randomization will be implemented using an IRT.

Confidential

07Y9YV 054WRH

Blinding issues related to the planned interim analyses are described in Section 9.7.

9.3 Hypotheses/Estimation

Objectives of the study are stated in Section 3. This is an estimation study and no formal hypothesis testing will be performed.

9.4 Analysis Endpoints

Immunogenicity and safety analysis endpoints that will be summarized are listed below.

9.4.1 Immunogenicity Endpoints

A description of immunogenicity assessments is contained in Section 8.2.

Immune responses will be measured for each of the following serotypes contained in V114: 1, 3, 4, 5, 6A, 6B, 7F, 9V, 14, 18C, 19A, 19F, 22F, 23F, and 33F.

The primary immunogenicity endpoint is the serotype-specific OPA GMTs at 30 days postvaccination with V114 and Prevnar 13™ (Day 30).

The secondary immunogenicity analysis endpoints include:

- Serotype-specific IgG GMCs at 30 days postvaccination with V114 and Prevnar 13™ (Day 30)
- Serotype-specific OPA GMTs and IgG GMCs at 30 days postvaccination with PNEUMOVAX™23 (Month 7)
- Serotype-specific GMFR and proportion of participants with a ≥4-fold rise from prevaccination (Day 1) to 30 days postvaccination with V114 or Prevnar 13™ (Day 30) for both OPA and IgG responses
- Serotype-specific GMFR and proportion of participants with a ≥4-fold rise from prevaccination (Day 1) to 30 days postvaccination with PNEUMOVAX™23 (Month 7) for both OPA and IgG responses
- Serotype-specific GMFR and proportion of participants with a ≥4-fold rise from prevaccination with PNEUMOVAX™23 (Month 6) to 30 days postvaccination with PNEUMOVAX™23 (Month 7) for both OPA and IgG responses

9.4.2 Safety Endpoints

A description of safety measures is contained in Sections 8.3 and 8.4. The analysis of safety results is described in Section 9.6.2.

Confidential
07Y9YV 054WRH

Safety and tolerability will be assessed by clinical review of all relevant parameters including adverse events and postvaccination temperature measurements following V114 or Prevnar 13™ and following PNEUMOVAX™23 (separately).

The safety analysis endpoints include:

- Proportion of participants with solicited injection-site AEs (redness/erythema, swelling, tenderness/pain) from Day 1 through Day 5 postvaccination
- Proportion of participants with solicited systemic AEs (muscle pain/myalgia, joint pain/arthralgia, headache, tiredness/fatigue) from Day 1 through Day 14 postvaccination
- Proportions of participants with the broad AE categories consisting of any AE, a vaccine-related AE, a SAE, an AE which is both vaccine-related and serious, and discontinuation due to an AE, and the proportion of participants who died
- Proportion of participants with maximum temperature measurements meeting the Brighton Collaboration cut points from Day 1 through Day 5 postvaccination

9.5 Analysis Populations

9.5.1 Immunogenicity Analysis Populations

The Per-Protocol (PP) population will serve as the primary population for the analysis of immunogenicity data in this study. The PP population consists of all randomized participants without deviations from the protocol that may substantially affect the results of the immunogenicity endpoints. Potential deviations that may result in the exclusion of a participant from the PP population for all immunogenicity analyses include:

- Failure to receive any study vaccine at Visit 2 (Day 1)
- Failure to receive correct clinical material as per randomization schedule (ie, participants who were cross-treated)
- Receipt of a prohibited medication or prohibited vaccine prior to study vaccination

Additional potential deviations that may result in the exclusion of a participant from the PP population for specific immunogenicity analyses (depending on the time point) include:

- Failure to receive PNEUMOVAX™23
- Receipt of a prohibited medication or prohibited vaccine prior to a blood sample collection
- Collection of a blood sample outside of the prespecified window (as described in Section 1.3)

Confidential
07Y9YV 054WRH

The final determination on protocol deviations, and thereby the composition of the PP population, will be made prior to the final unblinding of the database. Participants will be included in the vaccination group to which they are randomized for the analysis of immunogenicity data using the PP population.

A supportive analysis using the Full Analysis Set (FAS) population will also be performed for the primary immunogenicity endpoint. The FAS population consists of all randomized participants who received at least 1 study vaccination and have at least 1 serology result. Participants will be included in the vaccination group to which they are randomized for the analysis of immunogenicity data using the FAS population.

9.5.2 Safety Analysis Populations

Safety analyses will be conducted in the All Participants as Treated (APaT) population, which consists of all randomized participants who received at least 1 dose of study vaccination. Participants will be included in the group corresponding to the study vaccination they actually received for the analysis of safety data using the APaT population. This will be the group to which they are randomized except for participants who take incorrect study vaccination; such participants will be included in the group corresponding to the study vaccination actually received.

At least 1 temperature measurement obtained subsequent to study intervention is required for inclusion in the analysis of temperature.

9.6 Statistical Methods

Statistical testing and inference for immunogenicity and safety analyses are described in Section 9.6.1 and Section 9.6.2, respectively. Section 9.6.3 describes how demographic and baseline characteristics will be summarized.

9.6.1 Statistical Methods for Immunogenicity Analyses

This section describes the statistical methods that address the primary and secondary immunogenicity objectives. Methods related to exploratory objectives will be described in the supplemental Statistical Analysis Plan.

Immunogenicity analyses will be conducted for each of the 15 pneumococcal serotypes in V114 separately. To address the primary immunogenicity objective, evaluation of the OPA GMTs at 30 days postvaccination with V114 or Prevnar 13™ (Day 30) will include descriptive summaries, and within-group 95% CIs will be calculated for each vaccination group. Point estimates for the OPA GMTs will be calculated by exponentiating the estimates of the mean of the natural log values. The within-group CIs will be derived by exponentiating the CIs of the mean of the natural log values based on the t-distribution.

A similar statistical approach will be used to evaluate the IgG GMCs at 30 days postvaccination with V114 or Prevnar 13™ (Day 30) and OPA GMTs and IgG GMCs at 30 days postvaccination with PNEUMOVAX™23 (Month 7).

Confidential

07Y9YV 054WRH

Descriptive statistics with point estimates and within-group 95% CIs will be provided for all other immunogenicity endpoints. For the continuous endpoints, a similar statistical approach will be used as described above for the primary endpoint. Point estimates will be calculated by exponentiating the estimates of the mean of the natural log values, and the within-group CIs will be derived by exponentiating the CIs of the mean of the natural log values based on the t-distribution. For the dichotomous endpoints, the within-group CIs will be calculated based on the exact method proposed by Clopper and Pearson [Clopper, C. J. and Pearson, E. S. 1934].

To address the exploratory immunogenicity objectives aiming to evaluate the OPA GMTs and the IgG GMCs 30 days postvaccination with V114 or Prevnar 13™ (Day 30) and at 30 days postvaccination with PNEUMOVAX™23 (Month 7) for participants administered V114 compared to participants administered Prevnar 13™, the serotype-specific OPA GMTs and IgG GMTs will be compared between vaccination groups through the estimation of serotype-specific OPA GMT ratios and IgG GMC ratios for each of the 15 serotypes in V114 at each of these time points. Estimation of the OPA GMT ratios and computation of the corresponding 95% CIs will be calculated using a constrained longitudinal data analysis (cLDA) method proposed by Liang and Zeger [Liang, K-Y and Zeger, S. L. 2000]. Details regarding the cLDA models will be included in the supplemental Statistical Analysis Plan.

Reverse Cumulative Distribution Curves for OPA titers and IgG concentrations will be graphically displayed by serotype at each of the following time points: Day 30 and Month 7. A detailed analysis strategy for immunogenicity endpoints is listed in Table 5.

Table 5 Analysis Strategy for Immunogenicity Variables

Endpoint/Variable (Description, Time Point)	Primary vs. Supportive Approach†	Statistical Method	Analysis Population	Missing Data Approach
**Primary Endpoint**				
OPA GMTs at Day 30	P	Descriptive Statistics (estimate, 95% CI)	PP	Missing data will not be imputed
	S		FAS	
**Secondary Endpoints**				
IgG GMCs at Day 30	P	Descriptive Statistics (estimate, 95% CI)	PP	Missing data will not be imputed
GMFR and % of participants who achieved a ≥4-fold rise from Day 1 to Day 30	P	Descriptive Statistics (estimate, 95% CI)	PP	Missing data will not be imputed
OPA GMTs and IgG GMCs at Month 7	P	Descriptive Statistics (estimate, 95% CI)	PP	Missing data will not be imputed
GMFR and % of participants who achieved a ≥4-fold rise from Day 1 to Month 7	P	Descriptive Statistics (estimate, 95% CI)	PP	Missing data will not be imputed
GMFR and % of participants who achieved a ≥4-fold rise from Month 6 to Month 7	P	Descriptive Statistics (estimate, 95% CI)	PP	Missing data will not be imputed

† P = primary approach; S = supportive approach.

CI = confidence interval; FAS = Full Analysis Set; GMC = Geometric Mean Concentration; GMFR = Geometric Mean Fold Rise; GMT = Geometric Mean Titer; IgG = immunoglobulin G; OPA = opsonophagocytic activity; PP = Per-Protocol

9.6.2 Statistical Methods for Safety Analyses

Safety and tolerability will be assessed by clinical review of all relevant parameters, including AEs and postvaccination temperature measurements.

The analysis strategy for safety parameters following each vaccination is summarized in Table 6. The proportion of participants with solicited injection-site AEs (redness/erythema, swelling, tenderness/pain from Day 1 to Day 5 postvaccination) and solicited systemic AEs (muscle pain/myalgia, joint pain/arthralgia, headache, tiredness/fatigue from Day 1 to Day 14 postvaccination) will be provided, along with the corresponding within-group 95% CIs (based on the exact binomial method proposed by Clopper and Pearson [Clopper, C. J. and Pearson, E. S. 1934]). In addition, the broad AE categories consisting of the proportion of

Confidential

participants with any AE, a vaccine-related AE, a SAE, an AE that is both vaccine-related and serious, discontinuation due to an AE, and the proportion of participants who died will be summarized in the same manner. The proportion of participants with maximum temperature measurements meeting the Brighton Collaboration cut points (from Day 1 through Day 5) will also will be provided, along with the corresponding within-group 95% CIs. Point estimates by vaccination group will be provided for all other safety parameters (specific AE terms and system organ class terms).

The analysis of safety parameters will be evaluated at 2 separate time points: (1) following administration of V114 or Prevnar 13™ and (2) following administration of PNEUMOVAX™23. Descriptive summaries of AEs following administration of V114 or Prevnar 13™ will include nonserious adverse events (NSAEs) within 14 days of vaccination and SAEs occurring Day 1 through Month 6 (prior to vaccination with PNEUMOVAX™23). Descriptive summaries of AEs following administration of PNEUMOVAX™23 will include NSAEs within 14 days of vaccination and SAEs occurring Month 6 (Day 1 relative to vaccination with PNEUMOVAX™23) through Month 7 (Day 30 relative to vaccination with PNEUMOVAX™23).

Safety analyses will be based on the observed data (ie, with no imputation of missing data).

Table 6 Analysis Strategy for Safety Parameters Following Each Vaccination

Safety Endpoint	95% CI for Within-Group Comparison	Descriptive Statistics
Injection-site redness/erythema (Days 1 to 5)†	X	X
Injection-site swelling (Days 1 to 5)†	X	X
Injection-site tenderness/pain (Days 1 to 5)†	X	X
Muscle pain/myalgia (Days 1 to 14)†	X	X
Joint pain/arthralgia (Days 1 to 14)†	X	X
Headache (Days 1 to 14)†	X	X
Tiredness/fatigue (Days 1 to 14)†	X	X
Any AE‡	X	X
Any vaccine-related AE‡	X	X
Any SAE‡	X	X
Any vaccine-related SAE‡	X	X
Discontinuation due to AE‡	X	X
Death‡	X	X
Maximum temperature measurements meeting the Brighton Collaboration cut points (Days 1 to 5)	X	X
Specific AEs by SOC and PT		X

† Includes solicited events only.
‡ These endpoints are broad AE categories. For example, descriptive statistics for the safety endpoint of "Any AE" will provide the number and percentage of participants with at least 1 AE.
AE = adverse event; CI = confidence interval; PT = preferred term; SAE = serious adverse event; SOC = system organ class; X = results will be provided.

Confidential
07Y9YV 054WRH

9.6.3 Demographic and Baseline Characteristics

The comparability of the vaccination groups for each relevant demographic and baseline characteristic will be assessed by the use of summary tables. No statistical hypothesis tests will be performed on these characteristics. The number and percentage of participants screened and randomized and the primary reasons for screening failure and discontinuation will be displayed. Demographic variables (eg, age, race, and gender), baseline characteristics, and prior and concomitant vaccinations and therapies will be summarized by vaccination group either by descriptive statistics or categorical tables.

9.7 Interim Analyses

A periodic review of safety and tolerability data across the adult V114 Phase 3 program will be conducted by an independent, unblinded, external DMC. A description of the structure, function, and guidelines for decision-making by the DMC, along with the timing and content of the safety reviews, will be outlined in the DMC charter. Information regarding the composition of the DMC is provided in Appendix 1. There are no plans to conduct an interim analysis of unblinded immunogenicity data in this study. However, unblinded immunogenicity data will be made available to the DMC upon request to enable a benefit-risk assessment.

Study enrollment is likely to be ongoing at the time of any interim analyses. Blinding to intervention assignment will be maintained at all investigational sites. The results of interim analyses will not be shared with the investigators prior to the completion of the study. Participant-level unblinding will be restricted to an external unblinded statistician performing the interim analysis.

The DMC will serve as the primary reviewer of the results of the safety interim analyses and will make recommendations for discontinuation of the study or protocol modifications to an executive committee of the Sponsor (see Appendix 1 for details on the Committees Structure for this study). If the DMC recommends modifications to the design of the protocol or discontinuation of the study, this executive committee (and potentially other limited Sponsor personnel) may be unblinded to results at the intervention level in order to act on these recommendations. The extent to which individuals are unblinded with respect to results of interim analyses will be documented by the external unblinded statistician. Additional logistical details will be provided in the DMC Charter.

Intervention-level results from the safety interim analysis will be provided by the external unblinded statistician to the DMC. Prior to final study unblinding, the external unblinded statistician will not be involved in any discussions regarding modifications to the protocol, statistical methods, identification of protocol deviations, or data validation efforts after the interim analyses.

9.8 Multiplicity

No adjustment will be made for multiplicity.

Confidential

07Y9YV 054WRH

9.9 Sample Size and Power Calculations

9.9.1 Sample Size and Power for Immunogenicity Analyses

This is a descriptive study. This study will randomize approximately 1125 participants into the V114 group and 375 participants into the Prevnar 13™ group. It is assumed that approximately 1013 participants in the V114 group and 337 in the Prevnar 13™ group will be evaluable for PP immunogenicity analyses at Day 30 (based on a 90% evaluability rate).

The width of the within-group 95% CIs for the serotype-specific OPA GMTs depend on the sample size, variability of the natural log concentrations, and the magnitude of the OPA GMT. In Table 7, 95% CIs for various hypothetical Day 30 OPA GMTs and various hypothetical standard deviation estimates for the natural log titers are displayed.

Table 7 Within-group 95% CIs for Varying Hypothetical OPA GMTs and Varying Standard Deviations

Standard Deviation of Natural Log Titers†	Serotype-specific OPA GMT†					
	500		1000		5000	
	V114	Prevnar 13™	V114	Prevnar 13™	V114	Prevnar 13™
1.5	(456, 548)	(426, 587)	(912, 1097)	(852, 1174)	(4559, 5485)	(4260, 5868)
2	(442, 566)	(404, 619)	(884, 1131)	(808, 1238)	(4421, 5655)	(4039, 6190)
2.5	(429, 583)	(383, 653)	(857, 1166)	(766, 1306)	(4287, 5832)	(3829, 6530)

Based on 1013 evaluable participants in the V114 group and 337 evaluable participants in the Prevnar 13™ group.
†The estimates of the standard deviation and OPA GMTs are representative of those observed in V114-006.
CI = confidence interval; GMT = Geometric Mean Tier; OPA = opsonophagocytic activity.

9.9.2 Sample Size and Power for Safety Analyses

The sample size was selected to achieve a reasonably sized safety database in this population exposed to V114. The probability of observing at least 1 SAE in this study depends on the number of participants vaccinated and the underlying incidence of participants with a SAE in the study population. Calculations below assume that 100% of the randomized participants will be evaluable for safety analyses. There is an 80% chance of observing at least 1 SAE among 1125 participants in the V114 group if the underlying incidence of a SAE is 0.14% (1 of every 700 participants receiving the vaccine). There is a 50% chance of observing at least 1 SAE among 1125 participants in the V114 group if the underlying incidence of a SAE is 0.06% (1 of every 1623 participants receiving the vaccine). If no SAEs are observed among the 1125 participants in the V114 group, this study will provide 97.5% confidence that the underlying percentage of participants with a SAE is <0.34% (1 in every 294 participants) in the V114 group.

9.10 Subgroup Analyses

Serotype-specific OPA GMTs and IgG GMCs at Day 30 and at Month 7 and their corresponding within-group 95% CIs will be calculated among participants enrolled at CAIH.

In addition, these endpoints will be evaluated in additional subgroups depending on the distribution of participants across the risk factors. Details will be provided in the supplemental Statistical Analysis Plan.

A similar approach will be used for subgroup analyses for safety endpoints (including summary of AEs and a summary of solicited AEs).

9.11 Compliance (Medication Adherence)

Given that participants will receive just a single dose of V114 or Prevnar 13™ and a single dose of PNEUMOVAX™23, compliance will not be calculated. However, the number and proportion of randomized participants receiving each vaccination will be summarized (Section 9.12).

9.12 Extent of Exposure

The extent of exposure will be summarized by the number and proportion of randomized participants administered V114 or Prevnar 13™ and the number and proportion of randomized participants administered PNEUMOVAX™23.

07Y9YV 054WRH Confidential

10 SUPPORTING DOCUMENTATION AND OPERATIONAL CONSIDERATIONS

10.1 Appendix 1: Regulatory, Ethical, and Study Oversight Considerations

10.1.1 Code of Conduct for Clinical Trials

Merck Sharp and Dohme Corp., a subsidiary of Merck & Co., Inc. (MSD)

Code of Conduct for Interventional Clinical Trials

I. Introduction

A. Purpose

MSD, through its subsidiaries, conducts clinical trials worldwide to evaluate the safety and effectiveness of our products. As such, we are committed to designing, implementing, conducting, analyzing and reporting these trials in compliance with the highest ethical and scientific standards. Protection of participants in clinical trials is the overriding concern in the design of clinical trials. In all cases, MSD clinical trials will be conducted in compliance with local and/or national regulations (eg, International Council for Harmonisation Good Clinical Practice [ICH-GCP]) and in accordance with the ethical principles that have their origin in the Declaration of Helsinki.

B. Scope

Highest ethical and scientific standards shall be endorsed for all clinical interventional investigations sponsored by MSD irrespective of the party (parties) employed for their execution (eg, contract research organizations, collaborative research efforts). This Code is not intended to apply to trials that are observational in nature, or which are retrospective. Further, this Code does not apply to investigator-initiated trials, which are not under the full control of MSD.

II. Scientific Issues

A. Trial Conduct

1. Trial Design

Except for pilot or estimation trials, clinical trial protocols will be hypothesis-driven to assess safety, efficacy, and/or pharmacokinetic or pharmacodynamic indices of MSD or comparator products. Alternatively, MSD may conduct outcomes research trials, trials to assess or validate various endpoint measures, or trials to determine patient preferences, etc.

The design (ie, participant population, duration, statistical power) must be adequate to address the specific purpose of the trial. Participants must meet protocol entry criteria to be enrolled in the trial.

2. Site Selection

MSD selects investigative sites based on medical expertise, access to appropriate participants, adequacy of facilities and staff, previous performance in clinical trials, as well as budgetary considerations. Prior to trial initiation, sites are evaluated by MSD personnel to assess the ability to successfully conduct the trial.

3. Site Monitoring/Scientific Integrity

Investigative trial sites are monitored to assess compliance with the trial protocol and general principles of Good Clinical Practice (GCP). MSD reviews clinical data for accuracy, completeness, and consistency. Data are verified versus source documentation according to standard operating procedures. Per MSD policies and procedures, if fraud, scientific/research misconduct, or serious GCP-noncompliance is suspected, the issues are investigated. When necessary, the clinical site will be closed, the responsible regulatory authorities and ethics review committees notified.

B. Publication and Authorship

Regardless of trial outcome, MSD commits to publish primary and secondary results of its registered trials of marketed products in which treatment is assigned, according to the prespecified plans for data analysis. To the extent scientifically appropriate, MSD seeks to publish the results of other analyses it conducts that are important to patients, physicians, and payers. Some early phase or pilot trials are intended to be hypothesis-generating rather

than hypothesis testing, in such cases, publication of results may not be appropriate since the trial may be underpowered and the analyses complicated by statistical issues such as multiplicity.

MSD's policy on authorship is consistent with the recommendations published by the International Committee of Medical Journal Editors (ICMJE). In summary, authorship should reflect significant contribution to the design and conduct of the trial, performance or interpretation of the analysis, and/or writing of the manuscript. All named authors must be able to defend the trial results and conclusions. MSD funding of a trial will be acknowledged in publications.

III. Participant Protection

A. Ethics Committee Review (Institutiona Review Board [IRB]/Independent Ethics Committee [IEC])

All clinical trials will be reviewed and approved by an IRB/IEC before being initiated at each site. Significant changes or revisions to the protocol will be approved by the ethics committee prior to implementation, except changes required urgently to protect participant safety that may be enacted in anticipation of ethics committee approval. For each site, the ethics committee and MSD will approve the participant informed consent form.

B. Safety

The guiding principle in decision-making in clinical trials is that participant welfare is of primary importance. Potential participants will be informed of the risks and benefits of, as well as alternatives to, trial participation. At a minimum, trial designs will take into account the local standard of care.

All participation in MSD clinical trials is voluntary. Participants enter the trial only after informed consent is obtained. Participants may withdraw from an MSD trial at any time, without any influence on their access to, or receipt of, medical care that may otherwise be available to them.

C. Confidentiality

MSD is committed to safeguarding participant confidentiality, to the greatest extent possible. Unless required by law, only the investigator, Sponsor (or representative), ethics committee, and/or regulatory authorities will have access to confidential medical records that might identify the participant by name.

D. Genomic Research

Genomic research will only be conducted in accordance with a protocol and informed consent authorized by an ethics committee.

IV. Financial Considerations

A. Payments to Investigators

Clinical trials are time- and labor-intensive. It is MSD's policy to compensate investigators (or the sponsoring institution) in a fair manner for the work performed in support of MSD trials. MSD does not pay incentives to enroll participants in its trials. However, when enrollment is particularly challenging, additional payments may be made to compensate for the time spent in extra recruiting efforts.

MSD does not pay for participant referrals. However, MSD may compensate referring physicians for time spent on chart review to identify potentially eligible participants.

B. Clinical Research Funding

Informed consent forms will disclose that the trial is sponsored by MSD and that the investigator or sponsoring institution is being paid or provided a grant for performing the trial. However, the local ethics committee may wish to alter the wording of the disclosure statement to be consistent with financial practices at that institution. As noted above, all publications resulting from MSD trials will indicate MSD as a source of funding.

C. Funding for Travel and Other Requests

Funding of travel by investigators and support staff (eg, to scientific meetings, investigator meetings, etc.) will be consistent with local guidelines and practices.

V. Investigator Commitment

Investigators will be expected to review MSD's Code of Conduct as an appendix to the trial protocol, and in signing the protocol, agree to support these ethical and scientific standards.

Confidential
07Y9YV 054WRH

10.1.2 Financial Disclosure

Financial Disclosure requirements are outlined in the US Food and Drug Administration Regulations, Financial Disclosure by Clinical Investigators (21 CFR Part 54). It is the Sponsor's responsibility to determine, based on these regulations, whether a request for Financial Disclosure information is required. It is the investigator's/subinvestigator's responsibility to comply with any such request.

The investigator/subinvestigator(s) agree, if requested by the Sponsor in accordance with 21 CFR Part 54, to provide his/her financial interests in and/or arrangements with the Sponsor to allow for the submission of complete and accurate certification and disclosure statements. The investigator/subinvestigator(s) further agree to provide this information on a Certification/Disclosure Form, commonly known as a financial disclosure form, provided by the Sponsor. The investigator/subinvestigator(s) also consent to the transmission of this information to the Sponsor in the United States for these purposes. This may involve the transmission of information to countries that do not have laws protecting personal data.

10.1.3 Data Protection

Participants will be assigned a unique identifier by the Sponsor. Any participant records or datasets that are transferred to the Sponsor will contain the identifier only; participant names or any information that would make the participant identifiable will not be transferred.

The participant must be informed that his/her personal study-related data will be used by the Sponsor in accordance with local data protection law. The level of disclosure must also be explained to the participant.

The participant must be informed that his/her medical records may be examined by Clinical Quality Assurance auditors or other authorized personnel appointed by the Sponsor, by appropriate IRB/IEC members, and by inspectors from regulatory authorities.

10.1.3.1 Confidentiality of Data

By signing this protocol, the investigator affirms to the Sponsor that information furnished to the investigator by the Sponsor will be maintained in confidence, and such information will be divulged to the IRB, IEC, or similar or expert committee; affiliated institution and employees, only under an appropriate understanding of confidentiality with such board or committee, affiliated institution and employees. Data generated by this study will be considered confidential by the investigator, except to the extent that it is included in a publication as provided in the Publications section of this protocol.

10.1.3.2 Confidentiality of Participant Records

By signing this protocol, the investigator agrees that the Sponsor (or Sponsor representative), IRB/IEC, or regulatory authority representatives may consult and/or copy study documents to verify worksheet/CRF report form data. By signing the consent form, the participant agrees to this process. If study documents will be photocopied during the process of verifying

Confidential
07Y9YV 054WRH

worksheet/CRF information, the participant will be identified by unique code only; full names/initials will be masked prior to transmission to the Sponsor.

By signing this protocol, the investigator agrees to treat all participant data used and disclosed in connection with this study in accordance with all applicable privacy laws, rules and regulations.

10.1.3.3 Confidentiality of IRB/IEC Information

The Sponsor is required to record the name and address of each IRB/IEC that reviews and approves this study. The Sponsor is also required to document that each IRB/IEC meets regulatory and ICH GCP requirements by requesting and maintaining records of the names and qualifications of the IRB/IEC members and to make these records available for regulatory agency review upon request by those agencies.

10.1.4 Committees Structure

10.1.4.1 External Data Monitoring Committee

To supplement the routine study monitoring outlined in this protocol, an external DMC will monitor the interim data from this study. The voting members of the committee are external to the Sponsor. The members of the DMC must not be involved with the study in any other way (eg, they cannot be study investigators) and must have no competing interests that could affect their roles with respect to the study.

The DMC will make recommendations to the EOC regarding steps to ensure both participant safety and the continued ethical integrity of the study. Also, the DMC will review interim study results, consider the overall risk and benefit to study participants (Section 9.7) and recommend to the EOC whether the study should continue in accordance with the protocol.

Specific details regarding composition, responsibilities, and governance, including the roles and responsibilities of the various members and the Sponsor protocol team; meeting facilitation; the study governance structure; and requirements for and proper documentation of DMC reports, minutes, and recommendations will be described in the DMC charter that is reviewed and approved by all the DMC members.

10.1.4.2 Executive Oversight Committee

The Executive Oversight Committee (EOC) is comprised of members of Sponsor Senior Management. The EOC will receive and decide upon any recommendations made by the external DMC regarding the study.

10.1.4.3 Scientific Advisory Committee

This study was developed in collaboration with a Scientific Advisory Committee (SAC). The SAC is comprised of both Sponsor and non-Sponsor scientific experts who provide input with respect to study design, interpretation of study results, and subsequent peer-reviewed scientific publications.

Confidential
07Y9YV 054WRH

10.1.5 Publication Policy

The results of this study may be published or presented at scientific meetings. The Sponsor will comply with the requirements for publication of study results. In accordance with standard editorial and ethical practice, the Sponsor will generally support publication of multicenter studies only in their entirety and not as individual site data. In this case, a coordinating investigator will be designated by mutual agreement.

If publication activity is not directed by the Sponsor, the investigator agrees to submit all manuscripts or abstracts to the Sponsor before submission. This allows the Sponsor to protect proprietary information and to provide comments.

Authorship will be determined by mutual agreement and in line with International Committee of Medical Journal Editors authorship requirements.

10.1.6 Compliance with Study Registration and Results Posting Requirements

Under the terms of the Food and Drug Administration Amendments Act (FDAAA) of 2007 and the European Medicines Agency (EMA) clinical trial Directive 2001/20/EC, the Sponsor of the study is solely responsible for determining whether the study and its results are subject to the requirements for submission to http://www.clinicaltrials.gov, www.clinicaltrialsregister.eu or other local registries. MSD, as Sponsor of this study, will review this protocol and submit the information necessary to fulfill these requirements. MSD entries are not limited to FDAAA or the EMA clinical trial directive mandated trials. Information posted will allow participants to identify potentially appropriate studies for their disease conditions and pursue participation by calling a central contact number for further information on appropriate study locations and study site contact information.

By signing this protocol, the investigator acknowledges that the statutory obligations under FDAAA, the EMA clinical trials directive or other locally mandated registries are that of the Sponsor and agrees not to submit any information about this study or its results to those registries.

10.1.7 Compliance with Law, Audit, and Debarment

By signing this protocol, the investigator agrees to conduct the study in an efficient and diligent manner and in conformance with this protocol; generally accepted standards of GCP (eg, International Conference on Harmonization of Technical Requirements for Registration of Pharmaceuticals for Human Use GCP: Consolidated Guideline and other generally accepted standards of good clinical practice); and all applicable federal, state and local laws, rules and regulations relating to the conduct of the clinical study. The Code of Conduct, a collection of goals and considerations that govern the ethical and scientific conduct of clinical investigations sponsored by MSD, is provided in this appendix under the Code of Conduct for Clinical Studies.

Confidential
07Y9YV 054WRH

The investigator agrees not to seek reimbursement from participants, their insurance providers, or from government programs for procedures included as part of the study reimbursed to the investigator by the Sponsor.

The investigator will promptly inform the Sponsor of any regulatory authority inspection conducted for this study.

The investigator agrees to provide the Sponsor with relevant information from inspection observations/findings to allow the Sponsor to assist in responding to any citations resulting from regulatory authority inspection and will provide the Sponsor with a copy of the proposed response for consultation before submission to the regulatory authority.

Persons debarred from conducting or working on clinical studies by any court or regulatory authority will not be allowed to conduct or work on this Sponsor's studies. The investigator will immediately disclose in writing to the Sponsor if any person who is involved in conducting the study is debarred or if any proceeding for debarment is pending or, to the best of the investigator's knowledge, threatened.

10.1.8 Data Quality Assurance

All participant data relating to the study will be recorded on printed or electronic CRF unless transmitted to the Sponsor or designee electronically (eg, laboratory data). The investigator or qualified designee is responsible for verifying that data entries are accurate and correct by physically or electronically signing the CRF.

Detailed information regarding Data Management procedures for this protocol will be provided separately.

The investigator must maintain accurate documentation (source data) that supports the information entered in the CRF.

The investigator must permit study-related monitoring, audits, IRB/IEC review, and regulatory agency inspections and provide direct access to source data documents.

Study documentation will be promptly and fully disclosed to the Sponsor by the investigator upon request and also shall be made available at the study site upon request for inspection, copying, review, and audit at reasonable times by representatives of the Sponsor or any regulatory authorities. The investigator agrees to promptly take any reasonable steps that are requested by the Sponsor or any regulatory authorities as a result of an audit or inspection to cure deficiencies in the study documentation and worksheets/CRFs.

The Sponsor or designee is responsible for the data management of this study including quality checking of the data.

Study monitors will perform ongoing source data review and verification to confirm that data entered into the CRF by authorized site personnel are accurate, complete, and verifiable from source documents; that the safety and rights of participants are being protected; and that the

Confidential
07Y9YV 054WRH

study is being conducted in accordance with the currently approved protocol and any other study agreements, ICH GCP, and all applicable regulatory requirements.

Records and documents, including signed ICF, pertaining to the conduct of this study must be retained by the investigator for 15 years after study completion unless local regulations or institutional policies require a longer retention period. No records may be destroyed during the retention period without the written approval of the Sponsor. No records may be transferred to another location or party without written notification to the Sponsor.

10.1.9 Source Documents

Source documents provide evidence for the existence of the participant and substantiate the integrity of the data collected. Source documents are filed at the investigator's site.

Data reported on the CRF or entered in the eCRF that are transcribed from source documents must be consistent with the source documents or the discrepancies must be explained. The investigator may need to request previous medical records or transfer records, depending on the study. Also, current medical records must be available.

10.1.10 Study and Site Closure

The Sponsor or its designee may stop the study or study site participation in the study for medical, safety, regulatory, administrative, or other reasons consistent with applicable laws, regulations, and GCP.

In the event the Sponsor prematurely terminates a particular study site, the Sponsor will promptly notify that study site's IRB/IEC.

Confidential
07Y9YV 054WRH

10.2 Appendix 2: Clinical Laboratory Tests

- The tests detailed in Table 8 will be performed according to local requirements.
- Protocol-specific requirements for inclusion or exclusion of participants are detailed in Section 5 of the protocol.
- Additional tests may be performed at any time during the study as determined necessary by the investigator or required by local regulations.

Table 8 Protocol-required Safety Laboratory Assessments

Laboratory Assessments	Parameters
Other Screening Tests	Serum or urine β-hCG pregnancy test (as needed for WOCBP)
	**Disease-specific laboratory assessments:**<br>Diabetes mellitus: Hemoglobin A1c<br>Chronic liver disease: AST, ALT, total bilirubin, PT/INR, albumin, platelets

ALT = alanine aminotransferase; AST = aspartate aminotransferase; β-hCG = β human chorionic gonadotropin; INR = international normalized ratio; PT = prothrombin time; WOCBP = woman/women of childbearing potential.

07Y9YV 054WRH
Confidential

10.3 Appendix 3: Adverse Events: Definitions and Procedures for Recording, Evaluating, Follow-up, and Reporting

10.3.1 Definition of AE

AE definition

- An AE is any untoward medical occurrence in a clinical study participant, temporally associated with the use of study intervention, whether or not considered related to the study intervention.
- NOTE: An AE can therefore be any unfavorable and unintended sign (including an abnormal laboratory finding), symptom, or disease (new or exacerbated) temporally associated with the use of a study intervention.
- NOTE: For purposes of AE definition, study intervention (also referred to as Sponsor's product) includes any pharmaceutical product, biological product, vaccine, device, diagnostic agent, or protocol specified procedure whether investigational (including placebo or active comparator product) or marketed, manufactured by, licensed by, provided by, or distributed by the Sponsor for human use in this study.

Events meeting the AE definition

- Any abnormal laboratory test results (hematology, clinical chemistry, or urinalysis) or other safety assessments (eg, ECG, radiological scans, vital signs measurements), including those that worsen from baseline, or are considered clinically significant in the medical and scientific judgment of the investigator.
- Exacerbation of a chronic or intermittent pre-existing condition including either an increase in frequency and/or intensity of the condition.
- New conditions detected or diagnosed after study intervention administration even though it may have been present before the start of the study.
- Signs, symptoms, or the clinical sequelae of a suspected drug-drug interaction.
- Signs, symptoms, or the clinical sequelae of a suspected overdose of either study intervention or a concomitant medication.
- For all reports of overdose (whether accidental or intentional) with an associated AE, the AE term should reflect the clinical symptoms or abnormal test result. An overdose without any associated clinical symptoms or abnormal laboratory results is reported using the terminology "accidental or intentional overdose without adverse effect."
- Any new cancer or progression of existing cancer.

Confidential
07Y9YV 054WRH

Events NOT meeting the AE definition

- Medical or surgical procedure (eg, endoscopy, appendectomy): the condition that leads to the procedure is the AE.
- Situations in which an untoward medical occurrence did not occur (social and/or convenience admission to a hospital).
- Anticipated day-to-day fluctuations of pre-existing disease(s) or condition(s) present or detected at the start of the study that do not worsen.
- Surgery planned prior to informed consent to treat a pre-existing condition that has not worsened.
- Refer to Section 8.4.7 for protocol-specific exceptions.

10.3.2 Definition of SAE

If an event is not an AE per definition above, then it cannot be an SAE even if serious conditions are met.

An SAE is defined as any untoward medical occurrence that, at any dose:

a. **Results in death**

b. **Is life-threatening**

- The term "life-threatening" in the definition of "serious" refers to an event in which the participant was at risk of death at the time of the event. It does not refer to an event, which hypothetically might have caused death, if it were more severe.

c. **Requires inpatient hospitalization or prolongation of existing hospitalization**

- Hospitalization is defined as an inpatient admission, regardless of length of stay, even if the hospitalization is a precautionary measure for continued observation.
(Note: Hospitalization for an elective procedure to treat a pre-existing condition that has not worsened is not an SAE. A pre-existing condition is a clinical condition that is diagnosed prior to the use of an MSD product and is documented in the participant's medical history.

d. **Results in persistent or significant disability/incapacity**

- The term disability means a substantial disruption of a person's ability to conduct normal life functions.
- This definition is not intended to include experiences of relatively minor medical significance such as uncomplicated headache, nausea, vomiting, diarrhea, influenza, and accidental trauma (eg, sprained ankle) that may interfere with or prevent everyday life functions but do not constitute a substantial disruption.

Confidential
07Y9YV 054WRH

e. **Is a congenital anomaly/birth defect**

- In offspring of participant taking the product regardless of time to diagnosis.

f. **Other important medical events**

- Medical or scientific judgment should be exercised in deciding whether SAE reporting is appropriate in other situations such as important medical events that may not be immediately life-threatening or result in death or hospitalization but may jeopardize the participant or may require medical or surgical intervention to prevent 1 of the other outcomes listed in the above definition. These events should usually be considered serious.

  Examples of such events include invasive or malignant cancers, intensive treatment in an emergency room or at home for allergic bronchospasm, blood dyscrasias or convulsions that do not result in hospitalization, or development of drug dependency or drug abuse.

10.3.3 Additional Events Reported

Additional events that require reporting

In addition to the above criteria, AEs meeting either of the below criteria, although not serious per ICH definition, are reportable to the Sponsor.

- Is a cancer
- Is associated with an overdose

10.3.4 Recording AE and SAE

AE and SAE recording

- When an AE/SAE occurs, it is the responsibility of the investigator to review all documentation (eg, hospital progress notes, laboratory, and diagnostics reports) related to the event.
- The investigator will record all relevant AE/SAE information on the AE CRFs/worksheets at each examination.
- It is not acceptable for the investigator to send photocopies of the participant's medical records to the Sponsor in lieu of completion of the AE CRF page.
- There may be instances when copies of medical records for certain cases are requested by the Sponsor. In this case, all participant identifiers, with the exception of the participant number, will be blinded on the copies of the medical records before submission to the Sponsor.

Confidential
07Y9YV 054WRH

- The investigator will attempt to establish a diagnosis of the event based on signs, symptoms, and/or other clinical information. In such cases, the diagnosis (not the individual signs/symptoms) will be documented as the AE/SAE.

Assessment of Intensity/Toxicity

- An event is defined as "serious" when it meets at least 1 of the predefined outcomes as described in the definition of an SAE, not when it is rated as severe.
- The investigator will make an assessment of intensity for each AE and SAE (and other reportable safety event) reported during the study and assign it to 1 of the following categories:
  - Mild: An event that is easily tolerated by the participant, causing minimal discomfort and not interfering with everyday activities (for pediatric studies, awareness of symptoms, but easily tolerated).
  - Moderate: An event that causes sufficient discomfort to interfere with normal everyday activities (for pediatric studies definitely acting like something is wrong).
  - Severe: An event that prevents normal everyday activities. An AE that is assessed as severe should not be confused with an SAE. Severe is a category utilized for rating the intensity of an event; and both AE and SAE can be assessed as severe (for pediatric studies, extremely distressed or unable to do usual activities).
- Injection-site redness/erythema or swelling from the day of vaccination through Day 5 postvaccination will be evaluated by maximum size.
- The investigator will make an assessment of toxicity for each AE and SAE (and other reportable event) reported during the study. A toxicity grade will be assigned to injection-site AEs, specific systemic AEs, other systemic AEs, and vital sign (temperature) AEs as shown in the following tables. The toxicity grading scales used in this study are adapted from the "FDA Guidance for Industry: Toxicity Grading Scale for Healthy Adult and Adolescent Volunteers Enrolled in Preventive Vaccine Clinical Trials, September 2007."

Confidential
07Y9YV 054WRH

Injection-Site AE Toxicity Grading Scale

Injection Site Reaction to Study Vaccine/Placebo[a]	Grade 1	Grade 2	Grade 3	Grade 4
**Injection-site AEs occurring Days 1 through 5 following receipt of study vaccine/placebo**				
Pain/Tenderness	Does not interfere with activity	Repeated use of non-narcotic pain reliever >24 hours or interferes with activity	Any use of narcotic pain reliever or prevents daily activity	ER visit or hospitalization
Erythema/Redness	Size measured as B	Size measured as C or D	Size measured as E→	Necrosis or exfoliative dermatitis or results in ER visit or hospitalization
Induration/Swelling	Size measured as B	Size measured as C or D	Size measured as E→	Necrosis or ER visit or hospitalization
Other	Does not interfere with activity	Repeated use of non-narcotic pain reliever >24 hours or interferes with activity	Any use of narcotic pain reliever or prevents daily activity	ER visit or hospitalization
**Any injection-site reaction that begins ≥ 6 days after receipt of study vaccine/placebo**				
Pain/tenderness<br>Erythema/Redness<br>Induration/Swelling<br>Other	Does not interfere with activity	Repeated use of non-narcotic pain reliever >24 hours or interferes with activity	Any use of narcotic pain reliever or prevents daily activity	ER visit or hospitalization

Abbreviations: AE = adverse event; ER = emergency room; eVRC = electronic Vaccine Report Card; SAE = serious adverse event

[a] Based upon information provided by the patient on the eVRC and verbally during VRC review. Erythema/Redness and Induration/Swelling are specific injection-site AEs with size designations of letters A through E→, based upon a graphic in the eVRC. Size A is not assigned a toxicity grade; however, injection-site AEs that measure size A should be reported as adverse experiences. If the participant has an ER visit or is hospitalized for any injection-site AE, that AE is to be assigned a toxicity grade of 4, regardless of the size measured.

Specific Systemic AE Toxicity Grading Scale

Systemic (General)	Mild (Grade 1)	Moderate (Grade 2)	Severe (Grade 3)	Potentially Life Threatening (Grade 4)
Headache	No interference with activity	Repeated use of non-narcotic pain reliever >24 hours or some interference with activity	Significant; any use of narcotic pain reliever or prevents daily activity	ER visit or hospitalization
Fatigue	No interference with activity	Some interference with activity	Significant; prevents daily activity	ER visit or hospitalization
Myalgia	No interference with activity	Some interference with activity	Significant; prevents daily activity	ER visit or hospitalization

Abbreviations: ER = emergency room

Confidential
07Y9YV 054WRH

Other Systemic AE Toxicity Grading Scale

Systemic Illness[a]	Mild (Grade 1)	Moderate (Grade 2)	Severe (Grade 3)	Potentially Life Threatening (Grade 4)[b]
Illness or clinical AE (as defined according to applicable regulations)	No interference with activity	Some interference with activity not requiring medical intervention	Prevents daily activity and required medical intervention	ER visit or hospitalization

Abbreviations: ER = emergency room; eVRC = electronic Vaccine Report Card; SAE = serious adverse event

[a] Based upon information provided by the patient on the eVRC and verbally during the eVRC review during the primary safety follow-up period. For SAEs reported beyond the primary safety follow-up period, grading will be based upon the initial report and/or follow-up of the event.

[b] AEs resulting in death will be assessed as Grade 4

Vital Sign (Temperature) Toxicity Grading Scale

Vital Signs[a]	Mild (Grade 1)	Moderate (Grade 2)	Severe (Grade 3)	Potentially Life Threatening (Grade 4)
Fever (°C) [b]<br>(°F) [b]	38.0 to 38.4<br>100.4 to 101.1	38.5 to 38.9<br>101.2 to 102.0	39.0 to 40.0<br>102.1 to 104.0	>40.0<br>>104.0

[a] Participant should be at rest for all vital sign requirements

[b] Oral temperature; no recent hot or cold beverages or smoking

Assessment of causality

- Did the Sponsor’s product cause the AE?
- The determination of the likelihood that the Sponsor’s product caused the AE will be provided by an investigator who is a qualified physician. The investigator’s signed/dated initials on the source document or worksheet that supports the causality noted on the AE form, ensures that a medically qualified assessment of causality was done. This initialled document must be retained for the required regulatory time frame. The criteria below are intended as reference guidelines to assist the investigator in assessing the likelihood of a relationship between the test product and the AE based upon the available information.
- **The following components are to be used to assess the relationship between the Sponsor’s product and the AE;** the greater the correlation with the components and their respective elements (in number and/or intensity), the more likely the Sponsor’s product caused the AE:
  - **Exposure:** Is there evidence that the participant was actually exposed to the Sponsor’s product such as: reliable history, acceptable compliance assessment (diary, etc.), seroconversion or identification of vaccine virus in bodily specimen?
  - **Time Course:** Did the AE follow in a reasonable temporal sequence from administration of the Sponsor’s product? Is the time of onset of the AE compatible with a vaccine-induced effect?

Confidential
07Y9YV 054WRH

- **Likely Cause:** Is the AE not reasonably explained by another etiology such as underlying disease, other drug(s)/vaccine(s), or other host or environmental factors?
- **Rechallenge:** Was the participant re-exposed to the Sponsor's product in the study?
- If yes, did the AE recur or worsen?
- If yes, this is a positive rechallenge.
- If no, this is a negative rechallenge.

(Note: This criterion is not applicable if: (1) the initial AE resulted in death or permanent disability, or (2) the study is a single-dose vaccine study); or (3) Sponsor's product(s) is/are used only 1 time.)

NOTE: IF A RECHALLENGE IS PLANNED FOR AN AE THAT WAS SERIOUS AND MAY HAVE BEEN CAUSED BY THE SPONSOR'S PRODUCT, OR IF RE-EXPOSURE TO THE SPONSOR'S PRODUCT POSES ADDITIONAL POTENTIAL SIGNIFICANT RISK TO THE PARTICIPANT THEN THE RECHALLENGE MUST BE APPROVED IN ADVANCE BY THE SPONSOR CLINICAL DIRECTOR, AND IF REQUIRED, THE IRB/IEC.

- **Consistency with study intervention profile:** Is the clinical/pathological presentation of the AE consistent with previous knowledge regarding the Sponsor's product or drug class pharmacology or toxicology?
- The assessment of relationship will be reported on the CRFs /worksheets by an investigator who is a qualified physician according to his/her best clinical judgment, including consideration of the above elements.
- Use the following scale of criteria as guidance (not all criteria must be present to be indicative of a Sponsor's product relationship).
- Yes, there is a reasonable possibility of Sponsor's product relationship:
- There is evidence of exposure to the Sponsor's product. The temporal sequence of the AE onset relative to the administration of the Sponsor's product is reasonable. The AE is more likely explained by the Sponsor's product than by another cause.
- No, there is not a reasonable possibility of Sponsor's product relationship:
- Participant did not receive the Sponsor's product OR temporal sequence of the AE onset relative to administration of the Sponsor's product is not reasonable OR the AE is more likely explained by another cause than the Sponsor's product. (Also entered for a participant with overdose without an associated AE.)

• For each AE/SAE, the investigator must document in the medical notes that he/she has reviewed the AE/SAE and has provided an assessment of causality.

• There may be situations in which an SAE has occurred and the investigator has minimal information to include in the initial report to the Sponsor. However, it is very important that the investigator always make an assessment of causality for every event before the initial transmission of the SAE data to the Sponsor.

Confidential

07Y9YV 054WRH

- The investigator may change his/her opinion of causality in light of follow-up information and send an SAE follow-up report with the updated causality assessment.
- The causality assessment is 1 of the criteria used when determining regulatory reporting requirements.

Follow-up of AE and SAE

- The investigator is obligated to perform or arrange for the conduct of supplemental measurements and/or evaluations as medically indicated or as requested by Sponsor to elucidate the nature and/or causality of the AE or SAE as fully as possible. This may include additional laboratory tests or investigations, histopathological examinations, or consultation with other health care professionals.
- New or updated information will be recorded in the CRF.
- The investigator will submit any updated SAE data to the Sponsor within 24 hours of receipt of the information.

10.3.5 Reporting of AE, SAE, and Other Reportable Safety Events to the Sponsor

AE, SAE, and other reportable safety event reporting to Sponsor via electronic data collection tool

- The primary mechanism for reporting to the Sponsor will be the electronic data collection (EDC) tool.
    - Electronic reporting procedures can be found in the EDC data entry guidelines (or equivalent).
    - If the electronic system is unavailable for more than 24 hours, then the site will use the paper AE Reporting form.
        - Reference Section 8.4.1 for reporting time requirements.
- The site will enter the SAE data into the electronic system as soon as it becomes available.
- After the study is completed at a given site, the EDC tool will be taken off-line to prevent the entry of new data or changes to existing data.
- If a site receives a report of a new SAE from a study participant or receives updated data on a previously reported SAE after the EDC tool has been taken off-line, then the site can report this information on a paper SAE form or by telephone (see next section).
- Contacts for SAE reporting can be found in the Investigator Study File Binder (or equivalent).

Confidential

07Y9YV 054WRH

SAE reporting to the Sponsor via paper CRF

- If the EDC tool is not operational, facsimile transmission or secure e-mail of the SAE paper CRF is the preferred method to transmit this information to the Sponsor.
- In rare circumstances and in the absence of facsimile equipment, notification by telephone is acceptable with a copy of the SAE data collection tool sent by overnight mail or courier service.
- Initial notification via telephone does not replace the need for the investigator to complete and sign the SAE CRF pages within the designated reporting time frames.
- Contacts and instructions for SAE reporting and paper reporting procedures can be found in the Investigator Study File Binder (or equivalent).

Confidential
07Y9YV 054WRH

10.4 Appendix 4: Medical Device Incidents: Definition and Procedures for Recording, Evaluating, Follow-up, and Reporting

Not applicable.

Confidential

10.5 Appendix 5: Contraceptive Guidance and Pregnancy Testing

10.5.1 Definitions

Women of Childbearing Potential (WOCBP)

A woman is considered fertile following menarche and until becoming postmenopausal unless permanently sterile.

Women in the following categories are not considered WOCBP:

- Premenarchal
- Premenopausal female with 1 of the following:
    - Documented hysterectomy
    - Documented bilateral salpingectomy
    - Documented bilateral oophorectomy

Note: Documentation can come from the site personnel's review of the participant's medical records, medical examination, or medical history interview.

- Postmenopausal female
    - A postmenopausal state is defined as no menses for 12 months without an alternative medical cause.
- A high follicle stimulating hormone (FSH) level in the postmenopausal range may be used to confirm a postmenopausal state in women not using hormonal contraception or hormone replacement therapy (HRT). However, in the absence of 12 months of amenorrhea, confirmation with 2 FSH measurements in the postmenopausal range is required.
    - Females on HRT and whose menopausal status is in doubt will be required to use 1 of the nonhormonal highly effective contraception methods if they wish to continue their HRT during the study. Otherwise, they must discontinue HRT to allow confirmation of postmenopausal status before study enrollment.

Confidential

10.5.2 Contraception Requirements

Female Participants

Female participants of childbearing potential are eligible to participate if they agree to use 1 of the contraception methods described in Table 9 consistently and correctly during the protocol-defined time frame in Section 5.1.

Table 9 Contraceptive Methods

**Acceptable Contraceptive Methods**<br>*Failure rate of >1% per year when used consistently and correctly.*
• Male or female condom with or without spermicide<br>• Cervical cap, diaphragm or sponge with spermicide
**Highly Effective Contraceptive Methods That Are User Dependent**[a]<br>*Failure rate of <1% per year when used consistently and correctly.*
• Combined (estrogen- and progestogen- containing) hormonal contraception[b]<br>○ Oral<br>○ Intravaginal<br>○ Transdermal<br>○ Injectable
• Progestogen only hormonal contraception[b,c]<br>○ Oral<br>○ Injectable
**Highly Effective Methods That Have Low User Dependency**<br>*Failure rate of <1% per year when used consistently and correctly.*
• Progestogen- only contraceptive implant[b]<br>• Intrauterine hormone-releasing system (IUS)[b]<br>• Intrauterine device (IUD)<br>• Bilateral tubal occlusion
• Vasectomized partner<br>A vasectomized partner is a highly effective contraception method provided that the partner is the sole male sexual partner of the WOCBP and the absence of sperm has been confirmed. If not, an additional highly effective method of contraception should be used.
• Sexual abstinence<br>Sexual abstinence is considered a highly effective method only if defined as refraining from heterosexual intercourse during the entire period of risk associated with the study intervention. The reliability of sexual abstinence needs to be evaluated in relation to the duration of the study and the preferred and usual lifestyle of the participant.
Notes:<br>Use should be consistent with local regulations regarding the use of contraceptive methods for participants of clinical studies.<br>[a] Typical use failure rates are higher than perfect-use failure rates (ie, when used consistently and correctly).<br>[b] If locally required, in accordance with Clinical Trial Facilitation Group (CTFG) guidelines, acceptable hormonal contraceptives are limited to those which inhibit ovulation.

07Y9YV 054WRH Confidential

10.5.3 Pregnancy Testing

WOCBP should only be included after a negative highly sensitive urine or serum pregnancy test.

Pregnancy testing will be performed whenever an expected menstrual cycle is missed or when pregnancy is otherwise suspected.

10.6 Appendix 6: Collection and Management of Specimens for Future Biomedical Research (Does not apply to CAIH participants)

1. Definitions

- Biomarker: A biological molecule found in blood, other body fluids, or tissues that is a sign of a normal or abnormal process or of a condition or disease. A biomarker may be used to see how well the body responds to a treatment for a disease or condition.
- Pharmacogenomics: The investigation of variations of DNA and RNA characteristics as related to drug/vaccine response.[2]
- Pharmacogenetics: A subset of pharmacogenomics, pharmacogenetics is the influence of variations in DNA sequence on drug/vaccine response.[2]
- DNA: Deoxyribonucleic acid.
- RNA: Ribonucleic acid.

2. Scope of Future Biomedical Research

The specimens consented and/or collected in this study as outlined in Section 8.8 will be used in various experiments to understand:

- The biology of how drugs/vaccines work
- Biomarkers responsible for how a drug/vaccine enters and is removed by the body
- Other pathways drugs/vaccines may interact with
- The biology of disease

The specimen(s) may be used for future assay development and/or drug/vaccine development.

It is now well recognized that information obtained from studying and testing clinical specimens offers unique opportunities to enhance our understanding of how individuals respond to drugs/vaccines, enhance our understanding of human disease and ultimately improve public health through development of novel treatments targeted to populations with the greatest need. All specimens will be used by the Sponsor or those working for or with the Sponsor.

3. Summary of Procedures for Future Biomedical Research.

- Participants for Enrollment
- All participants enrolled in the clinical study study who are not Native American participants enrolled via the CAIH will be considered for enrollment in the future biomedical research substudy
- Informed Consent
- Informed consent for specimens (ie, DNA, RNA, protein, etc.) will be obtained during screening for protocol enrollment from all participants or legal guardians, at a study visit by the investigator or his or her designate. Informed consent for future biomedical research should be presented to the participants on the visit designated in the SoA. If delayed, present consent at next possible Participant Visit. Consent forms signed by the participant will be kept at the clinical study site under secure storage for regulatory reasons.
- A template of each study site's approved informed consent will be stored in the Sponsor's clinical document repository.
- eCRF Documentation for Future Biomedical Research Specimens
- Documentation of participant consent for future biomedical research will be captured in the eCRFs. Any specimens for which such an informed consent cannot be verified will be destroyed.
- Future Biomedical Research Specimen(s)
- Collection of specimens for future biomedical research will be performed as outlined in the SoA. In general, if additional blood specimens are being collected for future biomedical research, these will usually be obtained at a time when the participant is having blood drawn for other study purposes.

4. Confidential Participant Information for Future Biomedical Research

In order to optimize the research that can be conducted with future biomedical research specimens, it is critical to link participant' clinical information with future test results. In fact little or no research can be conducted without connecting the clinical study data to the specimen. The clinical data allow specific analyses to be conducted. Knowing participant characteristics like gender, age, medical history and treatment outcomes are critical to understanding clinical context of analytical results.

To maintain privacy of information collected from specimens obtained for future biomedical research, the Sponsor has developed secure policies and procedures. All specimens will be single-coded per ICH E15 guidelines as described below.

At the clinical study site, unique codes will be placed on the future biomedical research specimens. This code is a random number which does not contain any personally identifying information embedded within it. The link (or key) between participant identifiers and this unique code will be held at the study site. No personal identifiers will appear on the specimen tube.

5. Biorepository Specimen Usage

Specimens obtained for the Sponsor will be used for analyses using good scientific practices. Analyses utilizing the future biomedical research specimens may be performed by the Sponsor, or an additional third party (eg, a university investigator) designated by the Sponsor. The investigator conducting the analysis will follow the Sponsor's privacy and confidentiality requirements. Any contracted third party analyses will conform to the specific scope of analysis outlined in this substudy. Future biomedical research specimens remaining with the third party after specific analysis is performed will be reported to the Sponsor.

6. Withdrawal From Future Biomedical Research

Participants may withdraw their consent for future biomedical research and ask that their biospecimens not be used for future biomedical research. Participants may withdraw consent at any time by contacting the principal investigator for the main study. If medical records for the main study are still available, the investigator will contact the Sponsor using the designated mailbox (clinical.specimen.management@merck.com). Subsequently, the participant's specimens will be flagged in the biorepository and restricted to main study use only. If specimens were collected from study participants specifically for future biomedical research, these specimens will be removed from the biorepository and destroyed. Documentation will be sent to the investigator confirming withdrawal and/or destruction, if applicable. It is the responsibility of the investigator to inform the participant of completion of the withdrawal and/or destruction, if applicable. Any analyses in progress at the time of request for withdrawal/destruction or already performed prior to the request being received by the Sponsor will continue to be used as part of the overall research study data and results. No new analyses would be generated after the request is received.

In the event that the medical records for the main study are no longer available (eg, if the investigator is no longer required by regulatory authorities to retain the main study records) or the specimens have been completely anonymized, there will no longer be a link between the participant's personal information and their specimens. In this situation, the request for withdrawal of consent and/or destruction cannot be processed.

7. Retention of Specimens

Future biomedical research specimens will be stored in the biorepository for potential analysis for up to 20 years from the end of the main study. Specimens may be stored for longer if a regulatory or governmental authority has active questions that are being answered. In this special circumstance, specimens will be stored until these questions have been adequately addressed.

Specimens from the study site will be shipped to a central laboratory and then shipped to the Sponsor-designated biorepository. If a central laboratory is not utilized in a particular study, the study site will ship directly to the Sponsor-designated biorepository. The specimens will be stored under strict supervision in a limited access facility which operates to assure the integrity of the specimens. Specimens will be destroyed according

Confidential
07Y9YV 054WRH

to Sponsor policies and procedures and this destruction will be documented in the biorepository database.

8. Data Security

Databases containing specimen information and test results are accessible only to the authorized Sponsor representatives and the designated study administrator research personnel and/or collaborators. Database user authentication is highly secure, and is accomplished using network security policies and practices based on international standards to protect against unauthorized access.

9. Reporting of Future Biomedical Research Data to Participants

No information obtained from exploratory laboratory studies will be reported to the participant, family, or physicians. Principle reasons not to inform or return results to the participant include: Lack of relevance to participant health, limitations of predictive capability, and concerns regarding misinterpretation.

If important research findings are discovered, the Sponsor may publish results, present results in national meetings, and make results accessible on a public website in order to rapidly report this information to doctors and participants. Participants will not be identified by name in any published reports about this study or in any other scientific publication or presentation.

10. Future Biomedical Research Study Population

Every effort will be made to recruit all participants diagnosed and treated on Sponsor clinical studies who are not Native American participants enrolled via the CAIH for future biomedical research.

11. Risks Versus Benefits of Future Biomedical Research

For future biomedical research, risks to the participant have been minimized and are described in the Future Biomedical Research informed consent.

The Sponsor has developed strict security, policies, and procedures to address participant data privacy concerns. Data privacy risks are largely limited to rare situations involving possible breach of confidentiality. In this highly unlikely situation, there is risk that the information, like all medical information, may be misused.

12. Questions

Any questions related to the future biomedical research should be emailed directly to clinical.specimen.management@merck.com.

13. References

1. National Cancer Institute [Internet]: Available from https://www.cancer.gov/publications/dictionaries/cancer-terms?cdrid=45618

07Y9YV 054WRH
Confidential

2. International Conference on Harmonization [Internet]: E15: Definitions for Genomic Biomarkers, Pharmacogenomics, Pharmacogenetics, Genomic Data and Sample Coding Categories. Available from http://www.ich.org/products/guidelines/efficacy/efficacy-single/article/definitions-for-genomic-biomarkers-pharmacogenomics-pharmacogenetics-genomic-data-and-sample-cod.html
3. Industry Pharmacogenomics Working Group [Internet]: Understanding the Intent, Scope and Public Health Benefits of Exploratory Biomarker Research: A Guide for IRBs/IECs and Investigational Site Staff. Available at http://i-pwg.org/
4. Industry Pharmacogenomics Working Group [Internet]: Pharmacogenomics Informational Brochure for IRBs/IECs and Investigational Site Staff. Available at http://i-pwg.org/

Confidential

10.7 Appendix 7: Country-specific Requirements

10.7.1 Country-specific Request from Ministry of Healthcare of the Russian Federation

The Russian Ministry of Healthcare Ethics Committee requested that a summary of the relevant human clinical studies of V114 be included in the protocol and that additional rationale for the sequential vaccination schedule be provided. Information to address these concerns is provided in this section. Additional information related to the safety and immunogenicity of V114 is also provided in the IB as stated in Section 2.2.2 (Preclinical and Clinical Studies).

10.7.1.1 Clinical Experience With V114

The investigational 15-valent PCV (V114) was evaluated in 8 Phase 1 and Phase 2 clinical studies in immunocompetent adults and children (Table 10). Results of the completed Phase 1 and Phase 2 studies show that V114 displays acceptable safety and tolerability profiles in immunocompetent adults and children as well as induces serotype-specific IgG and opsonophagocytic antibodies that are comparable with the registered vaccine Prevnar 13™ for the 13 common serotypes. Study results are presented in the IB (Section 5.1 – Table 23; Section 5.3.2 – Immunogenicity; and Section 5.4 – Safety).

Table 10 Completed Clinical Studies in Immunocompetent Volunteers

**Protocol**	**Phase**	**Population**	
V114-001	1	Healthy immunocompetent adults 18 to 45 y.o. and children 12 to 15 month of age	Safety, tolerability, and immunogenicity study of V114 compared to Prevnar 13™
V114-002	2	Healthy immunocompetent adults ≥50 y.o.	Safety, tolerability, and immunogenicity study of V114 compared to PNEUMOVAX™23 and Prevnar 13™
V114-003	1-2	Healthy immunocompetent children 6 to 12 weeks of age	Safety, tolerability, and immunogenicity study of V114 compared to Prevnar 13™
V114-004	1-2	Healthy immunocompetent adults 18 to 49 y.o. and children 6 to 12 weeks of age	Safety, tolerability, and immunogenicity study of 5 different lots of V114
V114-005	1-2	Healthy immunocompetent adults 18 to 49 y.o. and children 6 to 12 weeks of age	Safety, tolerability, and immunogenicity study of 4 different lots of V114
V114-006	1-2	Healthy immunocompetent adults ≥50 y.o.	Safety, tolerability, and immunogenicity study of V114 compared to Prevnar 13™
V114-007	2	Healthy immunocompetent adults ≥65 y.o. previously vaccinated with PNEUMOVAX™23	Safety, tolerability, and immunogenicity study of V114 compared to Prevnar 13™
V114-015	1	Healthy immunocompetent Japanese adults from 50 to 64 y.o. and ≥65 y.o.	Safety, tolerability, and immunogenicity study of V114 compared to Prevnar 13™

y.o. = years old.

Confidential
07Y9YV 054WRH

Descriptive post-hoc subgroup analyses for safety and immunogenicity results were conducted for adults with chronic medical conditions such as diabetes, chronic heart disease, and COPD who participated in protocols V114-006 and V114-007. These analyses showed that serotype-specific IgG GMCs and OPA GMTs were generally comparable between participants with or without these chronic conditions. In addition, for each comorbid condition, serotype-specific IgG GMCs and OPA GMTs were comparable between recipients of V114 and PCV13 for the shared serotypes [Peterson, J., et al 2018]. In V114-006, the safety profile of V114 was generally comparable for individuals with or without these chronic conditions (Table 11) and safety was comparable to PCV13. The observed differences in the proportions of subjects with each specific comorbid condition who reported these AEs must be interpreted with caution given the difference in the number of subjects in each category and small number of subjects in some categories.

Table 11 V114-006: Safety Profile of V114 in Study Participants With and Without Specific Comorbid Conditions

	**Comorbid Condition**			
	**None %**	**COPD %**	**Diabetes %**	**Heart Disease %**
Participants in population with follow-up	N=101	N=28	N=40	N=110
**With 1 or more adverse events (Study Duration)**	**74.3**	**71.4**	**62.5**	**67.3**
Injection site	71.3	67.9	57.5	57.3
Non-injection site	46.5	46.4	27.5	36.4
Non-injection site, vaccine related	36.6	28.6	15.0	27.3
**Solicited injection site adverse events (Day 1-Day 5)**	**70.3**	**67.9**	**55.0**	**55.5**
Erythema	13.9	0.0	10.0	10.0
Pain	66.3	60.7	52.5	51.8
Swelling	20.8	17.9	15.0	11.8
**Solicited systemic adverse events (Day 1- Day14)**	**43.6**	**39.3**	**22.5**	**31.8**
Fatigue	20.8	14.3	2.5	15.5
Arthralgia	7.9	13.6	5.0	8.2
Myalgia	22.8	14.3	17.5	15.5
Headache	13.9	17.9	7.5	10.0

COPD = chronic obstructive pulmonary disease.

10.7.1.2 Rationale for Testing V114 in Study Population With a Sequential Vaccination Regimen

Adults living with chronic heart disease, chronic lung disease, diabetes, chronic liver disease, asthma, or smokers are at increased risk for pneumococcal disease compared with healthy individuals living without these conditions [Weycker, D., et al 2016] [Shea, K. M., et al 2014]. A substantial proportion of these individuals have more than 1 of the at-risk conditions, which is known to increase their risk to a level comparable to

Confidential
07Y9YV 054WRH

immunocompromised (high-risk) individuals. Adults with ≥2 concurrent comorbid conditions had pneumococcal disease incidence rates that were as high as or higher than rates observed in those with traditional high-risk conditions [Pelton, S. I., et al 2015].

This study is designed to investigate the safety and immunogenicity of the investigational V114 in adults who are immunocompetent but are at increased risk for pneumococcal disease due an underlying chronic medical condition (asthma, COPD, chronic liver disease with cirrhosis, or chronic heart failure) or due to environmental or behavioral factors such as smoking or alcohol use. The study design allows to enroll into specific strata those participants who have more than 1 underlying risk condition with an associated higher risk for pneumococcal disease.

Pneumococcal vaccination is a very important preventative intervention for this population and it is necessary to select a vaccination strategy that induces robust immune responses while providing the broadest possible serotype coverage. A sequential vaccination regimen of PCV13 followed by the 23-valent pneumococcal polysaccharide vaccine PNEUMOVAX™23 (PPV23) provides optimal protection against most of the serotypes causing pneumococcal disease in children and adults. Such sequential administration has been recommended by several policy bodies and professional societies for pneumococcal vaccination of adults at high risk for pneumococcal disease due to age-associated immunosenescence or due to underlying chronic medical conditions [Castiglia, P. 2014] [Protasov, A. D., et al 2018].

In the United States, PPV23 is recommended by the ACIP for routine use in immunocompetent adults ≥65 years of age as sequential administration at least 1 year after PCV13 [Kobayashi, M., et al 2015]. The recommended interval between PCV13 and PPV23 is shorter for immunocompromised adults 19 years of age and older (≥8 weeks) in order to broaden the serotype coverage to the additional serotypes contained in PPV23 sooner [Kobayashi, M., et al 2015]. PPV23 is also recommended for use in adults 19 to 64 years of age with underlying medical conditions that increase the risk for serious pneumococcal infection. These conditions include but are not limited to chronic heart disease, chronic lung disease (including COPD, emphysema, and asthma), diabetes mellitus, alcoholism, chronic liver disease (including cirrhosis), and cigarette smoking [Centers for Disease Control and Prevention 2010].

This study proposes a 6-month interval between PCV13 and PPV23 for the target population of adults with at-risk chronic conditions. This interval is mid-way between the interval used for high-risk individuals (8 weeks) and that used for healthy immunocompetent older adults without these conditions (12 months). A dosing interval of 6 to 12 months between PCV13 and PPV23 is based on limited clinical data showing that a dosing interval of 6 months was associated with fewer injection site AEs than a 2-month interval, with no differences in the frequencies of systemic AEs or vaccine-induced immune responses [Miernyk, K. M., et al 2009]. This vaccination regimen is in line with the Russian local Clinical Guidelines "Vaccine Prophylaxis of Pneumococcal Disease", issued in 2015 and recognized by the Ministry of Healthcare of Russian Federation, which recommends that adults living with chronic medical conditions that increase the risk of pneumococcal disease, such as those

Confidential

07Y9YV 054WRH

conditions studied in V114-017, receive a first dose of PCV13 followed by a dose of PPV23 6 to 12 months later.

Because the recommendation of health authorities is to vaccinate at-risk individuals such as those included in V114-017 with PCV13 followed 6 to 12 months later with PPV23, the experimental design of the V114-017 clinical study to assess safety, tolerability, and immunogenicity of participants that receive the recommended regimen in a double-blind randomized study with participants that receive the experimental regimen of V114 is justified.

As a pneumococcal vaccination regimen of PCV13 followed by PPV23 is part of national pneumococcal vaccination recommendations for several at-risk and high-risk populations, clinical staff and participants will be familiar with the approach of a sequential vaccine administration. The controlled setting of a randomized, double-blinded clinical study with a central randomization procedure will also ensure that the correct study treatment will be made available to each participant at each study visit.

A subset of participants will receive the standard of care (PCV13) and a subset of participants will receive the experimental V114, which is expected to provide comparable immune responses to PCV13 for the shared serotypes while providing additional coverage for the 22F and 33F serotypes. The potential benefit assessed by immunogenicity will be weighed against the risk assessed by safety and tolerability.

Considering the substantial data from completed clinical studies in adults as well as the vulnerable population of children in which the observed safety and tolerability profiles of V114 are acceptable, MSD considers a Phase 3 clinical study of V114 or active comparator administered in a sequential vaccination regimen with PPV23 to adults at increased risk for IPD due to chronic medical conditions, behavioral or environmental factors, to be safe and important.

Confidential
07Y9YV 054WRH

10.8 Appendix 8: Abbreviations

Abbreviation	Expanded Term
ACIP	Advisory Committee on Immunization Practices
AE	adverse event
ALT	alanine transaminase
APaT	All Participants as Treated
APRI	AST:platelet ratio index
AST	aspartate transaminase
β-hCG	beta-human chorionic gonadotropin
BNP	brain natriuretic peptide
CAIH	Center for American Indian Health
CAPiTA	Community-Acquired Pneumonia Immunization Trial in Adults
CI	confidence interval
cLDA	constrained longitudinal data analysis
CSR	Clinical Study Report
DMC	Data Monitoring Committee
DNA	deoxyribonucleic acid
ECI	event of clinical interest
ECL	electrochemiluminescence
eCRF	electronic case report form
EDC	electronic data collection
EF	ejection fraction
EMA	European Medicines Agency
EOC	Executive Oversight Committee
eVRC	electronic Vaccination Report Card
FAS	Full Analysis Set
FDAAA	Food and Drug Administration Amendments Act
FEV_1	forced expiratory volume in 1 second
FSH	follicle-stimulating hormone
GCP	Good Clinical Practice
GMC	Geometric Mean Concentration
GMFR	Geometric Mean Fold Rise
GMT	Geometric Mean Titer
HIV	Human Immunodeficiency Virus
HRT	hormonal replacement therapy
IB	Investigator’s Brochure
ICF	Informed Consent Form
ICH	International Council for Harmonisation Good Clinical Practice
IEC	Independent Ethics Committee
IgG	Immunoglobulin G
IMP	investigational medicinal product
IRB	Institutional Review Board
IPD	Invasive Pneumococcal Disease
IRT	interactive response technology
IUD	intrauterine device
MOPA	Multiplexed Opsonophagocytic Assay
MSD	Merck Sharp & Dohme Corp.
NIMP	non-investigational medicinal product
NP	nasopharyngeal
NSAE	nonserious adverse event
NYHA	New York Heart Association

Abbreviation	Expanded Term
OP	oropharyngeal
OPA	opsonophagocytic activity
PCV	pneumococcal conjugate vaccine
PCV13	Prevnar 13™
PE	physical examination
PnECL	pneumococcal electrochemiluminescence
PP	Per-Protocol
RNA	ribonucleic acid
SAE	serious adverse event
SLAB	supplemental laboratory test
SoA	schedule of activities
S. pneumoniae	*Streptococcus pneumoniae*
TEE	transesophageal echocardiography
TTE	transthoracic echocardiography
UAD	urine antigen detection assay
ULN	upper limit of normal
US	United States
VRC	Vaccination Report Card
WHO	World Health Organization
WOCBP	woman/women of childbearing potential

07Y9YV 054WRH Confidential

11 REFERENCES

[Anttila, M., et al 1999]	Anttila M, Voutilainen M, Jäntti V, Eskola J, Käyhty H. Contribution of serotype-specific IgG concentration, IgG subclasses and relative antibody avidity to opsonophagocytic activity against Streptococcus pneumoniae. Clin Exp Immunol 1999;118(3):402-7.	03QY70
[Australian Government Department of Health 2017]	Australian Government Department of Health. The Australian immunisation handbook: 4.13 pneumococcal disease [Internet]. 10th edition. Australia: Australian Government Department of Health; 2017 Aug. 21 p. Available from: http://immunise.health.gov.au/internet/immunise/publishing.nsf/Content/9234F08B6DE9BB34CA257D4D00234E27/$File/4%2013_Pneumococcal.pdf.	04VSW6
[Bhatty, M., et al 2011]	Bhatty M, Pruett SB, Swiatlo E, Nanduri B. Alcohol abuse and Streptococcus pneumoniae infections: consideration of virulence factors and impaired immune responses. Alcohol. 2011;45:523-39.	04WB7Q
[Bonten, M. J., et al 2015]	Bonten MJ, Huijts SM, Bolkenbaas M, Webber C, Patterson S, Gault S, et al. Polysaccharide conjugate vaccine against pneumococcal pneumonia in adults. N Engl J Med. 2015 Mar 19;372(12):1114-25.	04NFHD
[Burton, Robert L. and Nahm, Moon H. 2006]	Burton RL, Nahm MH. Development and validation of a fourfold multiplexed opsonization assay (MOPA4) for pneumococcal antibodies. Clin Vaccine Immunol 2006;13(9):1004-9.	03QT2R
[Castiglia, P. 2014]	Castiglia P. Recommendations for pneumococcal immunization outside routine childhood immunization programs in Western Europe. Adv Ther. 2014 Oct 10;31:1011-44.	04VSVW

Confidential

07Y9YV 054WRH

[Center for Disease Control 1978]	CDC. Recommendation of the Public Health Service Advisory Committee on Immunization Practices: Pneumococcal polysaccharide vaccine. MMWR Morb Mortal Wkly Rep. 1978 Jan 27;27(4):25-32.	04VSJL
[Center for Disease Control and Prevention 2012]	Center for Disease Control and Prevention. Geographic differences in HIV infection among hispanics or latinos - 46 states and Puerto Rico, 2010. MMWR 2012;61(40):805-24.	03RS5J
[Centers for Disease Control and Prevention (CDC) 2010]	Centers for Disease Control and Prevention (CDC). Multiple-serotype Salmonella gastroenteritis outbreak after a reception --- Connecticut, 2009. MMWR Morb Mortal Wkly Rep. 2010 Sep 3;59(34):1093-7.	04NJRK
[Centers for Disease Control and Prevention 1997]	CDC. Prevention of pneumococcal disease: recommendations of the Advisory Committee on Immunization Practices (ACIP). MMWR Morb Mortal Wkly Rep. 1997;46(RR-8):1-24.	04VSTK
[Centers for Disease Control and Prevention 2008]	Centers for Disease Control and Prevention (CDC). Invasive pneumococcal disease in children 5 years after conjugate vaccine introduction-eight states,1998-2005. MMWR Morb Mortal Wkly Rep. 2008 Feb 15;57(6):144-8.	04KW8S
[Centers for Disease Control and Prevention 2010]	Centers for Disease Control and Prevention. Prevention of Pneumococcal Disease Among Infants and Children - Use of 13-Valent Pneumococcal Conjugate Vaccine and 23-Valent Pneumococcal Polysaccharide Vaccine; Recommendations of the Advisory Committee on Immunization Practices (ACIP). MMWR 2010;59(RR-11):1-19.	03RSB6
[Centers for Disease Control and Prevention 2010]	Centers for Disease Control and Prevention. Updated recommendations for prevention of invasive pneumococcal disease among adults using the 23-valent pneumococcal polysaccharide vaccine (PPSV23). MMWR 2010;59(34):1102-6.	03RCFX

[Centers for Disease Control and Prevention 2015]	Centers for Disease Control and Prevention. Epidemiology and prevention of vaccine-preventable diseases. 13th ed. Hamborsky J, Kroger A, Wolfe S, editors. Washington (DC): Department of Health and Human Services (HHS); c2015. Chapter 6, Vaccine administration; p. 79-106.	0508PV
[Clopper, C. J. and Pearson, E. S. 1934]	Clopper CJ, Pearson ES. The use of confidence or fiducial limits illustrated in the case of the binomial. Biometrika 1934;26(4):404-13.	03Q0LW
[Curcio, D., et al 2015]	Curcio D, Cane A, Isturiz R. Redefining risk categories for pneumococcal disease in adults: critical analysis of the evidence. Int J Infect Dis. 2015;37:30-5.	04VSHL
[Esposito, S., et al 2004]	Esposito S, Bosis S, Colombo R, Carlucci P, Faelli N, Fossali E, et al. Evaluation of rapid assay for detection of Streptococcus pneumoniae urinary antigen among infants and young children with possible invasive pneumococcal disease. Pediatr Infect Dis J. 2004 Apr;23(4):365-7.	04VSTS
[European Centre for Disease Prevention and Control 2016]	European Centre for Disease Prevention and Control. Annual Epidemiological Report 2016 – Invasive pneumococcal disease. [Internet]. Stockholm: ECDC; 2016. Available from: http://ecdc.europa.eu/en/healthtopics/pneumococcal_infection/Pages/Annual-epidemiological-report-2016.aspx.	04LP3M
[Falkenhorst, G., et al 2017]	Falkenhorst G, Remschmidt C, Harder T, Hummers-Pradier E, Wichmann O, Bogdan C. Effectiveness of the 23-valent pneumococcal polysaccharide vaccine (PPV23) against pneumococcal disease in the elderly: systematic review and meta-analysis. PLoS One. 2017 Jan 6;12(1):e0169368.	04VSWH

Confidential
07Y9YV 054WRH

[Farrell, D. J, et al 2007]	Farrell DJ, Klugman KP, Pichichero M. Increased antimicrobial resistance among nonvaccine serotypes of Streptococcus pneumoniae in the pediatric population after the introduction of 7-valent pneumococcal vaccine in the United States. Pediatr Infect Dis J. 2007 Feb;26(2):123-8.	04KWD9
[French High Council for Public Health 2017]	French High Council for Public Health. Recommendations for vaccination against invasive pneumococcal disease in adults. 2017.	04VYX3
[Gillis, H. D., et al 2017]	Gillis HD, Lang ALS, ElSherif M, Martin I, Hatchette TF, McNeil SA, et al. Assessing the diagnostic accuracy of PCR-based detection of Streptococcus pneumoniae from nasopharyngeal swabs collected for viral studies in Canadian adults hospitalised with community-acquired pneumonia: a Serious Outcomes Surveillance (SOS) Network of the Canadian Immunization Research (CIRN) study. BMJ Open. 2017;7:e015008.	04VSWN
[Grijalva, C. G., et al 2015]	Grijalva CG, Wunderink RG, Zhu Y, Williams DJ, Balk R, Fakhran S, et al. In-hospital pneumococcal polysaccharide vaccination is associated with detection of pneumococcal vaccine serotypes in adults hospitalized for community-acquired pneumonia. Open Forum Infect Dis. 2015:2(4):ofv135.	04VT53
[Guevara, M., et al 2016]	Guevara M, Barricarte A, Torroba L, Herranz M, Gil-Setas A, Gil F, et al. Direct, indirect and total effects of 13-valent pneumococcal conjugate vaccination on invasive pneumococcal disease in children in Navarra, Spain, 2001 to 2014: cohort and case-control study. Euro Surveill. 2016;21(14).	04KSQ3
[Huijts, S. M., et al 2017]	Huijts SM, van Werkhoven CH, Bolkenbaas M, Grobbee DE, Bonten MJM. Post-hoc analysis of a randomized controlled trial: diabetes mellitus modifies the efficacy of the 13-valent pneumococcal conjugate vaccine in elderly. Vaccine. 2017;35:4444-9.	04VSYC

Confidential
07Y9YV 054WRH

[Jackson, L. A., et al 2013]	Jackson LA, Gurtman A, van Cleeff M, Jansen KU, Jayawardene D, Devlin C, et al. Immunogenicity and safety of a 13-valent pneumococcal conjugate vaccine compared to a 23-valent pneumococcal polysaccharide vaccine in pneumococcal vaccine-naive adults. Vaccine. 2013;31:3577-84.	04VT2S
[Jokinen, J., et al 2015]	Jokinen J, Rinta-Kokko H, Siira L, Palmu AA, Virtanen MJ, Nohynek H, et al. Impact of ten-valent pneumococcal conjugate vaccination on invasive pneumococcal disease in Finnish children a population-based study. PLoS One. 2015 Mar 17;10(3):e0120290.	04KW7F
[Knoll, M. D., et al 2016]	Knoll MD, Pride MW, Sebastian S, Hilton B, Isturiz R, Jansen KU. Pilot evaluation of a quantitative serotype-specific urine antigen detection (SS-UAD) assay to identify pneumococcal pneumonia in children <5 years [abstract]. Presented at: International Symposium on Pneumococci and Pneumococcal Diseases Conference; 2016 Jun 26-30; Glasgow, Scotland. Abstract No. 499.	04VT6W
[Kobayashi, M., et al 2015]	Kobayashi M, Bennett NM, Gierke R, Almendares O, Moore MR, Whitney CG, et al. Intervals Between PCV13 and PPSV23 Vaccines: Recommendations of the Advisory Committee on Immunization Practices (ACIP). MMWR Morb Mortal Wkly Rep. 2015 Sep 4;64(34):944-7.	04P7LH
[Lepoutre, A., et al 2015]	Lepoutre A, Varon E, Georges S, Dorleans F, Janoir C, Gutmann L, et al. Impact of the pneumococcal conjugate vaccines on invasive pneumococcal disease in France, 2001-2012. Vaccine. 2015 Jan 3;33(2):359-66.	04KW88
[Lexau, C. A., et al 2005]	Lexau CA, Lynfield R, Danila R, Pilishvili T, Facklam R, Farley MM, et al. Changing epidemiology of invasive pneumococcal disease among older adults in the era of pediatric pneumococcal conjugate vaccine. JAMA 2005;294(16):2043-51.	03RBPW

Confidential

[Liang, K-Y and Zeger, S. L. 2000]	Liang K-Y, Zeger SL. Longitudinal data analysis of continuous and discrete responses for pre-post designs. Sankyha: The Indian Journal of Statistics 2000;62(Series B, Part 1):134-48.	00V5V6
[Martinelli, D., et al 2014]	Martinelli D, Pedalino B, Cappelli MG, Caputi G, Sallustio A, Fortunato F, et al Towards the 13-valent pneumococcal conjugate universal vaccination: effectiveness in the transition era between PCV7 and PCV13 in Italy, 2010-2013. Hum Vaccin Immunother. 2014;10(1):33-9.	04KW8B
[McMahon, B. J., et al 1993]	McMahon BJ, Parkinson AJ, Bulkow L, Davidson M, Wainwright K, Wolfe P, et al. Immunogenicity of the 23-valent pneumococcal polysaccharide vaccine in Alaska Native chronic alcoholics compared with nonalcoholic Native and non-Native controls. Am J Med. 1993 Dec;95:589-94.	04WB7S
[Metlay, J. P., et al 2006]	Metlay JP, Fishman NO, Joffe M, Edelstein PH. Impact of pediatric vaccination with pneumococcal conjugate vaccine on the risk of bacteremic pneumococcal pneumonia in adults. Vaccine 2006;24:468-75.	03RC46
[Miernyk, K. M., et al 2009]	Miernyk KM, Butler JC, Bulkow LR, Singleton RJ, Hennessy TW, Dentinger CM, et al. Immunogenicity and reactogenicity of pneumococcal polysaccharide and conjugate vaccines in Alaska Native adults 55-70 years of age. Clin Infect Dis 2009;49(2):241-8.	03QXDG
[Moore, M. R., et al 2015]	Moore MR, Link-Gelles R, Schaffner W, Lynfield R, Lexau C, Bennett NM, et al. Effect of use of 13-valent pneumococcal conjugate vaccine in children on invasive pneumococcal disease in children and adults in the USA: analysis of multisite, population-based surveillance. Lancet Infect Dis. 2015 Feb 3. [Epub ahead of print].	043MRP

Confidential
07Y9YV 054WRH

[Morton, J. B., et al 2017]	Morton JB, Morrill HJ, LaPlante KL, Caffrey AR. Risk stacking of pneumococcal vaccination indications increases mortality in unvaccinated adults with Streptococcus pneumoniae infections. Vaccine. 2017;35:1692-7.	04VSTC
[Palmu, A. A., et al 2015]	Palmu AA, Kilpi TM, Rinta-Kokko H, Nohynek H, Toropainen M, Nuorti JP, et al. Pneumococcal conjugate vaccine and clinically suspected invasive pneumococcal disease. Pediatrics. 2015 Jul;136(1):e22-7.	04KVRL
[Pelton, S. I., et al 2015]	Pelton SI, Shea KM, Weycker D, Farkouh RA, Strutton DR, Edelsberg J. Rethinking risk for pneumococcal disease in adults: the role of risk stacking. Open Forum Infect Dis. 2015;2(1):ofv020.	050XJ0
[Peterson, J., et al 2018]	Peterson J, Julien K, Rosen J, Stacey H, Williams-Diaz A, Gakhar V, et al. Impact of comorbid conditions on immunogenicity of a new formulation of 15-valent pneumococcal conjugate vaccine in adults 50 years of age and older. Poster session presented at: 11th International Symposium on Pneumococci and Pneumococcal Diseases (ISPPD); 2018 Apr 15-19; Melbourne (Australia).	054RWK
[Pilishvili, T., et al 2014]	Pilishvili T, Ahmed s, Xing W, Farley M, Schaffner W, Thomas A, et al. Impact of 13-valent pneumococcal conjugate vaccine (PCV13) in the U.S. on invasive pneumococcal disease (IPD) among adults with chronic conditions [abstract]. National center for Immunization and Respiratory Disease, Division of Bacterial Diseases, Department of Health and Human Services USA. Report No. ISPPD-0638.	04VT67
[Pilishvili, Tamara, et al 2010]	Pilishvili T, Lexau C, Farley MM, Hadler J, Harrison LH, Bennett NM, et al. Sustained reductions in invasive pneumococcal disease in the era of conjugate vaccine. J Infect Dis 2010;201(1):32-41.	03R5S4

Confidential

07Y9YV 054WRH

[Priner, M., et al 2008]	Priner M, Cornillon C, Forestier D, Valero S, Paccalin M. Might Streptococcus pneumoniae urinary antigen test be positive because of pneumococcal vaccine? J Am Geriatr Soc. 2008 Jan;56(1):170-1.	04VSP8
[Protasov, A. D., et al 2018]	Protasov AD, Zhestkov AV, Kostinov MP, Korymasov EA, Shteyner ML, Tezikov YV, et al. Long-term clinical efficacy and a possible mechanism of action of different modes of pneumococcal vaccination in asthma patients. Pulmonology. 2018;28(2):193-9.	054VGL
[Romero-Steiner, S., et al 1997]	Romero-Steiner S, Libutti D, Pais LB, Dykes J, Anderson P, Whitin JC, et al. Standardization of an opsonophagocytic assay for the measurement of functional antibody activity against streptococcus pneumoniae using differentiated HL-60 cells. Clin Diagn Lab Immunol 1997;4(4):415-22.	03NWQ5
[Ruckinger, S., et al 2009]	Ruckinger S, van der Linden M, Reinert RR, von Kries R, Burckhardt F, Siedler A. Reduction in the incidence of invasive pneumococcal disease after general vaccination with 7-valent pneumococcal conjugate vaccine in Germany. Vaccine 2009;27:4136-41.	03QYQQ
[Shea, K. M., et al 2014]	Shea KM, Edelsberg J, Weycker D, Farkouh RA, Strutton DR, Pelton SI. Rates of pneumococcal disease in adults with chronic medical conditions. Open Forum Infect Dis. 2014 May 27;1(1):ofu024.	04NK7X
[Tomczyk, S., et al 2014]	Tomczyk S, Bennett NM, Stoecker C, Gierke R, Moore MR, Whitney CG, et al. Use of 13-valent pneumococcal conjugate vaccine and 23-valent pneumococcal polysaccharide vaccine among adults aged is greater than or equal to 65 years: recommendations of the Advisory Committee on Immunization Practices (ACIP). MMWR Morb Mortal Wkly Rep. 2014 Sep 19;63(37):822-5.	040XNF

Confidential

07Y9YV 054WRH

[Torres, A., et al 2015]	Torres A, Blasi F, Dartois N, Akova M. Which individuals are at increased risk of pneumococcal disease and why? Impact of COPD, asthma, smoking, diabetes, and/or chronic heart disease on community-acquired pneumonia and invasive pneumococcal disease. Thorax. 2015:70:984-9.	04VSHQ
[Turner, P., et al 2011]	Turner P, Turner C, Kaewcharernnet N, Mon NY, Goldblatt D, Nosten F. A prospective study of urinary pneumococcal antigen detection in healthy Karen mothers with high rates of pneumococcal nasopharyngeal carriage. BMC Infect Dis. 2011;11:108.	04VSPG
[U.S. Food and Drug Administration 2009]	U.S. Food and Drug Administration (CDER, CBER, CDRH). Guidance for industry patient-reported outcome measures: use in medical product development to support labeling claims [Internet]. Washington: U.S. Department of Health and Human Services; 2009. Available from: https://www.fda.gov/downloads/drugs/guidances/ucm193282.pdf	04MG9J
[Wagenvoort, G. H., et al 2016]	Wagenvoort GH, Knol MJ, de Melker HE, Vlaminckx BJ, van der Ende A, Rozenbaum MH, et al. Risk and outcomes of invasive pneumococcal disease in adults with underlying conditions in the post-PCV7 era, The Netherlands. Vaccine. 2016 Jan 12;34(3):334-40.	04KTDB
[Waight, P. A., et al 2015]	Waight PA, Andrews NJ, Ladhani SN, Sheppard CL, Slack MP, Miller E. Effect of the 13-valent pneumococcal conjugate vaccine on invasive pneumococcal disease in England and Wales 4 years after its introduction: an observational cohort study. Lancet Infect Dis. 2015 May;15(5):535-43.	04KTF2
[Watt, J. P., et al 2007]	Watt JP, O'Brien KL, Benin AL, McCoy SI, Donaldson CM, Reid R, et al. Risk factors for invasive pneumococcal disease among Navajo adults. Am J Epidemiol. 2007;166(9):1080-7.	04VSPB

07Y9YV 054WRH Confidential

[Weatherholtz, R., et al 2010]	Weatherholtz R, Millar EV, Moulton LH, Reid R, Rudolph K, Santosham M, et al. Invasive pneumococcal disease a decade after pneumococcal conjugate vaccine use in an American Indian population at high risk for disease. Clin Infect Dis. 2010 May 1;50(9):1238-46.	04VSP6
[Weiss, S., et al 2015]	Weiss S, Falkenhorst G, van der Linden M, Imohl M, von Kries R. Impact of 10- and 13-valent pneumococcal conjugate vaccines on incidence of invasive pneumococcal disease in children aged under 16 years in Germany, 2009 to 2012. Euro Surveill. 2015 Mar 12;20(10):21057.	04KTFC
[Weycker, D., et al 2016]	Weycker D, Farkouh RA, Strutton DR, Edelsberg J, Shea KM, Pelton SL. Rates and costs of invasive pneumococcal disease and pneumonia in persons with underlying medical conditions. BMC Health Serv Res. 2016;16:182.	04VSTX
[Weycker, D., et al 2016]	Weycker D, Farkouh RA, Strutton DR, Edelsberg J, Shea KM, Pelton SI. Rates and costs of invasive pneumococcal disease and pneumonia in persons with underlying medical conditions. BMC Health Serv Res. 2016 May 13;16:182.	04NK8F
[Whitney, Cynthia G., et al 2003]	Whitney CG, Farley MM, Hadler J, Harrison LH, Bennett NM, Lynfield R, et al. Decline in invasive pneumococcal disease after the introduction of protein-polysaccharide conjugate vaccine. N Engl J Med 2003;348(18):1737-46.	03QT0D
